# Genomics-Guided Drawing of Molecular and Pathophysiological Components of Malignant Regulatory Signatures Reveals a Pivotal Role in Human Diseases of Stem Cell-Associated Retroviral Sequences and Functionally-Active hESC Enhancers

**DOI:** 10.3389/fonc.2021.638363

**Published:** 2021-03-31

**Authors:** Gennadi V. Glinsky

**Affiliations:** ^1^ Institute of Engineering in Medicine, University of California, San Diego, CA, United States; ^2^ Department of Functional & Translational Genomics, OncoSCAR, Inc., Portland, OR, United States

**Keywords:** malignant regulatory signatures, stem cell-associated retroviral sequences, retrotransposition, human embryogenesis, cancer survival genes, cancer driver genes, multi-lineage markers expressing human embryonic cells

## Abstract

Repetitive DNA sequences (repeats) colonized two-third of human genome and a majority of repeats comprised of transposable genetic elements (TE). Evolutionary distinct categories of TE represent nucleic acid sequences that are repeatedly copied from and pasted into chromosomes at multiple genomic locations and acquired a multitude of regulatory functions. Here, genomics-guided maps of stemness regulatory signatures were drawn to dissect the contribution of TE to clinical manifestations of malignant phenotypes of human cancers. From patients’ and physicians’ perspectives, the clinical definition of a tumor’s malignant phenotype could be restricted to the early diagnosis of sub-types of malignancies with the increased risk of existing therapy failure and high likelihood of death from cancer. It is the viewpoint from which the understanding of stemness and malignant regulatory signatures is considered in this contribution. Genomics-guided analyses of experimental and clinical observations revealed the pivotal role of human stem cell-associated retroviral sequences (SCARS) in the origin and pathophysiology of clinically-lethal malignancies. SCARS were defined as the evolutionary- and biologically-related family of genomic regulatory sequences, the principal physiological function of which is to create and maintain the stemness phenotype during human preimplantation embryogenesis. For cell differentiation to occur, SCARS expression must be silenced and SCARS activity remains repressed in most terminally-differentiated human cells which are destined to perform specialized functions in the human body. Epigenetic reprogramming, de-repression, and sustained activity of SCARS results in various differentiation-defective phenotypes. One of the most prominent tissue- and organ-specific clinical manifestations of sustained SCARS activities is diagnosed as a pathological condition defined by a consensus of morphological, molecular, and genetic examinations as the malignant growth. Here, contemporary evidence are acquired, analyzed, and reported defining both novel diagnostic tools and druggable molecular targets readily amenable for diagnosis and efficient therapeutic management of clinically-lethal malignancies. These diagnostic and therapeutic approaches are based on monitoring of high-fidelity molecular signals of continuing SCARS activities in conjunction with genomic regulatory networks of thousands’ functionally-active embryonic enhancers affecting down-stream phenotype-altering genetic loci. Collectively, reported herein observations support a model of SCARS-activation triggered singular source code facilitating the intracellular propagation and intercellular (systemic) dissemination of disease states in the human body.

## Introduction

Transposable elements (TEs) represent a major evolutionary source of genomic regulatory sequences in mammalian genomes comprising gene promoters and enhancers, splicing and termination sites, and non-coding RNAs (1–4). TE-encoded sequences contribute to regulation of three-dimensional (3D) genome architecture by establishing boundary regions of 3D chromatin folding modules designated topologically-associating domains (5–9). Genomic regulatory sequences derived from species-specific endogenous retroviruses, including Human Endogenous Retroviruses (HERVs) in human genome, have been considered as one of the major sources of these evolutionary innovations establishing species-specific patterns of genomic regulatory networks (GRNs).

TEs and HERVs exert potent regulatory effects in specific types of GRNs governing embryogenesis and early development, pluripotency, pregnancy and placentation, innate immunity, responses to stress, environmental stimuli or infection (10–20), including establishment of human-specific regulatory elements of GRNs (21–31). In addition to regulation of transcription initiation, HERVs and other classes of TEs may also affect splicing, transcriptional termination and mRNA stability. For example, SINE elements located in the 3’ UTR of transcripts promote Staufen-mediated mRNA decay. This strategy to regulate mRNA stability appears evolutionary conserved because it is shared by mice and humans (32).

Activity of endogenous retroviruses and other TEs is suppressed in human cells to restrict the potentially harmful effects of mutations on functional genome integrity and to ensure the maintenance of genomic stability (33–37). Interestingly, recent observations revealed that genomic and epigenetic regulatory mechanisms which emerged during evolution to silence HERVs and other TEs have been repurposed to a numerous other gene expression regulatory functions (22, 38, 39).

Of particular interest are observations of significant correlations of KRAB zinc finger (KZNF) protein binding profiles with brain developmental gene expression patterns across multiple regions of the human brain (38). These findings suggest that KZNF proteins not only bind promoters of TEs and HERVs and repress their expression, but also bind to promoters of many other genes and regulate gene expression in the human brain in a region-specific manner (38). Collectively, these observations support the hypothesis that KZNF proteins and TE-encoded regulatory sequences may have a direct impact on gene expression in the developing human brain and became intrinsically integrated in neuronal genomic regulatory networks of developing and adult human brain. Consistent with this idea, KAP1 (KRAB-associated protein 1), a co-repressor protein responsible for heterochromatin formation at TE-derived loci, is likely to have multiple additional gene regulatory functions because it binds to the transcription start sites of actively transcribed genes, associates with the wide range of nucleic acid-binding proteins, nucleosome remodelers, chromatin state modifiers, and other modulators of transcription (39). Notably, KAP1 is recruited to the actively transcribed RNA polymerase II (RNAPII) promoters and exerts pleomorphic effects on RNAPII activity at promoters of genes with either constitutive or inducible modes of expression (39).

One of the rapidly expanding areas of research is focused on analyses of mechanisms causing dysregulation of HERVs in various pathological conditions and mechanisms by which their aberrant expression may contribute to the pathogenesis of human diseases. Aberrant activities of HERV-encoded regulatory sequences have been implicated in multiple types of human malignancies, autoimmune diseases, as well as neurodegenerative and neurodevelopmental disorders (40–71).

Investigations by numerous laboratories of HERV’s activities in various types of human cancers are accelerating particularly rapidly (40–62). Highly promising new area of research documenting the impacts of aberrant HERV’s activities in human neurodevelopmental disorders, including Autism Spectrum Disorders (ASD) and Attention Deficit Hyperactivity Disorders (ADHD), appears to advance toward discovery of novel therapeutic opportunities (63–70). Overall, experimental and clinical efforts in these areas appear to follow the blueprint of Herve Perron and colleagues pioneering work on discovery and characterization of multiple sclerosis-associated retroviruses (72–81), which underscored significant and multifaceted roles of HERV in human physiology and pathology.

This recent remarkable progress across multiple fields aiming to investigate various aspects of evolutionary origins, biogenesis, molecular biology, physiology, and pathology of TE- and HERV-encoded genomic regulatory sequences has been facilitated by marked advances in analytical, computational, and bioinformatics methodologies as well as CRISPR/Cas9 genome editing and nucleic acid sequencing technologies (55, 82–86). Collectively, these advances enabled the execution of structure-activity activity analyses of TE- and HERV-encoded genomic regulatory sequences at the levels of single cell resolution and individual locus precision. Expression of HERV-encoded regulatory sequences, in particular, HERVH subfamily, is markedly activated in hESCs (11, 25, 27, 87, 88). It has been reported that LTR7/HERVH sequences appear associated with binding sites for pluripotency core transcription factors (11, 25, 87). Functionally-defined categories include human-specific transcription binding sites (TFBS) and long noncoding RNAs (25, 89). Expression of HERVH in hESC is regulated by the pluripotency regulatory circuitry. For example, 80% of long terminal repeats (LTRs) of the 50 most highly expressed HERVH are occupied by pluripotency core transcription factors, including NANOG and POU5F1 (87). HERV-derived sequences (LTR7/HERVH, LTR5_Hs/HERVK) and L1HS, harbor 99.8% of the candidate human-specific regulatory sequences (HSRS) with putative TFBS in the genome of hESC (25). Based on the common functional features of these HERVs mediated by their active expression in the hESC and human embryos (46, 52, 56, 90), they were designated as the endogenous human stem cell-associated retroviral sequences (SCARS).

Epigenetic mechanisms play a crucial role in regulation of expression of HERV-encoded sequences, since the LTR7/HERVH subfamily is rapidly demethylated and upregulated in the blastocyst of human embryos and remains highly expressed in hESC (91). Sequences of LTR7, LTR7B, and LTR7Y, which are typically harbor the promoters for the downstream full-length HERVH-int elements, were found expressed at the highest levels and were the most statistically significantly up-regulated retrotransposons in human ESC and induced pluripotent stem cells, iPSC (92). It has been demonstrated that LTRs of HERVH subfamily, in particular, LTR7, function in hESC as enhancers and HERVH sequences encode nuclear non-coding RNAs, which are required for maintenance of pluripotency and identity of hESC (93). Transient hyper-activation of HERVH is required for reprogramming of differentiated human cells toward induced pluripotent stem cells (iPSC), maintenance of pluripotency and reestablishment of differentiation potential (94). Failure to control the LTR7/HERVH activity leads to the differentiation-defective phenotype in neural lineage (94, 95). Activation of L1 retrotransposons may also contribute to these processes because significant activities of both L1 transcription and transposition were reported in iPSC of humans and other great apes (96). Single-cell RNA sequencing of human preimplantation embryos and embryonic stem cells (97, 98) enabled identification of specific distinct populations of early human embryonic stem cells defined by marked activation of specific retroviral elements (99).

Notably, a sub-population of hESCs and human induced pluripotent stem cells (hiPSCs) with markedly elevated LTR7/HERVH expression manifests key properties of naive-like pluripotent stem cells (100). Furthermore, human naïve-like pluripotent stem cells have been genetically tagged and successfully isolated based on markers of elevated transcription of LTR7/HERVH (96). Embryonic stem cell-specific transcription factors NANOG, POU5F1, KLF4, and LBP9 drive LTR7/HERVH transcription in human pluripotent stem cells (100). Targeted interference with HERVH activity and HERVH-derived transcripts severely compromises self-renewal functions of human pluripotent stem cells (100). Transactivation of LTR5_Hs/HERVK by pluripotency master transcription factor POU5F1 (OCT4) at hypomethylated LTRs representing the evolutionary recent genomic integration sites of HERVK retroviruses induces HERVK expression during human embryogenesis (101). It occurs during embryonic genome activation at the eight-cell stage, continues through the stage of epiblast cells in preimplantation blastocysts, and ceases during hESC derivation from blastocyst outgrowths (101). The presence of HERVK viral-like particles and Gag proteins in human blastocysts has been documented during normal human embryogenesis (101), supporting the idea that endogenous human retroviruses are active and functional during early human embryonic development. It has been observed that overexpression of HERVK virus-accessory protein Rec in pluripotent cells was sufficient to increase the host protein IFITM1 level and inhibit viral infection (101), suggesting that this anti-viral defense mechanism in human early-stage embryos is associated with HERVK activation. Detailed analysis of experimental evidence documenting how activation of retrotransposons orchestrates species-specific gene expression in embryonic stem cells highlighted the fine regulatory balance established during evolution between activation and repression of genomic regulatory sequences derived from specific retrotransposons in human cells (102).

The idea that malignant growth originates from stem cells is more than a quarter century old (103). It was revived at the beginning of 21st century as the cancer stem cell theory (104, 105), which became one of dominant concepts of the contemporary cancer research. One of the key principles of the cancer stem cell theory is that a single cancer stem cell is sufficient to regrow a malignant tumor fully recapitulating morphological, molecular, genomic, and biological features of the parental tumor. Consequently, the theory predicts that cancer cannot be eradicated unless cancer stem cell-targeting therapies (106) will eliminate all cancer stem cells. This postulate is believe to be true because if even a single cancer stem cell would escape the therapeutic assault, it will continue to fuel the malignant growth. However, some fundamental clinical realities seem not necessarily fully compatible with the uniformly simplistic view of the human cancer origin and pathogenesis. First, tumors arising in the same organ are not equivalent in clinical responses to therapies, which could be correlated to their genetic and molecular features. Second, the clinical prognosis related to the organ of cancer origin is markedly different for cancers diagnosed in different organs even at the early stages. Third, in many instances, the clinical cure of malignant tumors has been achieved by the first-line cancer therapies, which are not specifically designed to target cancer stem cells.

On a parallel track, technological advances enabled genome-wide gene expression profiling analyses of human malignancies making a reality the search for gene expression signatures of clinically-lethal malignancies, thus, looking for statistically-significant gene expression correlates of increased likelihood of existing therapy failure and death from cancer. Historically, the theory defining a genomic link between degrees to which a malignancy recapitulates gene expression profiles of stem cells and clinical phenotypes of increased likelihood of therapy failure and death from cancer is originated from the discovery of the death-from-cancer gene expression signature (107). This genomic connectivity between the phenotypes of resemblance to stemness and high likelihood of death from cancer was initially documented for cancer patients diagnosed with 12 distinct types of human malignancies (107). Observations reported in the original contributions (107, 108) and follow-up studies (52, 56, 90, 109) directly implicated sustained activation of the Polycomb Group (PcG) Proteins chromatin silencing pathway (110), specifically, the *BMI1* gene, as the principal genomic contributor defining these associations (11–97, 99–114). Collectively, these observations formed the foundation for a concept stating that malignant clinical behaviors of human cancers are governed by stemness genomic laws (107–109, 111–114). The universal nature of the genomic connectivity between the degree of resemblance to stemness and the extent of malignant behavior of a tumor was validated in numerous experimental cancer models, including transgenic mouse models facilitating implementation of the mouse/human translational genomics approach (107, 115, 116); clinically-relevant orthotopic xenograft models of human cancers and xenograft-derived cancer cell lines, including blood-borne metastasis precursor cells (108, 117–121). Mechanistic roles of genes essential for functional integrity of PcG chromatin silencing pathway were demonstrated using targeted genetic interference approaches (115, 122) and gene-specific small molecule therapeutics (123). Overall, multiple studies have shown that BMI1 inhibition confer therapeutic effects on glioblastoma multiforme, colorectal and breast cancers, as well as chemoresistant ovarian, prostate, pancreatic, and skin cancers (123–126).

However, the major limitation of these and many other early studies was the lack of sufficient understanding of the genomic and molecular underpinning of the stemness phenotype as it emerges during human preimplantation embryogenesis. Remarkable advances in single cell expression profiling analyses of human preimplantation embryos closed this knowledge gap and provided the opportunity to address this limitation. Collectively, these advances facilitated the discovery of stem cell-associated retroviral sequences, which act as the master genomic regulatory elements driving the creation of stemness phenotype in human embryos and may be responsible for stem cell-like features of human malignancies diagnosed in multiple organs.

The term stem cell-associated retroviral sequences (SCARS) refers to the defined set of genomic regulatory sequences sustained expression of which is essential for acquisition and maintenance of stemness phenotype (46. 52, 56, 90). The canonical definition of “stemness” in reference to human Embryonic Stem Cells (hESC), normal stem cells and progenitor cells implies a combination of the phenotypic features of immortality/self-renewal/asymmetric division/pluripotency. Single cell expression profiling-guided deconvolution of a developmental timeline of human preimplantation embryos enabled the discovery of human embryonic Multi-Lineage Markers Expressing cells (MLME cells), emergence of which during human embryogenesis precedes lineage segregation events and subsequent creation of hESC (18). Specific members of SCARS termed human pluripotency-associated transcripts (HPATs) have been implicated in the creation of the MLME cells (18). It has been hypothesized that definition of the “stemness” phenotype for the human MLME cells should be expanded to include the totipotency feature and the human MLME cells could be defined biologically as the pan-lineage precursor cells (18).

For cell differentiation to occur, the expression of SCARS must be silenced: hESC fails to properly differentiate in response to differentiation-inducing cues if SCARS expression is maintained and resulting cells display differentiation-defective phenotypes (94, 95, 127, 128). It has been suggested that de-repression and sustained re-activation of SCARS expression in association with continuous activation of down-stream genomic regulatory targets (collectively defined as activation of SCARS-associated genomic regulatory networks) is the hallmark of therapy-resistant clinically-lethal malignancies with clinical phenotypes of increased risk of therapy failure and high likelihood of death from cancer (46, 52, 56, 90, 109). Evolutionary, SCARS are belong to the exceedingly large class of genomic sequences originated from TEs and comprising more than half of the human genome. Specifically, in hESC and human preimplantation embryos SCARS represent a functionally-related and structurally well-defined sub-set of TE-derived regulatory sequences originated from LTR7/HERV-H, LTR5_Hs/HERV-K, and recently implicated SVA-D retrotransposons (22, 129, 130), the set of which was further narrowed by restrictions to human-specific (unique-to-humans) genomic regulatory sequences (8, 25–30, 46, 52, 56, 90).

A range of genetic, molecular, and functional definitions of SCARS directly linked to a stemness state extends to different classes of regulatory DNA sequences (transcription factor-binding sites; functional enhancer elements; alternative promoters), donors of splicing sites, non-coding RNA molecules, and structural boundary elements of TADs. Precise mapping of individual transcriptionally-active genomic loci which generated RNA molecules from repetitive sequences (repeats), including highly diverse families of TE and HERV-encoded sequences, became possible only recently. Advances in RNAseq technology and bioinformatics approaches to data retrieval, processing, and analyses, including implementation of *de novo* transcriptome assembly protocols, facilitated identification of hundreds thousands of TE-encoded RNA molecules precisely mapped to corresponding transcriptionally active genomic loci in human dorsolateral prefrontal cortex (83) and across the spectrum of all major human cancer types (55). Using the pan-cancer *de novo* transcript assembly approach, the remarkable complexity and ubiquitous nature of transcripts encoded by endogenous retroviral elements (EREs) were uncovered in human malignancies of distinct origins and diverse spectrum of anatomical locations (55). It has been reported that thousands of transcripts overlapping with regulatory long terminal repeats (LTRs) derived from endogenous retroviruses were expressed in a cancer-specific manner in at least one or several related cancer types (55). Several of these cancer-specific LTR-harboring transcripts represent relatively large RNA molecules exceeding 50K nucleotides, perhaps, reflecting the read-through transcriptional activity in cancer cells due to the extensive chromatin reprogramming. Notably, cancer-specific RNA molecules derived from individual SCARS loci representing LTR7/HERV-H and LTR5_Hs/HERV-K families accounted for 31% of all reported cancer-specific LTR element-overlapping transcripts that are expressed in more than one cancer type. These cancer-specific LTR-harboring RNA molecules appear to affect the expression of disease-relevant genes and to produce previously unknown cancer-specific antigenic peptides (55). Therefore, it is now feasible to unequivocally map SCARS-harboring RNA molecules to specific transcriptionally-active genetic loci encoding these transcripts.

## Methods

### Data Source and Analytical Protocols

A total of 94,806 candidate HSRS, including 35,074 neuro-regulatory human-specific SNCs, detailed descriptions of which and corresponding references of primary original contributions are reported elsewhere (6, 18, 25–30, 52, 83, 131). Solely publicly available datasets and resources were used in this contribution. The significance of the differences in the expected and observed numbers of events was calculated using two-tailed Fisher’s exact test. Additional placement enrichment tests were performed for individual classes of HSRS taking into account the size in bp of corresponding genomic regions. Additional details of methodological and analytical approaches are provided in the Supplemental Methods and previously reported contributions (6, 18, 25–30, 52, 83).

### Gene Set Enrichment and Genome-Wide Proximity Placement Analyses

Gene set enrichment analyses were carried-out using the Enrichr bioinformatics platform, which enables the interrogation of nearly 200,000 gene sets from more than 100 gene set libraries. The Enrichr API (January 2018 through January 2020 releases) (132, 133) was used to test genes linked to HSRS of interest for significant enrichment in numerous functional categories. When technically feasible, larger sets of genes comprising several thousand entries were analyzed. Regulatory connectivity maps between HSRS, SCARS and coding genes and additional functional enrichment analyses were performed with the GREAT algorithm (134, 135) at default settings. The reproducibility of the results was validated by implementing two releases of the GREAT algorithm: GREAT version 3.0.0 (2/15/2015 to 08/18/2019) and GREAT version 4.0.4 (08/19/2019). The GREAT algorithm allows investigators to identify and annotate the genome-wide connectivity networks of user-defined distal regulatory loci and their putative target genes. Concurrently, the GREAT algorithm performs functional annotations and analyses of statistical enrichment of annotations of identified genes, thus enabling the inference of potential biological significance of interrogated genomic regulatory networks. Genome-wide Proximity Placement Analysis (GPPA) of distinct genomic features co-localizing with SCARS and HSRS was carried out as described previously and originally implemented for human-specific transcription factor binding sites (6, 18, 25–30, 52, 83).

#### Differential GSEA to Infer the Relative Contributions of Distinct Subsets of Genes on Phenotypes of Interest

When technically and analytically feasible, different sets of differentially-expressed genes (DEGs) defined at multiple significance levels of statistical metrics and comprising from dozens to several thousand individual genetic loci were analyzed using differential GSEA to gain insights into biological effects of DEGs and infer potential mechanisms of anticancer activities. This approach was successfully implemented for identification and characterization of human-specific regulatory networks governed by human-specific transcription factor-binding sites (6, 18, 25–30, 52, 83) and functional enhancer elements (6, 18, 25–28), 13,824 genes associated with 59,732 human-specific regulatory sequences (29), 8,405 genes associated with 35,074 human-specific neuroregulatory single-nucleotide changes (30), as well as human genes and medicinal molecules affecting the susceptibility to SARS-CoV-2 coronavirus (136).

Initial GSEA entail interrogations of each specific set of DEGs and SCARS-regulated genes using 29 distinct genomic databases, including comprehensive pathway enrichment Gene Ontology (GO) analyses. Upon completion, these analyses were followed by in-depth interrogations of the identified significantly-enriched genes employing selected genomic databases deemed most statistically informative at the initial GSEA. In all tables and plots (unless stated otherwise), in addition to the nominal p values and adjusted p values, the “combined score” calculated by Enrichr software is reported, which is a product of the significance estimate and the magnitude of enrichment (combined score c = log(p) ∗ z, where p is the Fisher’s exact test p-value and z is the z-score deviation from the expected rank).

### Statistical Analyses of the Publicly Available Datasets

All statistical analyses of the publicly available genomic datasets, including error rate estimates, background and technical noise measurements and filtering, feature peak calling, feature selection, assignments of genomic coordinates to the corresponding builds of the reference human genome, and data visualization, were performed exactly as reported in the original publications and associated references linked to the corresponding data visualization tracks (http://genome.ucsc.edu/). Any modifications or new elements of statistical analyses are described in the corresponding sections of the Results. Statistical significance of the Pearson correlation coefficients was determined using GraphPad Prism version 6.00 software. Both nominal and Bonferroni adjusted p values were estimated. The significance of the differences in the numbers of events between the groups was calculated using two-sided Fisher’s exact and Chi-square test, and the significance of the overlap between the events was determined using the hypergeometric distribution test (137).

## Results

### Global DNA Methylation Reprogramming and SCARS Activity Contribute to Creation of Telomerase-Positive MLME Cells During Human Preimplantation Embryogenesis

One of the principal molecular functions of activated SCARS is illustrated by their biological activities attributed to non-coding RNA (ncRNA) molecules transcribed from regulatory DNA segments harboring SCARS. Importantly, manifestations SCARS biological activities have been demonstrated for ncRNAs derived from individual genomic loci (46, 52, 56) and in human embryos SCARS activity has been associated with the creation of telomerase-positive cells co-expressing genetic markers of all embryonic lineages (180). These telomerase-positive Multi-Lineage Markers Expressing (MLME) cells have been identified employing single cell expression profiling analyses of viable human blastocysts and hundreds of individual cells recovered from preimplantation human embryos (18, 138, 139). Creation of cells in part resembling gene expression features of MLME cells was recapitulated in genetic engineering experiments, in which individual SCARS-encoded RNAs termed Human Pluripotency-Associated Transcripts (HPATs) were over-expressed in human cells (18, 138, 139). These observations support the hypothesis that SCARS activation in human embryos may have contributed to the creation of MLME cells.

The summary of the multi-step validation protocol of human embryonic Multi-Lineage Markers Expressing (MLME) cells is shown in **Table 1**. The MLME phenotype was assigned to individual telomerase-positive cells that co-expressed at least six genetic markers of the Epiblast (EPI) lineage; seven genetic markers of the Trophectoderm (TE) lineage; and four genetic markers of the Primitive endoderm (PE) lineage; and cells must express all three main master pluripotency transcription factors (*OCT4, NANOG, SOX2*). First, the expression levels of 58 genetic markers of human embryonic lineages were considered individually in a particular single cell by comparing the expression values of the markers in a given cell and the median expression value of the marker in the population of single cells of human embryos as previously reported (18, 140). The marker was considered expressed when the expression value in a cell exceeds the median expression value. The discovery set of 58 genetic markers of human embryonic lineages was utilized in these experiments and based on the above criteria a total of 135 MLME cells were selected from 839 telomerase-positive human embryonic cells. The discovery set of 58 genetic markers of human embryonic lineages was reported elsewhere (18, 141, 142). Next, independent sets of lineage-specific markers comprising of top 100 individual genetic markers for each embryonic lineage were utilized for validation of the MLME phenotype in each individually-selected cell. The validation sets of lineage-specific genetic markers of human embryonic lineages were reported elsewhere (140). To assess the statistical significance of the enrichment of the lineage-specific genetic markers in the MLME cells, p values were estimated using the hypergeometric distribution test. Results of these analyses revealed statistically significant enrichment of genes representing genetic markers of three main embryonic lineages among genes up-regulated in human embryonic MLME cells (**Table 1**). Similar patterns were observed for distinct populations of MLME cells identified in human preimplantation embryos using different approaches (**Supplemental Figure S1**).

**Table 1:** Enrichment of genes comprising top 100 lineage-specific genetic markers of each of three major embryonic lineages of human preimplantation embryos among genes that are significantly up-regulated in the MLME cells.

Classification category	Number of genes	Number of up-regulated genes in the MLME cells	Percent	P value*	Observed/expected ratio**
**Human genome**	26,178	9,430	36.0		
**Genetic markers of the human Epiblast (EPI)**	100	91	91.0	1.186E−30	2.53
**Genetic markers of the human Primitive Endoderm (PE)**	88	41	46.6	0.0107581	1.29
**Genetic markers of the human Trophectoderm (TE)**	100	81	81.0	2.799E−20	2.25

*p values were estimate using the hypergeometric distribution test; **, expected values were estimated based on the number of all analyzed genes (26,178) and the number of genes significantly up-regulated in the human embryonic MLME (9,430); MLME, multi-lineage markers expressing cells; A total of 819 telomerase-positive (TERTpos) individual human embryonic cells were analyzed and each single cell was identified as the putative immortal MLME cell if it expressed genetic markers of each of the three major lineages (epiblast, EPI; throphectoderm, TE; and primitive endoderm, PE) and all three (NANOG; POU5F1; SOX2) pluripotent state master regulators. The MLME phenotype was assigned to individual telomerase-positive cells that co-expressed at least six genetic markers of the EPI lineage; seven genetic markers of the TE lineage; and four genetic markers of the PE lineage; and three main master pluripotency transcription factors. The expression levels of 58 genetic markers of human embryonic lineages were considered individually in a particular single cell by comparing the expression values of the markers in a given cell and the median expression value of the marker in the population of single cells of human embryos as previously reported (18, 140). The marker was considered expressed when the expression value in a cell exceeded the median expression value. The set of 58 genetic markers of human embryonic lineages analyzed in these experiments during the selection a total of 135 MLME cells from 839 TERTpos human embryonic cells is listed in the **Supplemental Table S4** (18) and was originally reported elsewhere (141). Independent sets of lineage-specific markers comprising of top 100 individual genetic markers for each embryonic lineage were utilized for validation of the MLME phenotype and were reported elsewhere (140).

In agreement with the hypothesis that activities of SCARS contribute to creation of MLME cells, SCARS appear to affect expression of two-third of genes (8,374 of 12,735 genes; 66%) expression of which distinguishes MLME cells from other cells in preimplantation human embryos. Notably, SCARS activity affects expression of a dominant majority (84.1%) of genes up-regulated in human embryonic MLME cells, while expression of only a minor fraction of genes down-regulated in MLME cells (13.4%) appears affected by SCARS.

**Figure 1:**
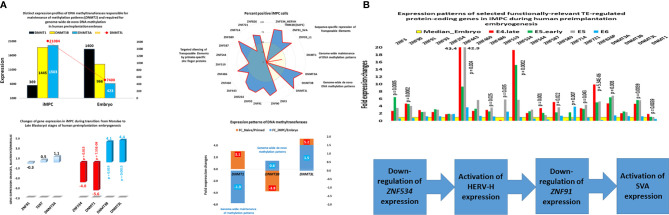
Expression changes of genes encoding DNA methyltransferases and primate-specific zinc finger proteins in human embryonic MLME cells. **(A)** Telomerase-positive MLME cells manifest decreased expression of the *DNMT1* gene, which is responsible for genome-wide maintenance of DNA methylation patterns, and increased expression of genes responsible for genome-wide *de novo* methylation patterns (*DNMT3A, DNMT3B, DNMT3L*). **(B)** Concurrently, MLME cells exhibit decreased expression of primate-specific zinc finger proteins responsible for sequence-specific silencing of SCARS and other TE-harboring loci during human preimplantation embryogenesis. Collectively, these changes of gene expression cause marked reprogramming of DNA methylation patterns in genomes of MLME cells and are associated with activation of SCARS expression. MLME cells are designated as immortal multi-lineage precursor cells, iMPC (18).

**Figure 2:**
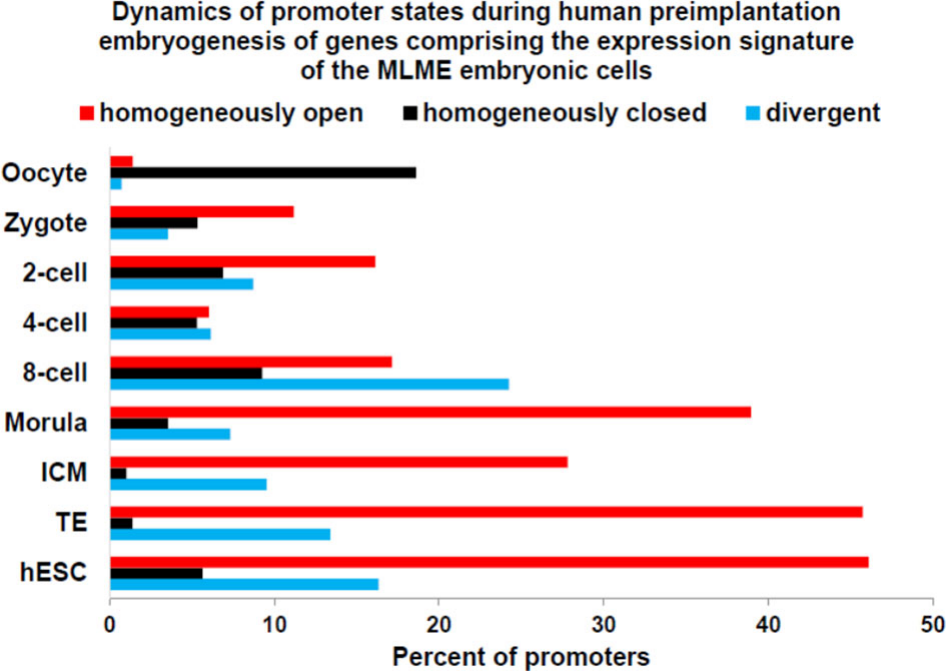
Dynamics of promoter state’s changes of genes comprising GES of human embryonic MLME cells during human preimplantation embryogenesis. Graphs reflect the gradual transition from predominantly homogenously closed (silent) promoter state in the oocyte to predominantly homogenously open (active) promoter state at the morula stage. Homogenously open promoter states of genes comprising the MLME GES (18) are maintained in human embryonic cells of the ICM, TE, and hESC. Divergent promoter state definition refers to a transitional state of partially closed and partially open promoters. Promoter states of human genes at different stages of preimplantation embryogenesis were reported elsewhere (144).

Zygote-to-embryo transition is accompanied by dramatic DNA methylation reprogramming which is governed by the placeholder nucleosome positioning (143). Newly established genome-wide dynamics of the chromatin accessibility landscape and concurrent changes of promoter methylation states affect expression of thousands genes and results in embryonic genome activation (129, 144). Importantly, DNase I hypersensitive site (DHS) sequencing revealed that human transposons SVA and HERV-K harbor DHSs and are highly expressed in early human embryos, but not in differentiated tissues (129). Analyses of genes comprising GES of human embryonic MLME cells revealed that DNA methylation reprogramming may have contributed to the creation and maintenance of the MLME phenotype in human preimplantation embryos (**Figure 1**). Collectively, observed in MLME cells gene expression changes of methyltransferases would cause marked reprogramming of genome-wide DNA methylation profiles by erasing the pre-existing cytosine methyl marks and establishing *de novo* methylation patterns (**Figure 1A**). Concurrently diminished expression of genes encoding primate-specific zinc finger proteins, in particular, *ZNF534* and *ZNF91* genes, would relieve the repressive chromatin from SCARS loci and facilitate activation of SCARS expression (**Figure 1B**). Consistently, during transition from the oocyte to the morula stage of human preimplantation embryogenesis, promoters of genes comprising the MLME GES shift from nearly exclusively homogenously closed (silenced) states to predominantly homogenously open (active) states (**Figure 2**). The predominantly homogenously open promoter states of genes comprising the MLME signature are maintained in human embryonic cells of the ICM, TE, and hESC (**Figure 2**). Thus, activation of SCARS expression is clearly the secondary event driven by global demethylation during zygote-to-embryo transition and fine-tuned DNA methylation reprogramming. In this context, transcriptional activation of SCARS should be regarded as the consequence of changes of epigenetic regulatory mechanisms designed to silence SCARS expression.

### SCARS Represent Both Intrinsic and Integral Components of Human-Specific Genomic Regulatory Networks

SCARS-encoding loci are predominantly primate-specific regulatory sequences because they are common for Modern Humans and non-human primates (56). However, sizable fractions of different SCARS families are represented by human-specific (unique-to-human) regulatory sequences. For example, 302 of 1,222 (24.7%) full-length LTR7/HERV-H elements have been identified as candidate human-specific regulatory sequences, HSRS (56). Species-specificity of SCARS is defined by the unique genomic coordinates of the insertions of corresponding parent transposons, which appear as segments of DNA present on human chromosomes and absent on chromosomes of non-human primates. Interestingly, 37.6% of highly active in hESC LTR7/HERV-H elements have been classified as HSRS (56). This is contrast to only 19.8% LTR7/HERV-H that are inactive in hESC being identified as candidate HSRS (p <0.0001). Therefore, globally SCARS should be viewed within the genomic regulatory context of other classes of HSRS (29).

**Figure 3:**
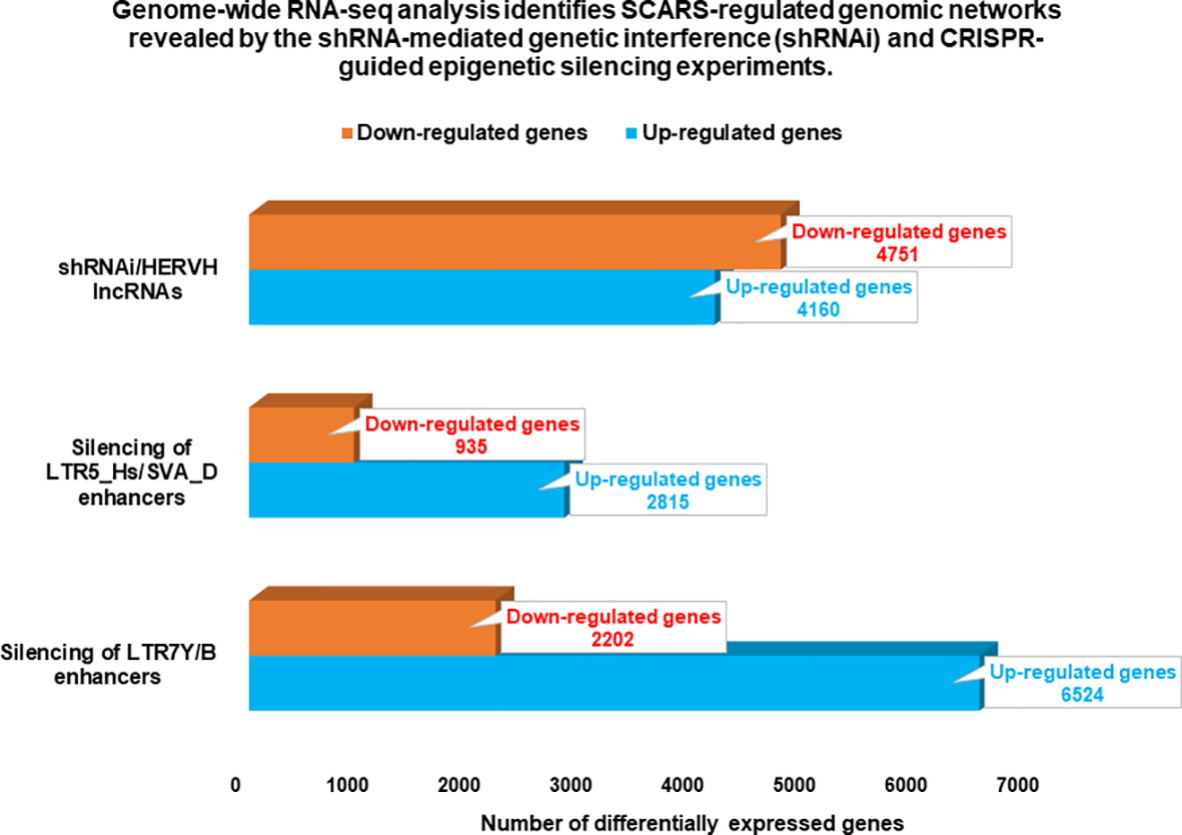
Genome-wide gene expression profiling experiments identify thousands of SCARS-regulated genes in hESC. Genome-wide RNAseq analyses were performed on genetically engineered hESC to identify genes regulated by SCARS-encoded regulatory signals derived from HERV-H, LTR5_Hs/SVA_D, and LTR7Y/B loci. Genes regulated by HERV-H ncRNA molecules were identified using shRNA-mediated genetic interference (100), while genes regulated by LTR5_Hs/SVA_D and LTR7Y/B enhancers were identified employing CRISPR/Cas-guided epigenetic silencing (22).

Candidate HSRS comprise a coherent compendium of nearly one hundred thousand genomic regulatory elements, including 59,732 HSRS which are markedly distinct in their structure, function, and evolutionary origin (29) as well as 35,074 human-specific neuro-regulatory single nucleotide changes (hsSNCs) located in differentially-accessible (DA) chromatin regions during human brain development (30, 131). Unified activities of HSRS may have contributed to development and manifestation of thousands human-specific phenotypic traits [30]. SCARS encoded by human endogenous retroviruses LTR7/HERV-H and LTR5_Hs/HERV-K have been identified as one of the significant sources of the evolutionary origin of HSRS (6, 18, 25–30, 46, 52, 56, 83, 90, 127), including human-specific transcription factor binding sites (TFBS) for NANOG, OCT4, and CTCF (25, 28). It was interest to determine whether genes previously linked to multiple classes of HSRS, which were identified without considerations of genes expression of which is regulated by SCARS, overlap with SCARS-regulated genes. To this end, 13,824 genes associated with different classes of HSRS were identified using the GREAT algorithm (29, 30), subjected to the GSEA, and compared with the sets of SCARS-regulated genes (**Figure 3**) identified by shRNA interference (100) and CRISR/Cas-guided epigenetic silencing experiments comparing regulatory networks of naïve and primed hESC (22, 130). These analyses revealed that SCARS appear to affect expression of a majority (8,384 genes; 61%) of genes associated with different classes of HSRS (**Table 2**; **Supplemental Table S1**), in agreement with the hypothesis that a large fraction of SCARS-regulated genes represents an intrinsic component of human-specific genomic regulatory networks. Consistently, SCARS affect expression of a majority of genes (5,389 of 8,405 genes; 64%) associated with neuro-regulatory hsSNCs (30). Overall, the common gene set of regulatory targets independently defined for HSRS, SCARS, and neuro-regulatory hsSNCs comprises of 7,990 coding genes or 95% of all genes associated with neuro-regulatory hsSNCs located in DA chromatin regions during human brain development (30).

**Table 2:** SCARS regulate expression of a majority of 13,824 genes associated with human-specific regulatory sequences (HSRS).

Classification category	Number of genes	Percent*
**HERV-H lncRNA-regulated genes**	4,805	34.76
**LTR7Y/B enhancers-regulated genes**	5,240	37.91
**LTR5_Hs/SVA_D enhancers-regulated genes**	2,022	14.63
**All SCARS-regulated HSRS-associated genes**	8,384	60.65

*percent of all HSRS-associated genes.

Genes associated with HSRS and neuro-regulatory hsSNCs manifest a staggering breadth of significant associations with morphological structures, physiological processes, and pathological conditions of Modern Humans (30), indicating that a preponderance of human-specific traits evolved under a combinatorial regulatory control of HSRS and neuro-regulatory loci harboring hsSNCs. SCARS-regulated genes comprise a large fraction of these human-specific genomic regulatory networks and represent an integral component of genomic regulatory wiring governing human-specific features of early embryonic development.

One of the important questions is whether the patterns of significant associations with physiological and pathological phenotypes observed for genes linked with HSRS, hsSNCs, and SCARS are specific and not related to the size effects of relatively large gene sets subjected to the GSEA (30). To address this questions, 42,847 human genes not linked by the GREAT algorithm with HSRS were randomly split into 21 control gene sets of various sizes ranging from 2,847 to 6,847 genes and subjected to the GSEA [30]. Importantly, no significant phenotypic associations were observed for 21 control gene sets, indicating that phenotypic associations attributed to genes linked with HSRS, hsSNCs, and SCARS are not likely due to non-specific gene set size effects captured by the GSEA. These observations are highly consistent with the conclusion that a broad spectrum of significant phenotypic associations documented for genes linked with HSRS, neuro-regulatory hsSNCs, and SCARS reflects their bona fide impacts on physiological and pathological phenotypes of Modern Humans. It should be underscored that the efficient execution of these analytical experiments was greatly facilitated by the web-based utilities provided by the Enrichr Bioinformatics System Biology platform (132, 133).

### Gene Set Enrichment Analyses (GSEA) of 8,384 Genes Associated With HSRS, Expression of Which Is Regulated by LTR7Y/B and LTR5_Hs/SVA_D Enhancers and HERVH lncRNAs

GSEA on multiple genomics databases revealed remarkable breadth and depth of significant associations with physiological and pathological phenotypes of Modern Humans of 8,834 SCARS-regulated genes linked with multiple families of HSRS (**Supplemental Text S1**). Consistent with the established role of SCARS in human embryogenesis, SCARS-regulated genes are significantly enriched in human embryo and neuronal epithelium according to GSEA of the ARCHS4 Human Tissues database. Consistently, POU5F1 and PRDM14 master stem cell regulators were identified by GSEA of the ESCAPE stem cell-focused database as top up-stream regulators, while pathways in Cancer (KEGG 2019 Human database) and Axon Guidance (KEGG 2019 Mouse database) were scored as top significantly-enriched pathways.

GSEA of the Allan Brain Atlas database focused on up-regulated genes identified 590 human brain regions among significantly enriched records, while GSEA of the Allen Brain Atlas of down-regulated genes identified 847 significant records (adjusted p-value <0.05). Notably, seven of the top ten significantly enriched records among up-regulated genes identified the Dentate Gyrus, while remaining three of the top 10 records identified the Fields CA3 of stratum pyramidale and stratum lucidum of the hippocampus (**Supplemental Text S1**; Allan Brain Atlas database; up-regulated genes).

GSEA of the Virus MINT database comprising of human genes that encode proteins known to physically interact with viruses and viral proteins identified the Epstein–Barr virus as the top-scoring record, indicating that upon entry in human cells the Epstein–Barr virus-encoded proteins target proteins encoded by SCARS-regulated genes. Overall, expression of nearly 60% of all human genes encoding virus-interacting proteins (2,574 of 4,433 VIP-encoding genes; 58%) is regulated by SCARS.

### GSEA of 2,846 Genes Associated With Created *De Novo* HSRS, Expression of Which Is Regulated by LTR7Y/B and LTR5_Hs/SVA_D Enhancers and HERVH lncRNAs

In human genome, there are 4,528 genes comprising putative regulatory targets of ~12,000 created *de novo* HSRS (29, 30). Notably, SCARS regulate expression of 2,846 genes (63%) of all genes identified as candidate regulatory targets of created *de novo* HSRS. GSEA of genomics databases revealed numerous significant enrichment records linked with 2,846 SCARS-regulated genes, thus highlighting their potential impacts on human physiology and pathology (**Supplemental Text S2**).

Unexpectedly, GSEA of the ENCODE and ChEA Consensus transcription factors (TFs) from ChIP-X database identified androgen receptor (AR) as a top-scoring candidate upstream regulator. In agreement with the above observations, GSEA of the ARCHS4 Human Tissues database identified Neuronal epithelium, Human embryo, and Prefrontal cortex as top significantly-enriched records (**Supplemental Text S2**). Pathways in Cancer (KEGG 2019 Human database) and Axon Guidance (KEGG 2019 Mouse database) were identified as top significantly enriched pathways. Additionally, pathways of Integrins in angiogenesis (NCI-Nature 2016 database) and Integrin signaling (Panther 2016 database) were identified as top-scoring significantly-enriched pathways (**Supplemental Text S2**).

GSEA of the Jensen Tissues database identified 134 significantly enriched records indicating that SCARS-regulated genes associated with created *de novo* HSRS have been previously identified among genes comprising expression signatures of many human tissues. Other notable findings were revealed by the GSEA of the Human Phenotype Ontology database (81 significant records); the MGI Mammalian Phenotype 2017 database (309 significant records); the Allen Brain Atlas databases of up-regulated genes (284 significantly-enriched brain regions) and down-regulated genes (408 significantly-enriched brain regions).

Systematic GSEA of genomic databases revealed that SCARS-regulated genes appear significantly enriched among genes associated with a multitude of human common and rare diseases. For example, GSEA of the Rare Diseases AutoRIF ARCHS4 Predictions database captured 353 significantly-enriched records of human rare disorders (**Supplemental Text S2**). GSEA of the Disease Perturbations from Gene Expression Omnibus (GEO) database of up-regulated genes identified 246 significant records, while interrogation of the Disease Perturbations from GEO database of down-regulated genes revealed 203 significantly-enriched records (**Supplemental Text S2**). Lastly, according to GSEA of the Jensen Diseases database, a significant majority of SCARS-regulated genes associated with created *de novo* HSGRS (2,008 of 2846 genes; 71%) have been implicated in development and clinical manifestations of multiple types of human cancers (**Supplemental Text S2**). Collectively, these observations indicate that a majority of genes expression of which is regulated by SCARS have been implicated in pathogenesis of the exceptionally broad spectrum of human rare and common disorders, supporting the hypothesis of deregulation of SCARS-associated genomic regulatory networks as a common denominator of the pathogenesis of human diseases.

### Inference of Potential Impacts of SCARS on Development and Clinical Behavior of Human Malignancies

SCARS activation hypothesis postulates the central role of a sustained activity of SCARS in acquisition and maintenance of stemness features in human cancer cells, clinical manifestations of which are reflected in high likelihood of therapy failure and death from cancer (6, 18, 25–30, 46, 52, 56, 83, 90, 127). This intrinsic propensity to evade the malignancy eradication therapies is proposed to exist even if SCARS-activation driven cancer is diagnosed as the early stage disease based on established pathomorphological and molecular criteria.

Observations capturing the principal molecular, genetic, and biological features attributed to regulatory impacts of SCARS were made in experimental models of naïve and primed hESC, human induced pluripotent stem cells (iPSC), and human preimplantation embryogenesis. These experiments identified genes expression of which is significantly altered in human cells subjected to targeted genetic manipulations to achieve SCARS over-expression (18, 138, 139) and/or silencing using shRNA interference (100, 138, 139), CRISPR/Cas gene knockout technology (138) as well as CRISPR/Cas-guided epigenetic silencing of SCARS (22), thus facilitating identification of multiple gene expression signatures (GES) reflecting fine details of experimentally-defined SCARS-associated genomic regulatory networks.

### Impacts of Genes Comprising Distinct GES Regulated by LTR7Y/B and LTR5_Hs/SVA_D Enhancers and HERVH lncRNAs

Potential biological relevance of several experimentally-defined GES comprising distinct panels of SCARS-regulated genes have been evaluated using Gene Set Enrichment Analyses (GSEA) across multiple genomic databases as previously described (29, 30). These analytical experiments were executed using the web-based tools of the Enrichr Bioinformatics System Biology platform (132, 133). To date, the following GES of SCARS-regulated networks in hESC are available for follow-up interrogations of their biological impacts and potential translational significance:

1. GES comprising a set of 1,141 genes that are regulated by both HERVH lncRNA and LTR5_Hs/SVA_D enhancers;
2. GES comprising a set of 3,063 genes regulated by both LTR7Y/B enhancers and HERVH lncRNA;
3. GES comprising a set of 1,477 genes regulated by both LTR7Y/B enhancers and HERVH lncRNA and manifesting concordant expression profiles;
4. GES comprising a set of 1,586 genes regulated by both LTR7Y/B enhancers and HERVH lncRNA and manifesting discordant expression profiles;

The up to date summary of the key findings for each of these four SCARS GES is reported in **Supplemental Text S3**. Notably, GSEA of 1,141 genes that are regulated by both LTR5_Hs/SVA_D enhancers and HERV-H lncRNA facilitated identification and characterization of sub-sets of SCARS-regulated genes implicated in Parkinson’s disease, autism, multiple types of cancer, and human embryonic development (**Supplemental Text S3**).

GSEA of the Jensen Diseases database revealed that a significant majority of genes regulated by both HERV-H lncRNA and LTR7Y/B enhancers (1,905 of 3,063 genes; 62%) have been implicated in development and clinical manifestations of multiple types of human cancer. Similarly, a significant majority of genes regulated by both HERV-H lncRNA and LTR7Y/B enhancers and manifesting concordant expression profiles (972 of 1,477 genes; 66%) have been implicated in development and clinical manifestations of multiple types of malignancies (**Supplemental Text S3**).

### HSRS and SCARS Regulate Expression of a Majority of Cancer Survival Predictor Genes and Cancer Driver Genes

**Figure 4:**
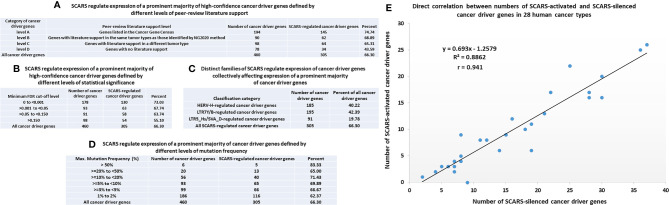
SCARS regulate expression of a prominent majority of cancer driver genes. A total of 460 cancer driver genes reported in (146) were evaluated for regulatory dependency from SCARS. **(A)** SCARS regulate expression of a prominent majority of high-confidence cancer driver genes defined by different levels of peer-review literature support. **(B)** SCARS regulate expression of a prominent majority of high-confidence cancer driver genes defined by different levels of statistical significance. **(C)** Distinct families of SCARS regulate expression of cancer driver genes collectively affecting expression of a prominent majority of cancer driver genes. **(D)** SCARS regulate expression of a prominent majority of cancer driver genes defined by different levels of mutation frequency. **(E)** Direct correlation between numbers of SCARS-activated and SCARS-silenced cancer driver genes in 28 human cancer types.

One of the approaches to evaluation of potential impacts of SCARS on development and clinical manifestations of human malignancies could be the assessment of regulatory effects of SCARS on cancer survival and cancer driver genes. To this end, analyses of 10,713 protein-coding genes expression changes of which are significantly associated with the increased likelihood of survival of cancer patients diagnosed with 17 major cancer types (145) and 460 cancer driver genes identified in 28 human cancer types (146) revealed that SCARS regulate a majority of both cancer survival predictor genes and cancer driver genes (**Tables 3**, **4**, **Figures 4A–E**; **Supplemental Text S4**). It has been observed (**Table 3**) that a prominent majority of human cancer survival predictor genes is regulated by HSRS (7,738 genes; 72%). As shown in **Table 4**, SCARS regulate expression of 7,609 of 10,713 (71%) human cancer survival predictor genes (**Table 4**).

**Table 3:** A prominent majority of human cancer survival predictor genes is associated with human-specific regulatory sequences (HSRS).

TYPE OF CANCER	CANCER SURVIVAL GENES	HSRS-ASSOCIATED	PERCENT
**Thyroid**	347	269	77.52
**Glioma**	271	206	76.01
**Melanoma**	205	153	74.63
**Head and neck**	808	597	73.89
**Colorectal**	603	440	72.97
**Renal**	6,070	4,418	72.78
**Ovarian**	504	366	72.62
**Liver**	2,892	2,086	72.13
**Lung**	662	477	72.05
**Breast**	582	414	71.13
**Urothelial**	1,101	783	71.12
**Stomach**	307	218	71.01
**Prostate**	161	114	70.81
**Endometrial**	1,631	1,153	70.69
**Cervical**	717	505	70.43
**Pancreatic**	1,549	1,075	69.40
**Testis**	60	42	70.00
**All human cancer survival genes**	10,713	7,738	72.23

Numbers of genes in each cell reflect the sum of records in the corresponding classification category when individual genes were recorded as a single count. Uhlen etal. (145) reported a total of 10,713 protein-coding genes expression changes of which are significantly associated with the increased likelihood of survival of cancer patients diagnosed with 17 major cancer types. Percent values were calculated as fractions of all cancer survival genes in the corresponding classification categories.

**Table 4:** SCARS regulate expression of a prominent majority of human cancer survival predictor genes.

TYPE OF CANCER	CANCER SURVIVAL GENES	SCARS-REGULATED	PERCENT	HERV-H-REGULATED	PERCENT	LTR7Y/B-REGULATED	PERCENT	LTR5_Hs/SVA_D-REGULATED	PERCENT
**BREAST**	582	405	69.59	229	39.35	284	48.80	80	13.75
**PROSTATE**	161	121	75.16	63	39.13	90	55.90	10	6.21
**PANCREATIC**	1,549	1,112	71.79	629	40.61	772	49.84	250	16.14
**LIVER**	2,892	2,217	76.66	1,267	43.81	1,565	54.11	382	13.21
**RENAL**	6,070	4,406	72.59	2579	42.49	2,881	47.46	965	15.90
**COLORECTAL**	603	448	74.30	262	43.45	320	53.07	104	17.25
**CERVICAL**	717	526	73.36	312	43.51	340	47.42	112	15.62
**LUNG**	662	488	73.72	298	45.02	312	47.13	105	15.86
**THYROID**	347	259	74.64	153	44.09	171	49.28	56	16.14
**OVARIAN**	504	368	73.02	202	40.08	233	46.23	78	15.48
**ENDOMETRIAL**	1,631	1,129	69.22	652	39.98	747	45.80	250	15.33
**UROTHELIAL**	1,101	772	70.12	458	41.60	483	43.87	164	14.90
**HEAD & NECK**	808	558	69.06	340	42.08	369	45.67	128	15.84
**GLIOMA**	271	204	75.28	115	42.44	128	47.23	48	17.71
**MEANOMA**	205	148	72.20	85	41.46	107	52.20	25	12.20
**STOMACH**	307	219	71.34	144	46.91	131	42.67	25	8.14
**TESTIS**	60	41	68.33	23	38.33	26	43.33	11	18.33
**ALL**	10,713	7,609	71.03	4436	41.41	5,013	46.79	1,641	15.32

Numbers of genes in each cell reflect the sum of records in the corresponding classification category when individual genes were recorded as a single count. Uhlen etal. (145) reported a total of 10,713 protein-coding genes expression changes of which are significantly associated with the increased likelihood of survival of cancer patients diagnosed with 17 major cancer types. Percent values were calculated as fractions of all cancer survival genes in the corresponding classification categories.

SCARS regulate expression of two-third cancer driver genes (305 of 460 genes; 66%) and as many as 73–75% of high-confidence cancer driver genes (**Figure 4**), which were defined by either the level of peer-reviewed literature support (**Figure 4A**) or the statistical significance levels (**Figure 4B**). Notably, SCARS regulate expression of a majority of cancer driver genes regardless of their maximum mutations’ frequency (**Figure 4D**). SCARS-regulated cancer driver genes were identified in all analyzed to date 28 types of human cancer (**Table 5**). From the therapeutic strategy stand point, it is important to map actionable cancer therapy-guiding nodes defined by the SCARS stemness matrix which is mapped to connect Cancer Driver Genes/Cancer Type/Regulatory SCARS (**Table 5**). Further details describing regulatory effects of HSRS and SCARS on cancer survival predictor and cancer driver genes are reported in **Supplemental Text S4**. Collectively, these findings indicate that SCARS regulate expression of a majority of cancer survival predictor genes and cancer driver genes, which is consistent with the hypothesis implicating deregulated SCARS-associated genomic regulatory networks in pathogenesis of multiple types of human malignancies.

**Table 5:** SCARS-guided cancer stemness matrix of diagnostic and therapeutic targets comprising of 237 SCARS-down-regulated and 141 SCARS-activated cancer driver genes mapped to 28 cancer types.

Cancer Type	Number of SCARS-silenced cancer driver genes	Number of SCARS-activated cancer driver genes
**Adenoid Cystic**	7	4
**Bladder**	30	16
**Blood**	25	22
**Brain**	28	16
**Breast**	28	17
**Cervix**	12	8
**Cholangiocarcinoma**	8	4
**Colorectal**	16	12
**Endometrium**	30	20
**Gastroesophageal**	37	26
**Head & Neck**	19	6
**Kidney Clear**	8	5
**Kidney Non-Clear**	14	6
**Liver**	18	10
**Lung AD**	15	9
**Lung SC**	7	3
**Lymph**	36	25
**Ovarian**	4	2
**Pancreas**	22	17
**Pheochromocytoma**	5	3
**Pleura**	9	0
**Prostate**	19	11
**Sarcoma**	6	3
**Skin**	21	13
**Testicular Germ Cell**	11	8
**Thymus**	7	2
**Thyroid**	8	9
**Uveal Melanoma**	2	1
**Number of actionable cancer therapy-guiding nodes defined by the SCARS stemness matrix mapped to connect Cancer Driver Genes/Cancer Type/Regulatory SCARS**	**1,365**	**834**

AD, adenocarcinoma; SC, small cell carcinoma. Bold values report total number of actionable cancer therapy-guiding nodes defined by the SCARS stemness matrix.

### Analysis of Potential Impacts of SCARS-Associated Malignancies on Clinical Intractability of Different Types of Human Cancers

**Figure 5:**
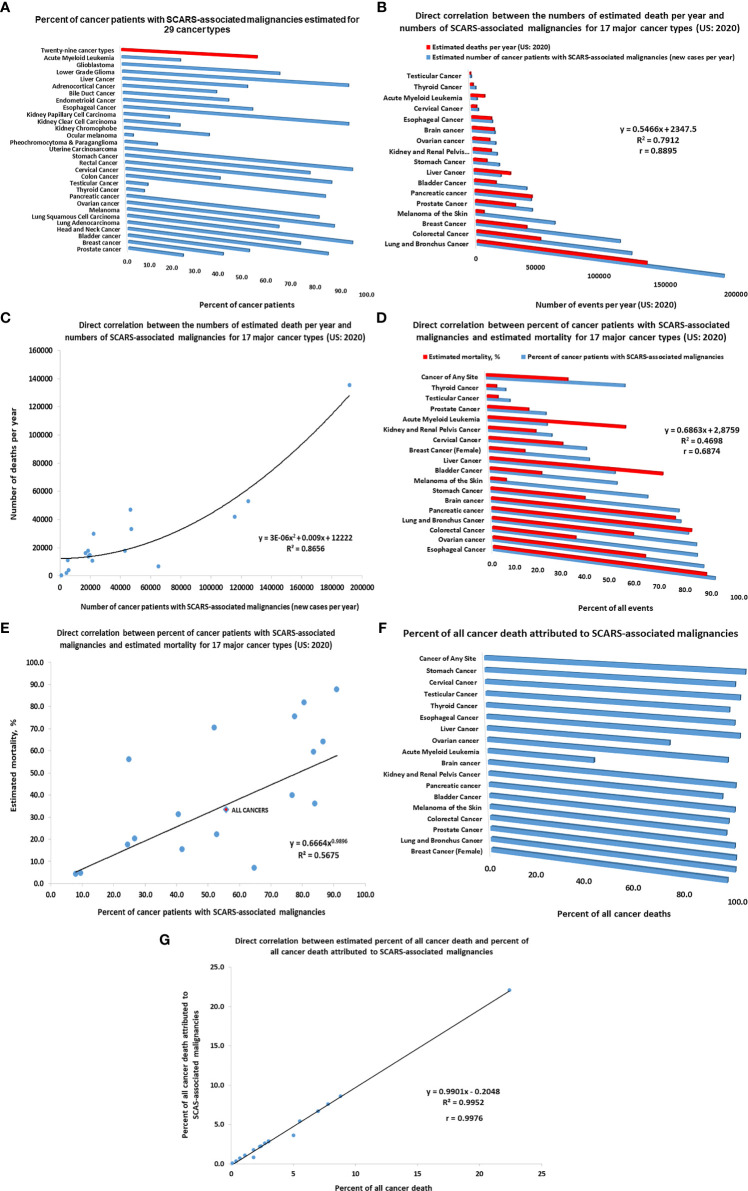
Inference of potential global impacts of SCARS-regulated genes on distinct mortality of different types of human malignancies. **(A)** Percent of cancer patients with SCARS-associated malignancies estimated for 29 cancer types (adopted from Refs (52, 56, 90). **(B)** Direct correlation between the numbers of estimated death per year and numbers of SCARS-associated malignancies for 17 major cancer types (US: 2020). **(C)** Correlation plot illustrating a direct correlation between the numbers of estimated death per year and numbers of SCARS-associated malignancies for 17 major cancer types (US: 2020). **(D)** Direct correlation between percent of cancer patients with SCARS-associated malignancies and estimated mortality rates for 17 major cancer types (US: 2020). **(E)** Correlation plot illustrating a direct correlation between percent of cancer patients with SCARS-associated malignancies and estimated mortality rates for 17 major cancer types (US: 2020). **(F)** Percent of all cancer death attributed to SCARS-associated malignancies estimated for 17 major cancer types. Estimates of maximum values are reported which were calculated not to exceed the total number of estimated death for each cancer type. **(G)** Correlation plot illustrating a direct correlation between estimated percent of all cancer death and percent of all cancer death attributed to SCARS-associated malignancies for 17 major cancer types.

Previous work (52, 56, 90) has identified the proportions of SCARS-associated malignancies among 29 different types of human cancers using The Cancer Genome Atlas (TCGA) database and somatic non-silent mutations (SNMs) signatures of SCARS-regulated genes. Using this approach, it has been observed that patients with malignancies harboring the SNM signatures had significantly higher likelihood of dying from cancer compared with patients whose tumors have no SNMs in SCARS-regulated genes (46, 52, 56, 90). Plotting these data as a set of bar graphs clearly demonstrate that different types of human cancers have markedly different proportions of cancer patients diagnosed with tumors containing SCARS-regulated genes with SNMs (**Figure 5A**). It was of interest to assess potential global impacts of SCARS-regulated genes on distinct mortality documented for different types of human malignancies.

Using the estimates of prevalence of cancer patients with SCARS-associated malignancies among different cancer types (46, 52, 56, 90) as well as estimated numbers of new cases and deaths in the United States reported for 17 major cancer types for 2020 (American Cancer Society, 2020; https://www.cancer.org/cancer/all-cancer-types.html), the numbers of newly diagnosed cases of cancers and deaths attributed to SCARS-associated malignancies have been calculated and analyzed. Results of these analyses reported in **Figure 5** indicate that differences between the relative prevalence of SCARS-associated malignancies among different cancer types appears directly correlated with estimated mortality (**Figure 5**). This conclusion is supported by the findings of direct correlation between the numbers of estimated death per year and numbers of SCARS-associated malignancies for 17 major cancer types (US: 2020; **Figures 5B, C**) as well as direct correlation between percent of cancer patients with SCARS-associated malignancies and estimated mortality rates for 17 major cancer types (US: 2020; **Figures 5D, E**). Further analyses revealed a direct correlation between estimated percent of all cancer death and percent of all cancer death attributed to SCARS-associated malignancies for 17 major cancer types (**Figures 5F, G**). Collectively, these findings support the idea that differences in the prevalence of SCARS-associated malignancies among different cancer types diagnosed in different organs may represent a significant (perhaps, major) determinant of markedly distinct mortality documented for different types of human cancers arising in different organs of the human body.

### SCARS Exert Global Impacts on Development and Pathophysiology of Modern Humans

Global impacts of SCARS development of pathological conditions are defined by the broad spectrum of their molecular functions and are not limited to pathogenesis of human cancers. One of the most significant molecular functions of SCARS is highlighted by their role as functionally active enhancers as well as the ability of SCARS to alter enhancers’ activity. DNA sequences defined as candidate enhancer elements could be divided into functionally silent and functionally active categories. Exceedingly large set of functionally silent enhancers could be defined by the presence of characteristic chromatin marks indicating that specific DNA sequences harboring these chromatin marks may function as enhancer elements. Accurate molecular and genetic definitions of functionally active enhancers require the application of specific assays in a particular cell type as it has been reported for hESC (147). It has been observed that SCARS are significantly enriched among regulatory DNA sequences identified in either primed or naïve hESC as functionally active enhancer elements (28, 147). Furthermore, human embryonic MLME cells, creation of which was associated with SCARS activity (18), appear to capture GES of both Naïve and Primed hESC (**Supplemental Text S5**) with more significant resemblance of hESC in the Naïve state. Notably, patterns of TE-derived regulatory loci differentially expressed in MLME cells versus embryo and Naïve versus Primed hESC appear highly similar (**Supplemental Figure S2**). Therefore, assessments of biological roles of functionally active enhancers in hESC may shed a light on our understanding of potential biological impacts of SCARS-associated genomic regulatory networks.

Arguably, two key biologically-distinct functions of active enhancers in hESC are the maintenance of self-renewal and pluripotency states by restricting the differentiation potential and changing on demand the expression of genes linked to major embryonic lineages. Primed hESCs, in particular, are thought to represent a state poised to differentiation in which functionally active enhancers linked to differentiation of various lineages can be quickly switched on or off in response to developmental cues (likely in response to changes in chromatin and histone modification patterns). The biological role of functionally active hESC enhancers could be inferred by evaluating the enrichment within regulatory networks governed by naïve and primed hESC enhancers of genes comprising expression signatures of different human and non-human embryonic lineages (**Table 6**). In these analyses gene expression signatures of major embryonic lineages of distinct species, including humans, monkeys, and mice were evaluated (18, 98, 100, 141, 142, 148, 149). To this end, all genes comprising expression signatures of distinct embryonic lineages were assessed and genes which are located in close genomic proximity (at a distance of 10 kb or less) to naïve and primed hESC functionally active enhancers were identified. It has been observed that in all instances a high proportion of marker genes distinguishing embryonic lineages are located in close genomic proximity to hESC functional enhancers (**Table 6**). Notably, proportions of genes associated with naïve and primed hESC enhancers appear similar, consistent with the hypothesis that both naive and primed hESC represent functionally distinct states with the complimentary relevance to mechanistic exploration of developmental pathways.

**Table 6:** Enrichment within regulatory networks of Naïve and Primed hESC active enhancers of gene expression signatures (GES) defining embryonic lineages of distinct species.

Human epiblast (EPI) vs naïve hESC (hESCp#0)				
Classification category	Number of genes	Percent	P value*	Observed/Expected
Human EPI vs hESCp#0 GES	1496	100.0		
Naïve functional enhancers network	762	50.9	1.544E-69	1.73
Primed functional enhancers network	726	48.5	5.669E-73	1.80
Naïve & Primed functional enhancers networks	976	65.2	4.326E-89	1.63
**Human epiblast (EPI) vs Trophectoderm (TE) GES**				
Classification category	Number of genes	Percent	P value*	Observed/Expected
Human EPI vs TE expression signature	836	100.0		
Naïve functional enhancers network	525	62.8	1.176E-89	2.13
Primed functional enhancers network	472	56.5	2.095E-73	2.10
Naïve & Primed functional enhancers networks	647	77.4	2.11E-109	1.94
**Monkey epiblast (EPI) GES**				
Classification category	Number of genes	Percent	P value*	Observed/Expected
Monkey EPI expression signature	719	100.0		
Naïve functional enhancers network	442	61.5	1.665E-71	2.09
Primed functional enhancers network	399	55.5	1.687E-59	2.06
Naïve & Primed functional enhancers networks	529	73.6	1.003E-75	1.84
**Mouse inner cell mass (ICM) vs Trophectoderm (TE)**				
Classification category	Number of genes	Percent	P value*	Observed/Expected
Mouse ICM vs TE expression signature	497	100.0		
Naïve functional enhancers network	246	49.5	2.533E-21	1.68
Primed functional enhancers network	211	42.5	2.314E-14	1.58
Naïve & Primed functional enhancers networks	303	61.0	1.061E-21	1.53

*p values were estimated using the hypergeometric distribution test; expected values were estimated based on the number of genes in the human genome (63,677); number of genes associated with functional enhancers of the Naïve hESC (18,766); number of genes associated with functional enhancers of the Primed hESC (17,131); and number of genes associated with functional enhancers of both Naive and Primed hESC (25,421); GES, gene expression signature.

To assess the statistical significance of these findings, observed numbers of genes associated with hESC functional enhancers were compared to the expected values based on associations by chance alone. Expected values were estimated based on the number of genes in the human genome (63,677); number of genes associated with functional enhancers of the Naïve hESC (18,766); number of genes associated with functional enhancers of the Primed hESC (17,131); number of genes associated with functional enhancers of both Naive and Primed hESC (25,421); and numbers of genes in the corresponding expression signatures of embryonic lineages. These analyses revealed that in all instances differences between the observed and expected numbers of observations appear highly statistically significant (**Table 6**). These findings indicate that genomic networks governed by both naïve and primed functional enhancers in hESC may represent valuable models for follow-up mechanistic studies of regulatory mechanisms governing critical stages of the human pre-implantation embryogenesis.

**Figure 6:**
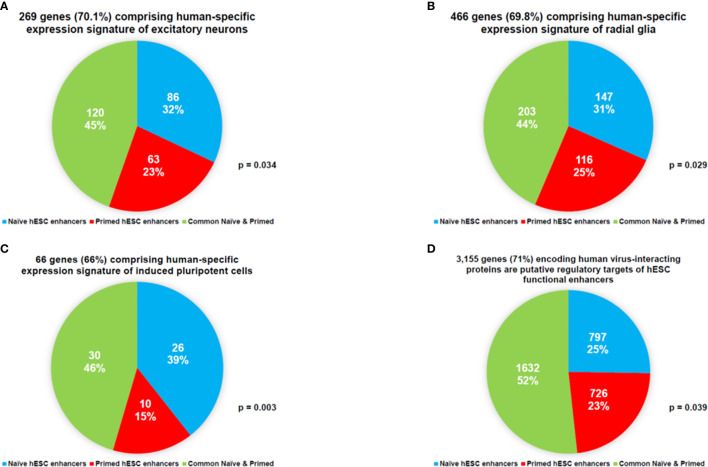
Networks of genes regulated in Naïve and Primed hESC hESC by functionally-active enhancers are enriched for genes comprising human-specific expression signatures of excitatory neurons **(A)**, radial glia **(B)**, induced pluripotent cells **(C)**, and human genes encoding a majority of virus-interacting proteins **(D)**.

This line of investigations have been extended to evaluate the potential biological role of hESC functionally active enhancers by performing the proximity placement analyses of genes associated with regulatory networks of naïve and primed hESC functional enhancers and compare these with genes involved in human embryonic, neurodevelopmental, and cancer survival predictors’ transcriptional networks, including human-specific GRNs (**Supplemental Tables S9**, **S10**), which were previously identified in multiple independent studies (18, 22, 27–30, 83, 98, 100, 130, 131, 140–142, 145, 148–155). A comprehensive genome-wide proximity placement analyses identifies all genes associated with functional enhancers, which were defined based on the location of their genomic coordinates within ±10 Kb windows of the corresponding enhancer’s genomic coordinates (28, 147). All genes in common have been identified for a set of genes associated with enhancers and a set of genes comprising the expression signatures of corresponding embryonic, neurodevelopmental, and cancer survival predictors’ networks. Finally, the assessment of statistical significance of observed versus expected numbers of genes in common has been performed for corresponding gene sets. Highly significant associations (**Supplemental Tables S9**, **S10**) of genes defining human embryonic, neurodevelopmental, and cancer survival predictors’ transcriptional networks with naïve (**Supplemental Table S9**) and primed (**Supplemental Table S10**) hESC functionally active enhancers have been observed. Genes associated with functionally active enhancers in Naïve and Primed hESC are significantly enriched for genes comprising human-specific expression signatures of excitatory neurons (**Figure 6A**), radial glia (**Figure 6B**), induced pluripotent cells (**Figure 6C**), and human genes encoding a majority of virus-interacting proteins (**Figure 6D**). It should be noted that these regulatory genomic features of functionally active hESC enhancers are markedly similar to the regulatory impacts of HSRS and SCARS on genes implicated in pathogenesis of neurodevelopmental, neuropsychiatric, and neurodegenerative disorders (27–30). The summary of observations supporting this conclusion is reported in **Supplemental Text S6**.

Collectively, these findings strongly argue that a comprehensive catalog of functionally active enhancers in hESC together with GES of SCARS-regulated genes may serve as an important previously unavailable resource for evidence-based mechanistic dissections of fine genomic regulatory architectures governing expression of genes implicated in transcriptional networks relevant to human development and diseases. Of particular interest would be experimental assessments of biological impacts of proteins bound to SCARS, in particular, HPAT-binding proteins many of which have been previously identified as virus-interacting proteins and shown to manifest a prominent expression in the human brain (**Supplemental Figure S3**).

## Discussion

### Evolutionary Aspects of the Emergence of Overlapping Genetic Networks Associated With Cancer and Other Common Human Disorders

**Figure 7:**
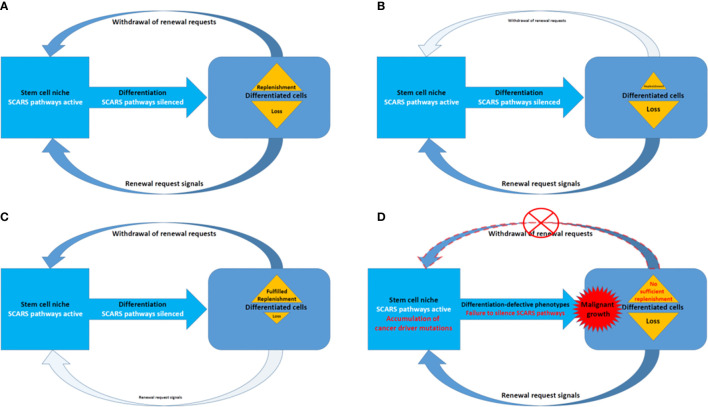
A model of SCARS expression dependence of oscillation patterns of loss and replenishment cycles of dying differentiated cells. **(A)** A model of the loss/replenishment cycle in the balanced state. **(B)** A model of the cycle at the prevalent loss of differentiated cells state. **(C)** A model of the cycle at the completed replenishment of differentiated cells state. **(D)** A model of the cycle with failed replenishment of differentiated cells due to failure of the SCARS silencing during attempts toward differentiation.

Present analyses support the idea of shared genomic regulatory networks impacting pathogenesis of human cancers, neuropsychiatric, neurodevelopmental, and neurodegenerative disorders. Many genes that expressed in the human brain and specific cells in human preimplantation embryos tend to be long because they have more introns. It has been noted that there is a large overlapping genetic networks operating in MLME cells of human embryos and fetal/adult neocortex of human brains (18, 27). Overall, we have more introns in our genes than, for example mouse, and about 10% less protein coding genes. Thus, in genomes of Modern Humans high transcripts’ diversity (which impacts both regulatory diversity of RNA molecules and diversity of peptides and proteins) was achieved by inserting more intronic sequences and increasingly relying on splicing. Retrotransposition is one of the major mechanistic contributors to these continuing processes with major impacts on stem cells survival and expansion to sustain the regeneration and replenishment of dying differentiated cells in various tissues and organs (**Figure 7**).

DNA of intronic sequences-reach long genes that are expressed and continuously transcribed in these long living cells for many years of the individuals’ lifetime have a significantly higher probability to acquire and accumulate functionally deleterious, regulatory, and disease causing mutations. Depending on when and where it happened, it would manifest as different diseases: for example, in cells of coherent peripheral tissues it would be diagnosed as malignant tumors, while in cells of central nervous system it would be diagnosed as neurodevelopmental, neuropsychiatric, or neurodegenerative disorders. It has been suggested (25, 26) that, in addition to deamination of methyl-cytosine causing C/T mutations, one of the main mechanisms promoting the increased likelihood of mutations at defined genomic loci is the RNA-mediated formation of energetically-stable DNA : RNA triple-stranded complexes designated R-loops. Specifically, this model anticipates a particularly important role for R-loops formation of which is driven by SCARS-encoded RNA molecules to maintain regulatory DNA readily accessible to sequence-specific transcription factors, thus, ensuring the transcriptionally-competent chromatin state of defined genomic loci.

### SCARS Expression Dependence of Homeostatic Oscillation Patterns of Loss and Replenishment Cycles of Differentiated Cells

Homeostasis maintenance requires balanced and coordinated physiological functions of multiple organs and tissues in the human body, which relies on a timely replenishment of dying differentiated cells to compensate diminishing physiological functions and restore homeostasis (**Figure 7**). In a balanced state, the loss of differentiated cells is continually replenished during the regeneration process afforded by differentiation of stem cells (**Figure 7A**). The homeostatic balance of these oscillation patterns of loss (**Figure 7B**) and replenishment (**Figure 7C**) cycles of differentiated cells became disrupted when the silencing of SCARS expression failed in stem cells primed toward differentiation (**Figure 7D**). Failure to silence SCARS expression in stem cells induced toward differentiation results in breakdown of differentiation programs and accumulation of cells with differentiation-defective phenotype. According to this model, the persistent lack of sufficient replenishment of dying differentiated cells and resulting collapse of the replenishment cycle would signify the emergence of malignant growth (**Figure 7D**). Consequently, an apparent efficient approach to restore the homeostasis of loss and replenishment cycles of dying differentiated cells would be the silencing of SCARS expression.

### Emerging Role of Extracellular Vesicles in Accumulation, Transport, and Distal Reprogramming Effects of Retroviral Sequences

Human cells constitutively produce lipid-encapsulated extracellular vesicles (EVs) of different sizes classified as apoptotic bodies (500–2,000 nm), microvesicles (50–1,000 n), and exosomes (30–100 nm). Different types of EVs are distinguished by their biogenesis and contents of biologically active cargo of proteins, lipids, microRNAs, messenger RNAs, and long non-coding RNAs (156, 157). Cell-to-cell communications *via* release and reception of EVs have been recognized as one of the important mechanisms of intercellular exchange of biological information which do not require direct cell to cell contacts (158, 159).

Aberrant overexpression of TEs (see *Introduction*) and satellite repeats (160) have been documented in multiple types of human cancers. TE-encoded RNA molecules, including human endogenous retroviruses (HERV)-encoded sequences, appear preferentially accumulated in EVs isolated from blood of cancer patients (161). Interestingly, cancer-associated EVs seem capable of transmitting the TE-encoded biological information to various types of target cells, including stromal cells and immune cells. These findings are consistent with the hypothesis of a novel biological pathway of intercellular transmission and dissemination of TE-encoded genetic information explaining how aberrant expression of specific HERV-encoded RNAs may contribute to the pathogenesis of clinically lethal malignancies.

In agreement with this concept, the apparent association with metastatic disease of increased abundance of TE-encoded RNA molecules in EVs isolated from cancer patients’ blood has been observed (161). Notably, both HERVH- and HERVK-encoded transcripts were detected in cancer-associated EVs, including LTR7/HERVH—and LTR5_Hs/HERVK—derived transcripts. LTR7/HERVH- and LTR5_Hs/HERVK loci were previously identified as stem cell-associated retroviral sequences (SCARS), aberrant expression of which in malignant cells confers stemness phenotype and has been associated with the increased likelihood of therapy failure and death from cancer in multiple types of malignant tumors (46, 52, 56, 90). Detection of SCARS-encoded RNA molecules in cancer-associated EVs is particularly important in the context of the observed interference with cellular differentiation induced by the exposure of differentiating cells to cancer-associated EVs (161).

The remarkable diversity of RNA molecules encoded by a multitude of different HERV-derived sequences and packaged in the cancer-associated EVs has been documented (161). However, the reported analyses of the relative abundance of TE-encoded transcript packaged in cancer-associated EVs were limited to the RNAs with extended ORFs. This approach may represent a significant limitation, because many of the TE-encoded RNAs, including HERV-encoded RNA molecules, are most likely represented by small RNAs and other non-coding RNAs with known (or putative) regulatory functions.

**Figure 8:**
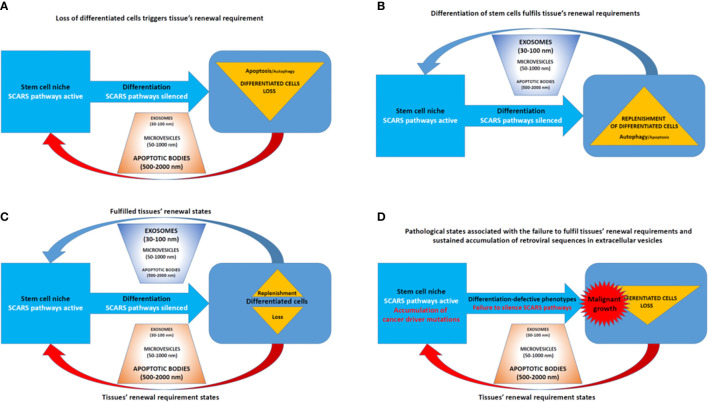
Extracellular vesicles (EVs)—Guided regulation of tissue homeostasis cycles of the loss and replenishment of differentiated cells. **(A)** A model of tissue homeostasis at the loss of differentiated cells stage. **(B)** A model of tissue homeostasis at the stage of completed replenishment of the loss of differentiated cells. **(C)** Continuing maintenance of tissue homeostasis cycles is associated with fluctuations of distinct types of EVs. **(D)** A model of pathological states associated with altered tissue homeostasis due to the failure of differentiated cells replenishment.

Collectively, these findings indicate that EVs and exosomes may play an important role in accumulation, transport, and distal reprogramming effects of RNA molecules encoded by SCARS and other retroviral sequences (**Figure 8**). Consistent with this model, SCARS-regulated genes represent a majority (74 of 115 genes; 64%) of genes expression of which is significantly up-regulated (p <0.01) in target cells exposed *in vitro* to cancer-associated EVs (unpublished observations). These considerations in conjunction with the oscillation model of loss and replenishment cycles of differentiated cells (**Figures 7**, **8**) provide experimentally testable hypotheses of molecular mechanisms of intercellular SCARS-mediated communications contributing to a systemic dissemination of cancer and other disease states.

### Hypothesis of an Essential Singular Source Code Driving the Faithful Execution of Early Embryogenesis Programs and Contributing to the Emergence of Disease States in Human Cells

**Figure 9:**
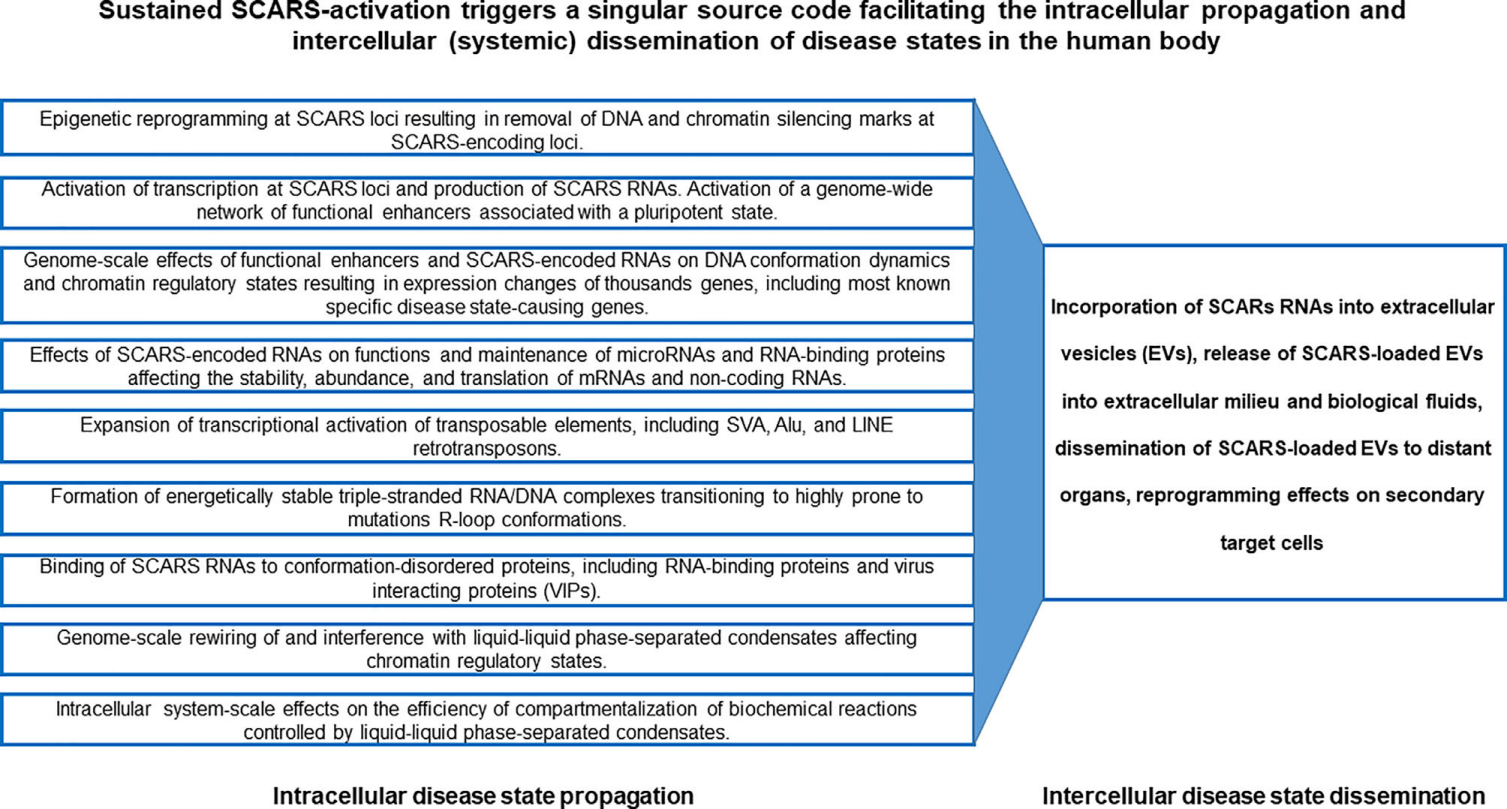
SCARS-activation triggered singular source code facilitating the intracellular propagation and intercellular (systemic) dissemination of disease states in the human body.

Precisely controlled waves of activities of distinct families of TEs, including SCARS, provide a genomic source code for proper execution of high-complexity developmental programs during human preimplantation embryogenesis. In human embryonic stem cells (hESC), sustained activities of SCARS is required for maintenance of the stemness state. Conversely, failure to silence SCARS during neuronal differentiation of hESC is associated with development of differentiation-defective phenotypes, indicating that SCARS activity is not compatible with physiological functions of differentiated human cells. Consequently, aberrant sustained activation of SCARS in long-living human cells might represent a genomic source code driving the emergence, propagation, and dissemination of various disease states, including cancer, neurodegeneration, neurodevelopmental and neuropsychiatric disorders (**Figure 9**). According to this model, the initial triggering event represents the epigenetic reprograming of the silent chromatin state leading to activation of genetic loci encoding SCARS. Subsequent continuing expression of RNA molecules harboring SCARS and SCARS-encoded peptides facilitates a cascading stream of molecular aberrations defining both the propagation of an intracellular pathological state and intercellular (systemic) dissemination of a disease state (**Figure 9**). In the context of neurodegenerative disorders, the toxicity of HERV-encoded RNAs and proteins may play an important role (162). It is hypothesized that underlying mechanisms enabling the intercellular (systemic) dissemination of a disease state are mediated by EVs loaded with SCARS-encoded RNAs and peptides, which exert the reprogramming effects on secondary (distant) target cells.

Therefore, one of the important end points of present analyses is the assembly of experimental evidence and theoretical considerations supporting the model of a singular genomic source code, activation and execution of which contributes to development of multiple types of human disorders. This model of a singular genomic source code captures the mechanistic complexity of multilevel intracellular effects of SCARS activation-driven malignant regulatory signatures and their potential global reprogramming impacts facilitating emergence, propagation, and dissemination of disease states in primary and secondary (distal) target cells (**Figure 9**).

## Conclusions and Future Prospects

One of the most promising avenues of research efforts toward understanding of genomic and molecular underpinning of malignant regulatory signatures has its origin in the fundamental advances revealing principal regulatory elements of genomic and molecular pathways of the stemness phenotype creation and maintenance during human embryonic development. Remarkable achievements of single-cell genomics of human preimplantation embryogenesis facilitated the emergence of the concept of SCARS as both intrinsic and integral components of human-specific genomic regulatory networks (GRNs), the main biological function of which is to enable the creation and maintenance of stemness features in human embryonic cells.

Several independent yet complementary approaches were utilized to discern the potential impacts of SCARS, other families of HSRS, and functionally-active hESC enhancers on physiological and pathological phenotypes of Modern Humans.

First, comprehensive lists of genes comprising down-stream targets of corresponding regulatory loci of interest have been identified.

Second, multiple gene expression signatures (GES) linked to regulatory loci of interest were deconvoluted from large sets of down-stream target genes.

Third, GSEA using an extensive collection of genomic databases have been carried out to statistically link down-stream target genes with phenotypic traits, morphological features, and physiological and pathological conditions.

Fourth, disease type-specific sets of genes were identified and assembled into panels of GES for follow-up interrogations of their potential pathophysiological impact and translational utilities.

Fifth, multiple human-specific genomic regulatory networks (GRNs) have been identified operating in developmentally and physiologically distinct human tissues and cells to dissect associations of down-stream target genes with defined human-specific GRNs.

The task of identification of down-stream-target genes was achieved using either overexpression of regulatory loci or genetic interference approaches, including shRNA-mediated interference and CRISPR/Cas9-guided epigenetic silencing (6, 18, 22, 25–30, 52, 56, 90, 100, 130, 138, 139, 147). Alternatively, proximity placement analyses of regulatory elements and down-stream targets were performed employing the GREAT algorithm (29, 30, 134, 135, 147).

Examples of the interrogated human-specific GRNs include the following data sets:

- Great Apes’ whole-genome sequencing-guided human-specific insertions and deletions (152);
- Genome-wide analysis of retrotransposon’s transcriptome in postmortem samples of human dorsolateral prefrontal cortex (83);
- shRNA-mediated silencing of LTR7/HERVH retrovirus-derived long non-coding RNAs in hESC (100);
- Single-cell expression profiling analyses of human preimplantation embryos (18, 140);
- Network of genes associated with regulatory transposable elements (TE) operating in naïve and primed hESC (22, 130);
- Pluripotency-related network of genes manifesting concordant expression changes in human fetal brain and adult neocortex (27);
- Network of genes governing human neurogenesis *in vivo* (153);
- Network of genes differentially expressed during human corticogenesis *in vitro* (154);
- Human-specific gene expression signatures of the adult brain (155);
- Single-cell analyses defined genomic signatures of the adult human brain (150, 151).

Thus, selected for these analyses human-specific GRNs appear to function in a developmentally and physiologically diverse spectrum of human cells that are biologically and anatomically highly relevant to manifestations of human-specific phenotypes ranging from preimplantation embryos to adult dorsolateral prefrontal cortex (6, 18, 22, 25–30, 52, 83, 131, 140, 148, 151–155).

In accord with the expected *in vivo* regulatory role of SCARS and hESC functional enhancers during human embryonic development, a significant enrichment of genes comprising expression signatures of major embryonic lineages of distinct species, including humans, monkeys, and mice has been observed within regulatory networks of Naïve and Primed hESC functional enhancers. Results of these analyses further support the hypothesis that key regulatory features of human neurodevelopmental networks are engaged during the early-stages of human embryogenesis (6, 18, 25–30, 52, 83, **Supplemental Tables S11–S12**]. Analyses of regulatory networks of Naïve and Primed hESC functional enhancers revealed a highly consistent pattern of significant enrichment of genes that were previously identified as principal components of major neurodevelopmental networks (**Supplemental Tables S9–S12**), including GES of human neuronal and non-neuronal brain cells (150), human neurons’ sub-types and neuronal diversity signatures (151), and human fetal brain/adult neocortex GES (27). Consistent with the idea that activation of stemness genomic networks in cancer cells contributes to development of clinically-lethal death-from-cancer phenotypes, interrogation of regulatory networks of SCARS as well as Naïve and Primed hESC functional enhancers revealed a significant enrichment of cancer survival predictors’ genes that were defined for 17 distinct types of human malignancies (145). Similar regulatory connectivity has been observed for SCARS and cancer driver’s genes identified for 28 human cancer types [146]. Importantly, in all instances these analyses demonstrated that regulatory networks of SCARS and functional enhancers operating in hESC in both Naïve and Primed states appear to capture distinct arrays of genomic regulatory networks engaged in human embryogenesis, neurodevelopmental processes, and human malignancies. Consequently, collective considerations of all observations summarized in this contribution strongly argue that highly tractable experimental model systems tailored for precise structure-activity-phenotype interrogations of SCARS and functional enhancers in both Naïve and Primed hESC would represent a valuable, perhaps, indispensable, resource for dissections of principal genetic elements governing primate-specific and unique to human features of development, physiology, and pathology of Modern Humans.

From the clinical perspective, perhaps, reflecting the best interest of cancer patients, the most important translational impact of malignant regulatory signatures would be the reliable early diagnosis of sub-types of malignancies with the increased risk of existing therapy failure and high likelihood of death from cancer. It is this yet unfulfilled promise of malignant regulatory signatures defining stemness of human malignancies is the main focus of this contribution.

The predominant focus of the contemporary research effort on elucidation of molecular interconnectivity of the stemness phenotype and development of human cancers remains on the advancement of the cancer stem cell concept. The impact of recent remarkable advancements of single cell genomics of preimplantation human embryos, the bone fide source of the stemness phenotype creation during human development, had relatively modest influence on cancer research and, in particular, on progress in our understanding of mechanistic underpinning of malignant regulatory signatures. This contribution attempts to fill this void and stimulate the research effort comprehensively addressing potential translational implications of recent advances in single-cell genomics of human preimplantation embryogenesis.

The in-depth analyses of the critically important impact of SCARS as the essential elements of malignant regulatory signatures of clinically lethal human cancers will be one of the main topic of the future research. These studies should include precise identification and detailed structure-function analyses of individual transcriptionally-active regulatory genomic loci harboring SCARS and down-stream target genes making vital contributions to pathogenesis of human malignancies and multiple other common and rare disorders. Reflecting the critical role of epigenetic regulatory mechanisms at both DNA methylation and chromatin remodeling levels in SCARS silencing, the in-depth interrogation of specific epigenetic alterations causing the sustained activation of defined SCARS loci in various human disorders should be one of the major avenues of future laboratory and clinical investigations.

## Data Availability Statement

The original contributions presented in the study are included in the article/**Supplementary Material**. Further inquiries can be directed to the corresponding author.

## Author Contributions

This is a single author contribution. All elements of this work, including the conception of ideas, formulation, and development of concepts, execution of experiments, analysis of data, and writing of the paper, were performed by the author.

## Conflict of Interest

GG is co-founder of the OncoSCAR, LLC, early-stage privately-held company with the principal business goal of exploring translational utility of SCARS.
