# Supplementary material for: Genomics-Guided Drawing of Molecular and Pathophysiological Components of Malignant Regulatory Signatures Reveals a Pivotal Role in Human Diseases of Stem Cell-Associated Retroviral Sequences and Functionally-Active hESC Enhancers
Source: Front Oncol. 2021 Mar 31;11:638363. doi: 10.3389/fonc.2021.638363 (PMC8044830; doi:10.3389/fonc.2021.638363)
Supplement: Supplementary file 1 [file Presentation_1.zip › Supplemental Figure S1. Distinct populations of TERTpos cells.pptx]

## Slide 1
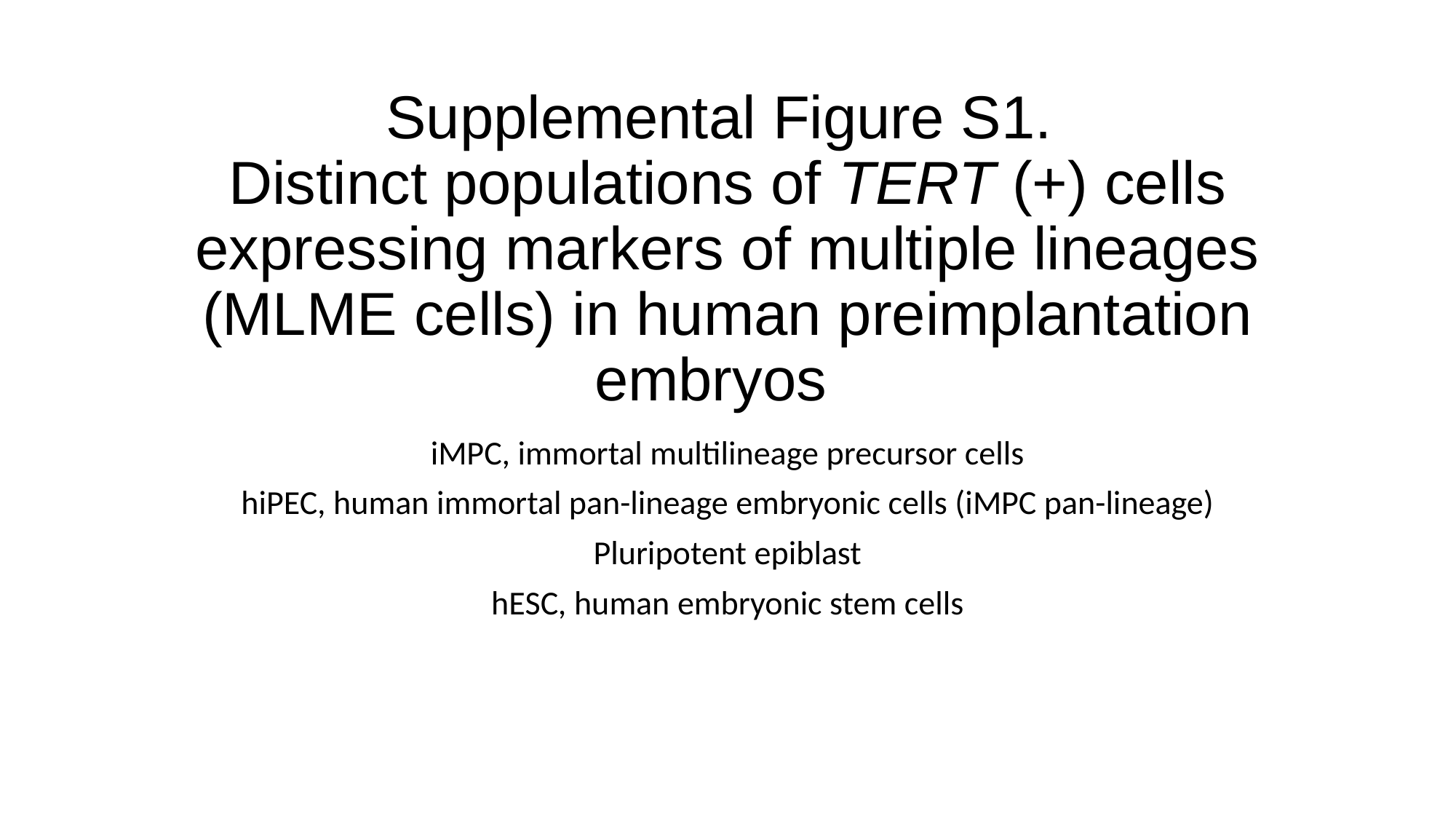

# Supplemental Figure S1. Distinct populations of TERT (+) cells expressing markers of multiple lineages (MLME cells) in human preimplantation embryos
iMPC, immortal multilineage precursor cells
hiPEC, human immortal pan-lineage embryonic cells (iMPC pan-lineage)
Pluripotent epiblast
hESC, human embryonic stem cells

## Slide 2
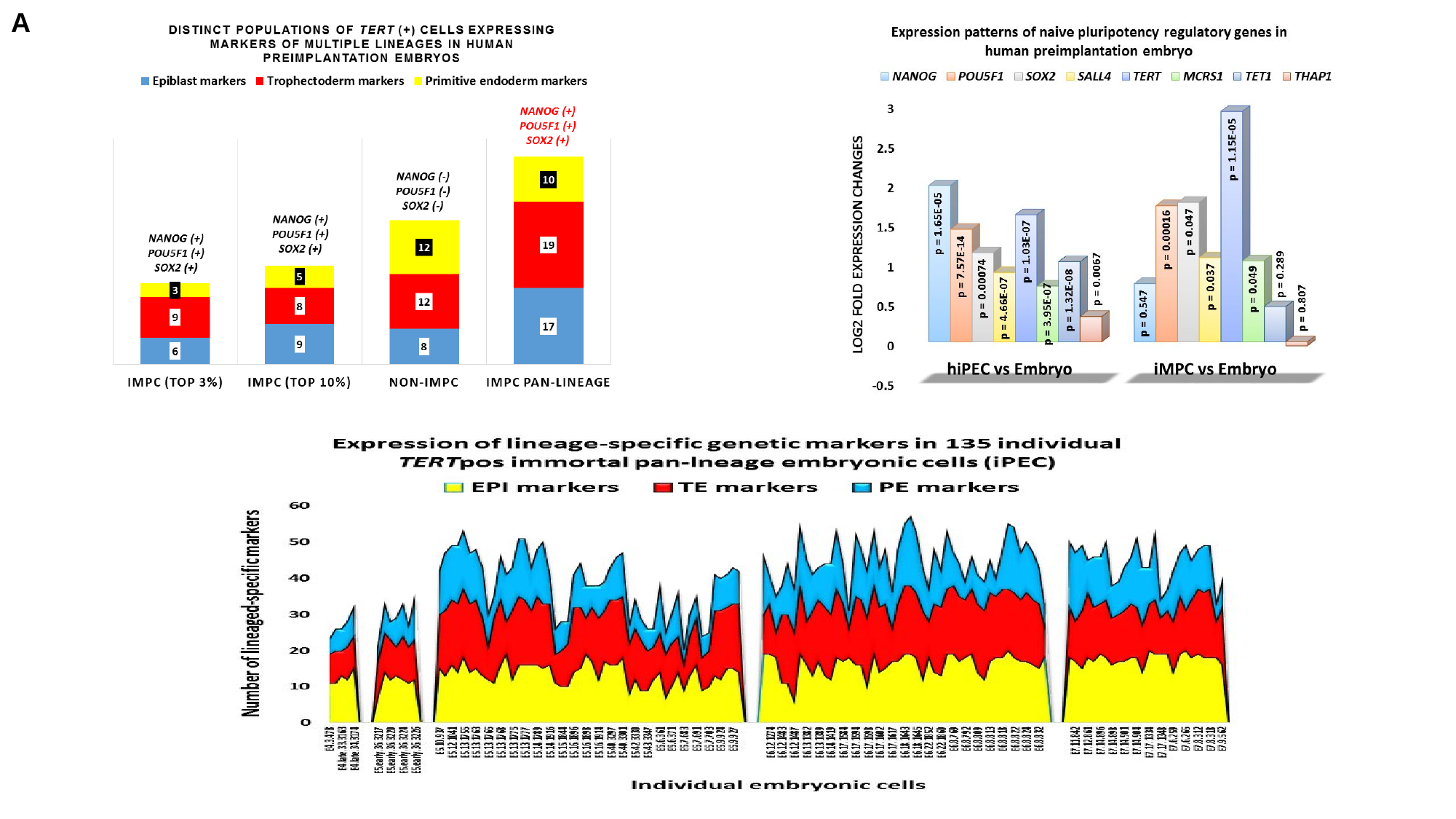

A

## Slide 3
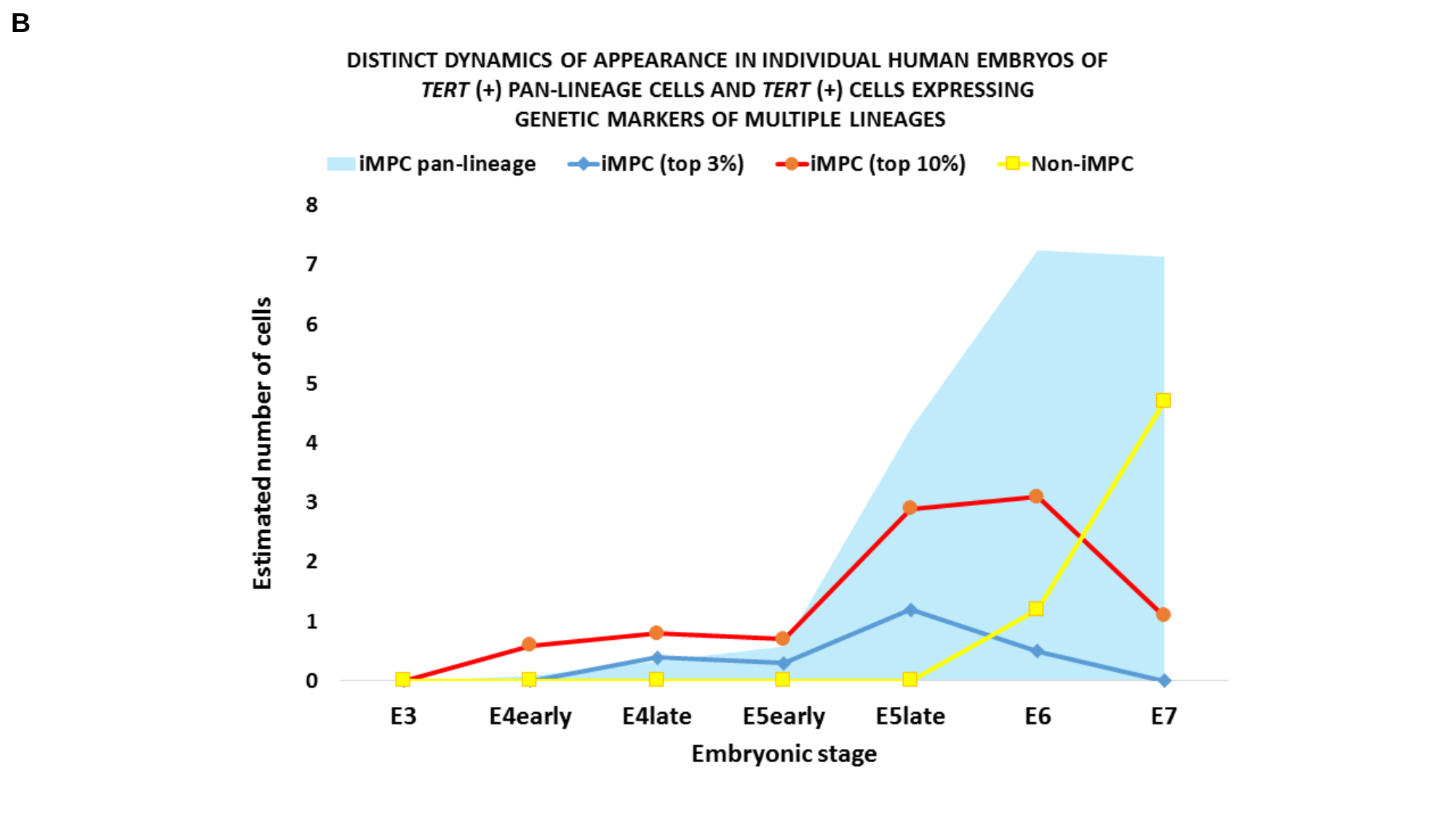

B

## Slide 4
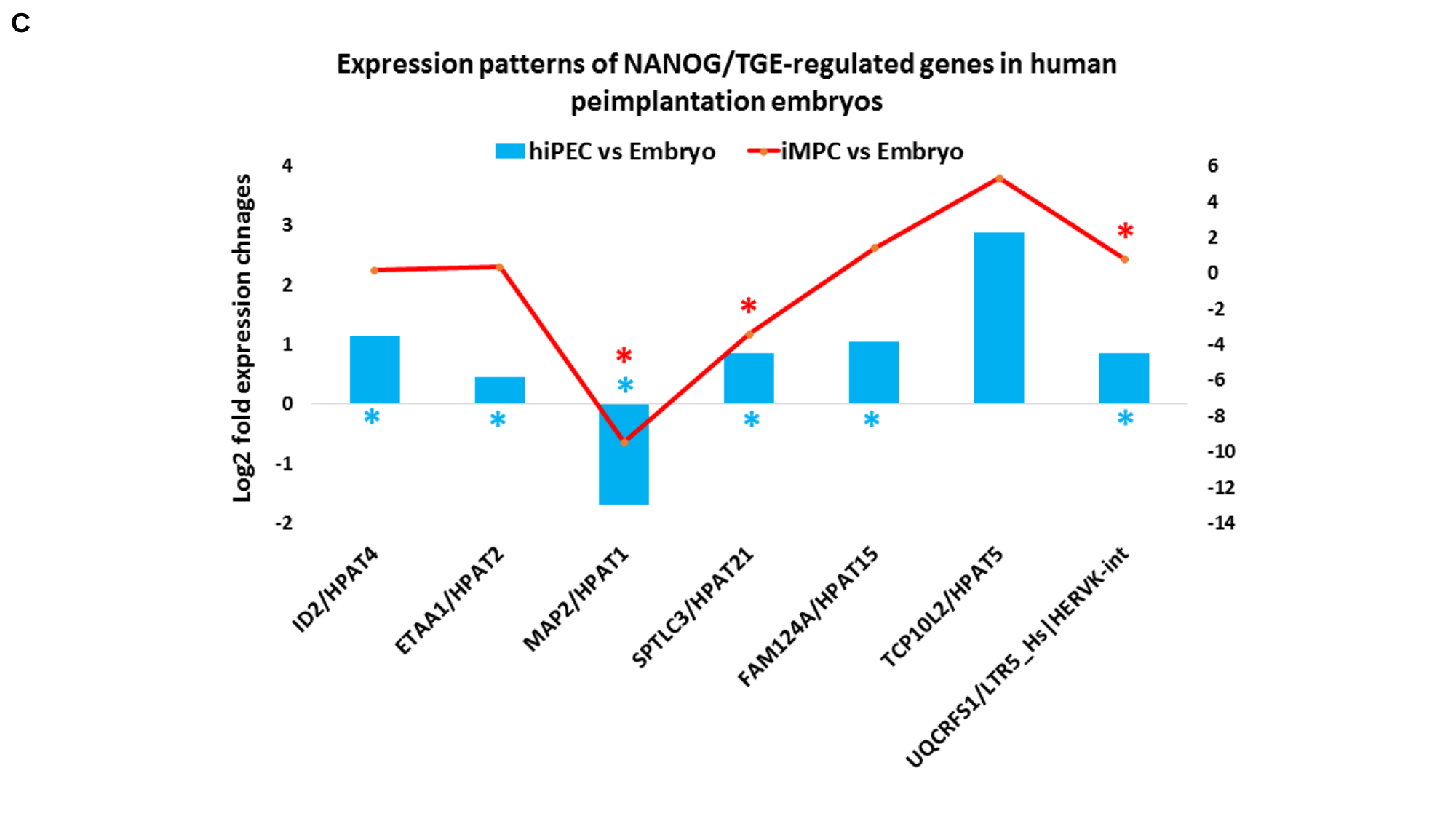

C

## Slide 5
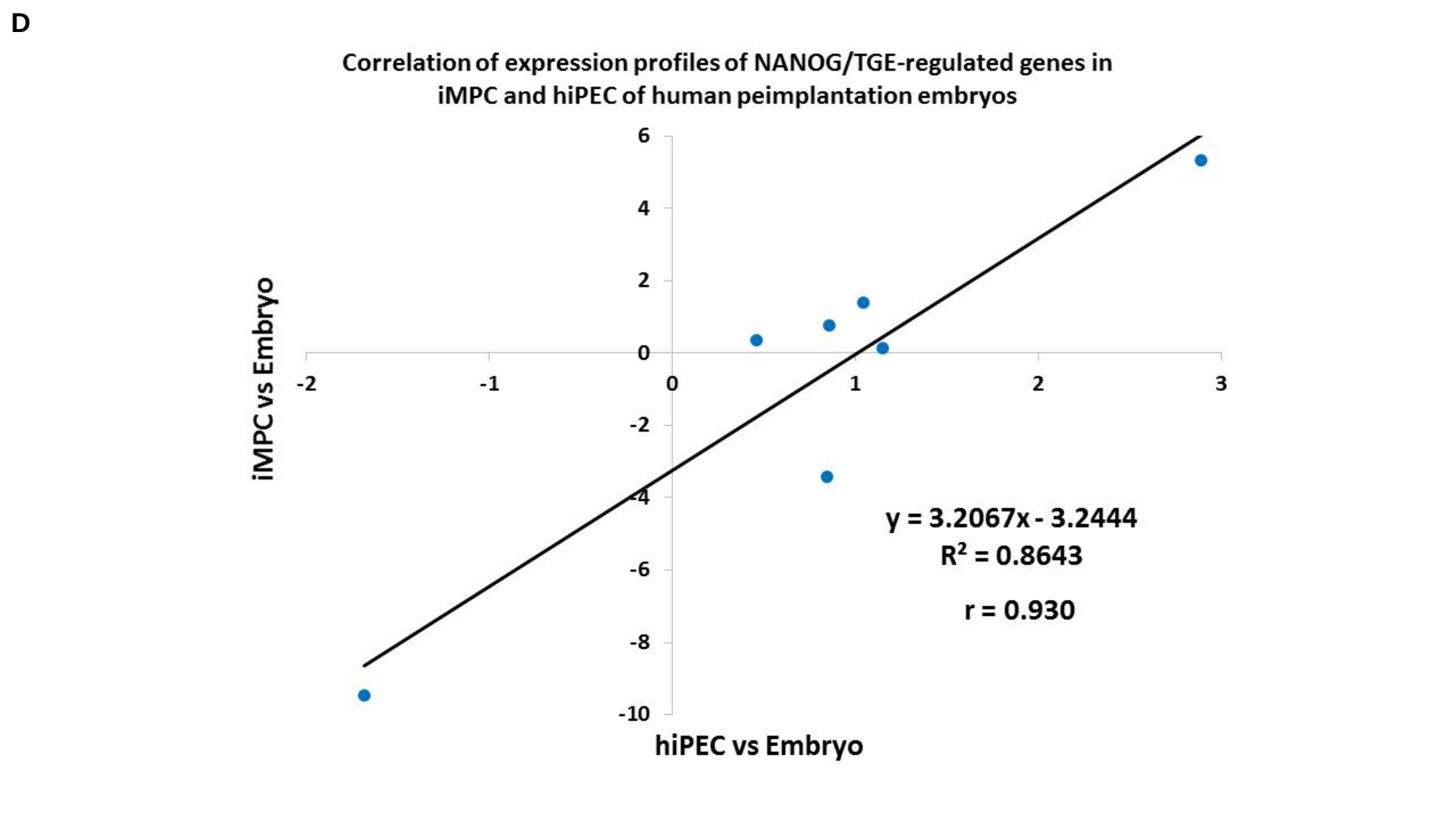

D

## Slide 6
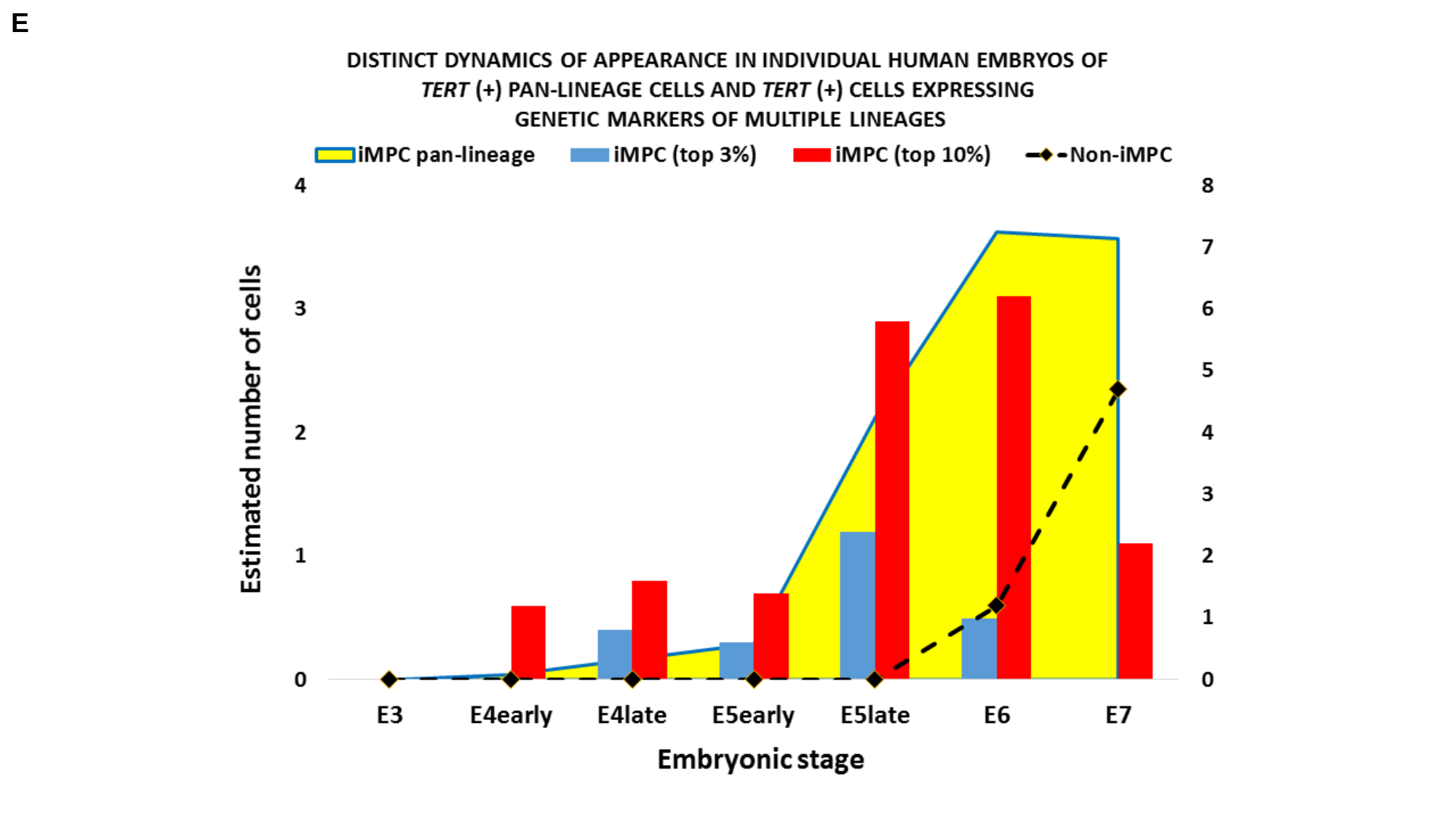

E
