# Supplementary material for: Genomics-Guided Drawing of Molecular and Pathophysiological Components of Malignant Regulatory Signatures Reveals a Pivotal Role in Human Diseases of Stem Cell-Associated Retroviral Sequences and Functionally-Active hESC Enhancers
Source: Front Oncol. 2021 Mar 31;11:638363. doi: 10.3389/fonc.2021.638363 (PMC8044830; doi:10.3389/fonc.2021.638363)
Supplement: Supplementary file 1 [file Presentation_1.zip › Supplemental Figure S2. TE loci MLME vs Embryo and Naïve vs Primed.pptx]

## Slide 1
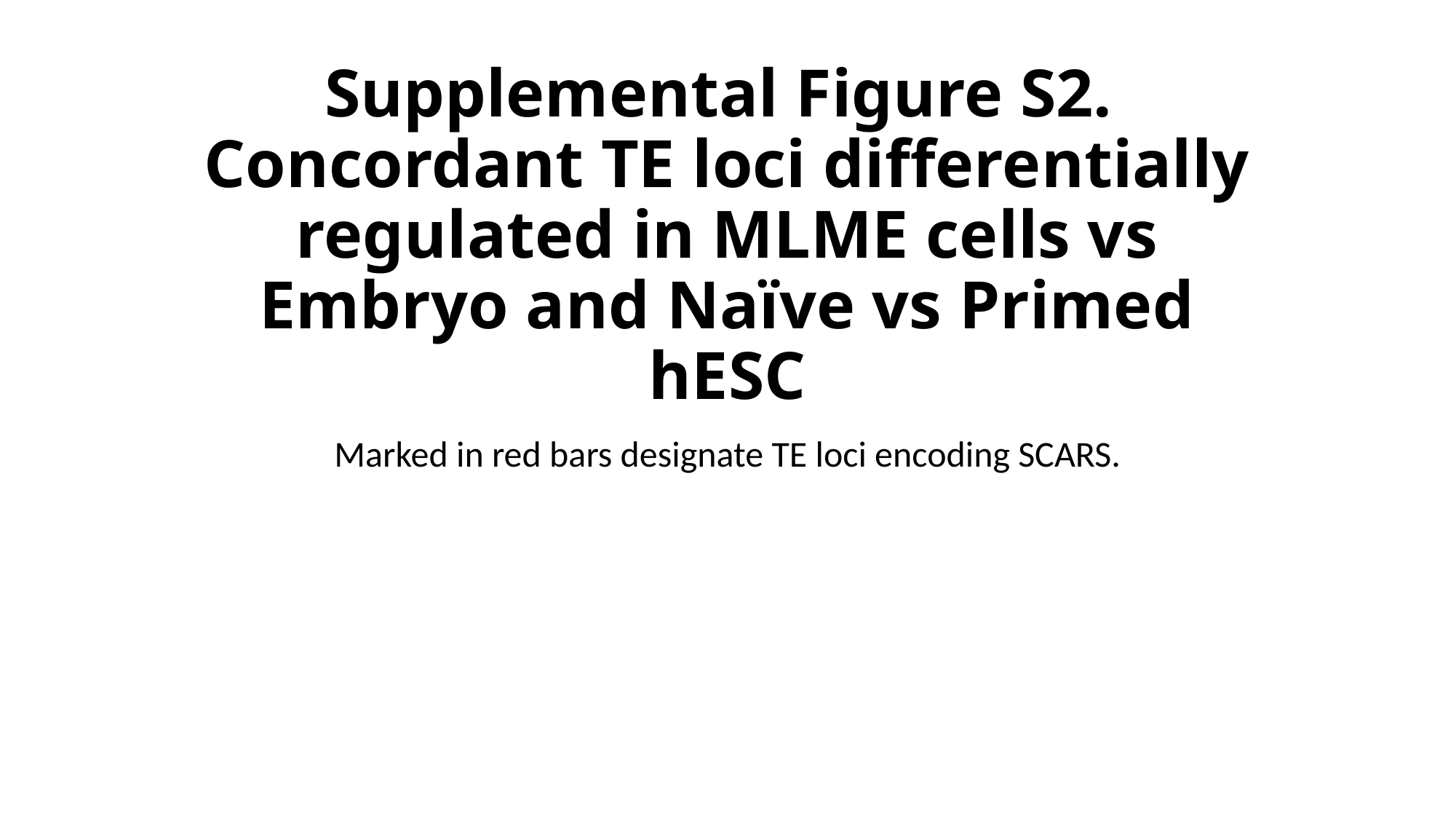

# Supplemental Figure S2. Concordant TE loci differentially regulated in MLME cells vs Embryo and Naïve vs Primed hESC
Marked in red bars designate TE loci encoding SCARS.

## Slide 2
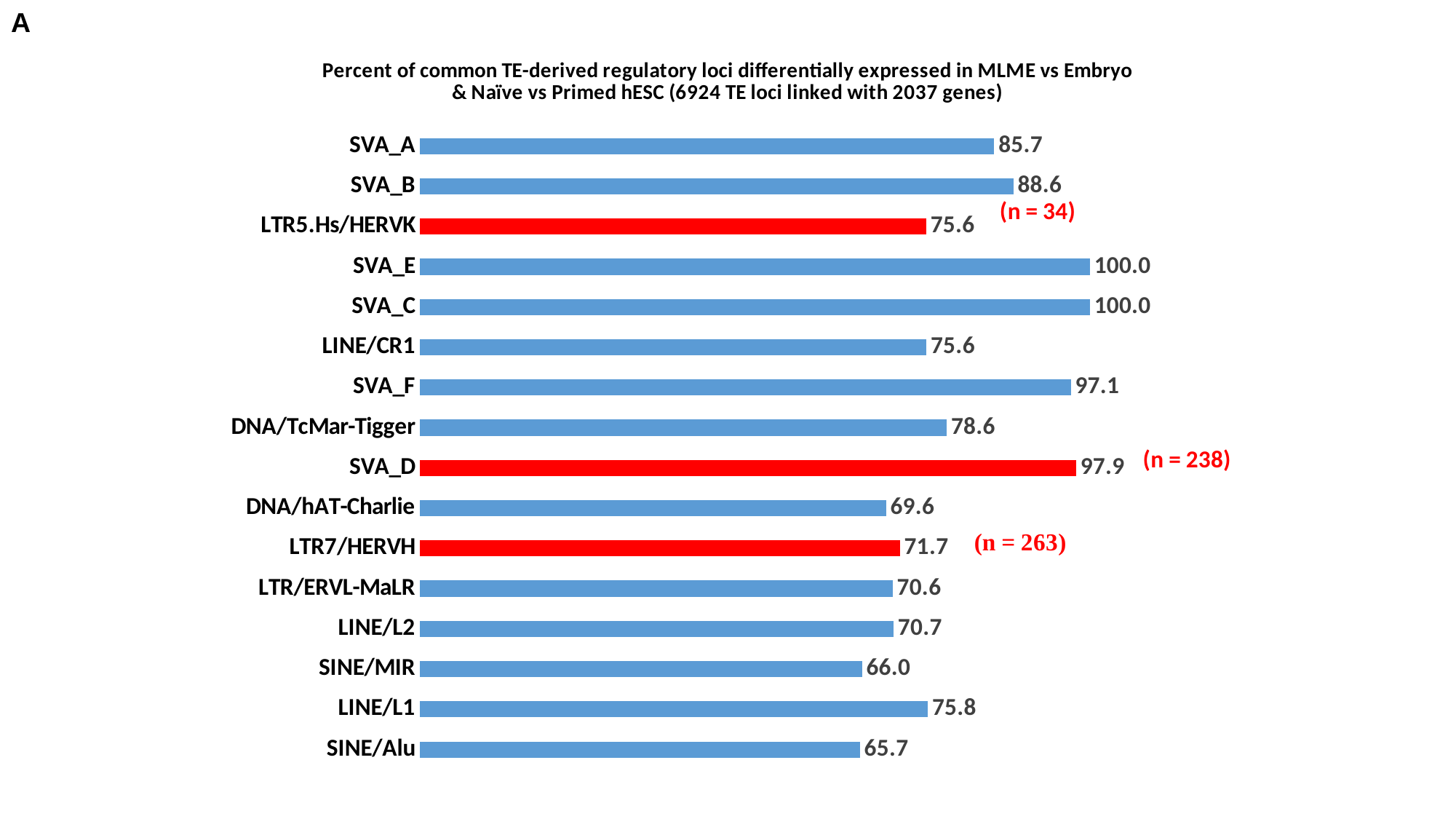

A
### Chart: Percent of common TE-derived regulatory loci differentially expressed in MLME vs Embryo & Naïve vs Primed hESC (6924 TE loci linked with 2037 genes)
| Category | Percent of common TE-derived regulatory loci differentially expressed in iMPC vs Embryo & Naïve vs Primed hESC (6924 TE loci linked with 2037 genes) |
|---|---|
| SINE/Alu | 65.69195136074117 |
| LINE/L1 | 75.83011583011583 |
| SINE/MIR | 65.99799398194584 |
| LINE/L2 | 70.71428571428572 |
| LTR/ERVL-MaLR | 70.56451612903226 |
| LTR7/HERVH | 71.66212534059946 |
| DNA/hAT-Charlie | 69.58637469586375 |
| SVA_D | 97.94238683127571 |
| DNA/TcMar-Tigger | 78.63636363636364 |
| SVA_F | 97.14285714285714 |
| LINE/CR1 | 75.60975609756098 |
| SVA_C | 100.0 |
| SVA_E | 100.0 |
| LTR5.Hs/HERVK | 75.55555555555556 |
| SVA_B | 88.57142857142857 |
| SVA_A | 85.71428571428571 |

## Slide 3
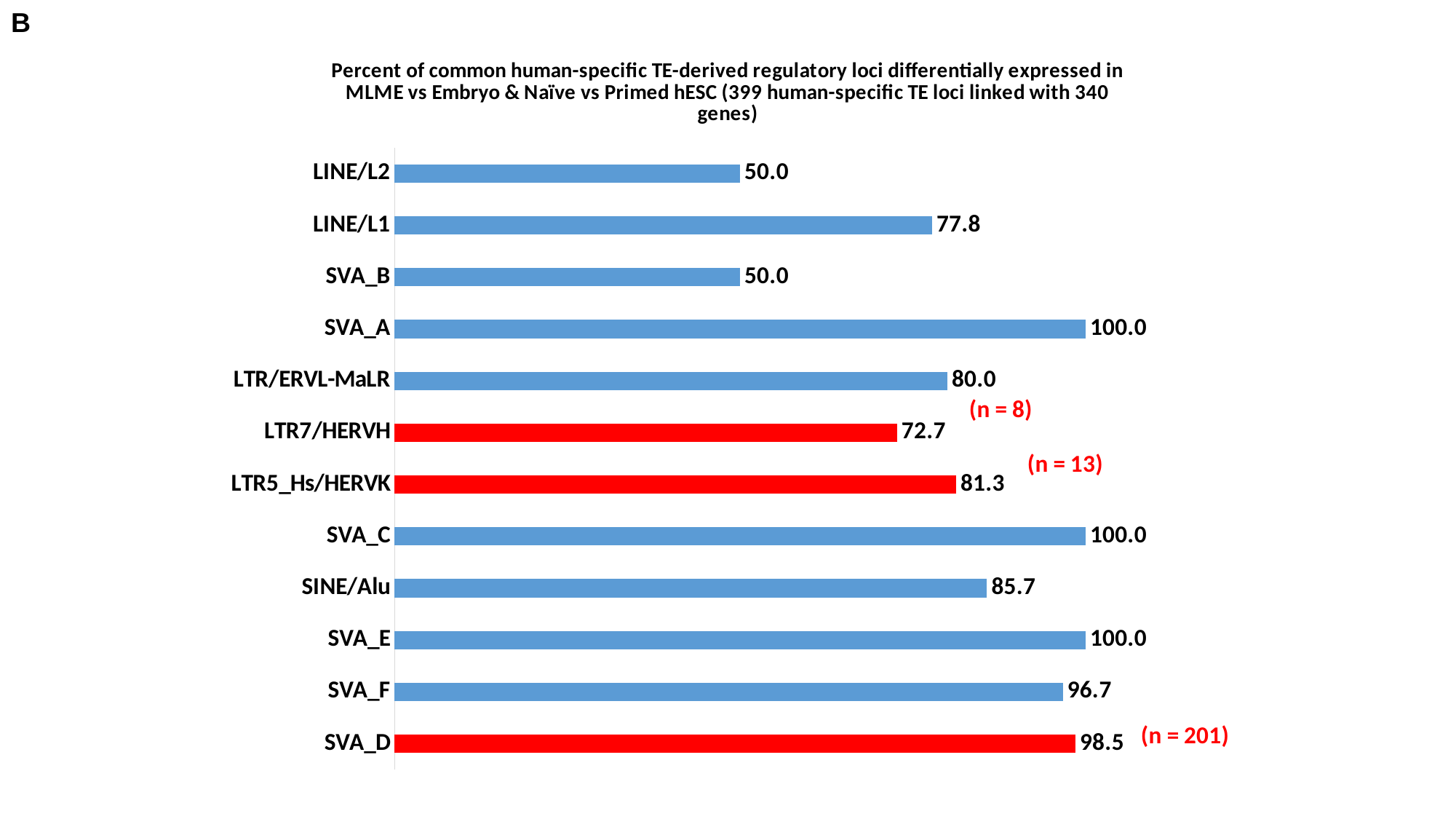

B
### Chart: Percent of common human-specific TE-derived regulatory loci differentially expressed in MLME vs Embryo & Naïve vs Primed hESC (399 human-specific TE loci linked with 340 genes)
| Category | Percent of common human-specific TE-derived regulatory loci differentially expressed in iMPC vs Embryo & Naïve vs Primed hESC |
|---|---|
| SVA_D | 98.52941176470588 |
| SVA_F | 96.72131147540983 |
| SVA_E | 100.0 |
| SINE/Alu | 85.71428571428571 |
| SVA_C | 100.0 |
| LTR5_Hs/HERVK | 81.25 |
| LTR7/HERVH | 72.72727272727273 |
| LTR/ERVL-MaLR | 80.0 |
| SVA_A | 100.0 |
| SVA_B | 50.0 |
| LINE/L1 | 77.77777777777779 |
| LINE/L2 | 50.0 |

## Slide 4
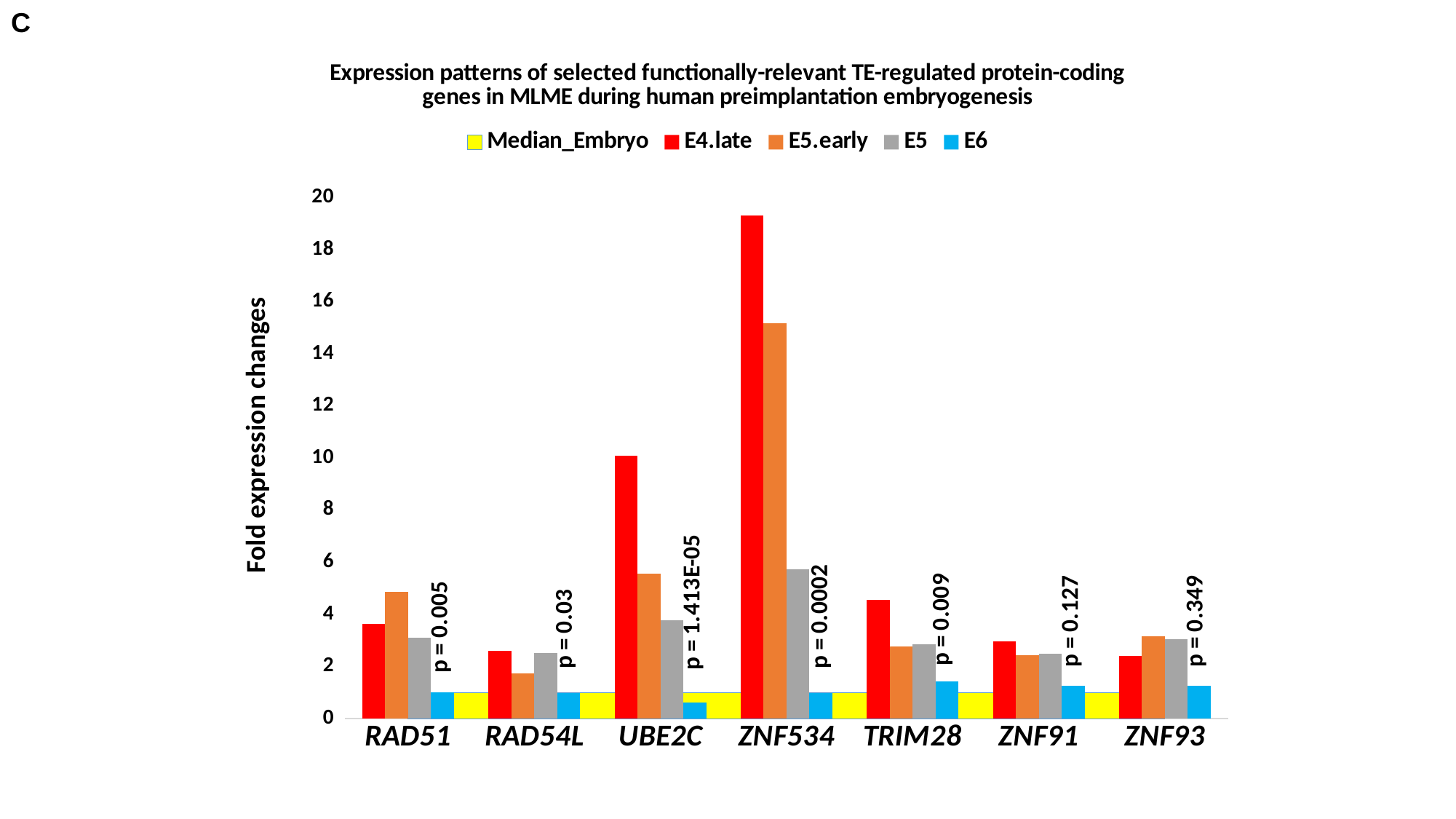

C
### Chart: Expression patterns of selected functionally-relevant TE-regulated protein-coding genes in MLME during human preimplantation embryogenesis
| Category | Median_Embryo | E4.late | E5.early | E5 | E6 |
|---|---|---|---|---|---|
| RAD51 | 1.0 | 3.6242990654205607 | 4.862928348909658 | 3.0997997329773033 | 1.0023364485981308 |
| RAD54L | 1.0 | 2.598412698412698 | 1.7301587301587302 | 2.518707482993197 | 0.9867724867724867 |
| UBE2C | 1.0 | 10.105978705978705 | 5.559377559377559 | 3.7894582894582896 | 0.6063336063336063 |
| ZNF534 | 1.0 | 19.32 | 15.2 | 5.742857142857144 | 0.9733333333333333 |
| TRIM28 | 1.0 | 4.566953199617957 | 2.754855141674626 | 2.8458862054850593 | 1.4333014963387456 |
| ZNF91 | 1.0 | 2.9722222222222223 | 2.435185185185185 | 2.488095238095238 | 1.2685185185185184 |
| ZNF93 | 1.0 | 2.415686274509804 | 3.1546840958605666 | 3.0560224089635852 | 1.2679738562091503 |
