# Supplementary material for: Genomics-Guided Drawing of Molecular and Pathophysiological Components of Malignant Regulatory Signatures Reveals a Pivotal Role in Human Diseases of Stem Cell-Associated Retroviral Sequences and Functionally-Active hESC Enhancers
Source: Front Oncol. 2021 Mar 31;11:638363. doi: 10.3389/fonc.2021.638363 (PMC8044830; doi:10.3389/fonc.2021.638363)
Supplement: Supplementary file 1 [file Presentation_1.zip › Supplemental Figure S3. SCARS Protein binding and mRNA expression.pptx]

## Slide 1
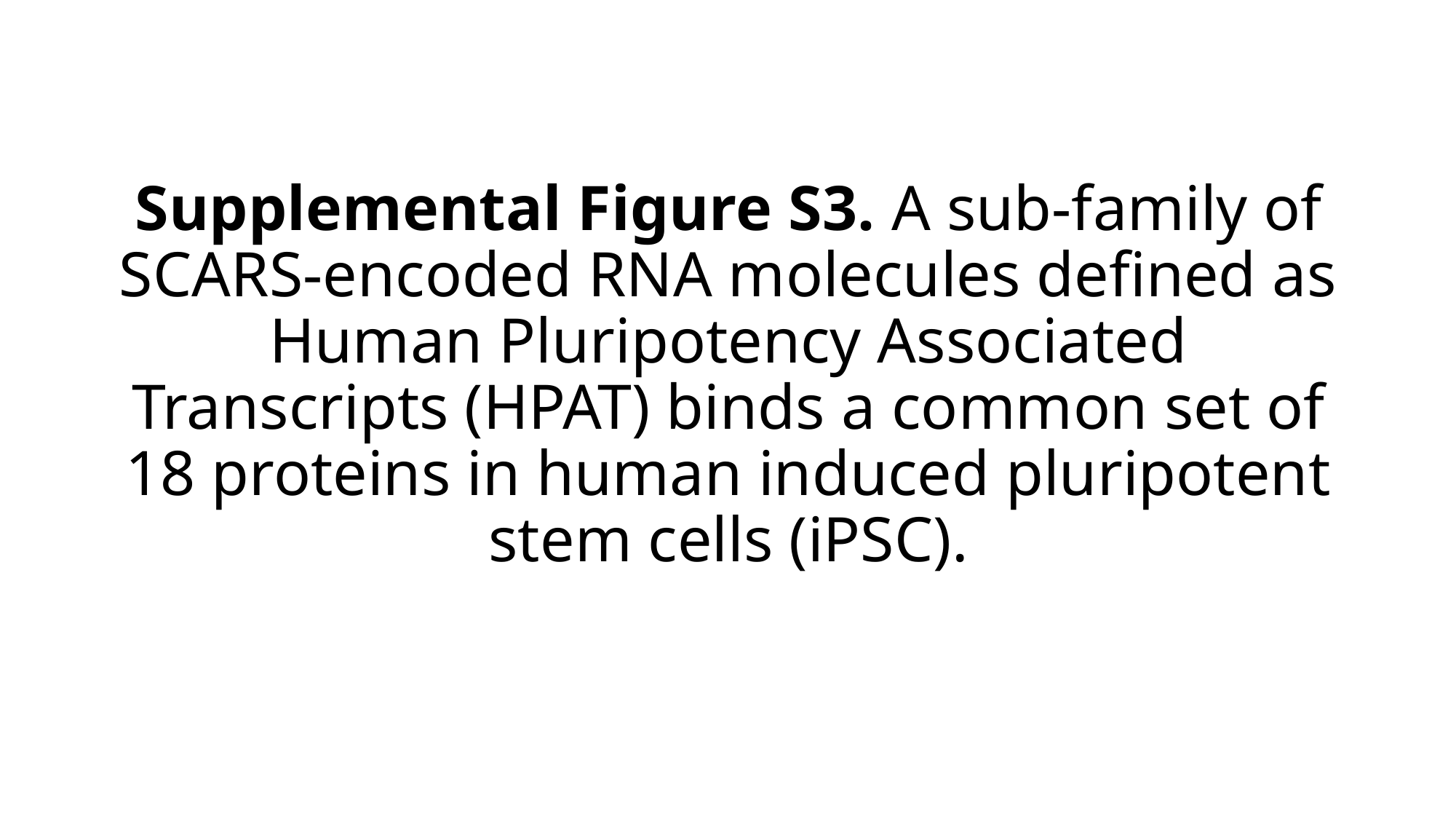

# Supplemental Figure S3. A sub-family of SCARS-encoded RNA molecules defined as Human Pluripotency Associated Transcripts (HPAT) binds a common set of 18 proteins in human induced pluripotent stem cells (iPSC).

## Slide 2
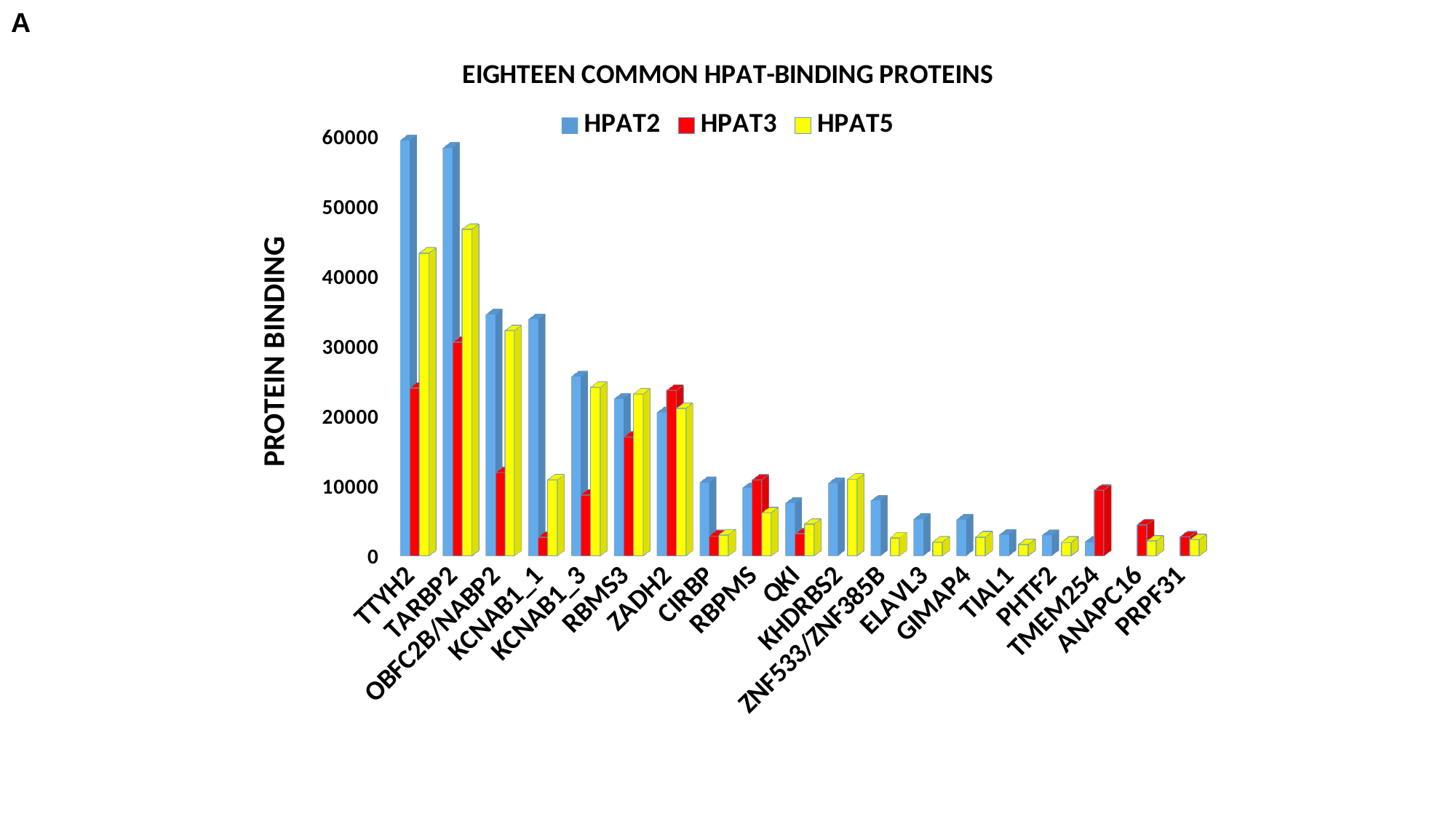

A
[unsupported chart]

## Slide 3
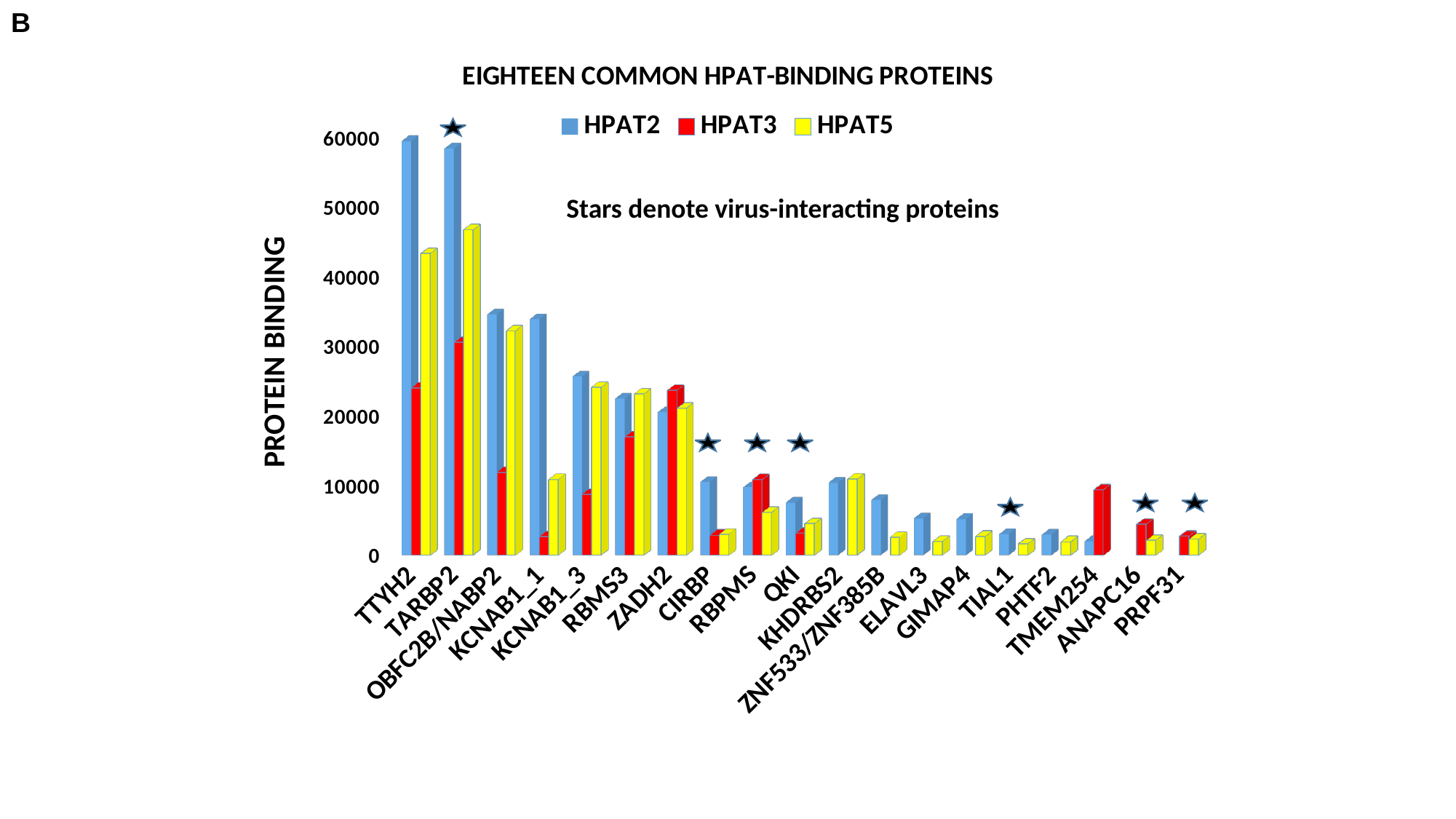

B
[unsupported chart]
Stars denote virus-interacting proteins

## Slide 4
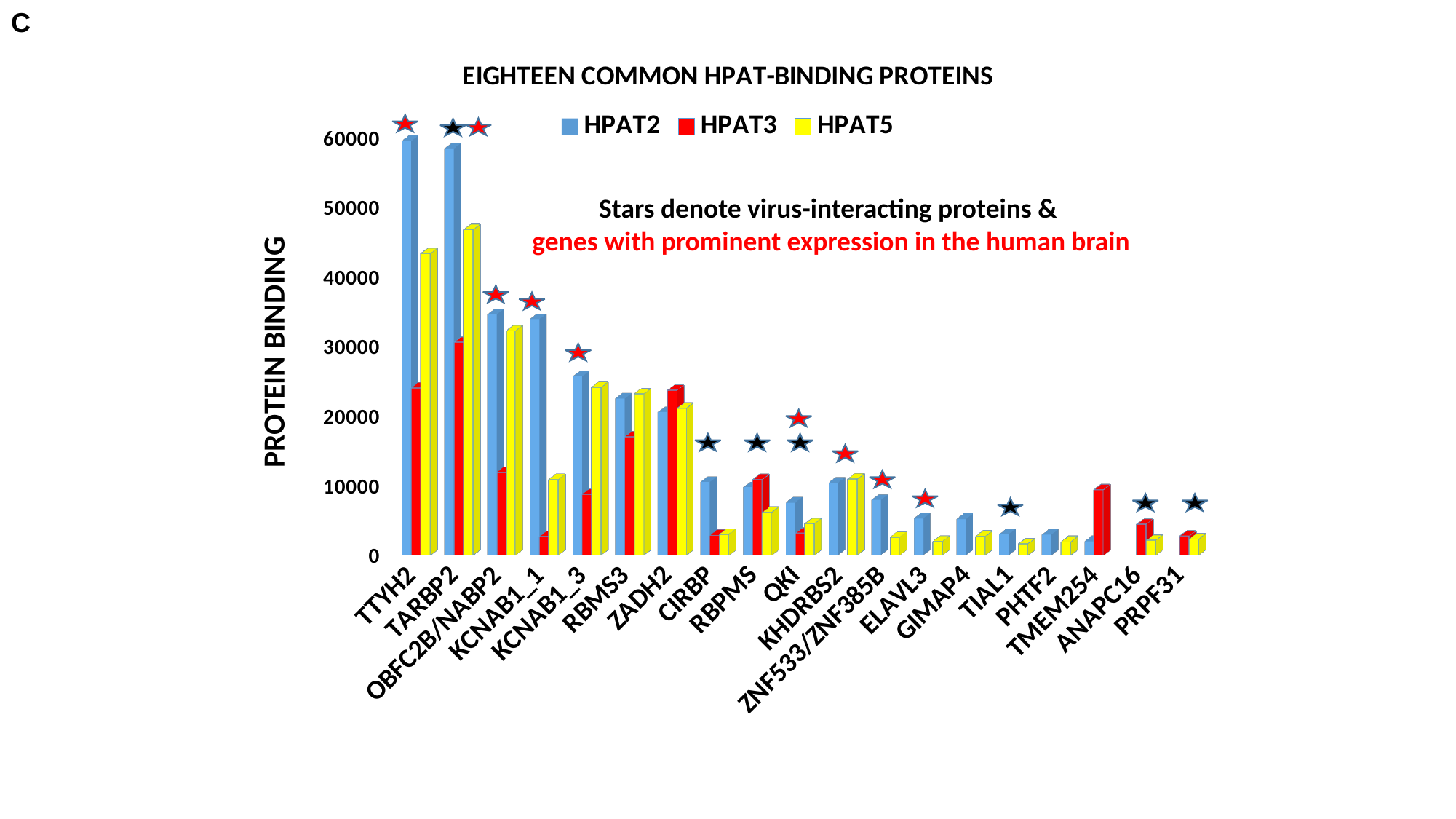

C
[unsupported chart]
Stars denote virus-interacting proteins &
genes with prominent expression in the human brain

## Slide 5
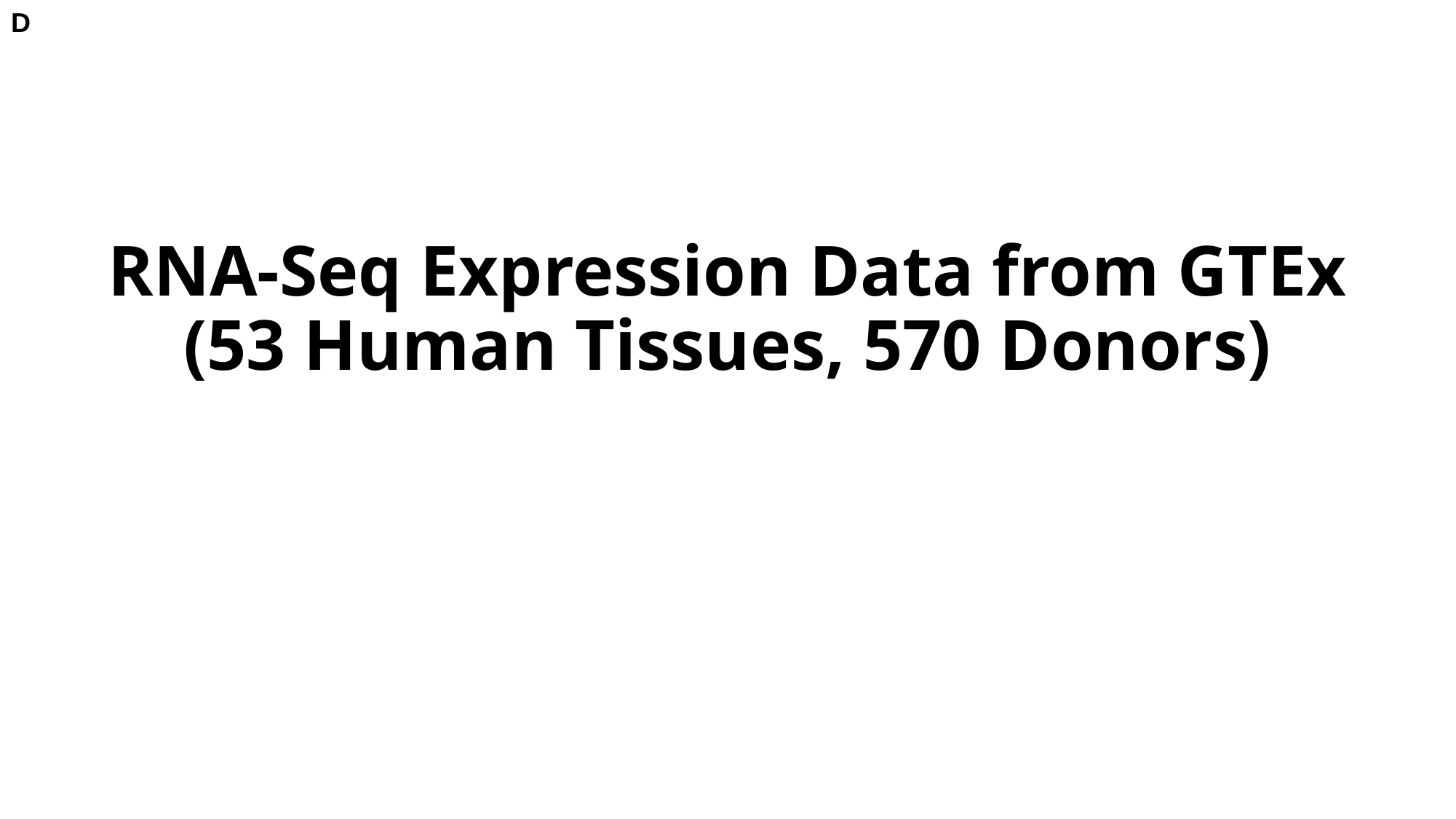

D
# RNA-Seq Expression Data from GTEx (53 Human Tissues, 570 Donors)

## Slide 6
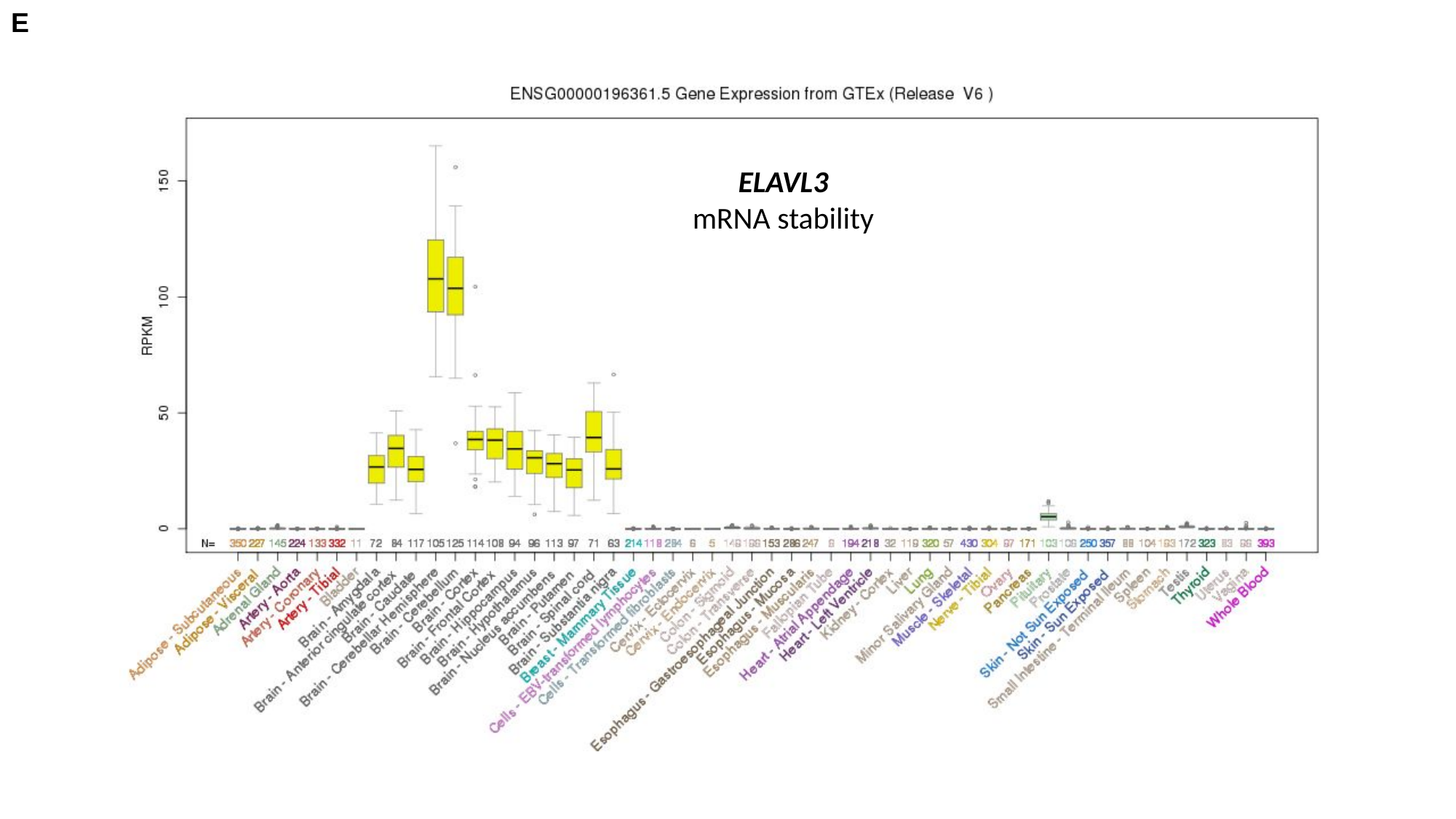

E
ELAVL3
mRNA stability

## Slide 7
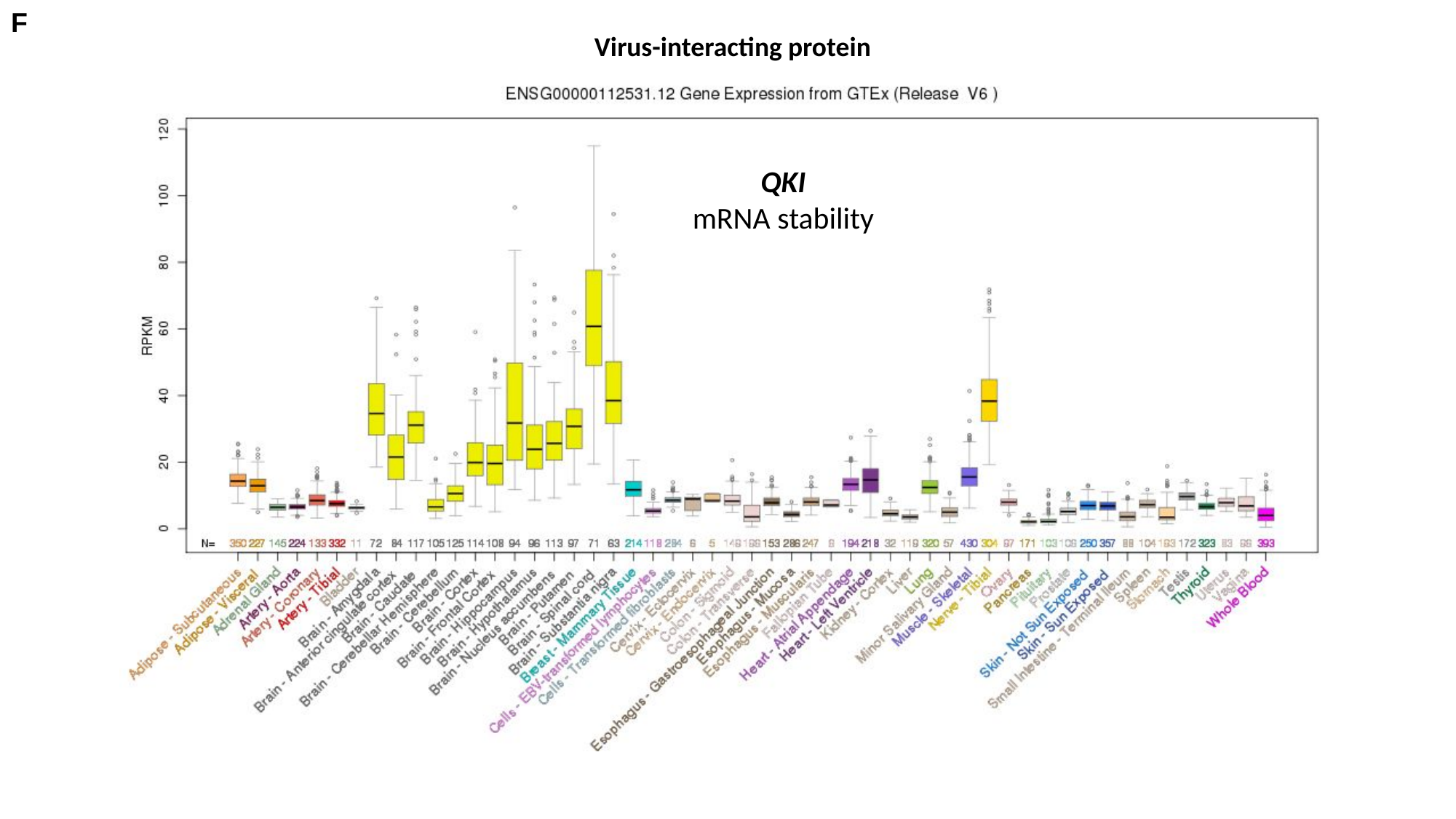

F
Virus-interacting protein
QKI
mRNA stability

## Slide 8
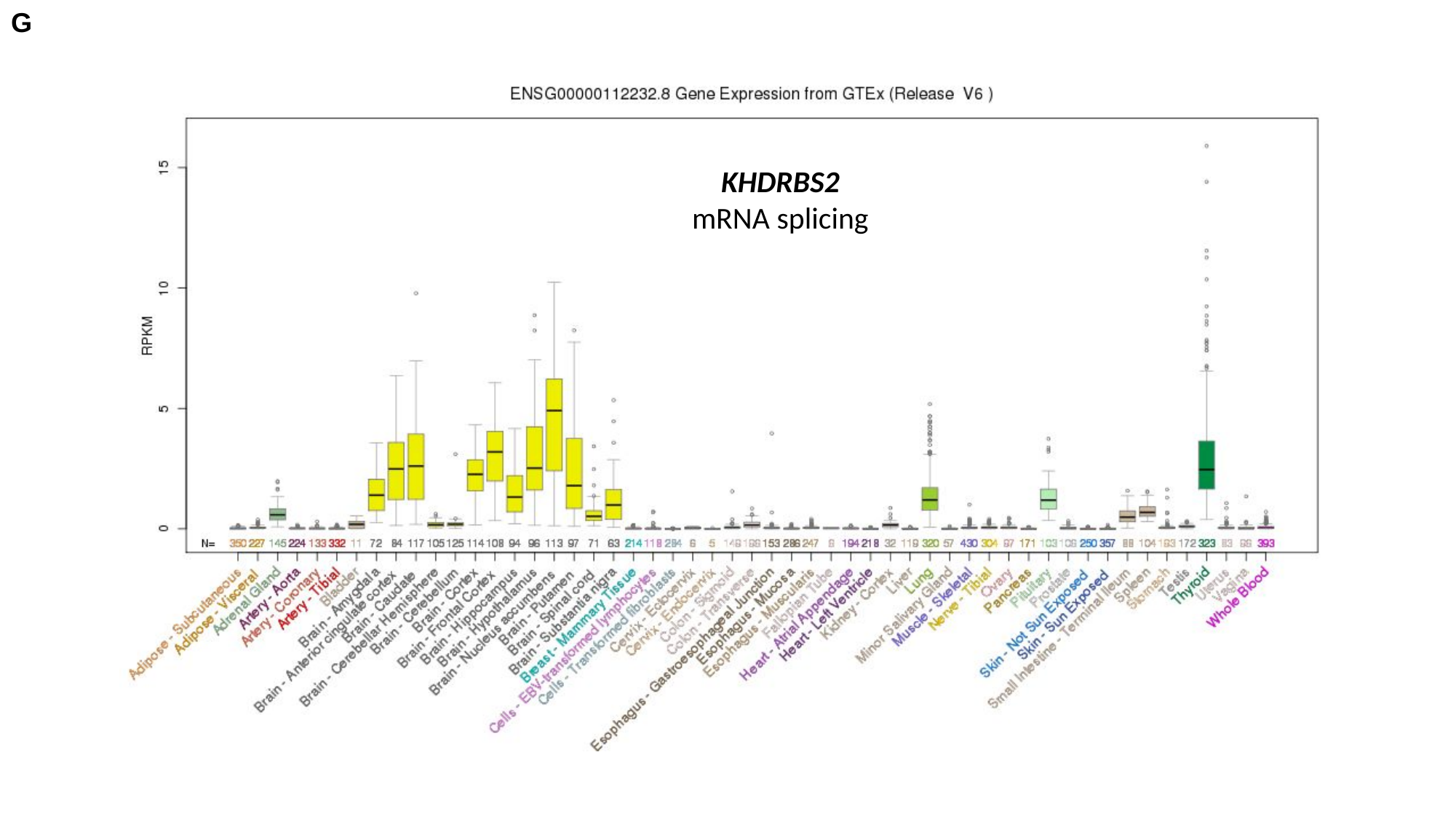

G
KHDRBS2
mRNA splicing

## Slide 9
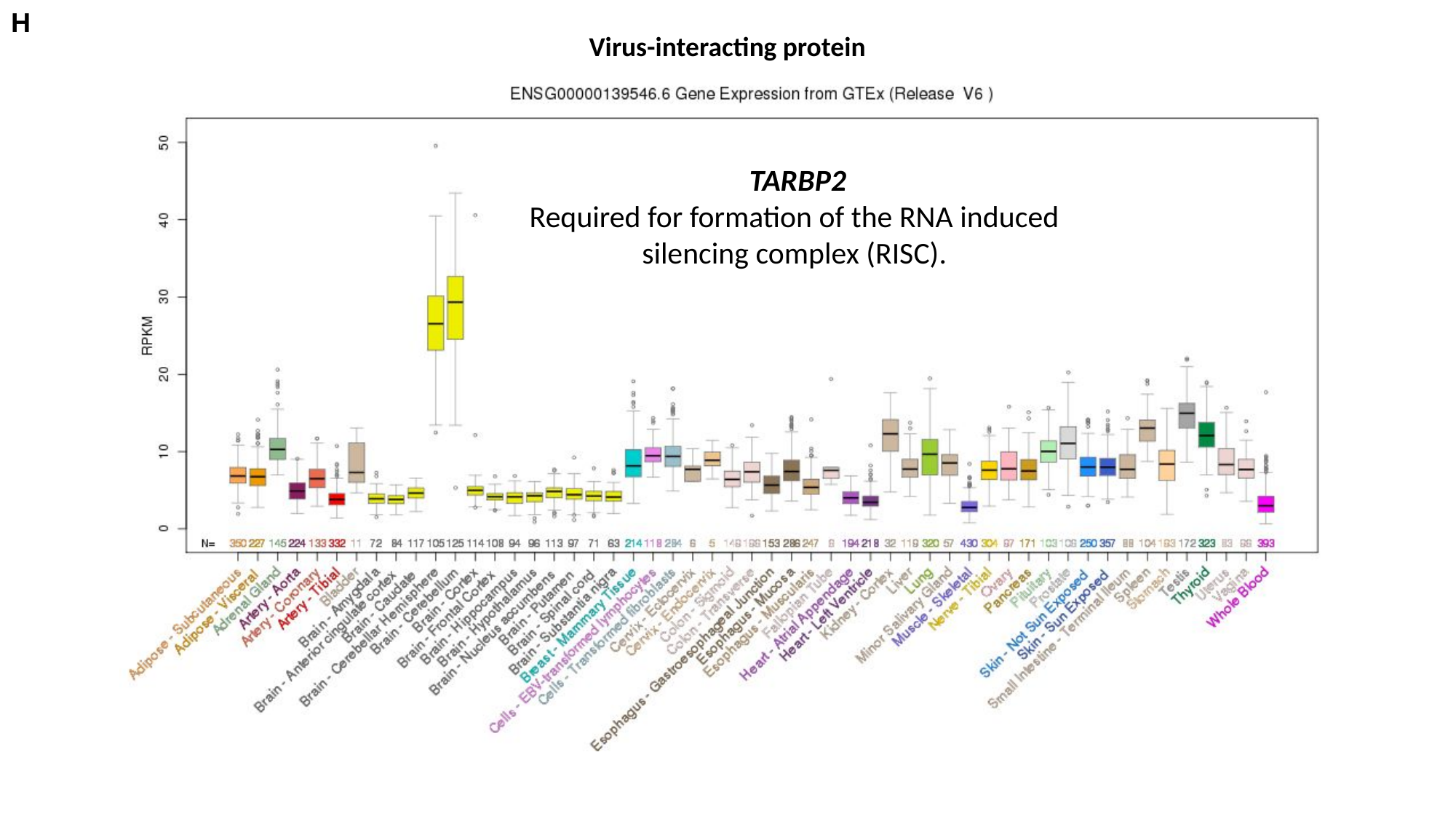

H
Virus-interacting protein
TARBP2
Required for formation of the RNA induced
silencing complex (RISC).

## Slide 10
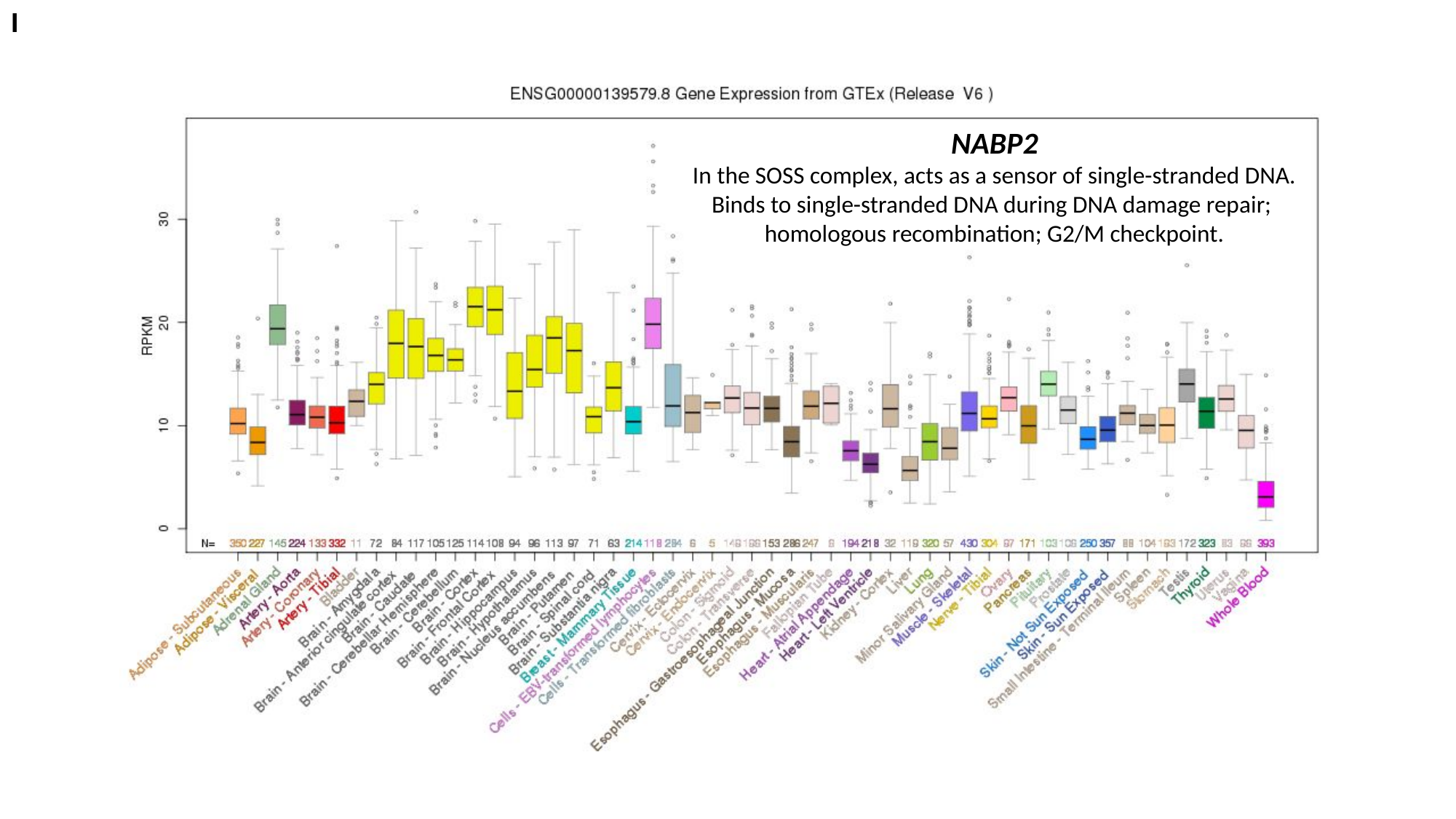

I
NABP2
In the SOSS complex, acts as a sensor of single-stranded DNA.
Binds to single-stranded DNA during DNA damage repair;
homologous recombination; G2/M checkpoint.

## Slide 11
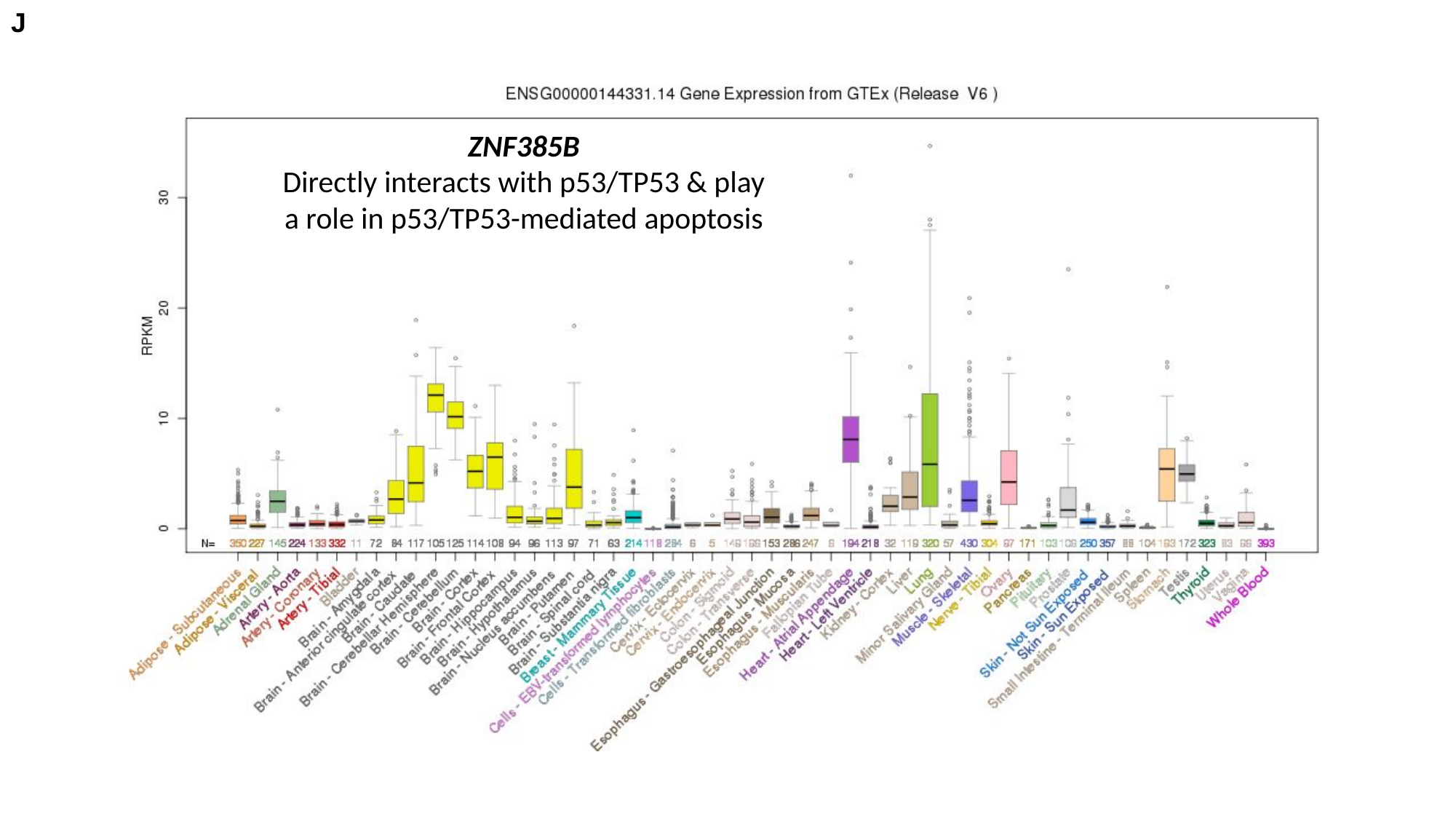

J
ZNF385B
Directly interacts with p53/TP53 & play
a role in p53/TP53-mediated apoptosis

## Slide 12
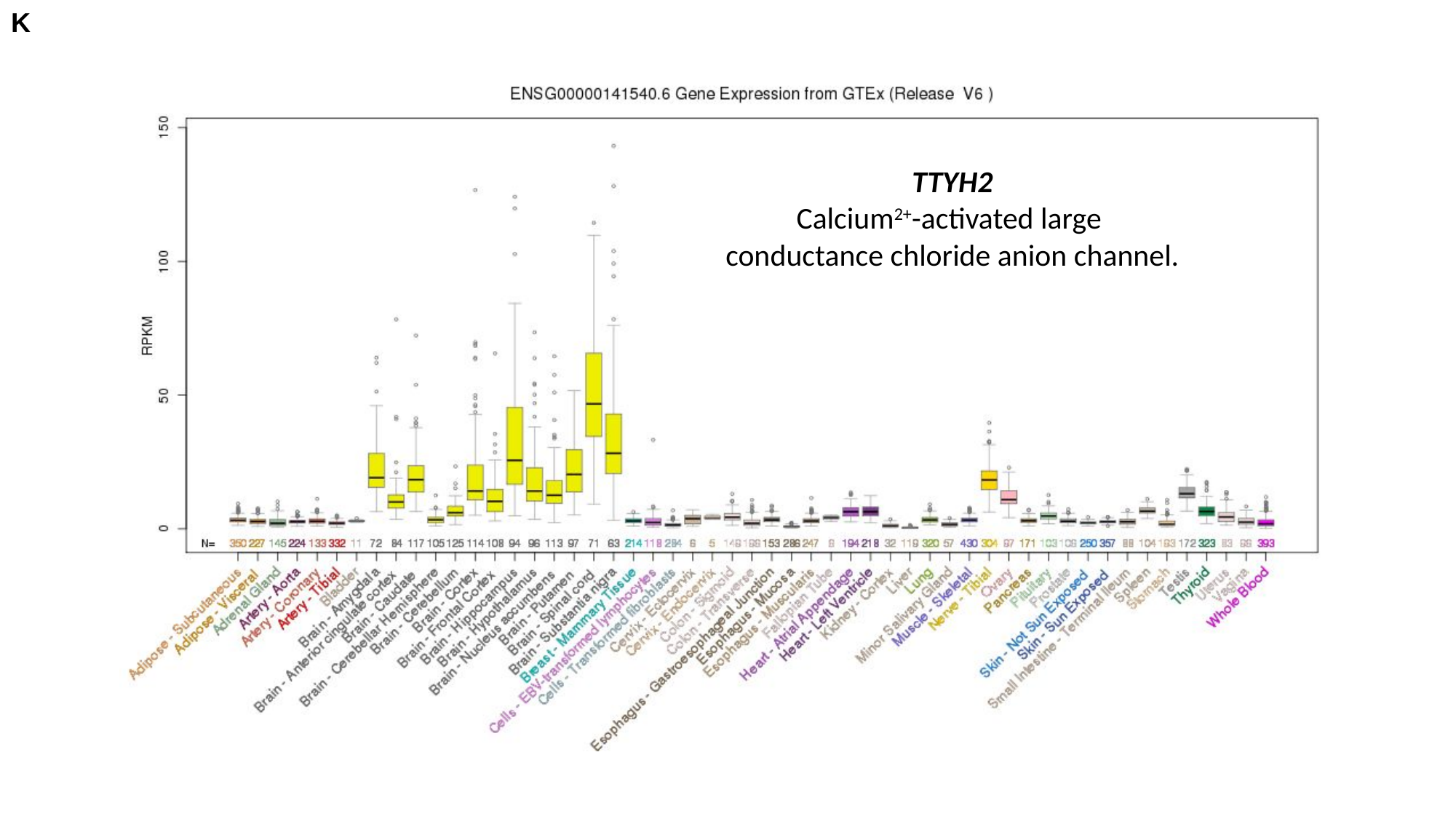

K
TTYH2
Calcium2+-activated large
conductance chloride anion channel.

## Slide 13
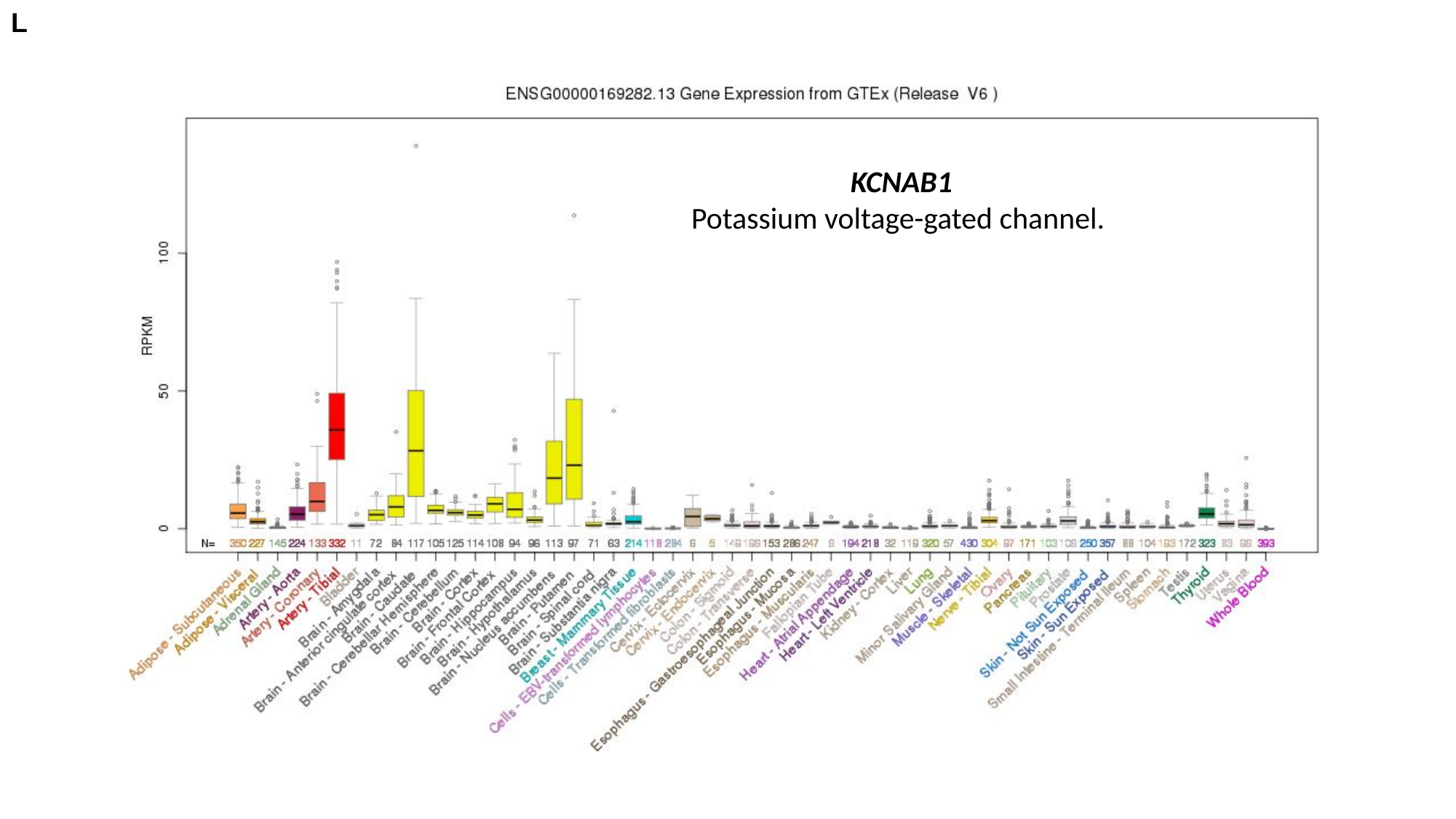

L
KCNAB1
Potassium voltage-gated channel.

## Slide 14
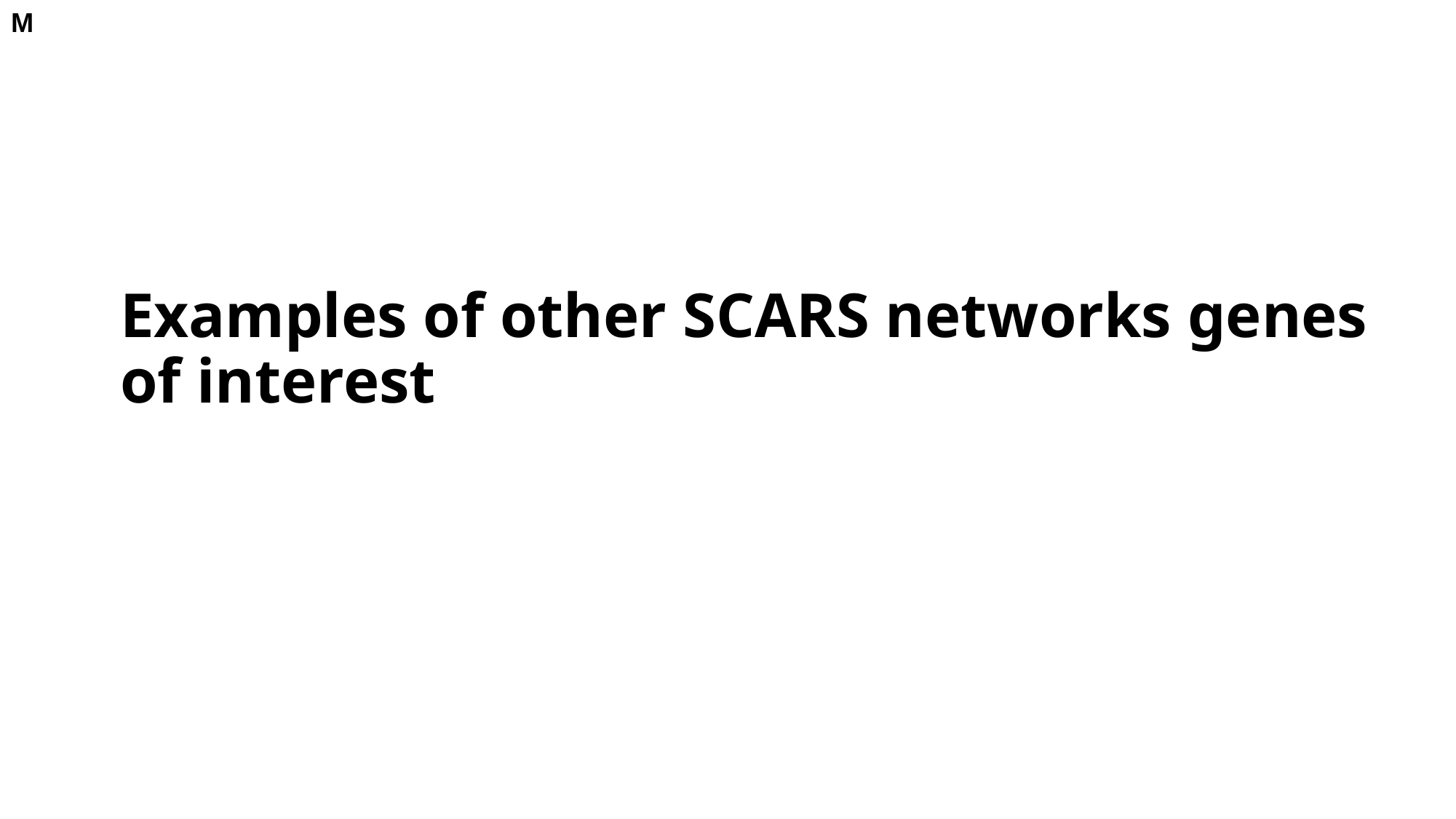

M
# Examples of other SCARS networks genes of interest

## Slide 15
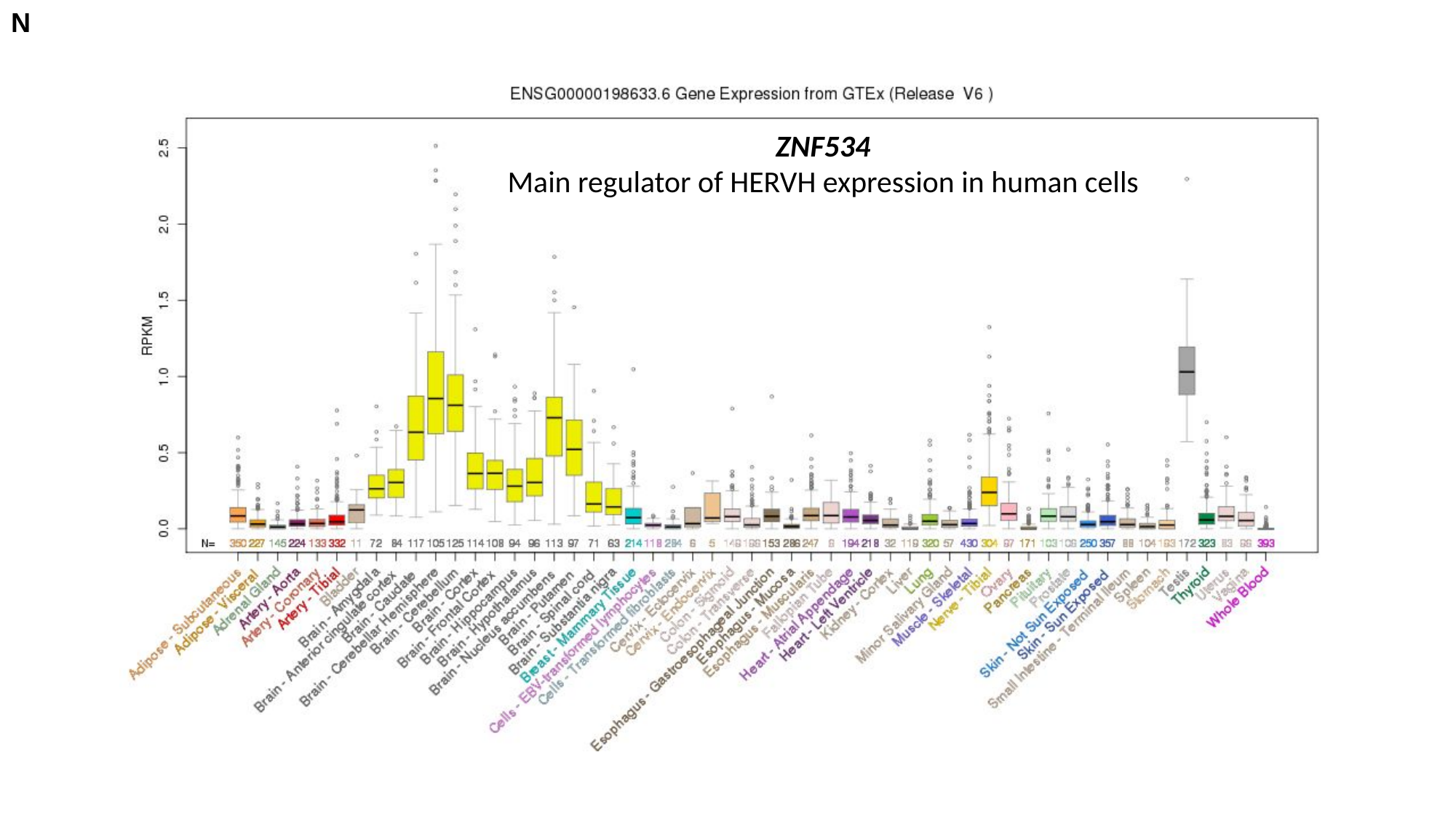

N
ZNF534
Main regulator of HERVH expression in human cells

## Slide 16
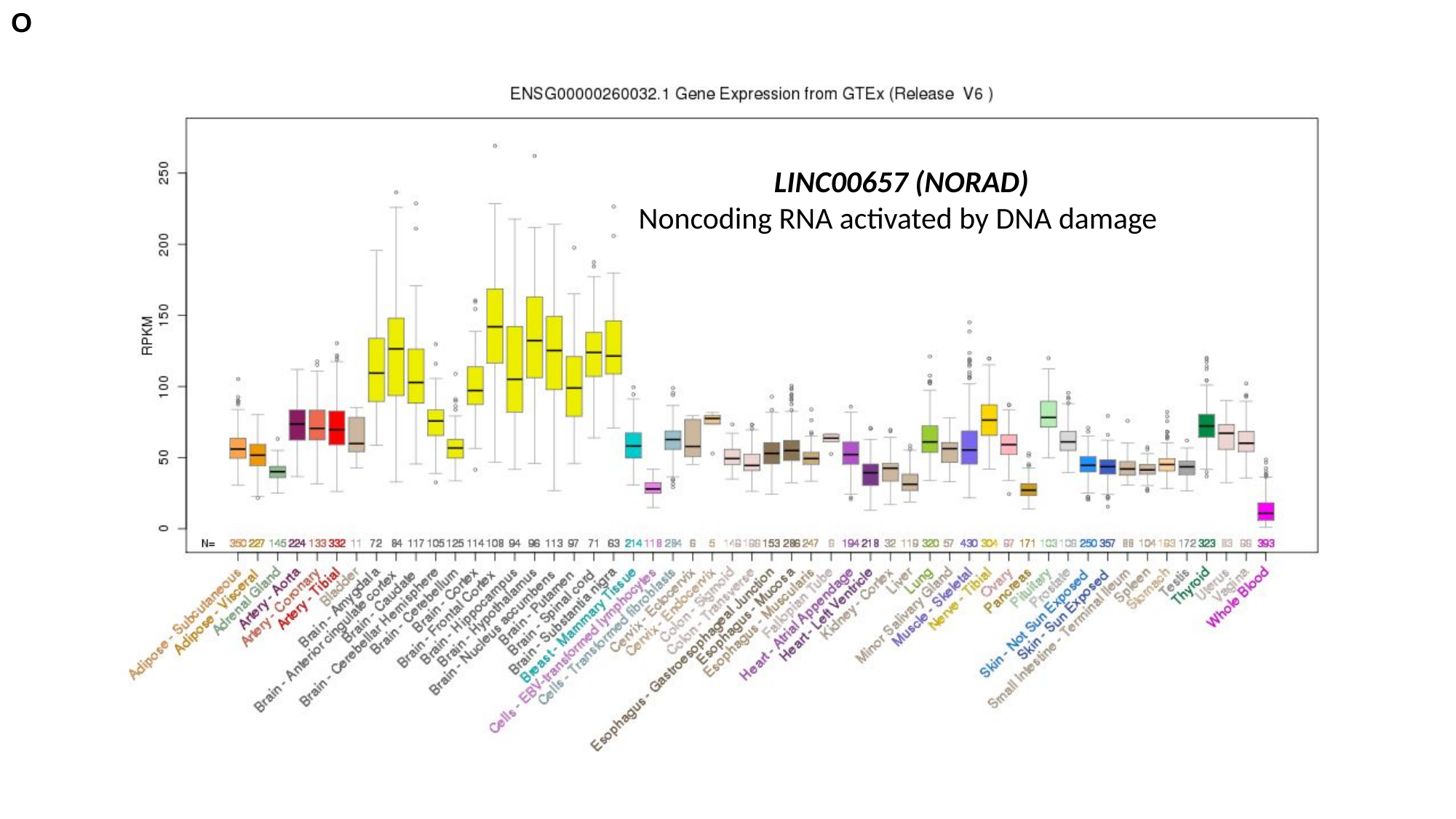

O
LINC00657 (NORAD)
Noncoding RNA activated by DNA damage

## Slide 17
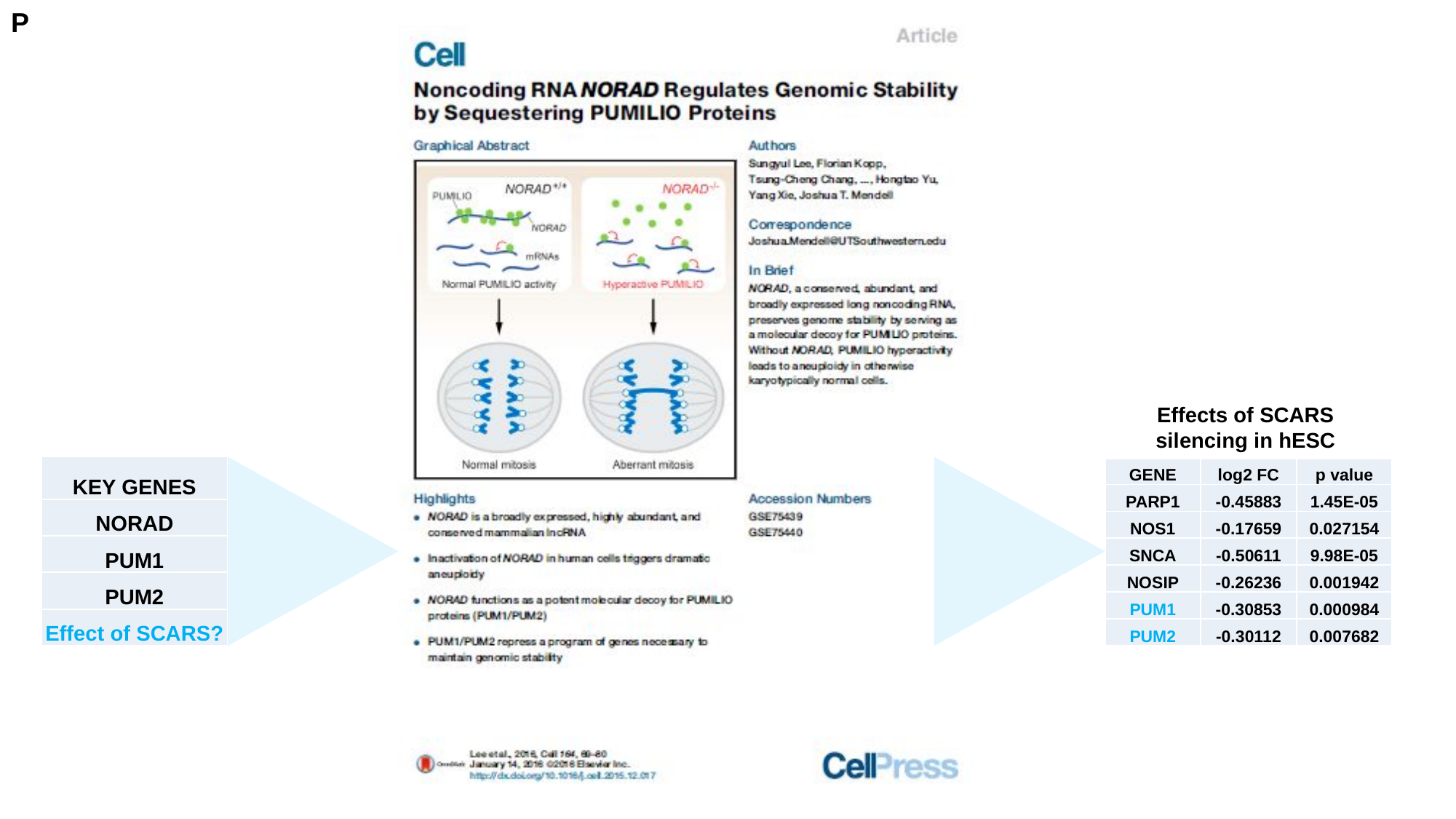

P
Effects of SCARS
silencing in hESC
| KEY GENES |
| --- |
| NORAD |
| PUM1 |
| PUM2 |
| Effect of SCARS? |
| GENE | log2 FC | p value |
| --- | --- | --- |
| PARP1 | -0.45883 | 1.45E-05 |
| NOS1 | -0.17659 | 0.027154 |
| SNCA | -0.50611 | 9.98E-05 |
| NOSIP | -0.26236 | 0.001942 |
| PUM1 | -0.30853 | 0.000984 |
| PUM2 | -0.30112 | 0.007682 |

## Slide 18
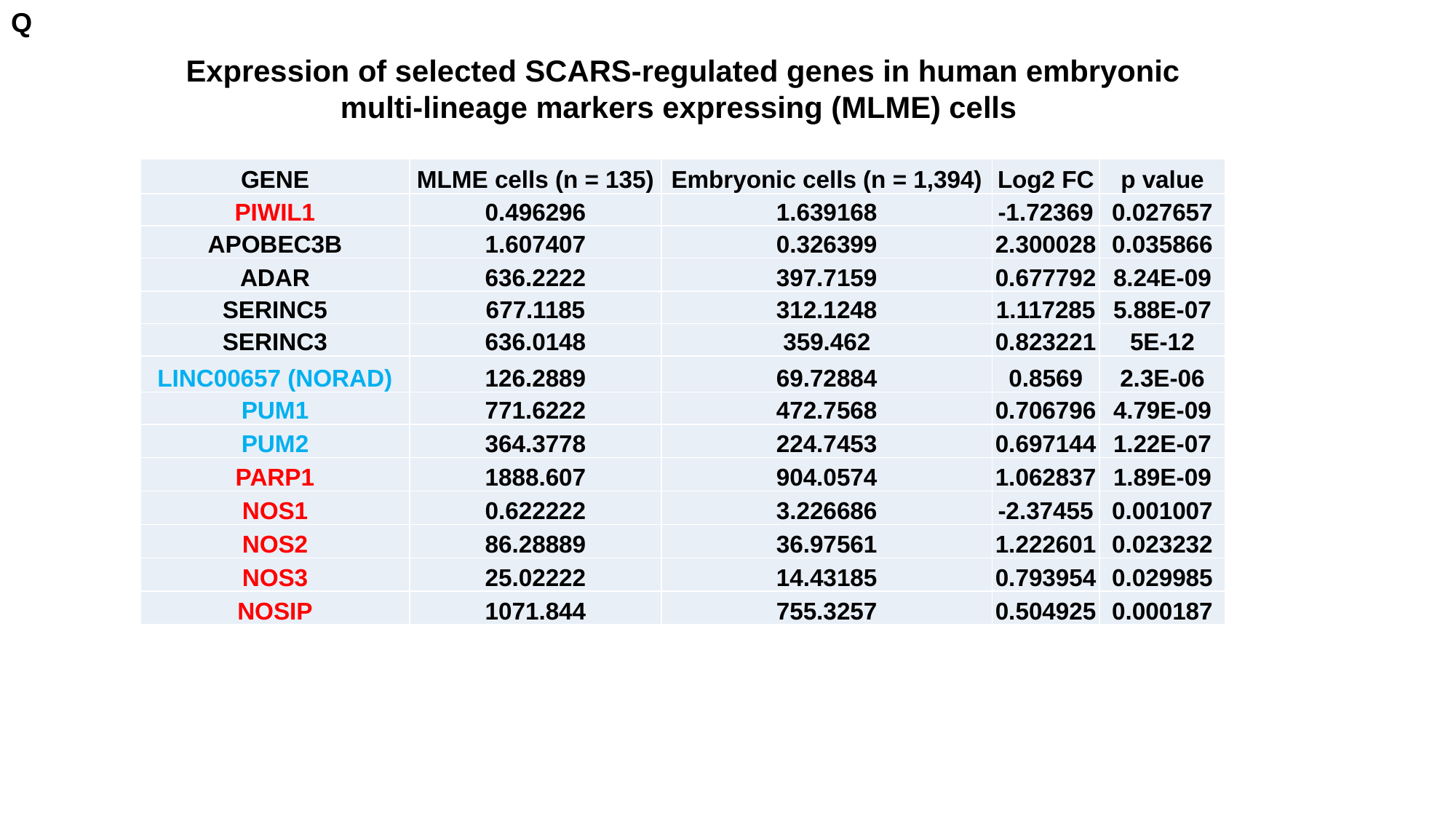

Q
Expression of selected SCARS-regulated genes in human embryonic multi-lineage markers expressing (MLME) cells
| GENE | MLME cells (n = 135) | Embryonic cells (n = 1,394) | Log2 FC | p value |
| --- | --- | --- | --- | --- |
| PIWIL1 | 0.496296 | 1.639168 | -1.72369 | 0.027657 |
| APOBEC3B | 1.607407 | 0.326399 | 2.300028 | 0.035866 |
| ADAR | 636.2222 | 397.7159 | 0.677792 | 8.24E-09 |
| SERINC5 | 677.1185 | 312.1248 | 1.117285 | 5.88E-07 |
| SERINC3 | 636.0148 | 359.462 | 0.823221 | 5E-12 |
| LINC00657 (NORAD) | 126.2889 | 69.72884 | 0.8569 | 2.3E-06 |
| PUM1 | 771.6222 | 472.7568 | 0.706796 | 4.79E-09 |
| PUM2 | 364.3778 | 224.7453 | 0.697144 | 1.22E-07 |
| PARP1 | 1888.607 | 904.0574 | 1.062837 | 1.89E-09 |
| NOS1 | 0.622222 | 3.226686 | -2.37455 | 0.001007 |
| NOS2 | 86.28889 | 36.97561 | 1.222601 | 0.023232 |
| NOS3 | 25.02222 | 14.43185 | 0.793954 | 0.029985 |
| NOSIP | 1071.844 | 755.3257 | 0.504925 | 0.000187 |
