# Supplementary material for: Genomics-Guided Drawing of Molecular and Pathophysiological Components of Malignant Regulatory Signatures Reveals a Pivotal Role in Human Diseases of Stem Cell-Associated Retroviral Sequences and Functionally-Active hESC Enhancers
Source: Front Oncol. 2021 Mar 31;11:638363. doi: 10.3389/fonc.2021.638363 (PMC8044830; doi:10.3389/fonc.2021.638363)
Supplement: Supplementary file 1 [file Presentation_1.zip › Supplemental Methods.docx]

**Supplemental Methods**

**Methods**

**Data source and analytical protocols**

*Candidate human-specific regulatory sequences and African Apes-specific retroviral insertions*

A total of 94,806 candidate HSRS, including 35,074 neuro-regulatory human-specific SNCs, detailed descriptions of which and corresponding references of primary original contributions are reported elsewhere (Glinsky et al., 2015-2019; Kanton et al., 2019). Solely publicly available datasets and resources were used in this contribution. The significance of the differences in the expected and observed numbers of events was calculated using two-tailed Fisher’s exact test. Additional placement enrichment tests were performed for individual classes of HSRS taking into account the size in bp of corresponding genomic regions.

**Data analysis**

**Categories of DNA sequence conservation**

Identification of highly-conserved in primates (pan-primate), primate-specific, and human-specific sequences was performed as previously described (Glinsky, 2015-2019). In brief, all categories were defined by direct and reciprocal mapping using LiftOver. Specifically, the following categories of candidate regulatory sequences were distinguished:

- Highly conserved in primates’ sequences: DNA sequences that have at least 95% of bases remapped during conversion from/to human (Homo sapiens, hg38), chimp (Pan troglodytes, v5), and bonobo (Pan paniscus, v2; in specifically designated instances, Pan paniscus, v1 was utilized for comparisons). Similarly, highly-conserved sequences were defined for hg38 and latest releases of genomes of Gorilla, Orangutan, Gibbon, and Rhesus.
- Primate-specific: DNA sequences that failed to map to the mouse genome (mm10).
- Human-specific: DNA sequences that failed to map at least 10% of bases from human to both chimpanzee and bonobo. All candidate HSRS identified based on the sequence alignments failures to genomes of both chimpanzee and bonobo were subjected to more stringent additional analyses requiring the mapping failures to genomes of Gorilla, Orangutan, Gibbon, and Rhesus. These loci were considered created *de novo* human-specific regulatory sequences (HSRS).

To infer the putative evolutionary origins, each evolutionary classification was defined independently by running the corresponding analyses on all candidate HSRS representing the specific category. For example, human-rodent conversion identify sequences that are absent in the mouse genome based on the sequence identity threshold of 10%). Additional comparisons were performed using the same methodology and exactly as stated in the manuscript text. Human brain regions’ marker genes were identified among genes linked to hsSNCs by analyzing genes significantly up-regulated in specified human brain regions using the Allen Brain Atlas database (brain region-specific records manifesting significantly increased expression at 1.5-fold cut-off were selected for analyses). Genes differentially expressed in human versus chimpanzee adult brains were identified among hsSNCs-linked genes by analyzing genes differentially expressed in eight regions of human versus chimpanzee adult brains (Xu et al., 2018).

**Gene set enrichment and genome-wide proximity placement analyses**

Gene set enrichment analyses were carried-out using the Enrichr bioinformatics platform, which enables the interrogation of nearly 200,000 gene sets from more than 100 gene set libraries. The Enrichr API (January 2018 through January 2020 releases) (Chen et al., 2013; Kuleshov et al., 2016) was used to test genes linked to HSRS of interest for significant enrichment in numerous functional categories. In all tables and plots (unless stated otherwise), in addition to the nominal p values and adjusted p values, the “combined score” calculated by Enrichr is reported, which is a product of the significance estimate and the magnitude of enrichment (combined score *c = log(p) * z*, where *p* is the Fisher’s exact test p-value and *z* is the z-score deviation from the expected rank). When technically feasible, larger sets of genes comprising several thousand entries were analyzed. Regulatory connectivity maps between HSRS and coding genes and additional functional enrichment analyses were performed with the GREAT algorithm (McLean et al., 2010; 2011) at default settings. The reproducibility of the results was validated by implementing two releases of the GREAT algorithm: GREAT version 3.0.0 (2/15/2015 to 08/18/2019) and GREAT version 4.0.4 (08/19/2019). Genome-wide Proximity Placement Analysis (GPPA) of distinct genomic features co-localizing with HSRS was carried out as described previously and originally implemented for human-specific transcription factor binding sites (Glinsky, 2015-2019).

When technically and analytically feasible, different sets of DEGs defined at multiple significance levels of statistical metrics and comprising from dozens to several thousand individual genetic loci were analyzed using differential GSEA to gain insights into biological effects of DEGs and infer potential mechanisms of anticancer activities. This approach was successfully implemented for identification and characterization of human-specific regulatory networks governed by human-specific transcription factor-binding sites (Glinsky, 2015; 2016a; b; c; 2017) and functional enhancer elements (Glinsky and Barakat, 2019; Glinsky, 2018; Glinsky et al., 2018), 13,824 genes associated with 59,732 human-specific regulatory sequences (Glinsky, 2020a), 8,405 genes associated with 35,074 human-specific neuroregulatory single-nucleotide changes (Glinsky, 2020b), as well as human genes and medicinal molecules affecting the susceptibility to SARS-CoV-2 coronavirus (Glinsky, 2020c). Initial GSEA entail interrogations of each specific set of DEGs using 29 distinct genomic databases, including comprehensive pathway enrichment Gene Ontology (GO) analyses followed by in-depth analyses of the selected genomic databases deemed most statistically informative. In all tables and plots (unless stated otherwise), in addition to the nominal p values and adjusted p values, the “combined score” calculated by Enrichr software is reported, which is a product of the significance estimate and the magnitude of enrichment (combined score c = log(p) * z, where p is the Fisher’s exact test p-value and z is the z-score deviation from the expected rank).

**Mammalian Phenotype Ontology and Human Disease Ontology analyses**

To validate and extend findings afforded by the gene set enrichment analyses and to identify all genes linked with human-specific regulatory SNCs that are associated with defined mammalian phenotypes as well as implicated in development of human diseases with one or more mouse models, the additional analyses have been carried out utilizing the Mouse Genome Informatics (MGI) database (<http://www.informatics.jax.org/>).

*Statistical Analyses of the Publicly Available Datasets*

All statistical analyses of the publicly available genomic datasets, including error rate estimates, background and technical noise measurements and filtering, feature peak calling, feature selection, assignments of genomic coordinates to the corresponding builds of the reference human genome, and data visualization, were performed exactly as reported in the original publications and associated references linked to the corresponding data visualization tracks (<http://genome.ucsc.edu/>). Any modifications or new elements of statistical analyses are described in the corresponding sections of the Results. Statistical significance of the Pearson correlation coefficients was determined using GraphPad Prism version 6.00 software. Both nominal and Bonferroni adjusted p values were estimated. The significance of the differences in the numbers of events between the groups was calculated using two-sided Fisher’s exact and Chi-square test, and the significance of the overlap between the events was determined using the hypergeometric distribution test (Tavazoie et al., 1999).
