# Supplementary material for: Genomics-Guided Drawing of Molecular and Pathophysiological Components of Malignant Regulatory Signatures Reveals a Pivotal Role in Human Diseases of Stem Cell-Associated Retroviral Sequences and Functionally-Active hESC Enhancers
Source: Front Oncol. 2021 Mar 31;11:638363. doi: 10.3389/fonc.2021.638363 (PMC8044830; doi:10.3389/fonc.2021.638363)
Supplement: Supplementary file 1 [file Presentation_1.zip › Supplemental Note S1. 8384 genes HSRS_SCARS.pptx]

## Slide 1
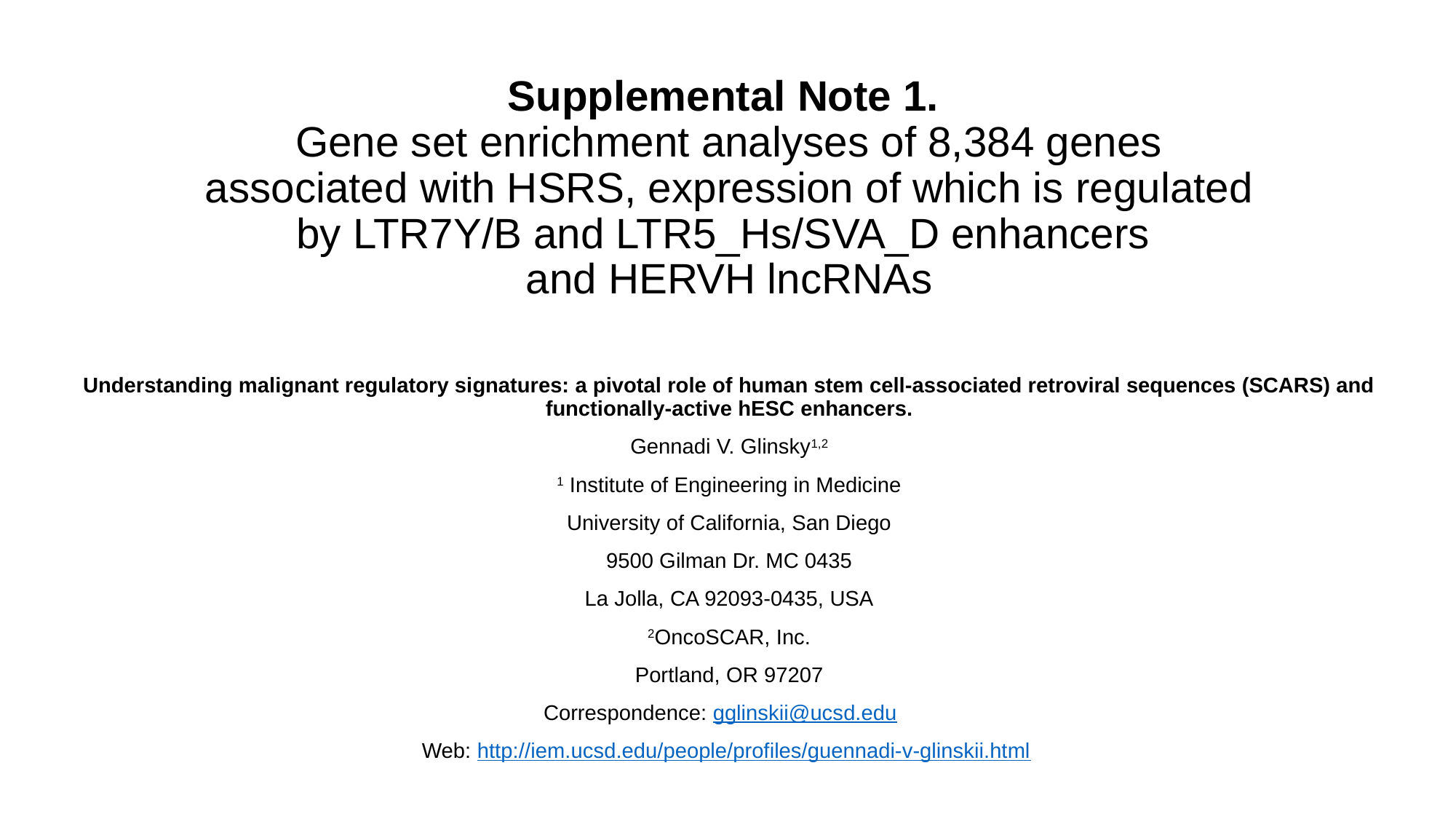

# Supplemental Note 1. Gene set enrichment analyses of 8,384 genes associated with HSRS, expression of which is regulated by LTR7Y/B and LTR5_Hs/SVA_D enhancers and HERVH lncRNAs
Understanding malignant regulatory signatures: a pivotal role of human stem cell-associated retroviral sequences (SCARS) and functionally-active hESC enhancers.
Gennadi V. Glinsky1,2
1 Institute of Engineering in Medicine
University of California, San Diego
9500 Gilman Dr. MC 0435
La Jolla, CA 92093-0435, USA
2OncoSCAR, Inc.
Portland, OR 97207
Correspondence: gglinskii@ucsd.edu
Web: http://iem.ucsd.edu/people/profiles/guennadi-v-glinskii.html

## Slide 2
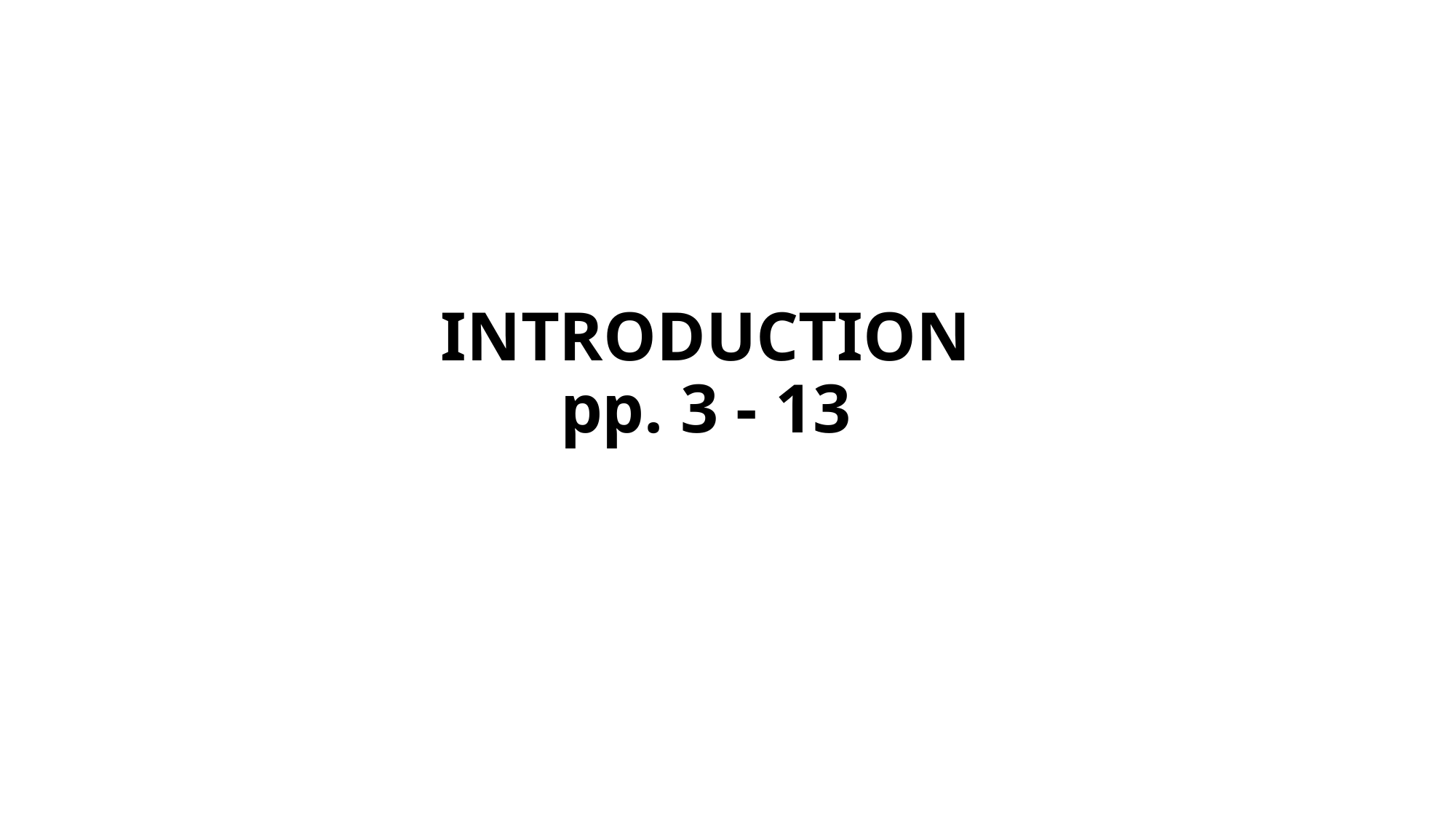

# INTRODUCTION pp. 3 - 13

## Slide 3
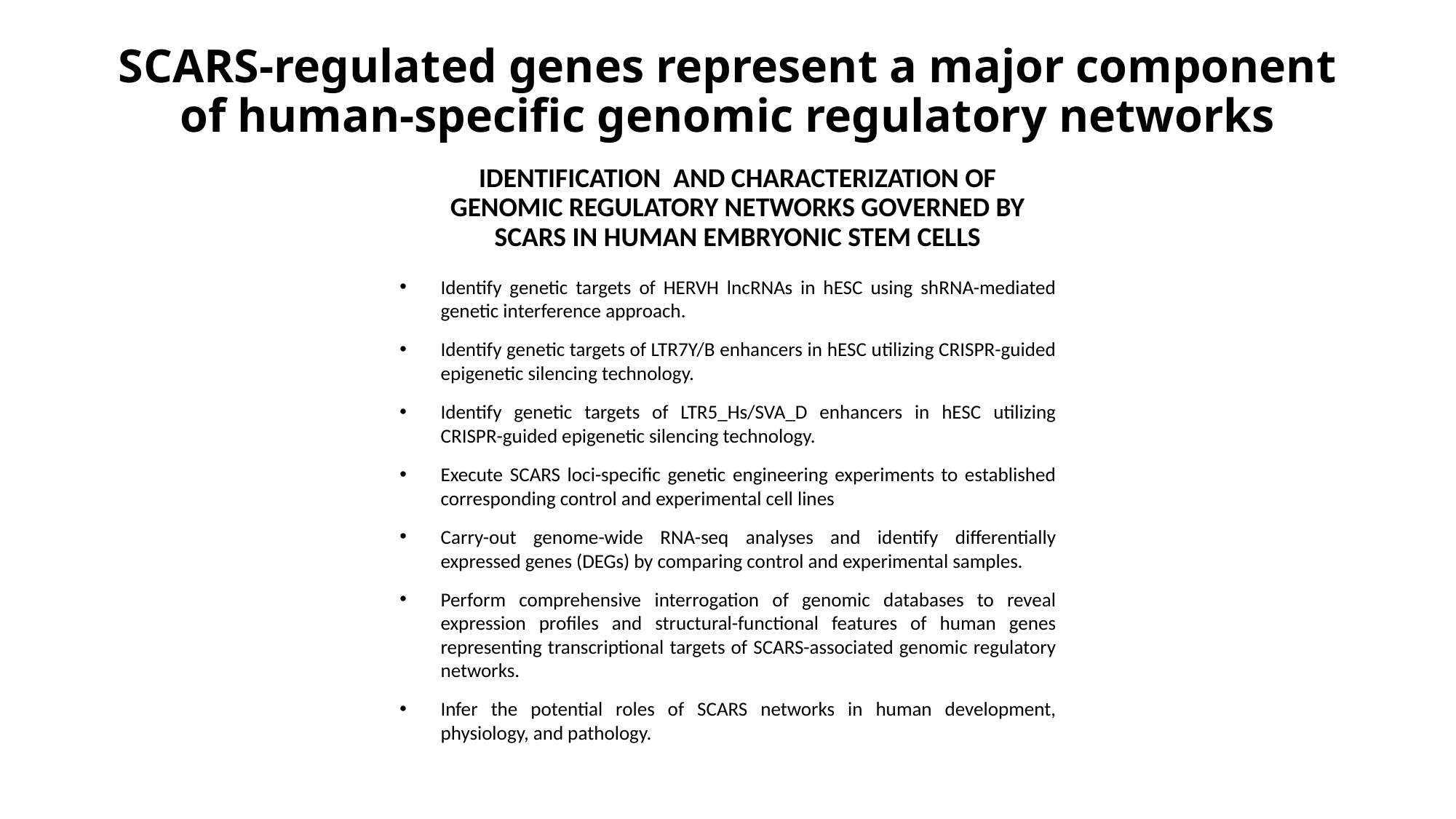

# SCARS-regulated genes represent a major component of human-specific genomic regulatory networks
IDENTIFICATION AND CHARACTERIZATION OF GENOMIC REGULATORY NETWORKS GOVERNED BY SCARS IN HUMAN EMBRYONIC STEM CELLS
Identify genetic targets of HERVH lncRNAs in hESC using shRNA-mediated genetic interference approach.
Identify genetic targets of LTR7Y/B enhancers in hESC utilizing CRISPR-guided epigenetic silencing technology.
Identify genetic targets of LTR5_Hs/SVA_D enhancers in hESC utilizing CRISPR-guided epigenetic silencing technology.
Execute SCARS loci-specific genetic engineering experiments to established corresponding control and experimental cell lines
Carry-out genome-wide RNA-seq analyses and identify differentially expressed genes (DEGs) by comparing control and experimental samples.
Perform comprehensive interrogation of genomic databases to reveal expression profiles and structural-functional features of human genes representing transcriptional targets of SCARS-associated genomic regulatory networks.
Infer the potential roles of SCARS networks in human development, physiology, and pathology.

## Slide 4
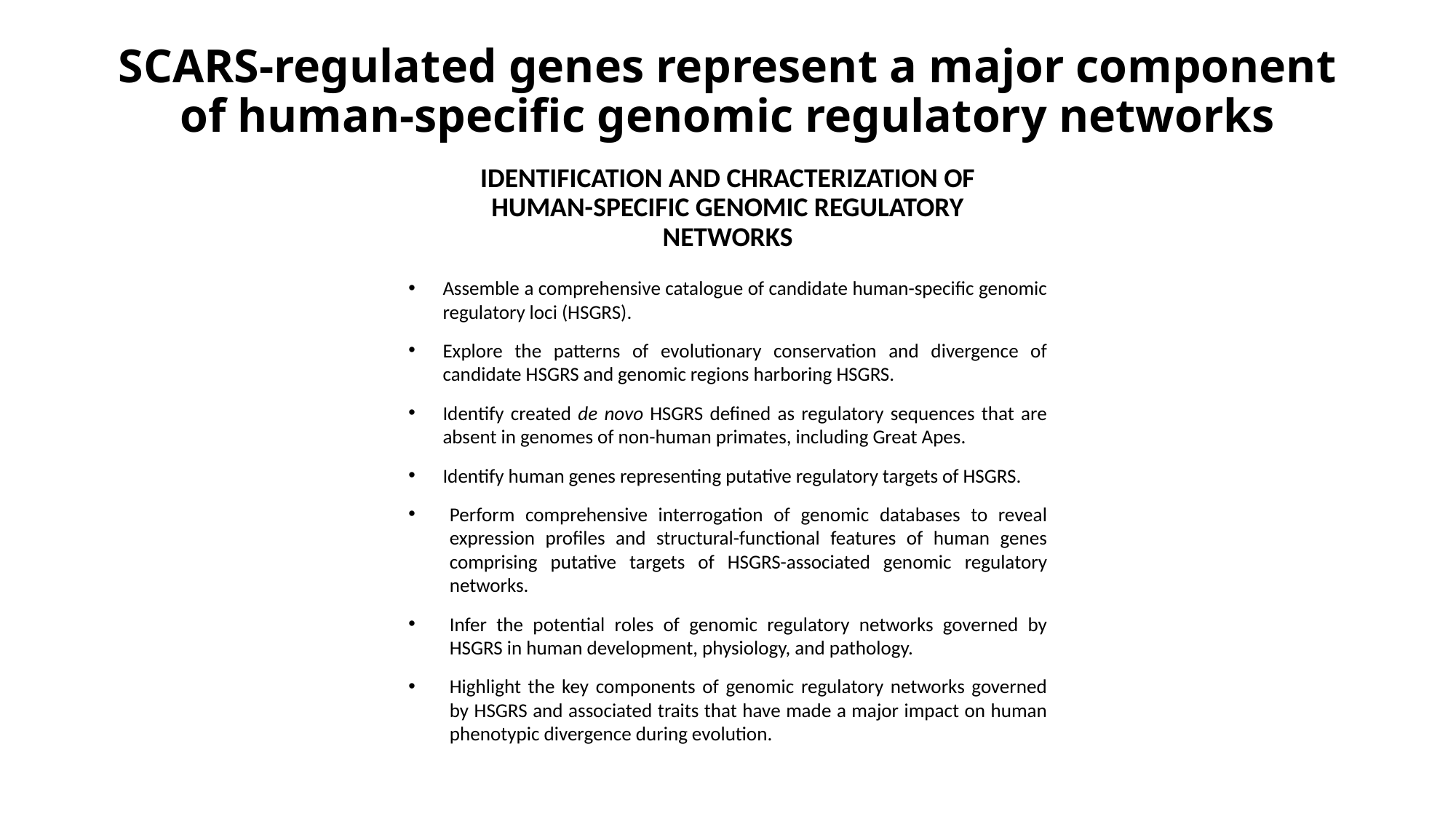

# SCARS-regulated genes represent a major component of human-specific genomic regulatory networks
IDENTIFICATION AND CHRACTERIZATION OF HUMAN-SPECIFIC GENOMIC REGULATORY NETWORKS
Assemble a comprehensive catalogue of candidate human-specific genomic regulatory loci (HSGRS).
Explore the patterns of evolutionary conservation and divergence of candidate HSGRS and genomic regions harboring HSGRS.
Identify created de novo HSGRS defined as regulatory sequences that are absent in genomes of non-human primates, including Great Apes.
Identify human genes representing putative regulatory targets of HSGRS.
Perform comprehensive interrogation of genomic databases to reveal expression profiles and structural-functional features of human genes comprising putative targets of HSGRS-associated genomic regulatory networks.
Infer the potential roles of genomic regulatory networks governed by HSGRS in human development, physiology, and pathology.
Highlight the key components of genomic regulatory networks governed by HSGRS and associated traits that have made a major impact on human phenotypic divergence during evolution.

## Slide 5
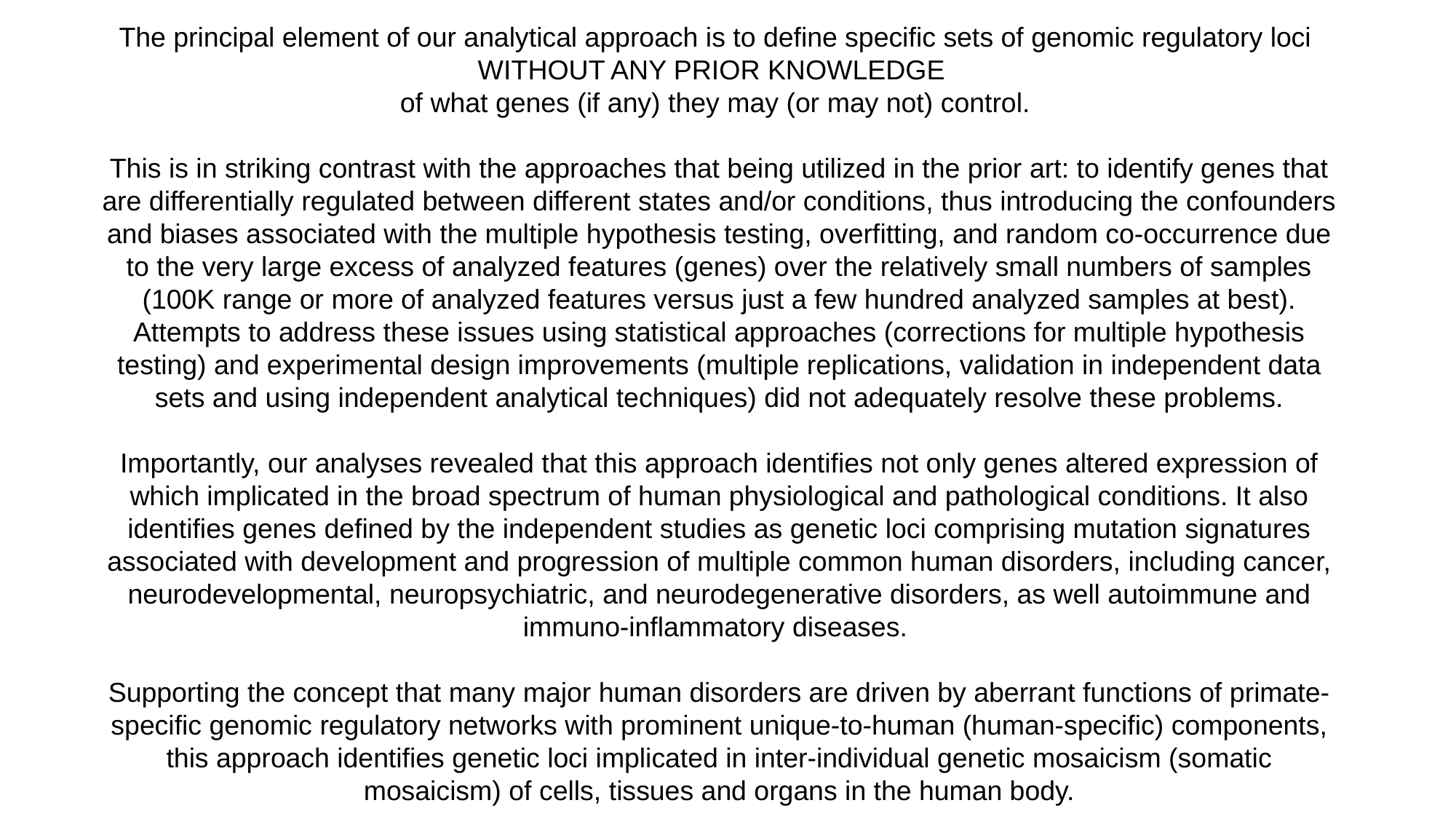

The principal element of our analytical approach is to define specific sets of genomic regulatory loci
WITHOUT ANY PRIOR KNOWLEDGE
of what genes (if any) they may (or may not) control.
This is in striking contrast with the approaches that being utilized in the prior art: to identify genes that are differentially regulated between different states and/or conditions, thus introducing the confounders and biases associated with the multiple hypothesis testing, overfitting, and random co-occurrence due to the very large excess of analyzed features (genes) over the relatively small numbers of samples (100K range or more of analyzed features versus just a few hundred analyzed samples at best). Attempts to address these issues using statistical approaches (corrections for multiple hypothesis testing) and experimental design improvements (multiple replications, validation in independent data sets and using independent analytical techniques) did not adequately resolve these problems.
Importantly, our analyses revealed that this approach identifies not only genes altered expression of which implicated in the broad spectrum of human physiological and pathological conditions. It also identifies genes defined by the independent studies as genetic loci comprising mutation signatures associated with development and progression of multiple common human disorders, including cancer, neurodevelopmental, neuropsychiatric, and neurodegenerative disorders, as well autoimmune and immuno-inflammatory diseases.
Supporting the concept that many major human disorders are driven by aberrant functions of primate-specific genomic regulatory networks with prominent unique-to-human (human-specific) components, this approach identifies genetic loci implicated in inter-individual genetic mosaicism (somatic mosaicism) of cells, tissues and organs in the human body.

## Slide 6
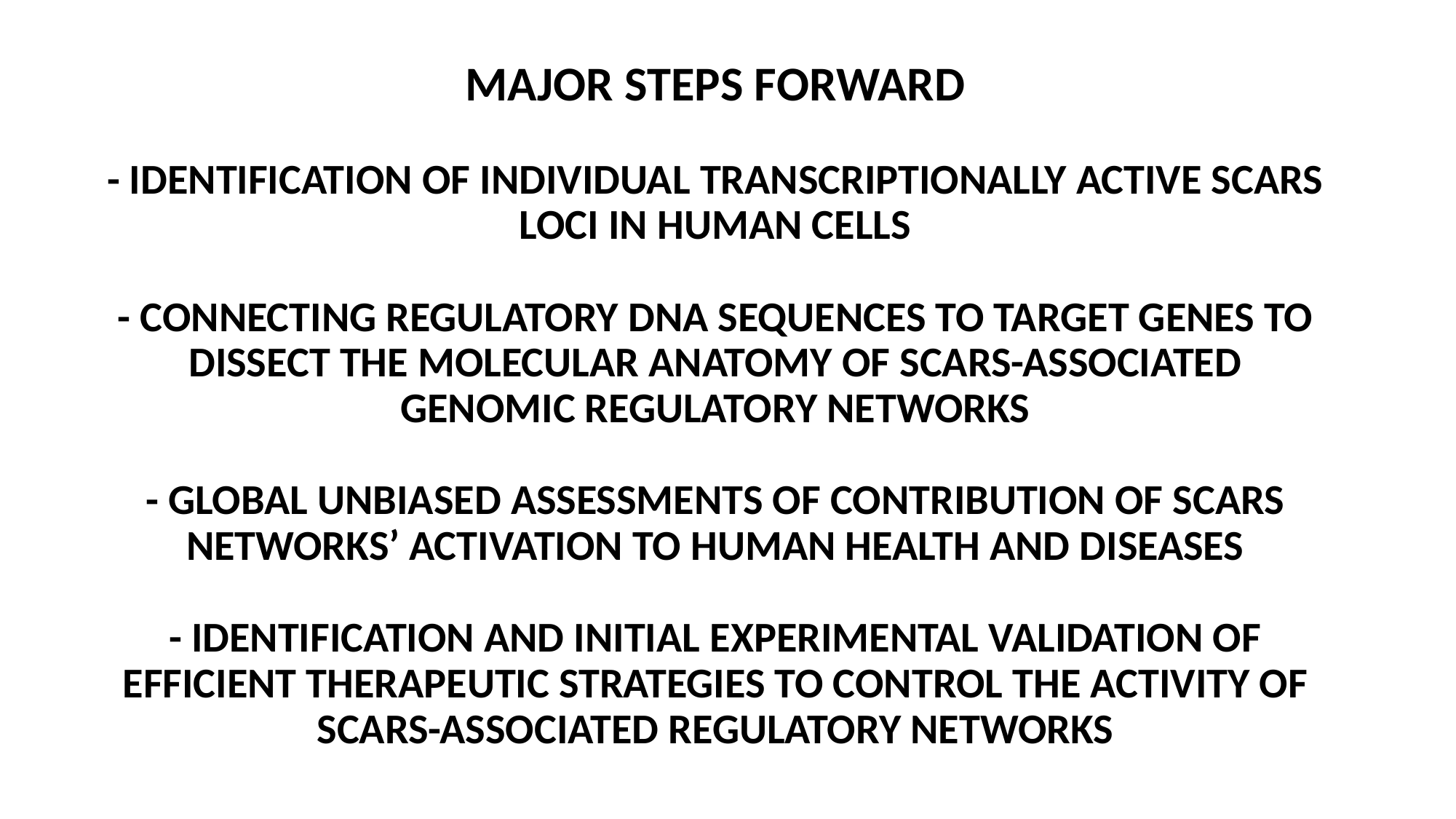

# MAJOR STEPS FORWARD - IDENTIFICATION OF INDIVIDUAL TRANSCRIPTIONALLY ACTIVE SCARS LOCI IN HUMAN CELLS - CONNECTING REGULATORY DNA SEQUENCES TO TARGET GENES TO DISSECT THE MOLECULAR ANATOMY OF SCARS-ASSOCIATED GENOMIC REGULATORY NETWORKS - GLOBAL UNBIASED ASSESSMENTS OF CONTRIBUTION OF SCARS NETWORKS’ ACTIVATION TO HUMAN HEALTH AND DISEASES - IDENTIFICATION AND INITIAL EXPERIMENTAL VALIDATION OF EFFICIENT THERAPEUTIC STRATEGIES TO CONTROL THE ACTIVITY OF SCARS-ASSOCIATED REGULATORY NETWORKS

## Slide 7
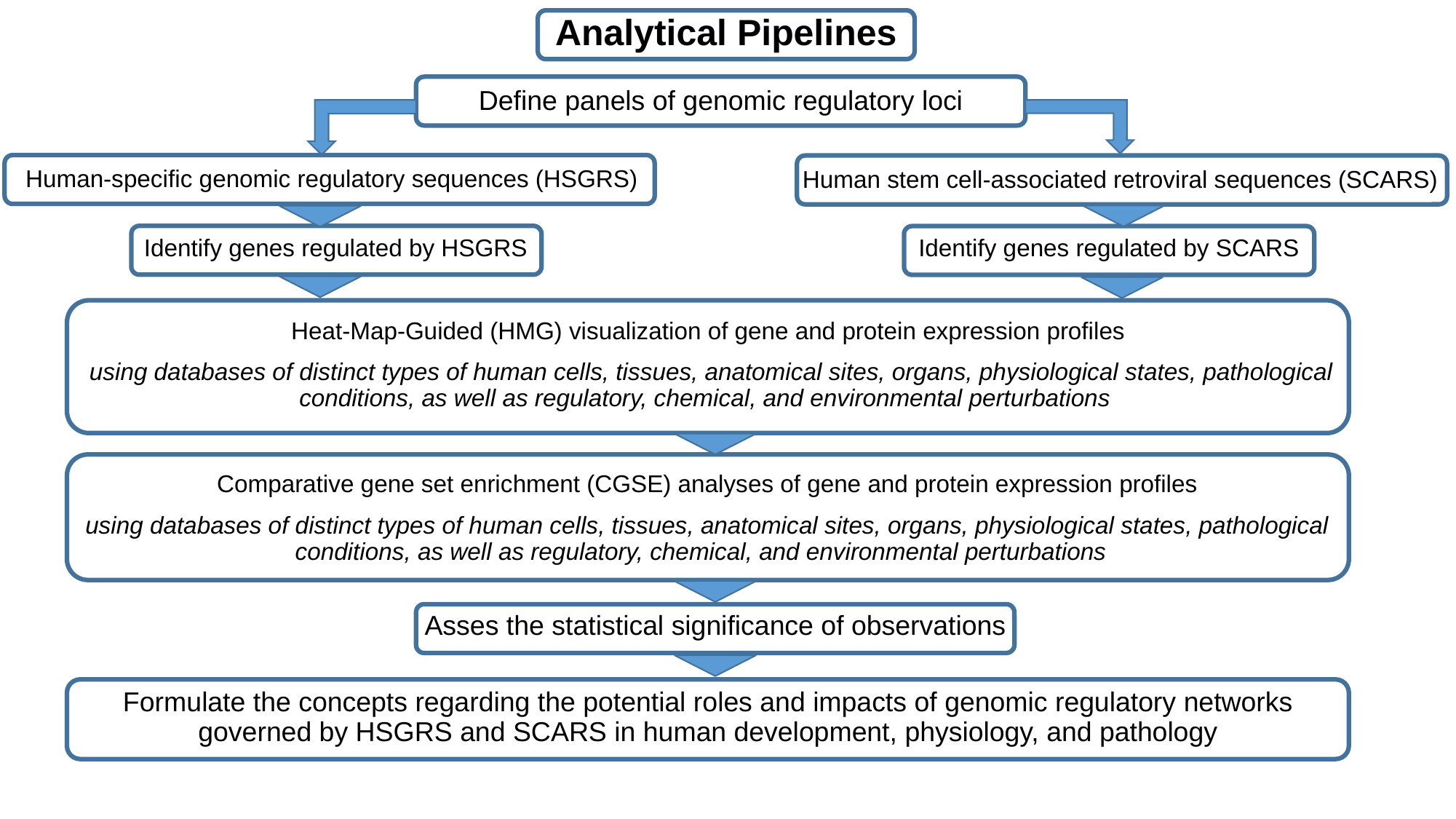

# Analytical Pipelines
Define panels of genomic regulatory loci
Human-specific genomic regulatory sequences (HSGRS)
Human stem cell-associated retroviral sequences (SCARS)
Identify genes regulated by HSGRS
Identify genes regulated by SCARS
Heat-Map-Guided (HMG) visualization of gene and protein expression profiles
using databases of distinct types of human cells, tissues, anatomical sites, organs, physiological states, pathological conditions, as well as regulatory, chemical, and environmental perturbations
Comparative gene set enrichment (CGSE) analyses of gene and protein expression profiles
using databases of distinct types of human cells, tissues, anatomical sites, organs, physiological states, pathological conditions, as well as regulatory, chemical, and environmental perturbations
Asses the statistical significance of observations
Formulate the concepts regarding the potential roles and impacts of genomic regulatory networks governed by HSGRS and SCARS in human development, physiology, and pathology

## Slide 8
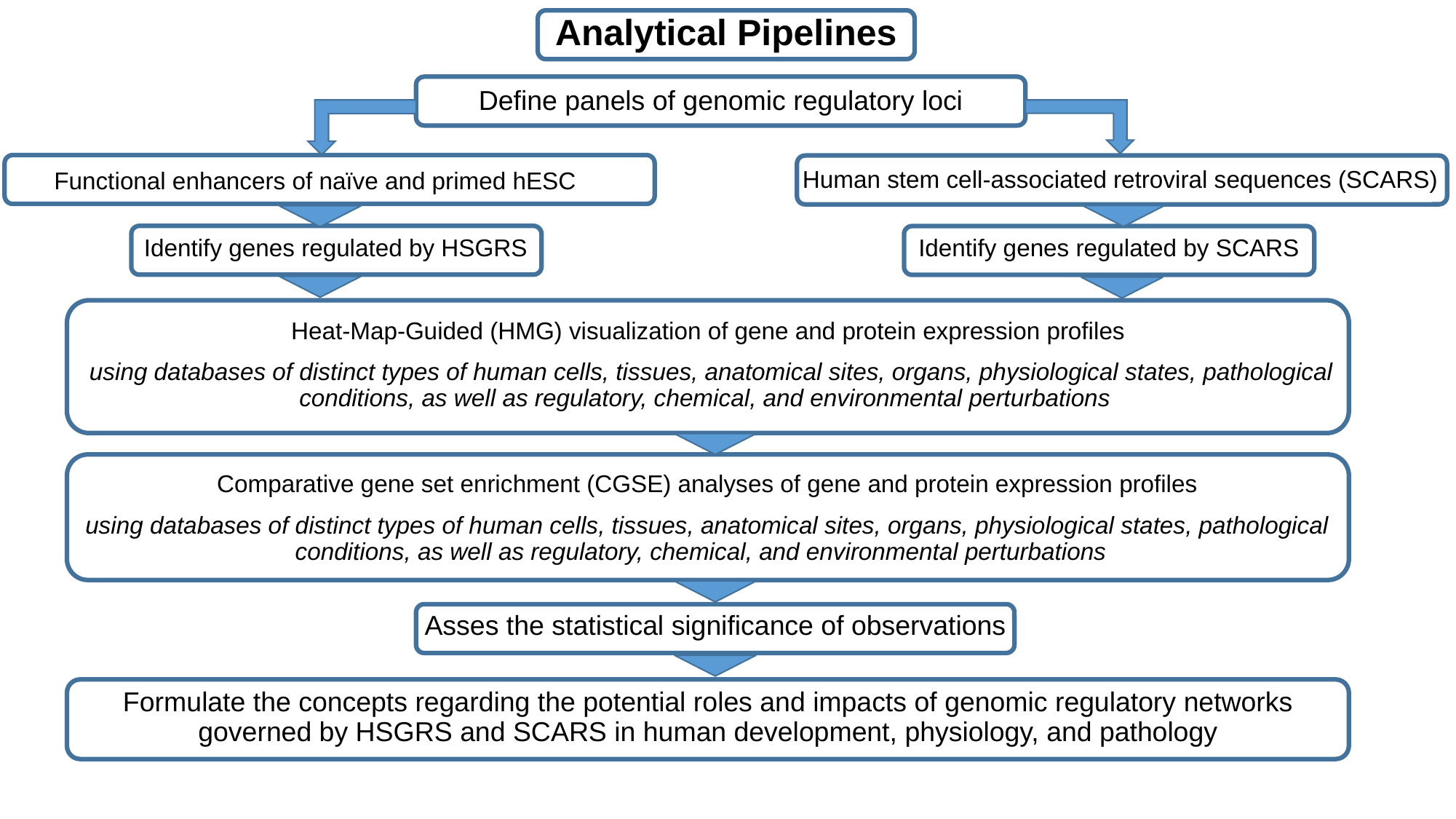

# Analytical Pipelines
Define panels of genomic regulatory loci
Human stem cell-associated retroviral sequences (SCARS)
Functional enhancers of naïve and primed hESC
Identify genes regulated by HSGRS
Identify genes regulated by SCARS
Heat-Map-Guided (HMG) visualization of gene and protein expression profiles
using databases of distinct types of human cells, tissues, anatomical sites, organs, physiological states, pathological conditions, as well as regulatory, chemical, and environmental perturbations
Comparative gene set enrichment (CGSE) analyses of gene and protein expression profiles
using databases of distinct types of human cells, tissues, anatomical sites, organs, physiological states, pathological conditions, as well as regulatory, chemical, and environmental perturbations
Asses the statistical significance of observations
Formulate the concepts regarding the potential roles and impacts of genomic regulatory networks governed by HSGRS and SCARS in human development, physiology, and pathology

## Slide 9
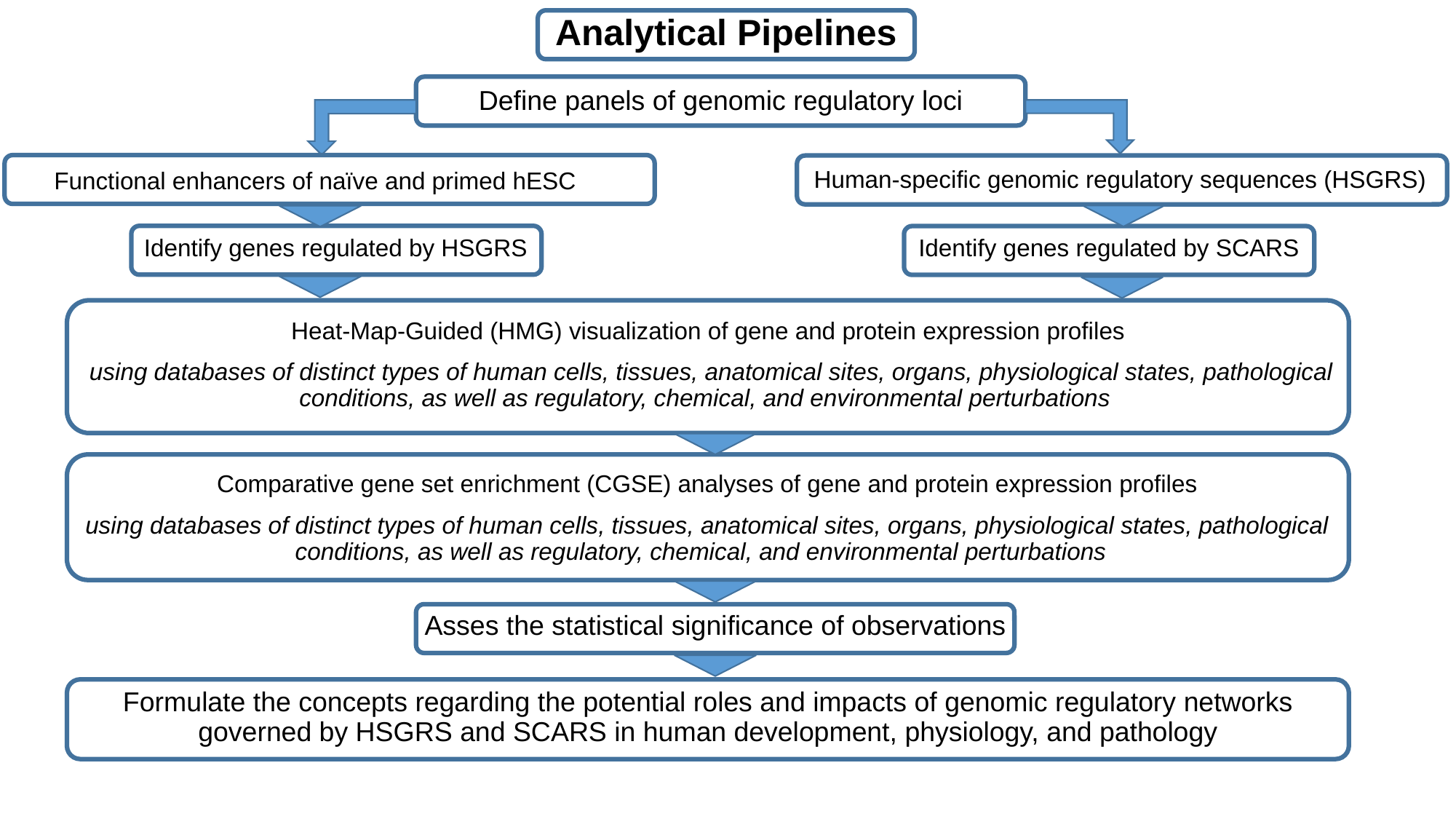

# Analytical Pipelines
Define panels of genomic regulatory loci
Human-specific genomic regulatory sequences (HSGRS)
Functional enhancers of naïve and primed hESC
Identify genes regulated by HSGRS
Identify genes regulated by SCARS
Heat-Map-Guided (HMG) visualization of gene and protein expression profiles
using databases of distinct types of human cells, tissues, anatomical sites, organs, physiological states, pathological conditions, as well as regulatory, chemical, and environmental perturbations
Comparative gene set enrichment (CGSE) analyses of gene and protein expression profiles
using databases of distinct types of human cells, tissues, anatomical sites, organs, physiological states, pathological conditions, as well as regulatory, chemical, and environmental perturbations
Asses the statistical significance of observations
Formulate the concepts regarding the potential roles and impacts of genomic regulatory networks governed by HSGRS and SCARS in human development, physiology, and pathology

## Slide 10
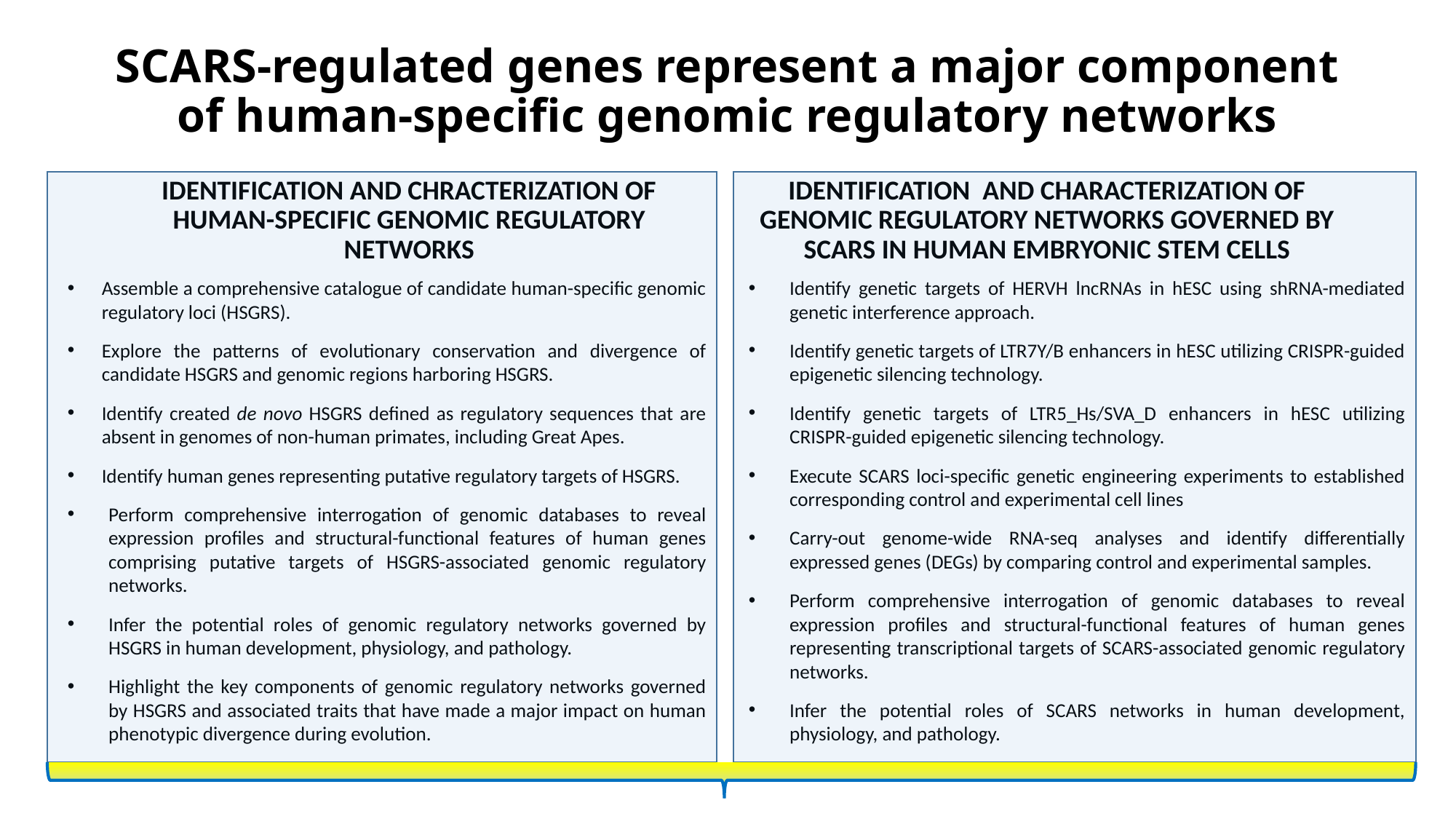

# SCARS-regulated genes represent a major component of human-specific genomic regulatory networks
IDENTIFICATION AND CHRACTERIZATION OF HUMAN-SPECIFIC GENOMIC REGULATORY NETWORKS
IDENTIFICATION AND CHARACTERIZATION OF GENOMIC REGULATORY NETWORKS GOVERNED BY SCARS IN HUMAN EMBRYONIC STEM CELLS
Assemble a comprehensive catalogue of candidate human-specific genomic regulatory loci (HSGRS).
Explore the patterns of evolutionary conservation and divergence of candidate HSGRS and genomic regions harboring HSGRS.
Identify created de novo HSGRS defined as regulatory sequences that are absent in genomes of non-human primates, including Great Apes.
Identify human genes representing putative regulatory targets of HSGRS.
Perform comprehensive interrogation of genomic databases to reveal expression profiles and structural-functional features of human genes comprising putative targets of HSGRS-associated genomic regulatory networks.
Infer the potential roles of genomic regulatory networks governed by HSGRS in human development, physiology, and pathology.
Highlight the key components of genomic regulatory networks governed by HSGRS and associated traits that have made a major impact on human phenotypic divergence during evolution.
Identify genetic targets of HERVH lncRNAs in hESC using shRNA-mediated genetic interference approach.
Identify genetic targets of LTR7Y/B enhancers in hESC utilizing CRISPR-guided epigenetic silencing technology.
Identify genetic targets of LTR5_Hs/SVA_D enhancers in hESC utilizing CRISPR-guided epigenetic silencing technology.
Execute SCARS loci-specific genetic engineering experiments to established corresponding control and experimental cell lines
Carry-out genome-wide RNA-seq analyses and identify differentially expressed genes (DEGs) by comparing control and experimental samples.
Perform comprehensive interrogation of genomic databases to reveal expression profiles and structural-functional features of human genes representing transcriptional targets of SCARS-associated genomic regulatory networks.
Infer the potential roles of SCARS networks in human development, physiology, and pathology.

## Slide 11
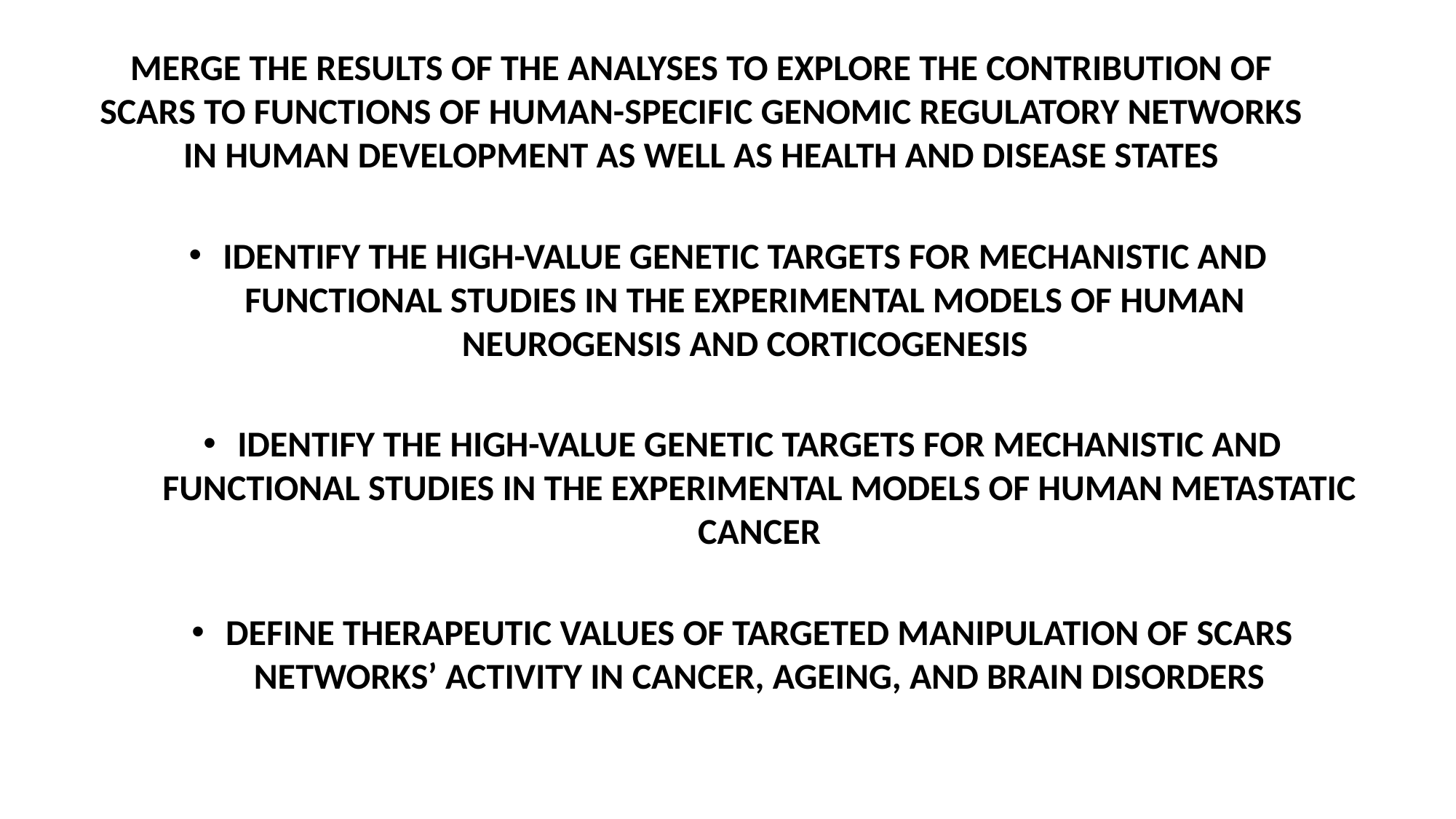

MERGE THE RESULTS OF THE ANALYSES TO EXPLORE THE CONTRIBUTION OF SCARS TO FUNCTIONS OF HUMAN-SPECIFIC GENOMIC REGULATORY NETWORKS IN HUMAN DEVELOPMENT AS WELL AS HEALTH AND DISEASE STATES
IDENTIFY THE HIGH-VALUE GENETIC TARGETS FOR MECHANISTIC AND FUNCTIONAL STUDIES IN THE EXPERIMENTAL MODELS OF HUMAN NEUROGENSIS AND CORTICOGENESIS
IDENTIFY THE HIGH-VALUE GENETIC TARGETS FOR MECHANISTIC AND FUNCTIONAL STUDIES IN THE EXPERIMENTAL MODELS OF HUMAN METASTATIC CANCER
DEFINE THERAPEUTIC VALUES OF TARGETED MANIPULATION OF SCARS NETWORKS’ ACTIVITY IN CANCER, AGEING, AND BRAIN DISORDERS

## Slide 12
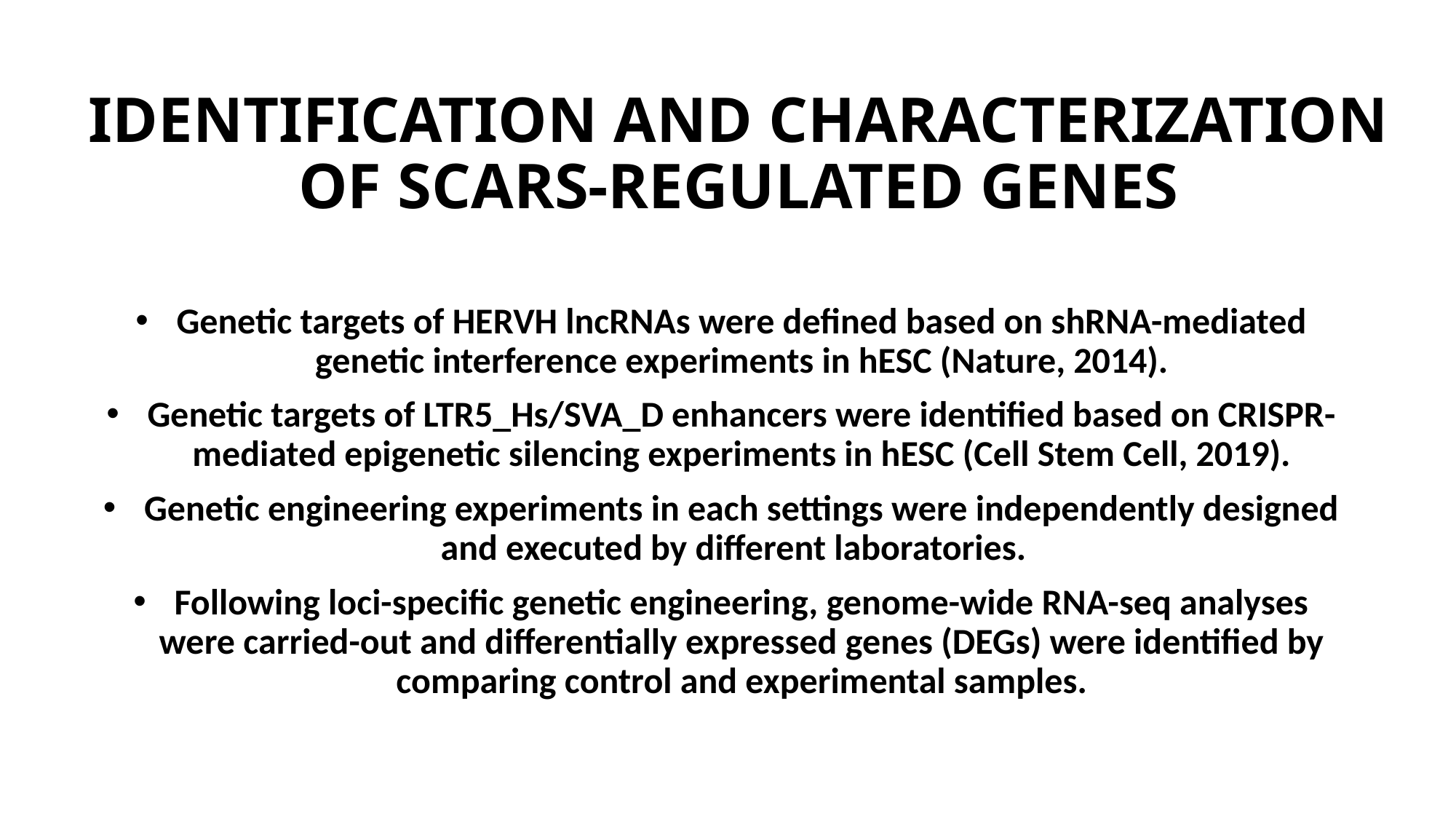

# IDENTIFICATION AND CHARACTERIZATION OF SCARS-REGULATED GENES
Genetic targets of HERVH lncRNAs were defined based on shRNA-mediated genetic interference experiments in hESC (Nature, 2014).
Genetic targets of LTR5_Hs/SVA_D enhancers were identified based on CRISPR-mediated epigenetic silencing experiments in hESC (Cell Stem Cell, 2019).
Genetic engineering experiments in each settings were independently designed and executed by different laboratories.
Following loci-specific genetic engineering, genome-wide RNA-seq analyses were carried-out and differentially expressed genes (DEGs) were identified by comparing control and experimental samples.

## Slide 13
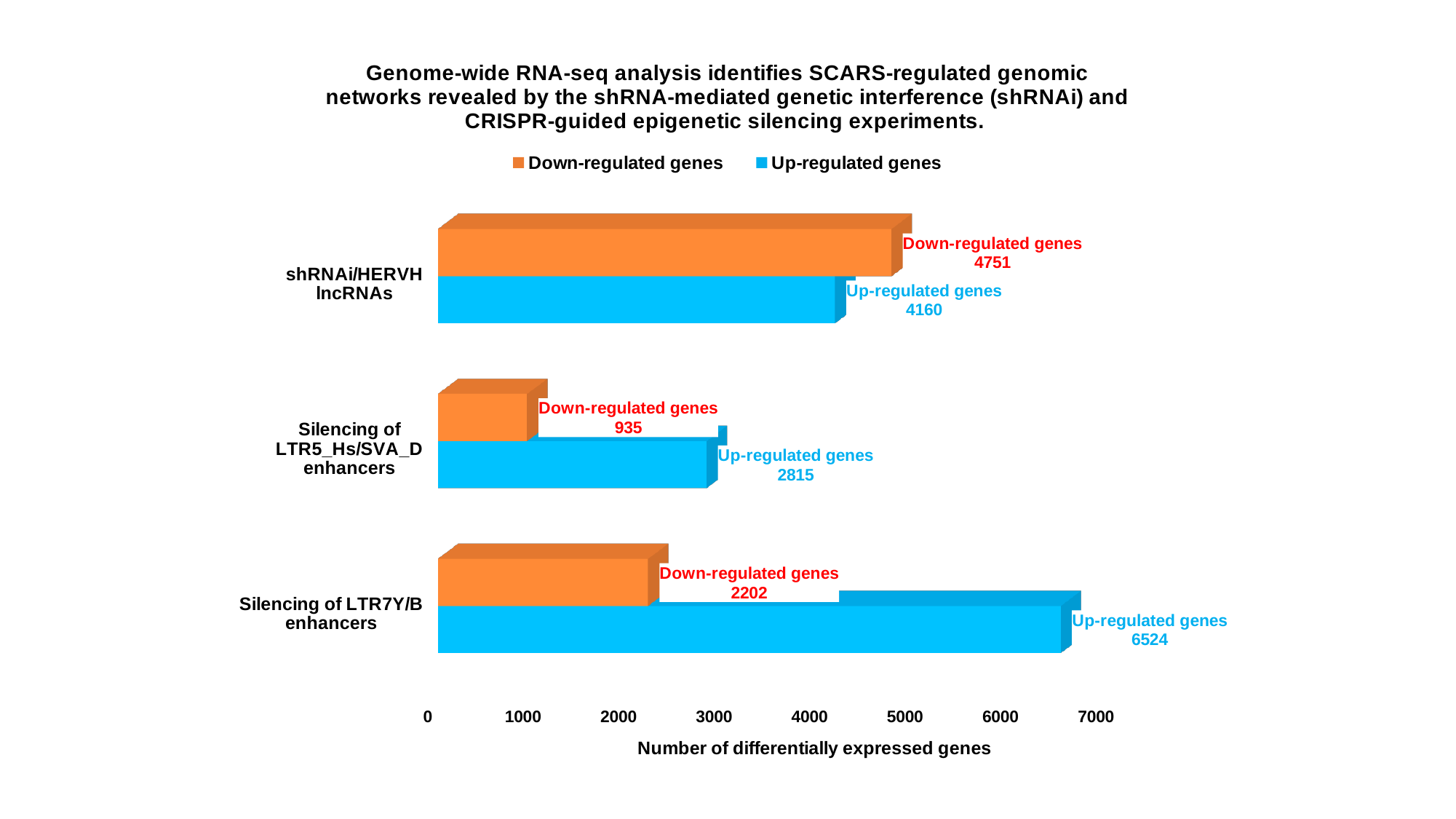

[unsupported chart]

## Slide 14
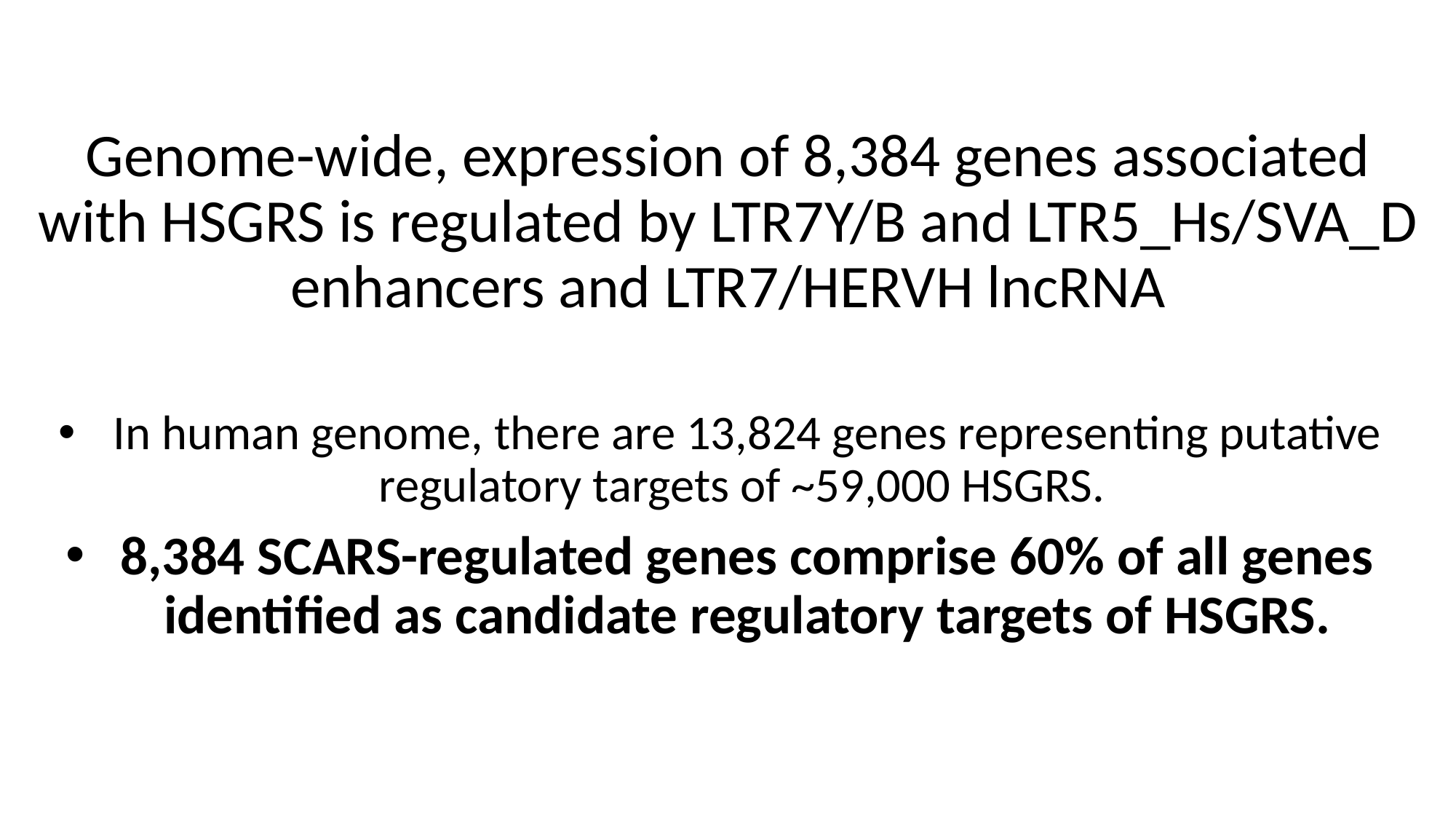

# Genome-wide, expression of 8,384 genes associated with HSGRS is regulated by LTR7Y/B and LTR5_Hs/SVA_D enhancers and LTR7/HERVH lncRNA
In human genome, there are 13,824 genes representing putative regulatory targets of ~59,000 HSGRS.
8,384 SCARS-regulated genes comprise 60% of all genes identified as candidate regulatory targets of HSGRS.

## Slide 15
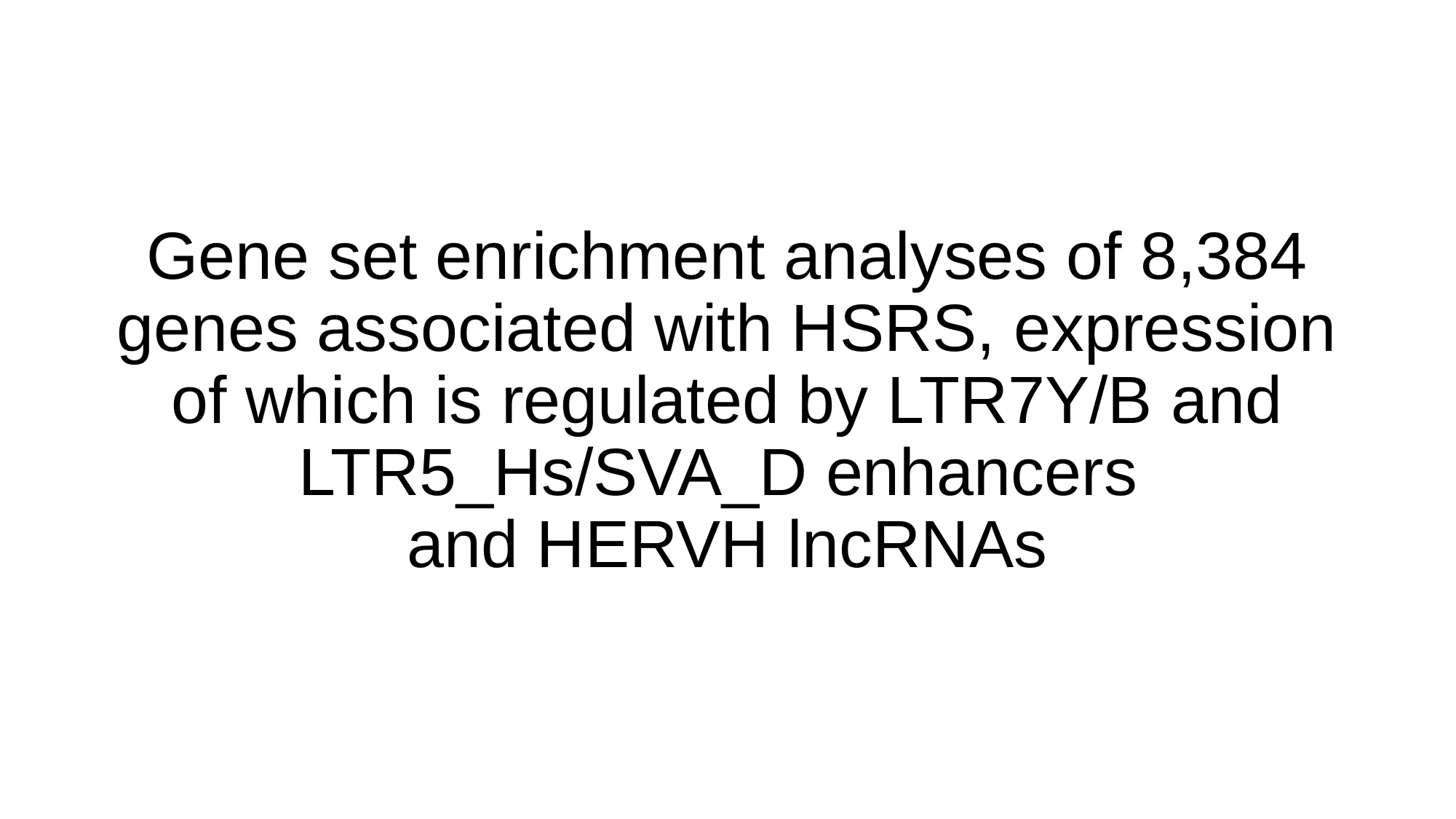

# Gene set enrichment analyses of 8,384 genes associated with HSRS, expression of which is regulated by LTR7Y/B and LTR5_Hs/SVA_D enhancers and HERVH lncRNAs

## Slide 16
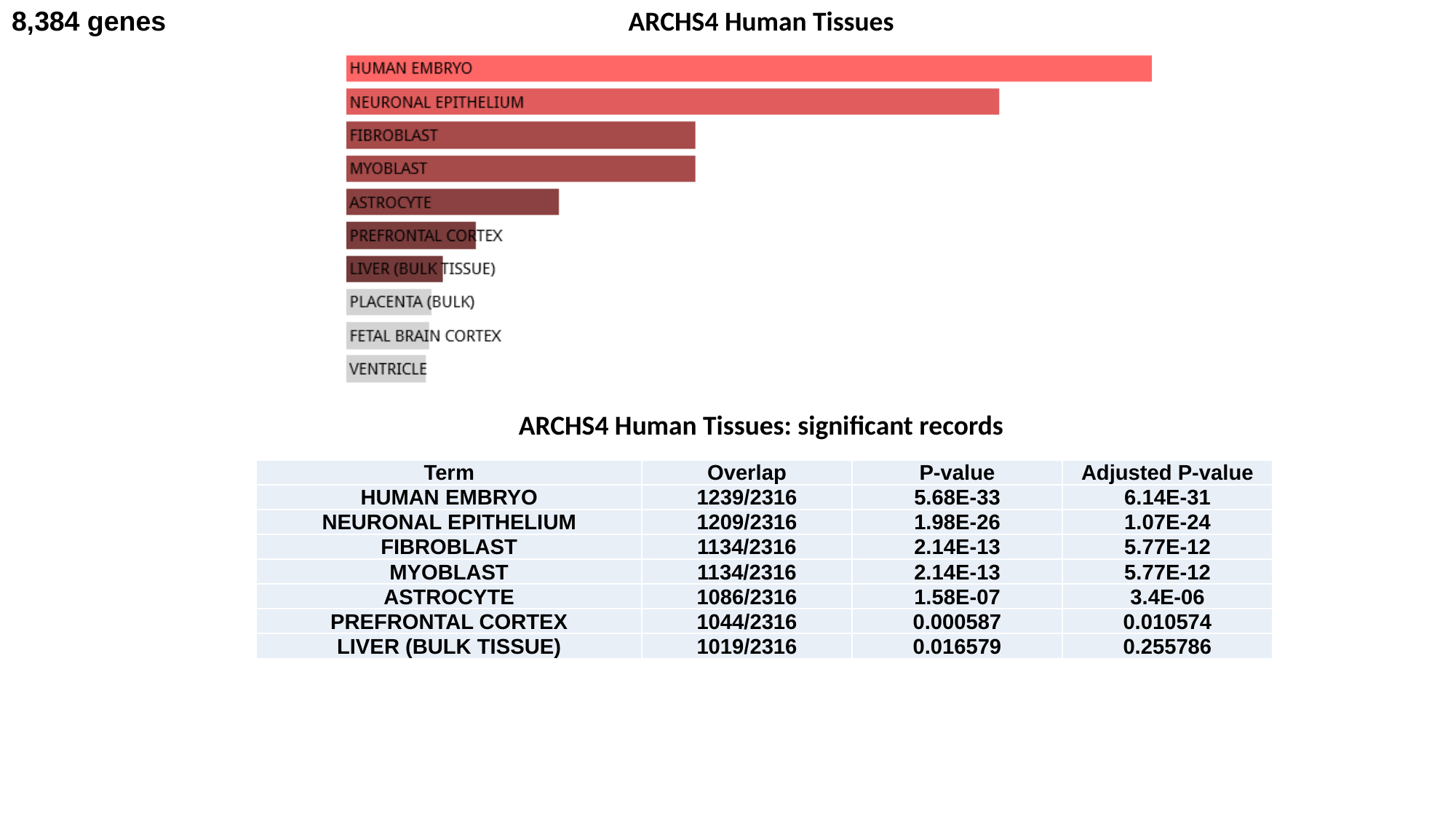

8,384 genes
ARCHS4 Human Tissues
ARCHS4 Human Tissues: significant records
| Term | Overlap | P-value | Adjusted P-value |
| --- | --- | --- | --- |
| HUMAN EMBRYO | 1239/2316 | 5.68E-33 | 6.14E-31 |
| NEURONAL EPITHELIUM | 1209/2316 | 1.98E-26 | 1.07E-24 |
| FIBROBLAST | 1134/2316 | 2.14E-13 | 5.77E-12 |
| MYOBLAST | 1134/2316 | 2.14E-13 | 5.77E-12 |
| ASTROCYTE | 1086/2316 | 1.58E-07 | 3.4E-06 |
| PREFRONTAL CORTEX | 1044/2316 | 0.000587 | 0.010574 |
| LIVER (BULK TISSUE) | 1019/2316 | 0.016579 | 0.255786 |

## Slide 17
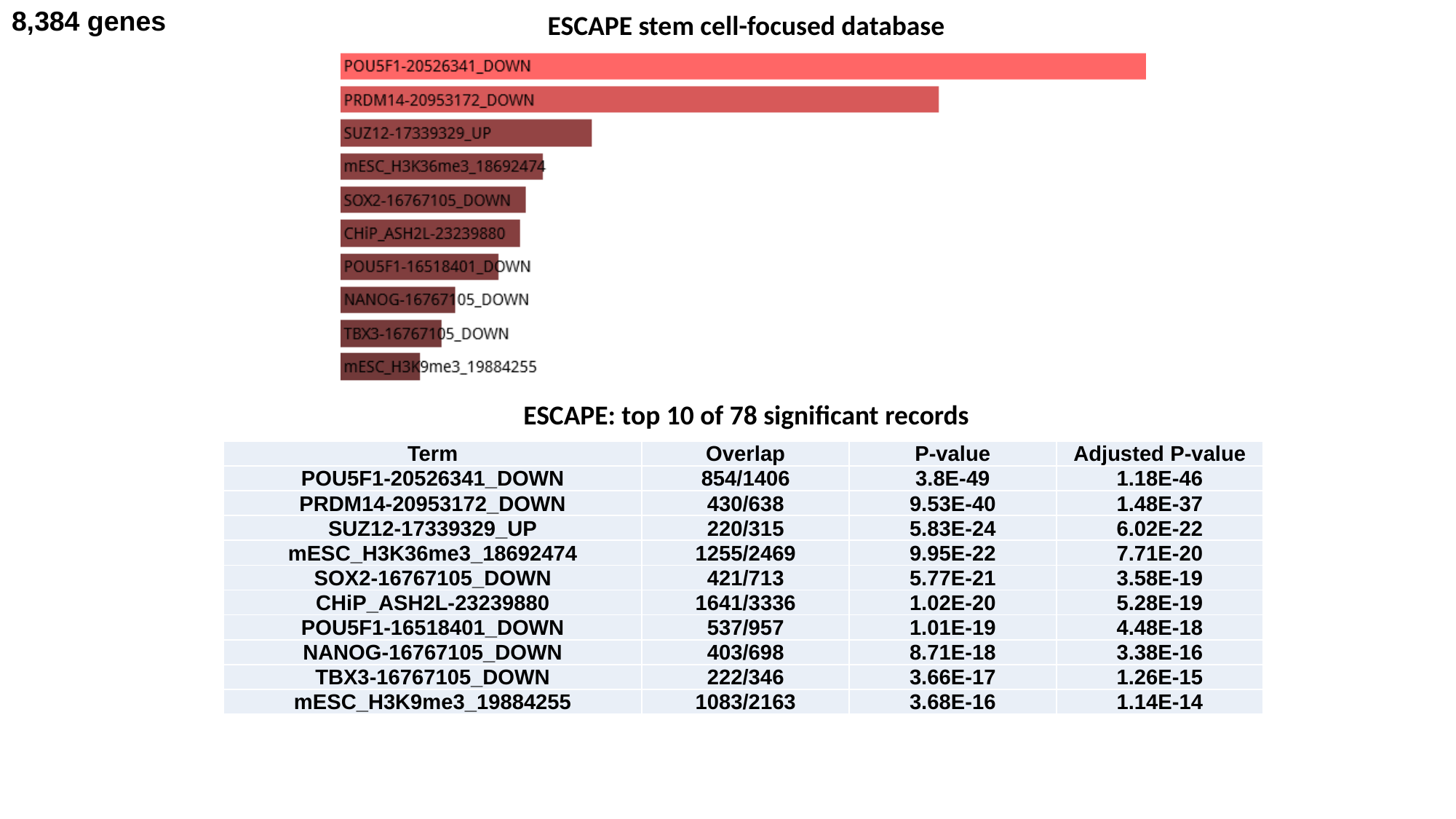

8,384 genes
ESCAPE stem cell-focused database
ESCAPE: top 10 of 78 significant records
| Term | Overlap | P-value | Adjusted P-value |
| --- | --- | --- | --- |
| POU5F1-20526341\_DOWN | 854/1406 | 3.8E-49 | 1.18E-46 |
| PRDM14-20953172\_DOWN | 430/638 | 9.53E-40 | 1.48E-37 |
| SUZ12-17339329\_UP | 220/315 | 5.83E-24 | 6.02E-22 |
| mESC\_H3K36me3\_18692474 | 1255/2469 | 9.95E-22 | 7.71E-20 |
| SOX2-16767105\_DOWN | 421/713 | 5.77E-21 | 3.58E-19 |
| CHiP\_ASH2L-23239880 | 1641/3336 | 1.02E-20 | 5.28E-19 |
| POU5F1-16518401\_DOWN | 537/957 | 1.01E-19 | 4.48E-18 |
| NANOG-16767105\_DOWN | 403/698 | 8.71E-18 | 3.38E-16 |
| TBX3-16767105\_DOWN | 222/346 | 3.66E-17 | 1.26E-15 |
| mESC\_H3K9me3\_19884255 | 1083/2163 | 3.68E-16 | 1.14E-14 |

## Slide 18
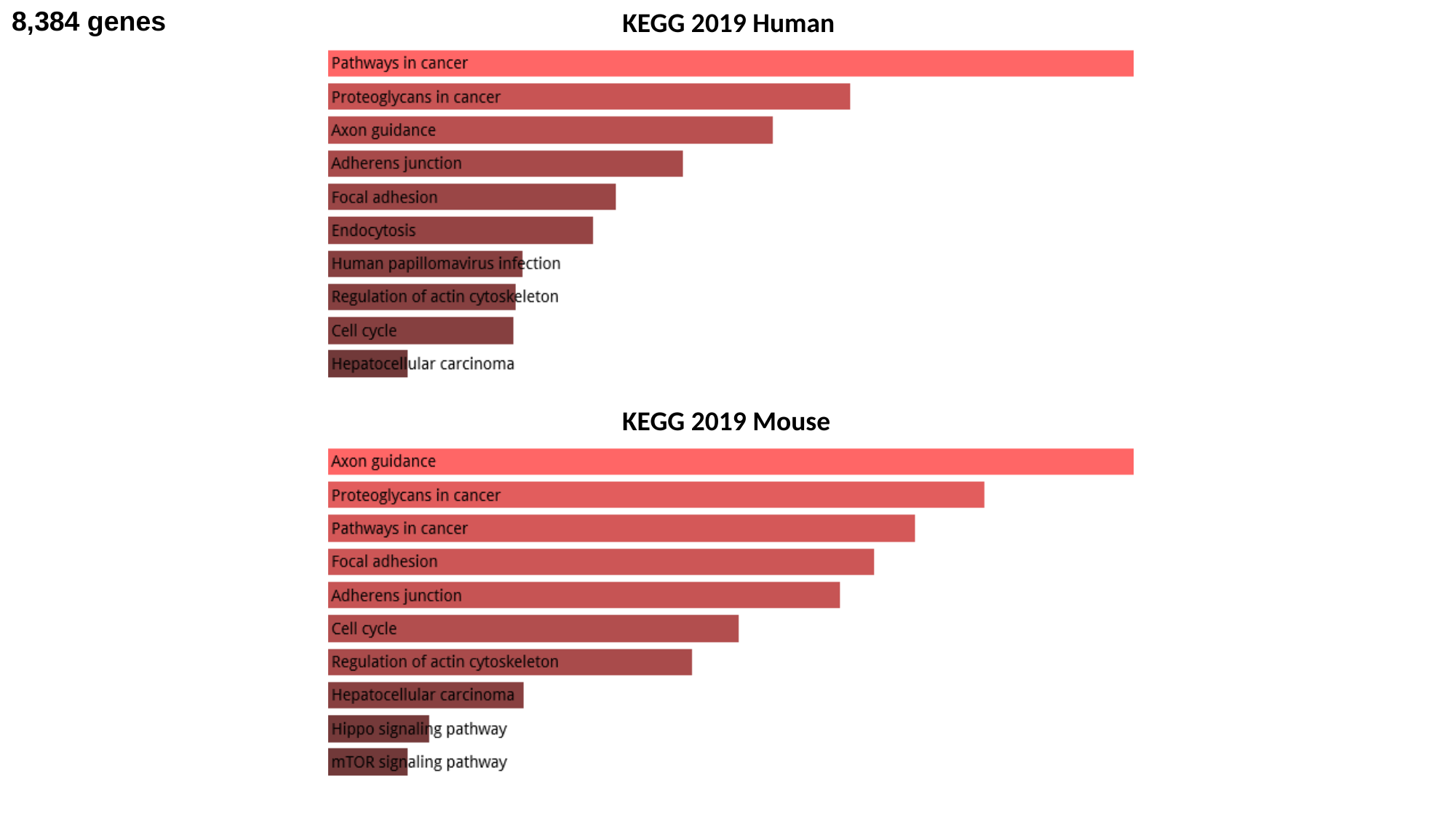

KEGG 2019 Human
8,384 genes
KEGG 2019 Mouse

## Slide 19
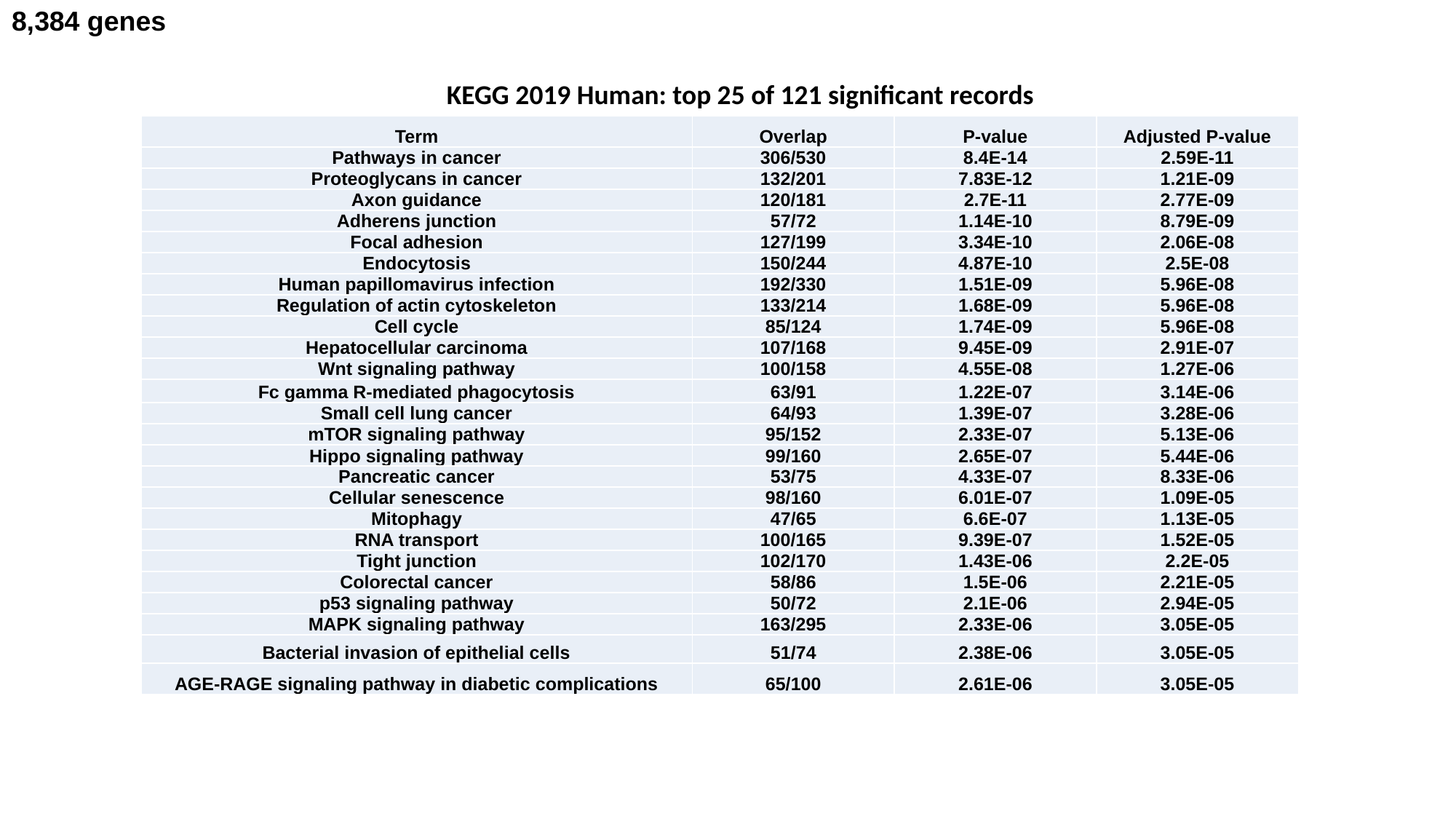

8,384 genes
KEGG 2019 Human: top 25 of 121 significant records
| Term | Overlap | P-value | Adjusted P-value |
| --- | --- | --- | --- |
| Pathways in cancer | 306/530 | 8.4E-14 | 2.59E-11 |
| Proteoglycans in cancer | 132/201 | 7.83E-12 | 1.21E-09 |
| Axon guidance | 120/181 | 2.7E-11 | 2.77E-09 |
| Adherens junction | 57/72 | 1.14E-10 | 8.79E-09 |
| Focal adhesion | 127/199 | 3.34E-10 | 2.06E-08 |
| Endocytosis | 150/244 | 4.87E-10 | 2.5E-08 |
| Human papillomavirus infection | 192/330 | 1.51E-09 | 5.96E-08 |
| Regulation of actin cytoskeleton | 133/214 | 1.68E-09 | 5.96E-08 |
| Cell cycle | 85/124 | 1.74E-09 | 5.96E-08 |
| Hepatocellular carcinoma | 107/168 | 9.45E-09 | 2.91E-07 |
| Wnt signaling pathway | 100/158 | 4.55E-08 | 1.27E-06 |
| Fc gamma R-mediated phagocytosis | 63/91 | 1.22E-07 | 3.14E-06 |
| Small cell lung cancer | 64/93 | 1.39E-07 | 3.28E-06 |
| mTOR signaling pathway | 95/152 | 2.33E-07 | 5.13E-06 |
| Hippo signaling pathway | 99/160 | 2.65E-07 | 5.44E-06 |
| Pancreatic cancer | 53/75 | 4.33E-07 | 8.33E-06 |
| Cellular senescence | 98/160 | 6.01E-07 | 1.09E-05 |
| Mitophagy | 47/65 | 6.6E-07 | 1.13E-05 |
| RNA transport | 100/165 | 9.39E-07 | 1.52E-05 |
| Tight junction | 102/170 | 1.43E-06 | 2.2E-05 |
| Colorectal cancer | 58/86 | 1.5E-06 | 2.21E-05 |
| p53 signaling pathway | 50/72 | 2.1E-06 | 2.94E-05 |
| MAPK signaling pathway | 163/295 | 2.33E-06 | 3.05E-05 |
| Bacterial invasion of epithelial cells | 51/74 | 2.38E-06 | 3.05E-05 |
| AGE-RAGE signaling pathway in diabetic complications | 65/100 | 2.61E-06 | 3.05E-05 |

## Slide 20
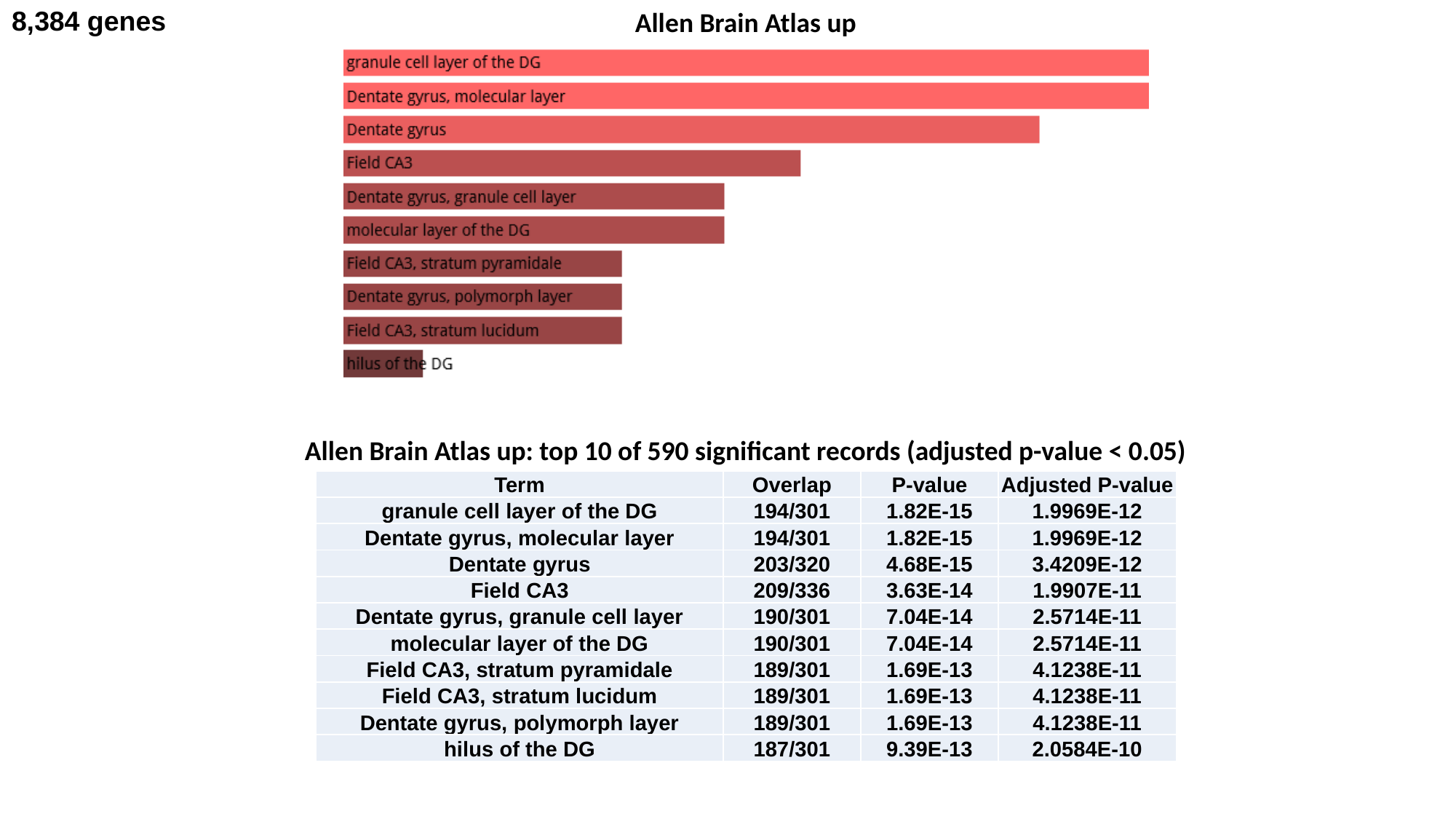

Allen Brain Atlas up
8,384 genes
Allen Brain Atlas up: top 10 of 590 significant records (adjusted p-value < 0.05)
| Term | Overlap | P-value | Adjusted P-value |
| --- | --- | --- | --- |
| granule cell layer of the DG | 194/301 | 1.82E-15 | 1.9969E-12 |
| Dentate gyrus, molecular layer | 194/301 | 1.82E-15 | 1.9969E-12 |
| Dentate gyrus | 203/320 | 4.68E-15 | 3.4209E-12 |
| Field CA3 | 209/336 | 3.63E-14 | 1.9907E-11 |
| Dentate gyrus, granule cell layer | 190/301 | 7.04E-14 | 2.5714E-11 |
| molecular layer of the DG | 190/301 | 7.04E-14 | 2.5714E-11 |
| Field CA3, stratum pyramidale | 189/301 | 1.69E-13 | 4.1238E-11 |
| Field CA3, stratum lucidum | 189/301 | 1.69E-13 | 4.1238E-11 |
| Dentate gyrus, polymorph layer | 189/301 | 1.69E-13 | 4.1238E-11 |
| hilus of the DG | 187/301 | 9.39E-13 | 2.0584E-10 |

## Slide 21
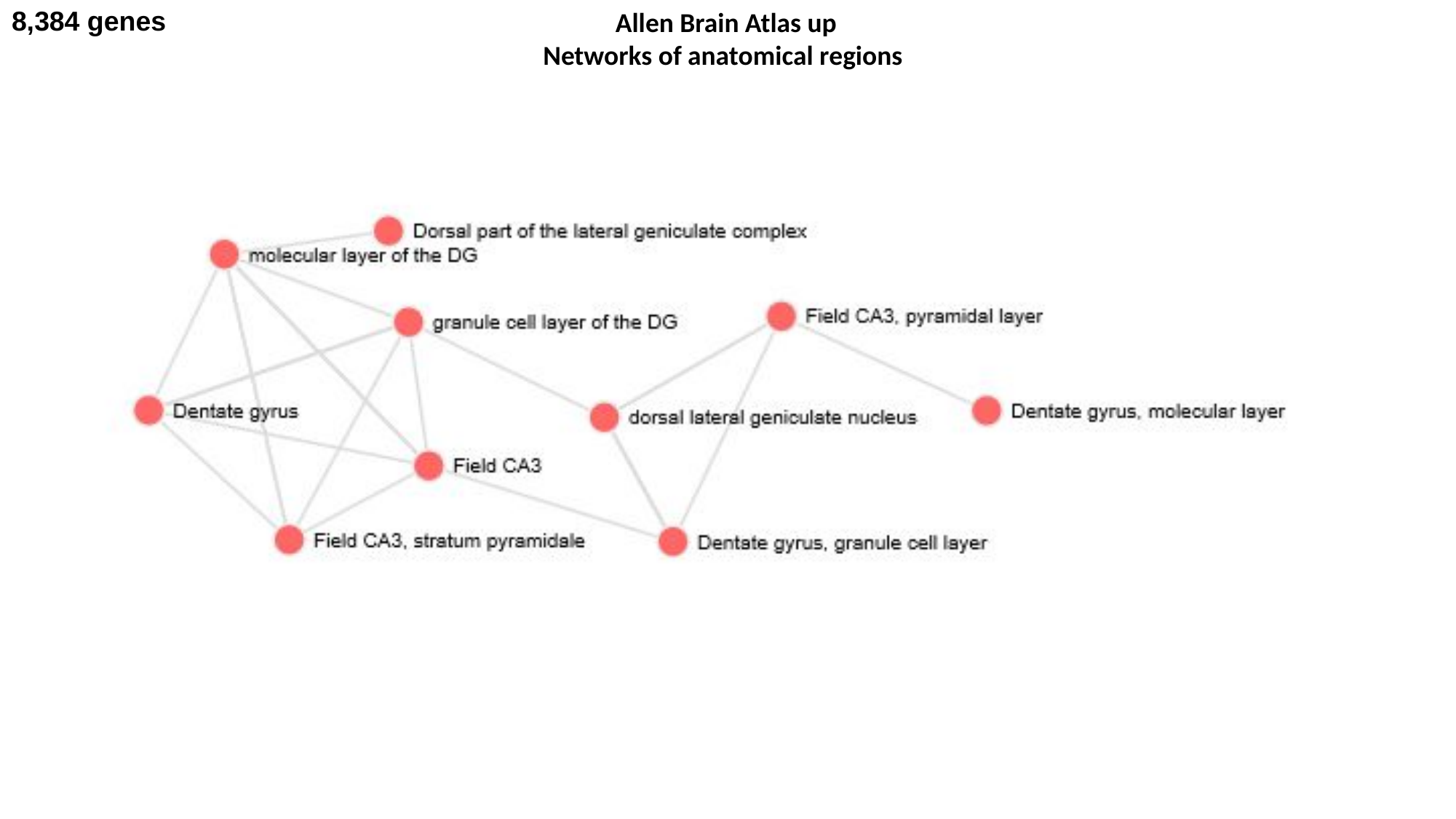

Allen Brain Atlas up
Networks of anatomical regions
8,384 genes

## Slide 22
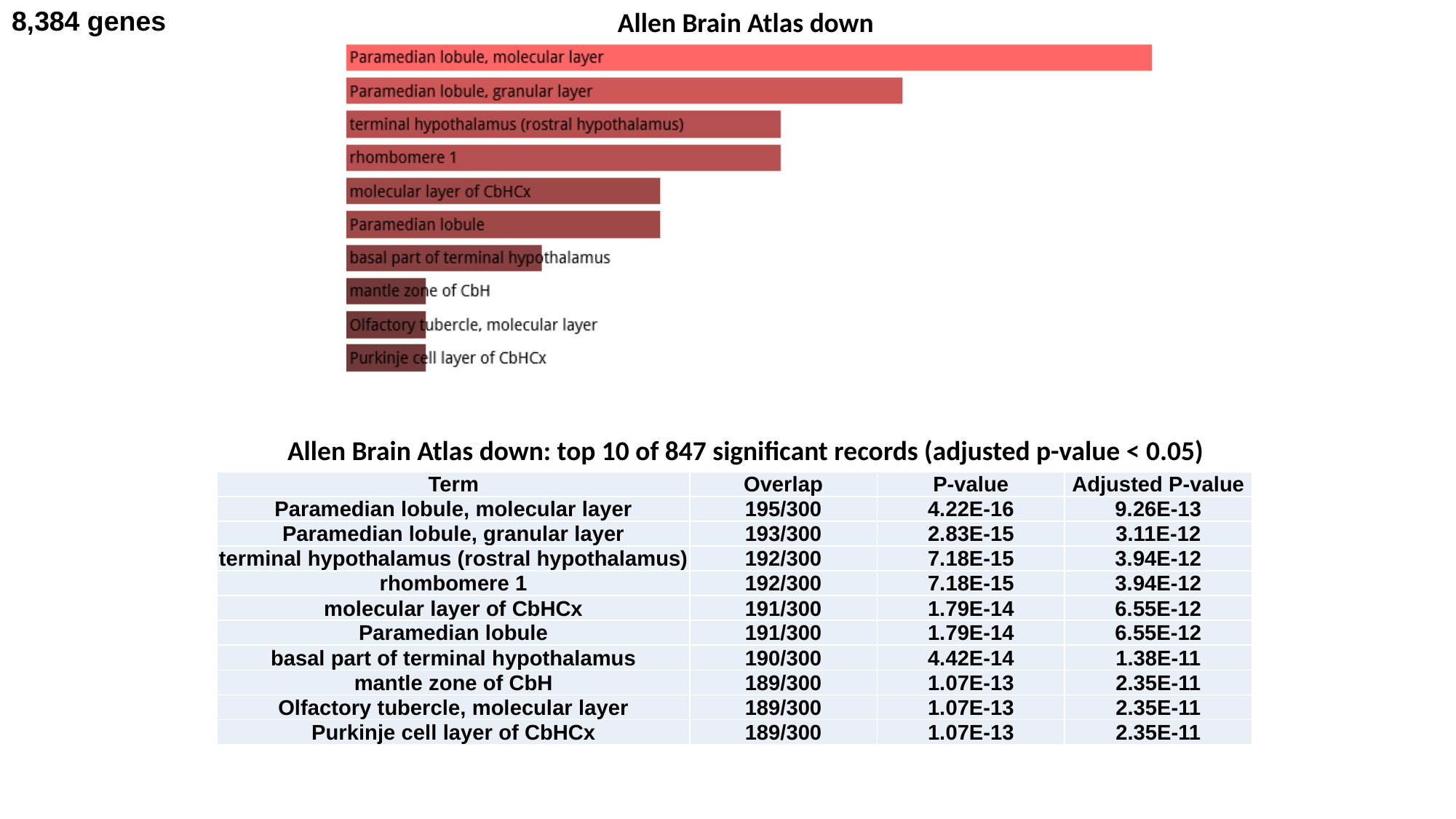

Allen Brain Atlas down
8,384 genes
Allen Brain Atlas down: top 10 of 847 significant records (adjusted p-value < 0.05)
| Term | Overlap | P-value | Adjusted P-value |
| --- | --- | --- | --- |
| Paramedian lobule, molecular layer | 195/300 | 4.22E-16 | 9.26E-13 |
| Paramedian lobule, granular layer | 193/300 | 2.83E-15 | 3.11E-12 |
| terminal hypothalamus (rostral hypothalamus) | 192/300 | 7.18E-15 | 3.94E-12 |
| rhombomere 1 | 192/300 | 7.18E-15 | 3.94E-12 |
| molecular layer of CbHCx | 191/300 | 1.79E-14 | 6.55E-12 |
| Paramedian lobule | 191/300 | 1.79E-14 | 6.55E-12 |
| basal part of terminal hypothalamus | 190/300 | 4.42E-14 | 1.38E-11 |
| mantle zone of CbH | 189/300 | 1.07E-13 | 2.35E-11 |
| Olfactory tubercle, molecular layer | 189/300 | 1.07E-13 | 2.35E-11 |
| Purkinje cell layer of CbHCx | 189/300 | 1.07E-13 | 2.35E-11 |

## Slide 23
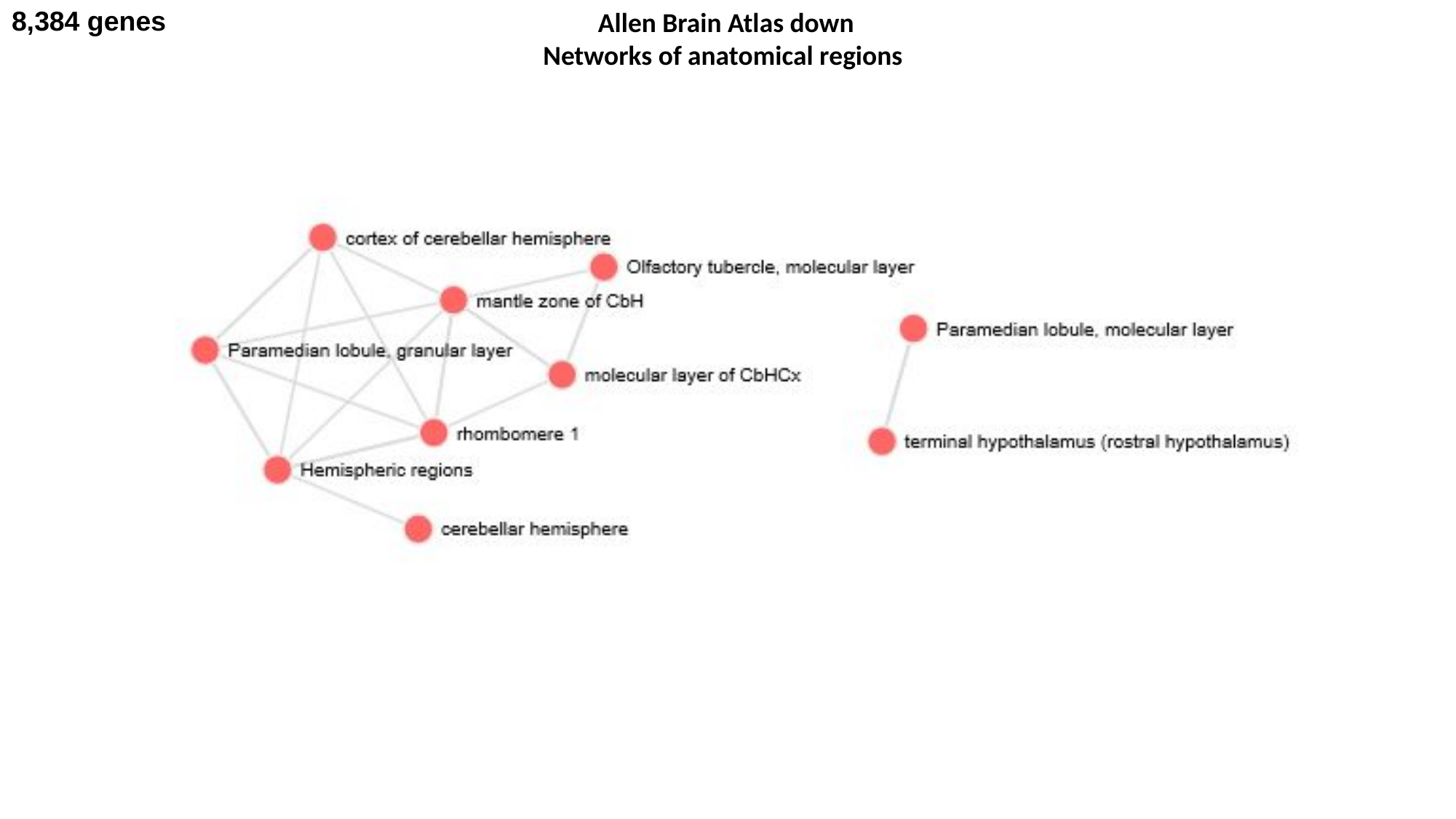

Allen Brain Atlas down
Networks of anatomical regions
8,384 genes

## Slide 24
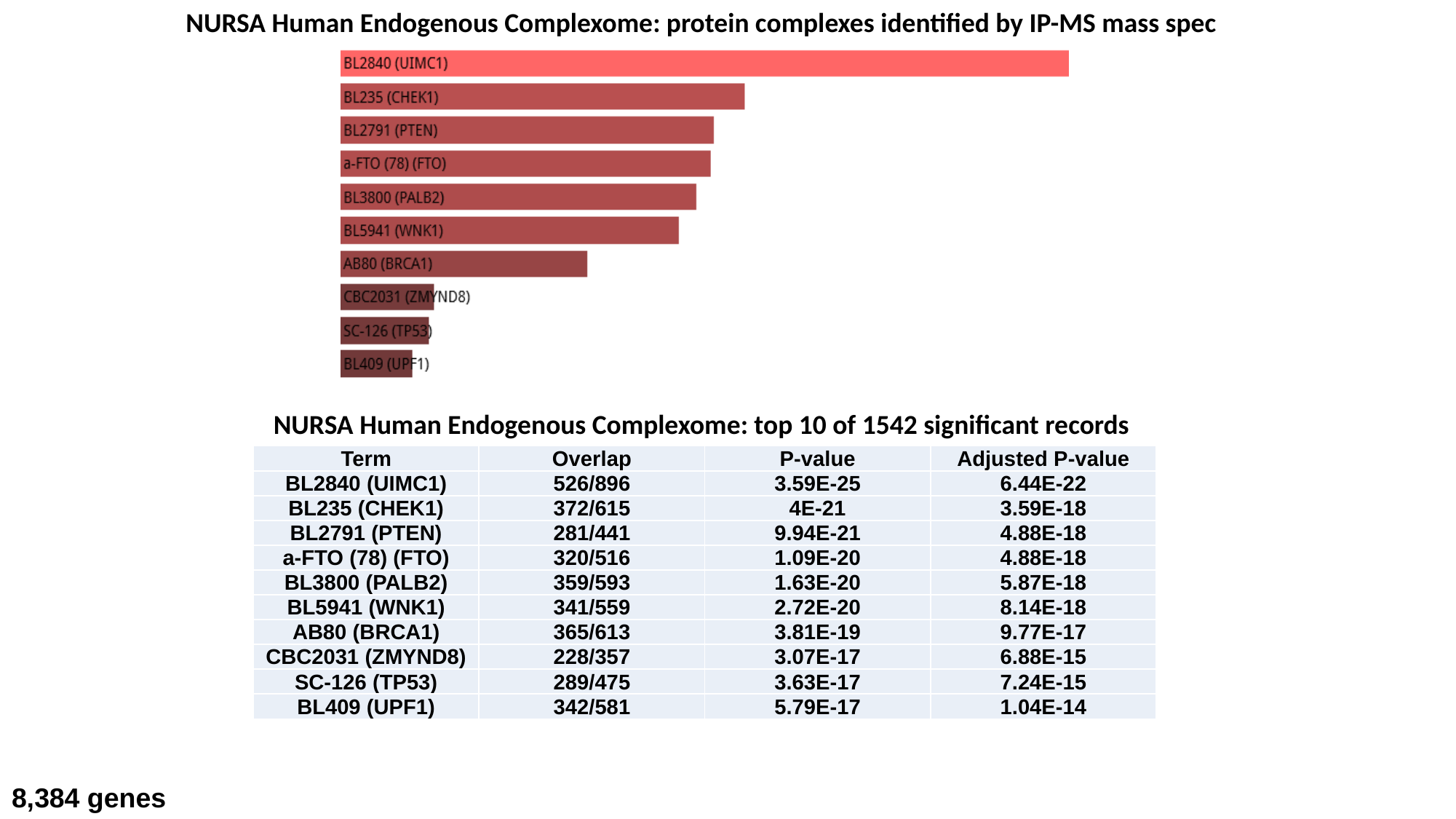

NURSA Human Endogenous Complexome: protein complexes identified by IP-MS mass spec
NURSA Human Endogenous Complexome: top 10 of 1542 significant records
| Term | Overlap | P-value | Adjusted P-value |
| --- | --- | --- | --- |
| BL2840 (UIMC1) | 526/896 | 3.59E-25 | 6.44E-22 |
| BL235 (CHEK1) | 372/615 | 4E-21 | 3.59E-18 |
| BL2791 (PTEN) | 281/441 | 9.94E-21 | 4.88E-18 |
| a-FTO (78) (FTO) | 320/516 | 1.09E-20 | 4.88E-18 |
| BL3800 (PALB2) | 359/593 | 1.63E-20 | 5.87E-18 |
| BL5941 (WNK1) | 341/559 | 2.72E-20 | 8.14E-18 |
| AB80 (BRCA1) | 365/613 | 3.81E-19 | 9.77E-17 |
| CBC2031 (ZMYND8) | 228/357 | 3.07E-17 | 6.88E-15 |
| SC-126 (TP53) | 289/475 | 3.63E-17 | 7.24E-15 |
| BL409 (UPF1) | 342/581 | 5.79E-17 | 1.04E-14 |
8,384 genes

## Slide 25
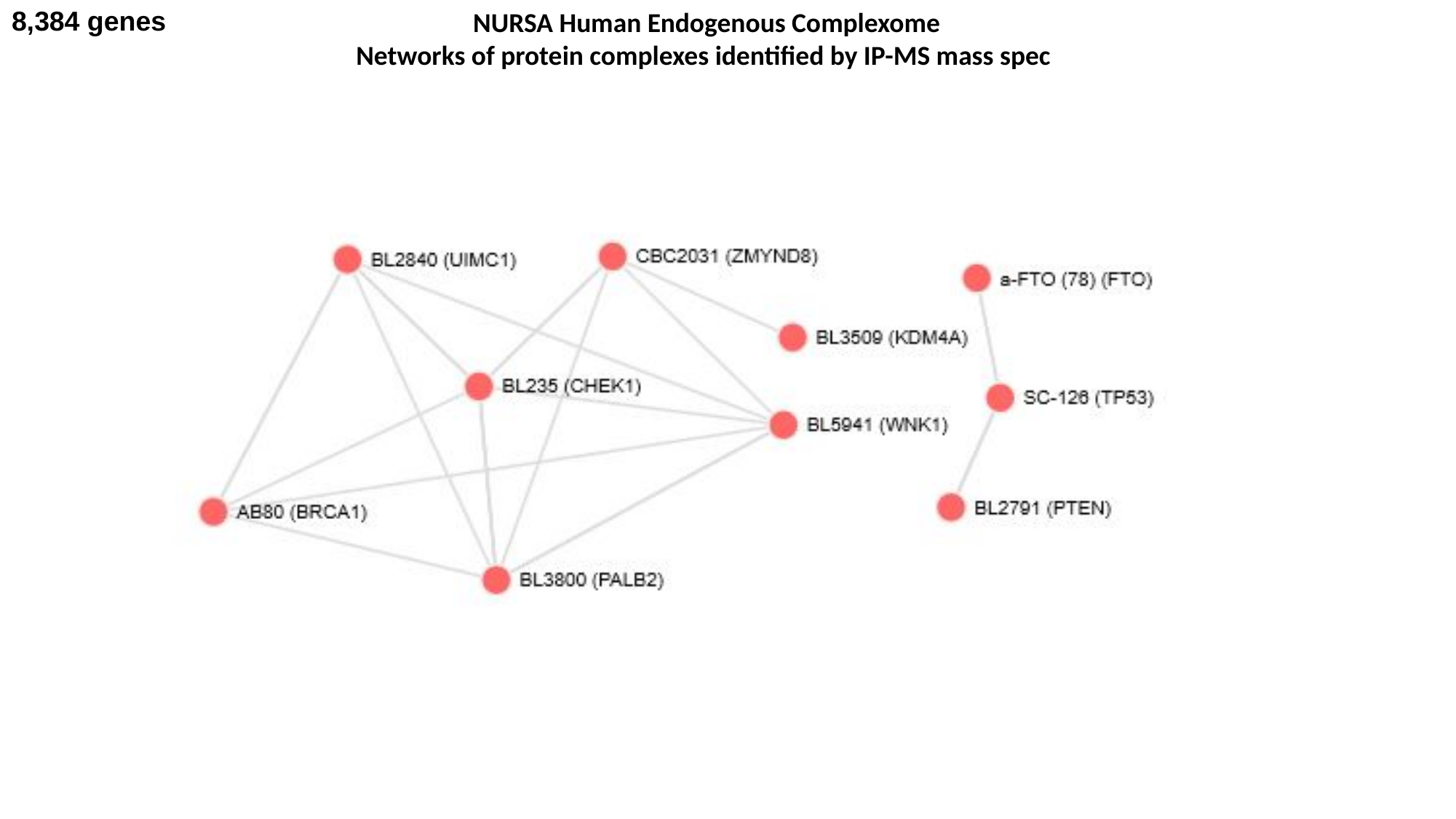

NURSA Human Endogenous Complexome
Networks of protein complexes identified by IP-MS mass spec
8,384 genes

## Slide 26
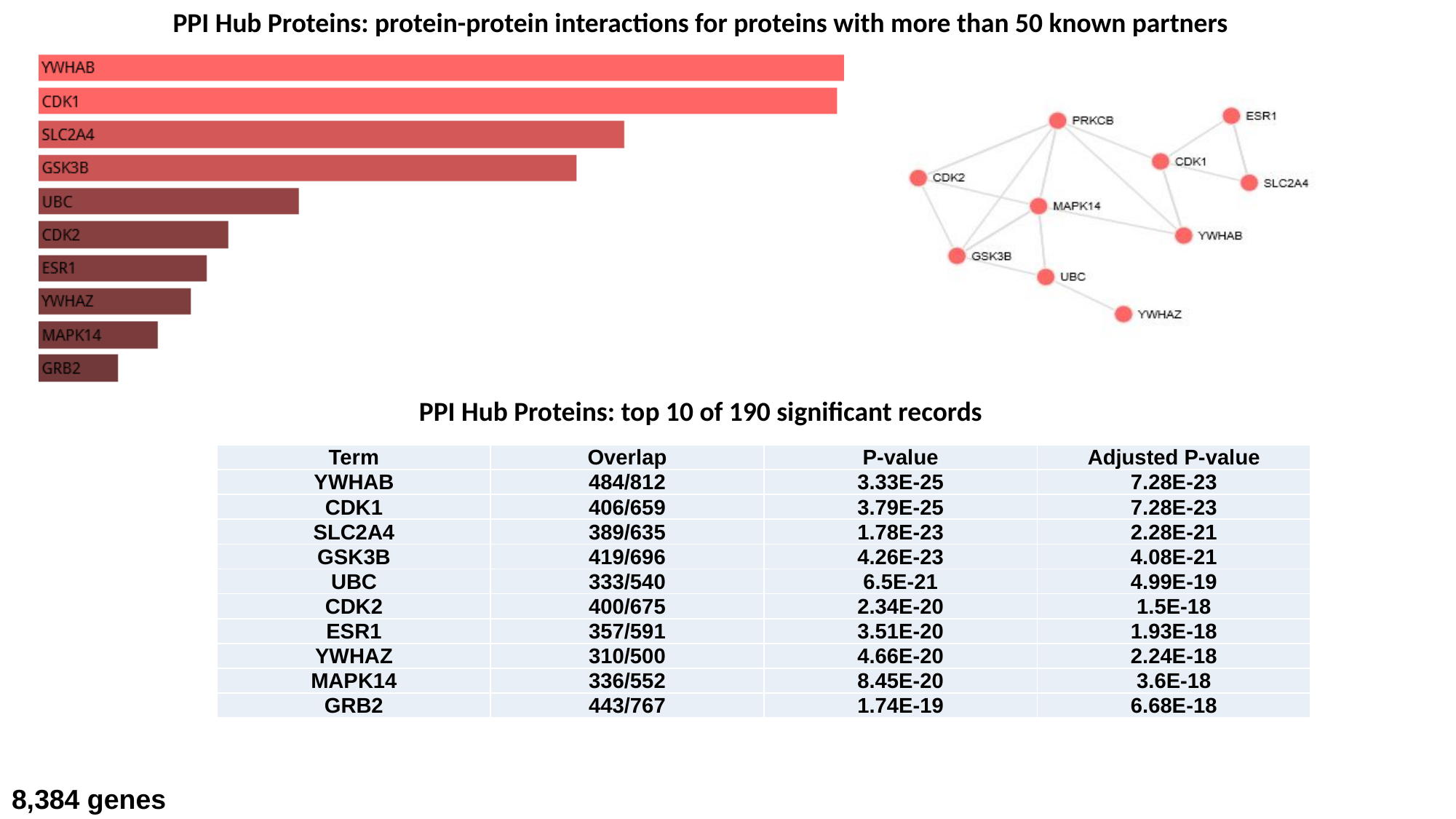

PPI Hub Proteins: protein-protein interactions for proteins with more than 50 known partners
PPI Hub Proteins: top 10 of 190 significant records
| Term | Overlap | P-value | Adjusted P-value |
| --- | --- | --- | --- |
| YWHAB | 484/812 | 3.33E-25 | 7.28E-23 |
| CDK1 | 406/659 | 3.79E-25 | 7.28E-23 |
| SLC2A4 | 389/635 | 1.78E-23 | 2.28E-21 |
| GSK3B | 419/696 | 4.26E-23 | 4.08E-21 |
| UBC | 333/540 | 6.5E-21 | 4.99E-19 |
| CDK2 | 400/675 | 2.34E-20 | 1.5E-18 |
| ESR1 | 357/591 | 3.51E-20 | 1.93E-18 |
| YWHAZ | 310/500 | 4.66E-20 | 2.24E-18 |
| MAPK14 | 336/552 | 8.45E-20 | 3.6E-18 |
| GRB2 | 443/767 | 1.74E-19 | 6.68E-18 |
8,384 genes

## Slide 27
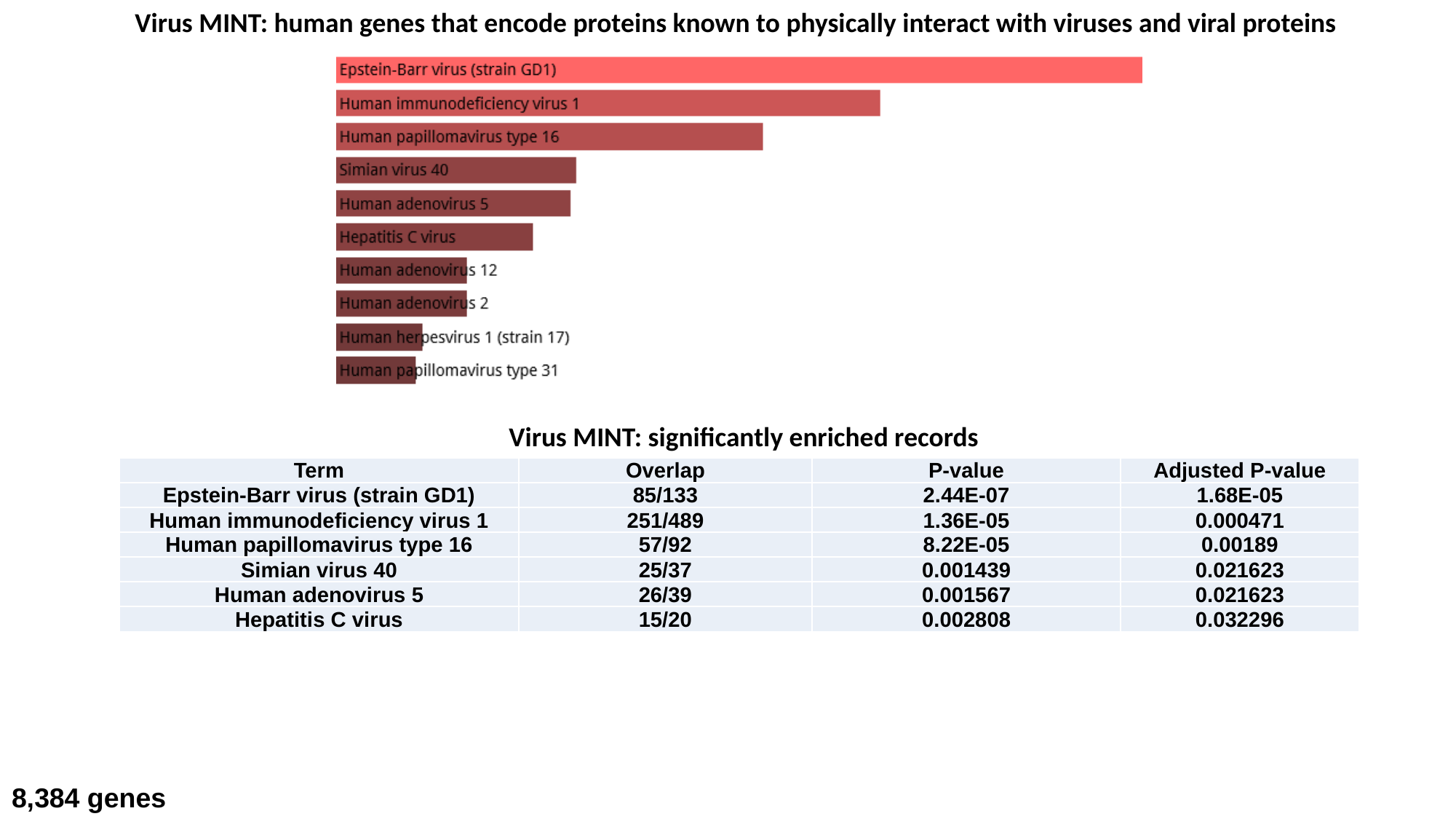

Virus MINT: human genes that encode proteins known to physically interact with viruses and viral proteins
Virus MINT: significantly enriched records
| Term | Overlap | P-value | Adjusted P-value |
| --- | --- | --- | --- |
| Epstein-Barr virus (strain GD1) | 85/133 | 2.44E-07 | 1.68E-05 |
| Human immunodeficiency virus 1 | 251/489 | 1.36E-05 | 0.000471 |
| Human papillomavirus type 16 | 57/92 | 8.22E-05 | 0.00189 |
| Simian virus 40 | 25/37 | 0.001439 | 0.021623 |
| Human adenovirus 5 | 26/39 | 0.001567 | 0.021623 |
| Hepatitis C virus | 15/20 | 0.002808 | 0.032296 |
8,384 genes

## Slide 28
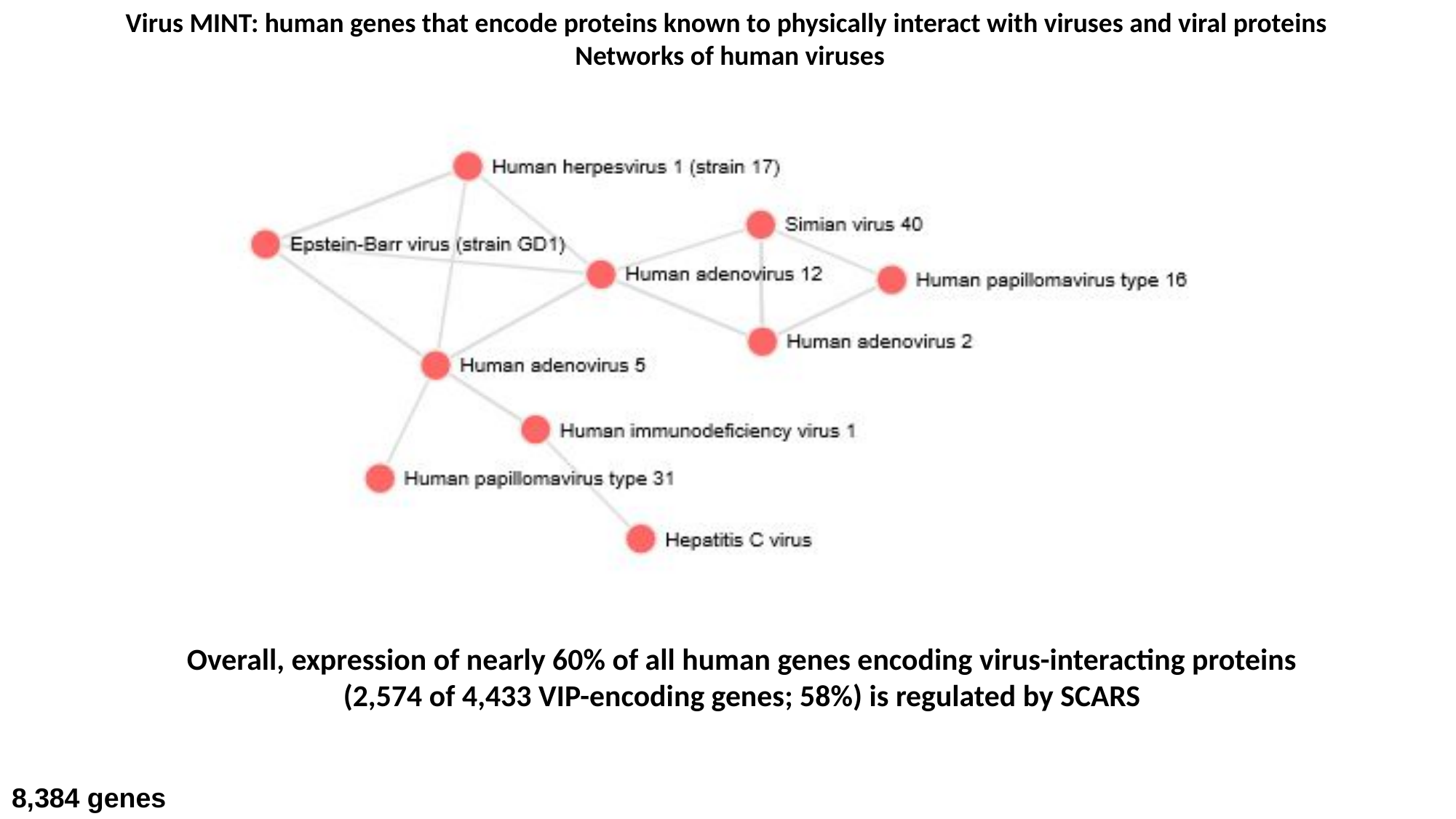

Virus MINT: human genes that encode proteins known to physically interact with viruses and viral proteins
Networks of human viruses
Overall, expression of nearly 60% of all human genes encoding virus-interacting proteins
(2,574 of 4,433 VIP-encoding genes; 58%) is regulated by SCARS
8,384 genes

## Slide 29
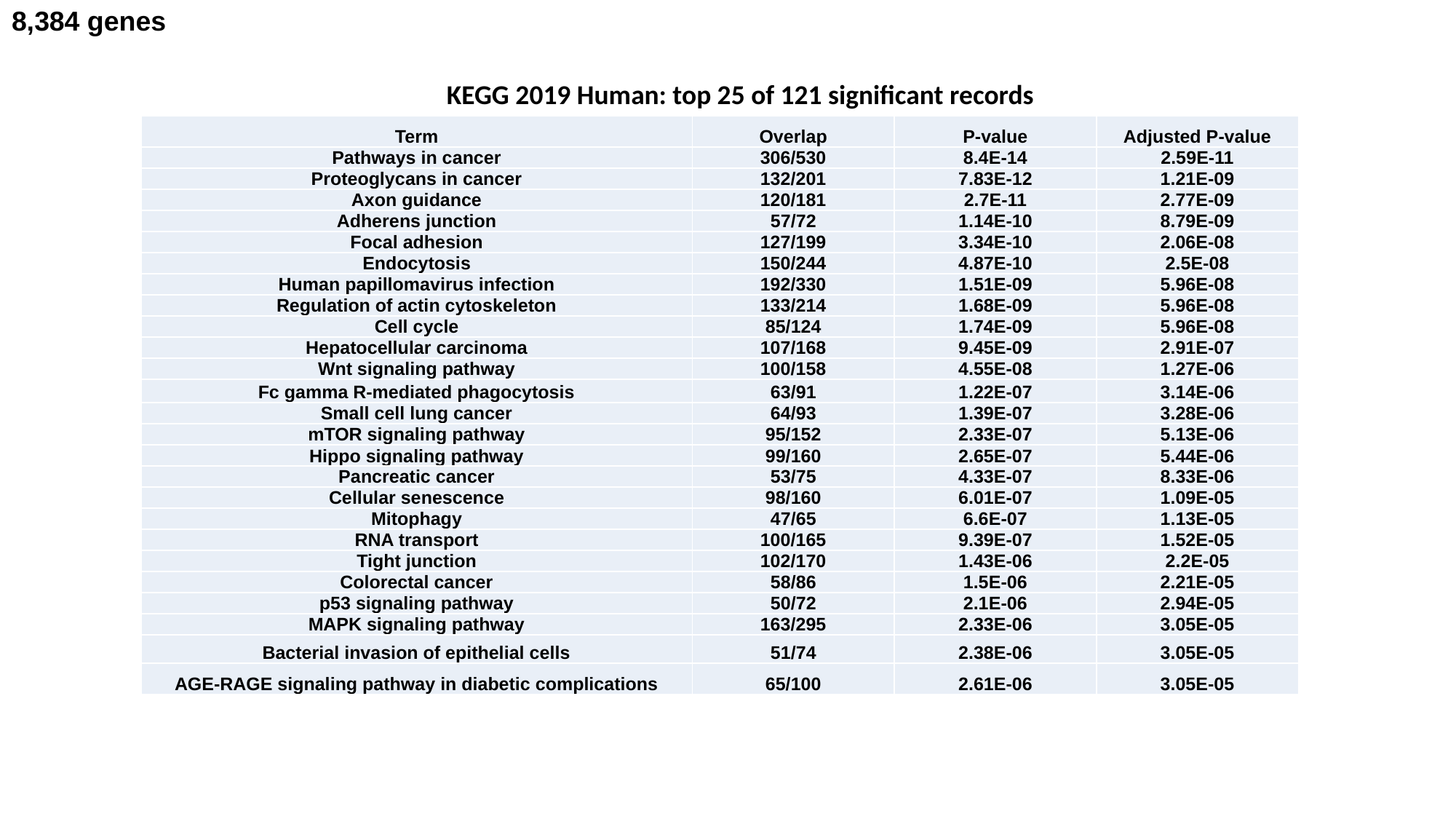

8,384 genes
KEGG 2019 Human: top 25 of 121 significant records
| Term | Overlap | P-value | Adjusted P-value |
| --- | --- | --- | --- |
| Pathways in cancer | 306/530 | 8.4E-14 | 2.59E-11 |
| Proteoglycans in cancer | 132/201 | 7.83E-12 | 1.21E-09 |
| Axon guidance | 120/181 | 2.7E-11 | 2.77E-09 |
| Adherens junction | 57/72 | 1.14E-10 | 8.79E-09 |
| Focal adhesion | 127/199 | 3.34E-10 | 2.06E-08 |
| Endocytosis | 150/244 | 4.87E-10 | 2.5E-08 |
| Human papillomavirus infection | 192/330 | 1.51E-09 | 5.96E-08 |
| Regulation of actin cytoskeleton | 133/214 | 1.68E-09 | 5.96E-08 |
| Cell cycle | 85/124 | 1.74E-09 | 5.96E-08 |
| Hepatocellular carcinoma | 107/168 | 9.45E-09 | 2.91E-07 |
| Wnt signaling pathway | 100/158 | 4.55E-08 | 1.27E-06 |
| Fc gamma R-mediated phagocytosis | 63/91 | 1.22E-07 | 3.14E-06 |
| Small cell lung cancer | 64/93 | 1.39E-07 | 3.28E-06 |
| mTOR signaling pathway | 95/152 | 2.33E-07 | 5.13E-06 |
| Hippo signaling pathway | 99/160 | 2.65E-07 | 5.44E-06 |
| Pancreatic cancer | 53/75 | 4.33E-07 | 8.33E-06 |
| Cellular senescence | 98/160 | 6.01E-07 | 1.09E-05 |
| Mitophagy | 47/65 | 6.6E-07 | 1.13E-05 |
| RNA transport | 100/165 | 9.39E-07 | 1.52E-05 |
| Tight junction | 102/170 | 1.43E-06 | 2.2E-05 |
| Colorectal cancer | 58/86 | 1.5E-06 | 2.21E-05 |
| p53 signaling pathway | 50/72 | 2.1E-06 | 2.94E-05 |
| MAPK signaling pathway | 163/295 | 2.33E-06 | 3.05E-05 |
| Bacterial invasion of epithelial cells | 51/74 | 2.38E-06 | 3.05E-05 |
| AGE-RAGE signaling pathway in diabetic complications | 65/100 | 2.61E-06 | 3.05E-05 |
