# Supplementary material for: Genomics-Guided Drawing of Molecular and Pathophysiological Components of Malignant Regulatory Signatures Reveals a Pivotal Role in Human Diseases of Stem Cell-Associated Retroviral Sequences and Functionally-Active hESC Enhancers
Source: Front Oncol. 2021 Mar 31;11:638363. doi: 10.3389/fonc.2021.638363 (PMC8044830; doi:10.3389/fonc.2021.638363)
Supplement: Supplementary file 1 [file Presentation_1.zip › Supplemental Note S2. 2846 genes HSRS_SCARS.pptx]

## Slide 1
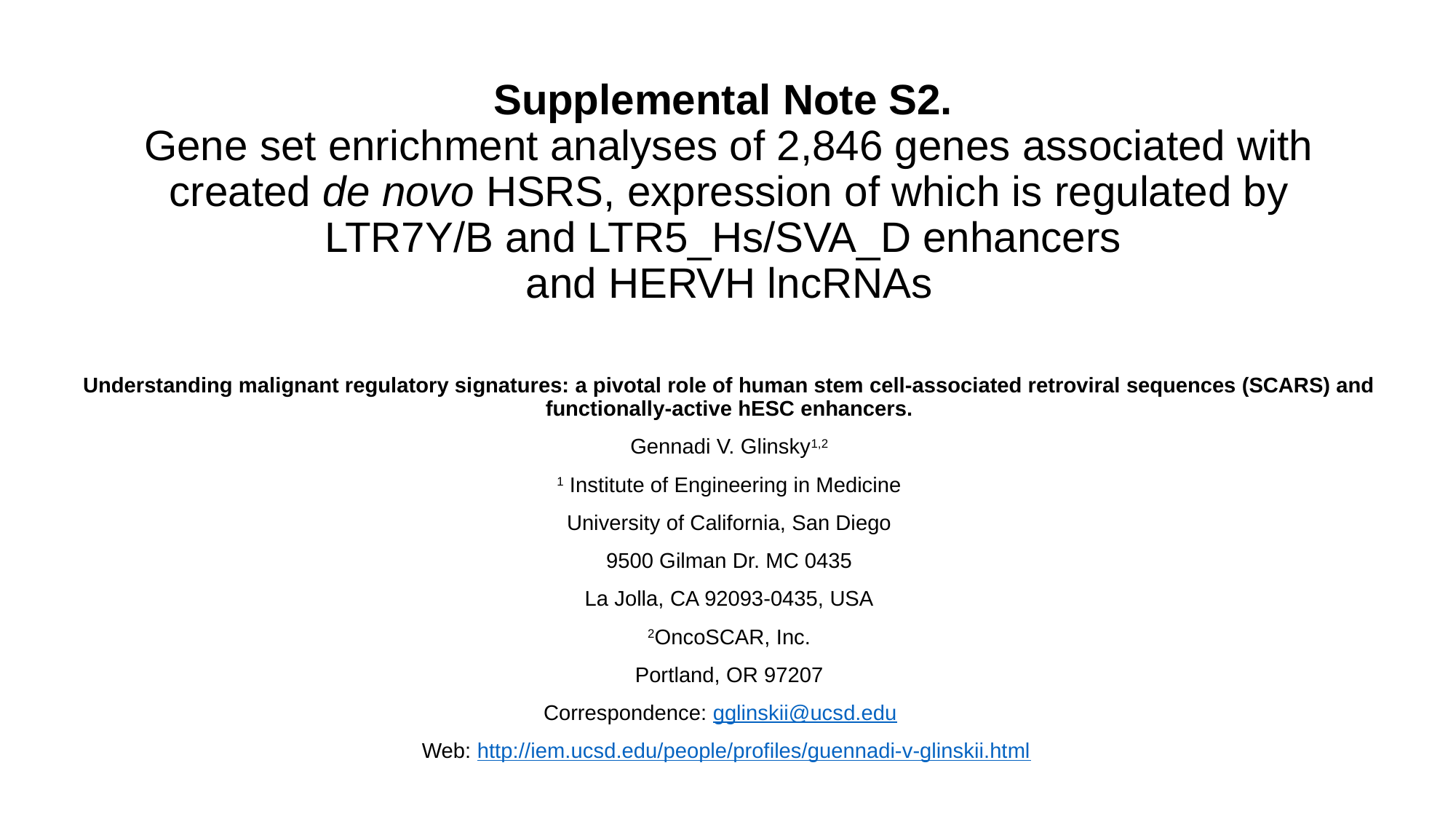

# Supplemental Note S2. Gene set enrichment analyses of 2,846 genes associated with created de novo HSRS, expression of which is regulated by LTR7Y/B and LTR5_Hs/SVA_D enhancers and HERVH lncRNAs
Understanding malignant regulatory signatures: a pivotal role of human stem cell-associated retroviral sequences (SCARS) and functionally-active hESC enhancers.
Gennadi V. Glinsky1,2
1 Institute of Engineering in Medicine
University of California, San Diego
9500 Gilman Dr. MC 0435
La Jolla, CA 92093-0435, USA
2OncoSCAR, Inc.
Portland, OR 97207
Correspondence: gglinskii@ucsd.edu
Web: http://iem.ucsd.edu/people/profiles/guennadi-v-glinskii.html

## Slide 2
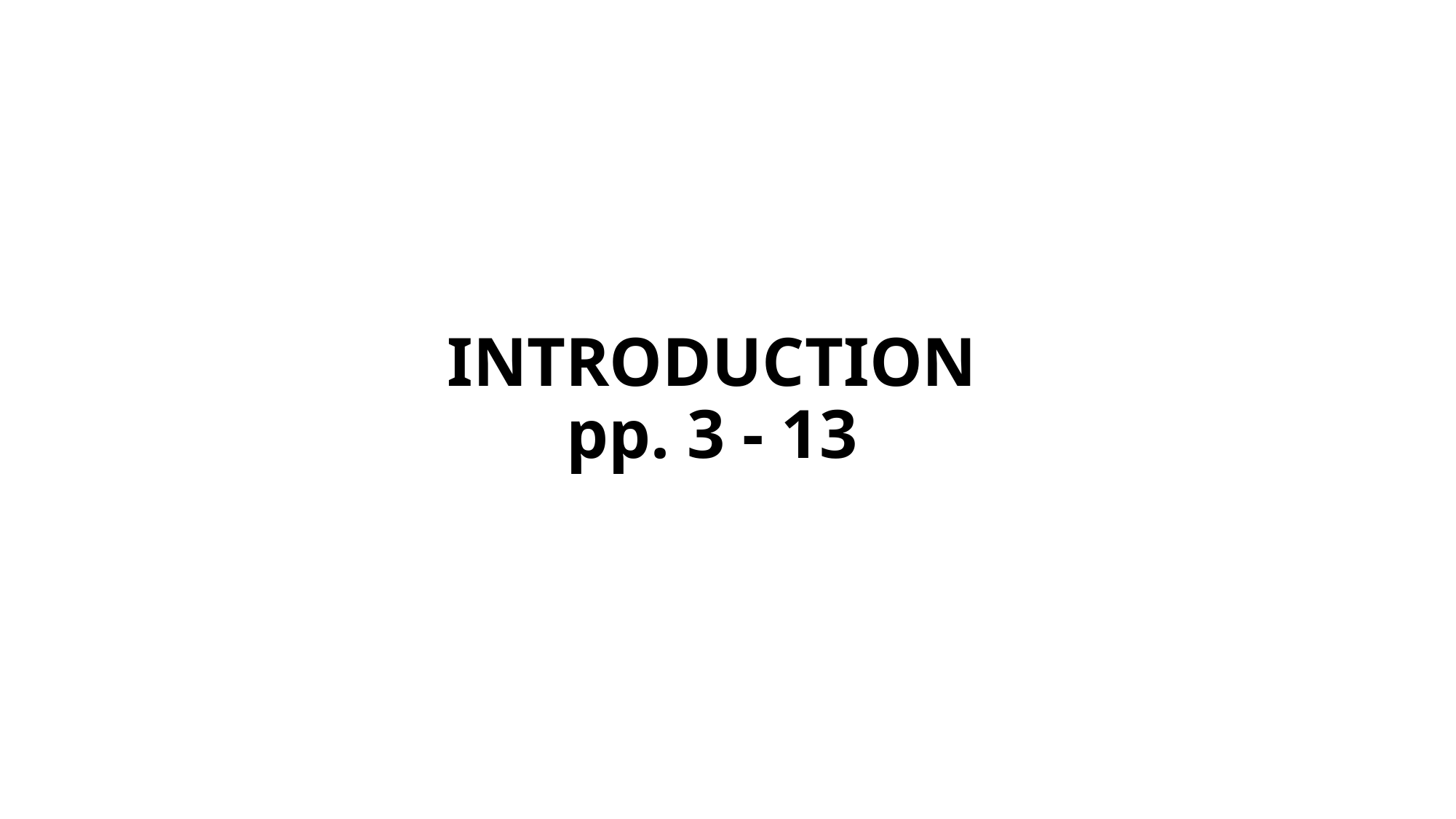

# INTRODUCTION pp. 3 - 13

## Slide 3
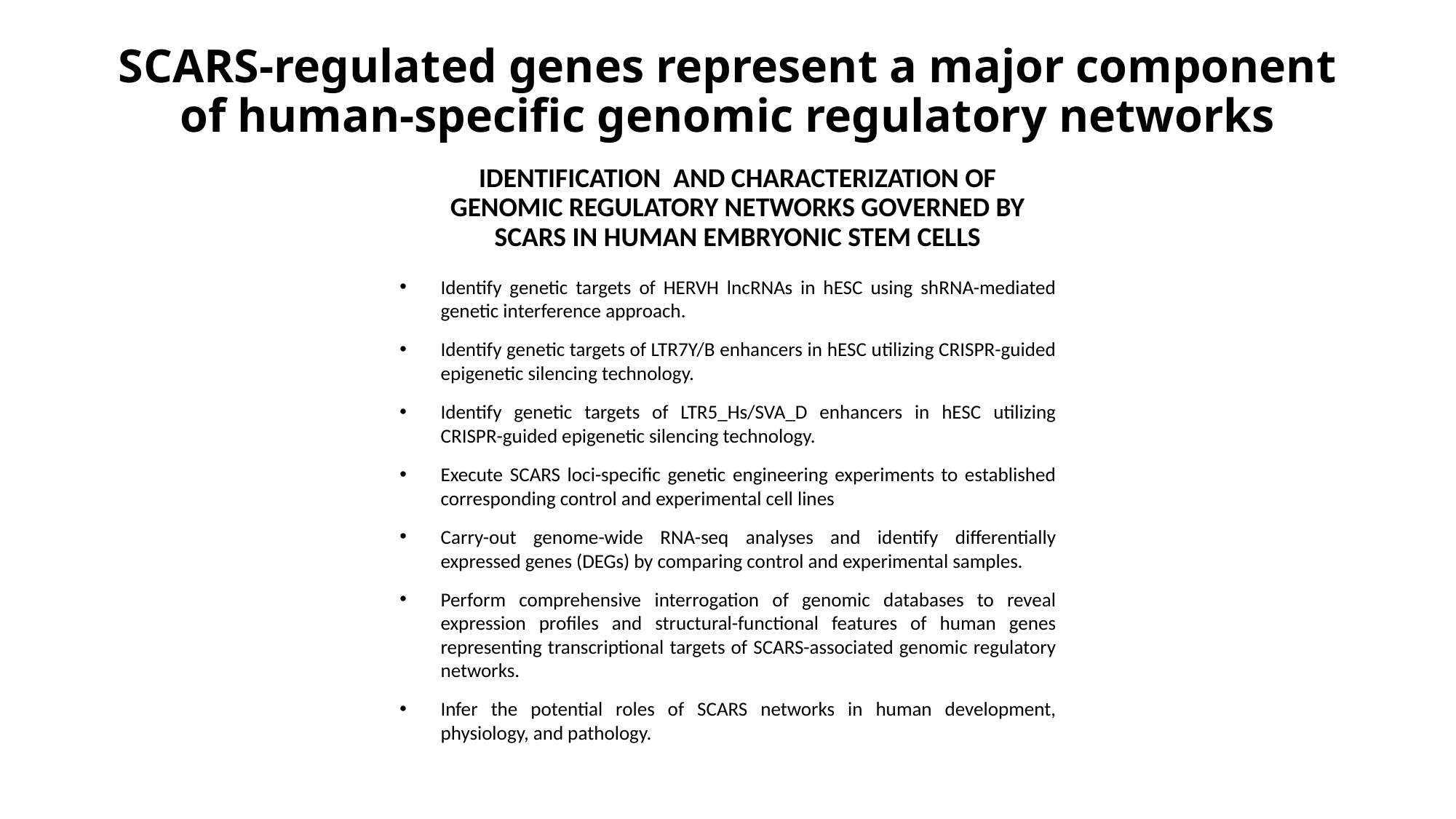

# SCARS-regulated genes represent a major component of human-specific genomic regulatory networks
IDENTIFICATION AND CHARACTERIZATION OF GENOMIC REGULATORY NETWORKS GOVERNED BY SCARS IN HUMAN EMBRYONIC STEM CELLS
Identify genetic targets of HERVH lncRNAs in hESC using shRNA-mediated genetic interference approach.
Identify genetic targets of LTR7Y/B enhancers in hESC utilizing CRISPR-guided epigenetic silencing technology.
Identify genetic targets of LTR5_Hs/SVA_D enhancers in hESC utilizing CRISPR-guided epigenetic silencing technology.
Execute SCARS loci-specific genetic engineering experiments to established corresponding control and experimental cell lines
Carry-out genome-wide RNA-seq analyses and identify differentially expressed genes (DEGs) by comparing control and experimental samples.
Perform comprehensive interrogation of genomic databases to reveal expression profiles and structural-functional features of human genes representing transcriptional targets of SCARS-associated genomic regulatory networks.
Infer the potential roles of SCARS networks in human development, physiology, and pathology.

## Slide 4
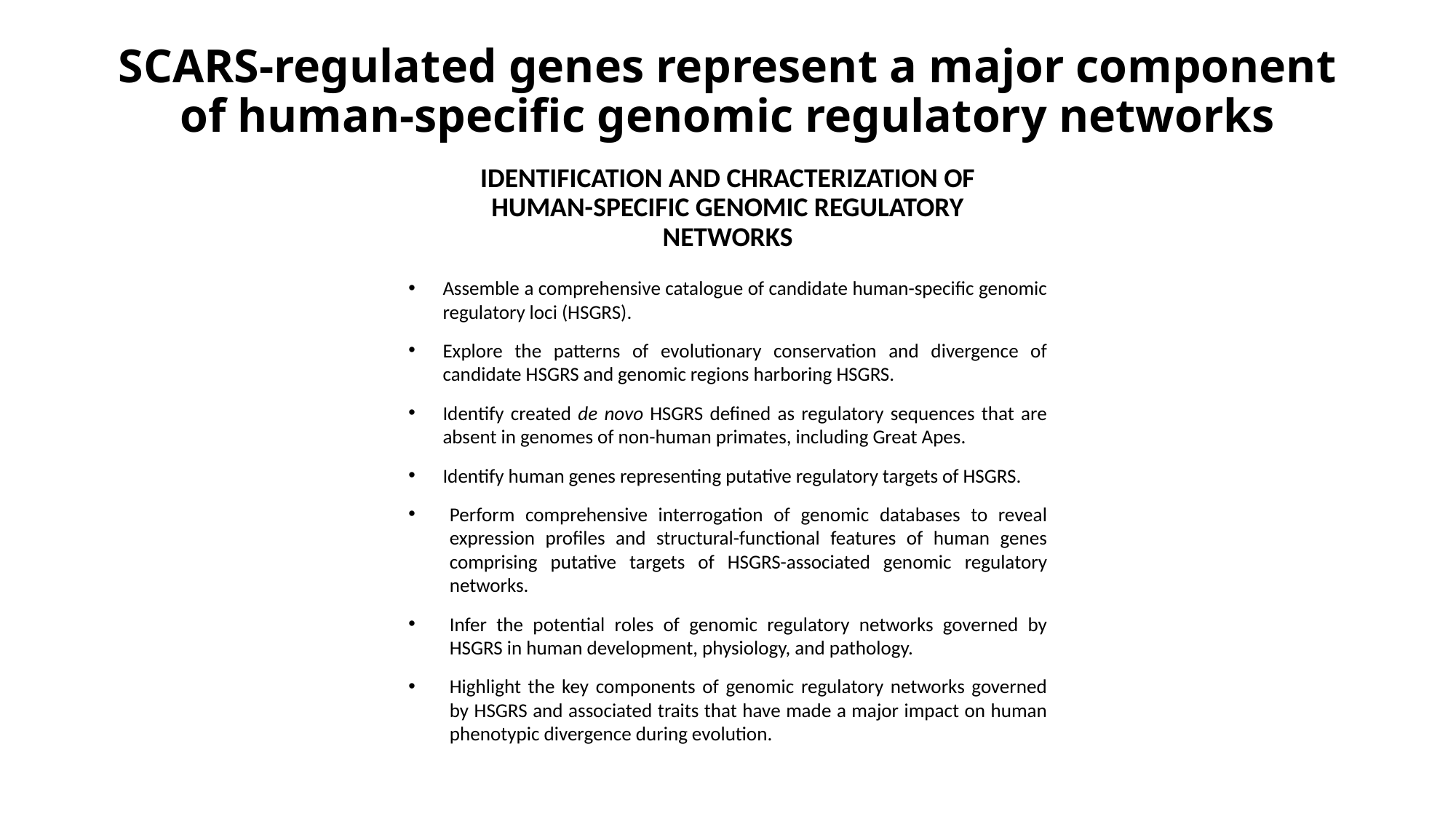

# SCARS-regulated genes represent a major component of human-specific genomic regulatory networks
IDENTIFICATION AND CHRACTERIZATION OF HUMAN-SPECIFIC GENOMIC REGULATORY NETWORKS
Assemble a comprehensive catalogue of candidate human-specific genomic regulatory loci (HSGRS).
Explore the patterns of evolutionary conservation and divergence of candidate HSGRS and genomic regions harboring HSGRS.
Identify created de novo HSGRS defined as regulatory sequences that are absent in genomes of non-human primates, including Great Apes.
Identify human genes representing putative regulatory targets of HSGRS.
Perform comprehensive interrogation of genomic databases to reveal expression profiles and structural-functional features of human genes comprising putative targets of HSGRS-associated genomic regulatory networks.
Infer the potential roles of genomic regulatory networks governed by HSGRS in human development, physiology, and pathology.
Highlight the key components of genomic regulatory networks governed by HSGRS and associated traits that have made a major impact on human phenotypic divergence during evolution.

## Slide 5
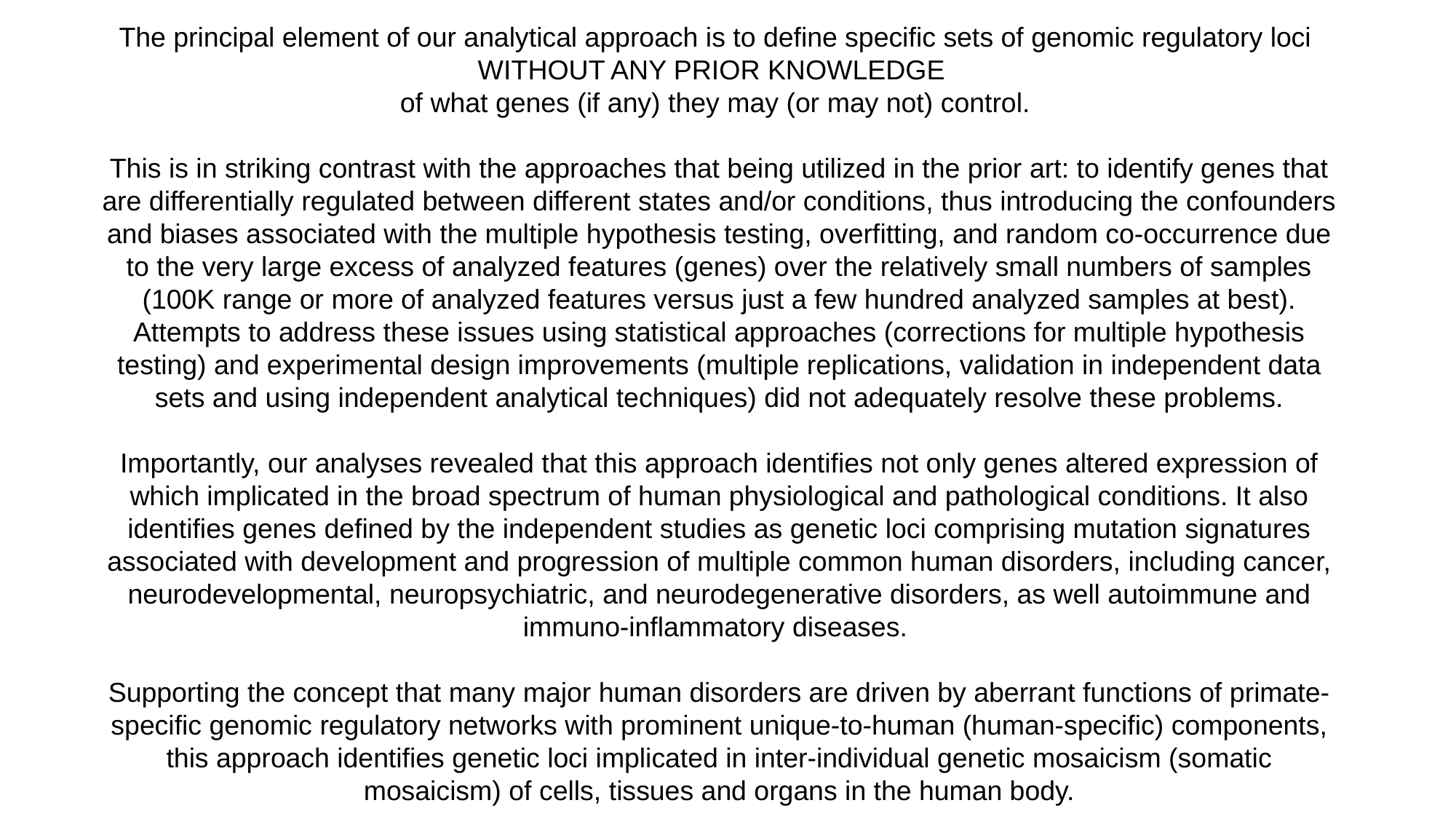

The principal element of our analytical approach is to define specific sets of genomic regulatory loci
WITHOUT ANY PRIOR KNOWLEDGE
of what genes (if any) they may (or may not) control.
This is in striking contrast with the approaches that being utilized in the prior art: to identify genes that are differentially regulated between different states and/or conditions, thus introducing the confounders and biases associated with the multiple hypothesis testing, overfitting, and random co-occurrence due to the very large excess of analyzed features (genes) over the relatively small numbers of samples (100K range or more of analyzed features versus just a few hundred analyzed samples at best). Attempts to address these issues using statistical approaches (corrections for multiple hypothesis testing) and experimental design improvements (multiple replications, validation in independent data sets and using independent analytical techniques) did not adequately resolve these problems.
Importantly, our analyses revealed that this approach identifies not only genes altered expression of which implicated in the broad spectrum of human physiological and pathological conditions. It also identifies genes defined by the independent studies as genetic loci comprising mutation signatures associated with development and progression of multiple common human disorders, including cancer, neurodevelopmental, neuropsychiatric, and neurodegenerative disorders, as well autoimmune and immuno-inflammatory diseases.
Supporting the concept that many major human disorders are driven by aberrant functions of primate-specific genomic regulatory networks with prominent unique-to-human (human-specific) components, this approach identifies genetic loci implicated in inter-individual genetic mosaicism (somatic mosaicism) of cells, tissues and organs in the human body.

## Slide 6
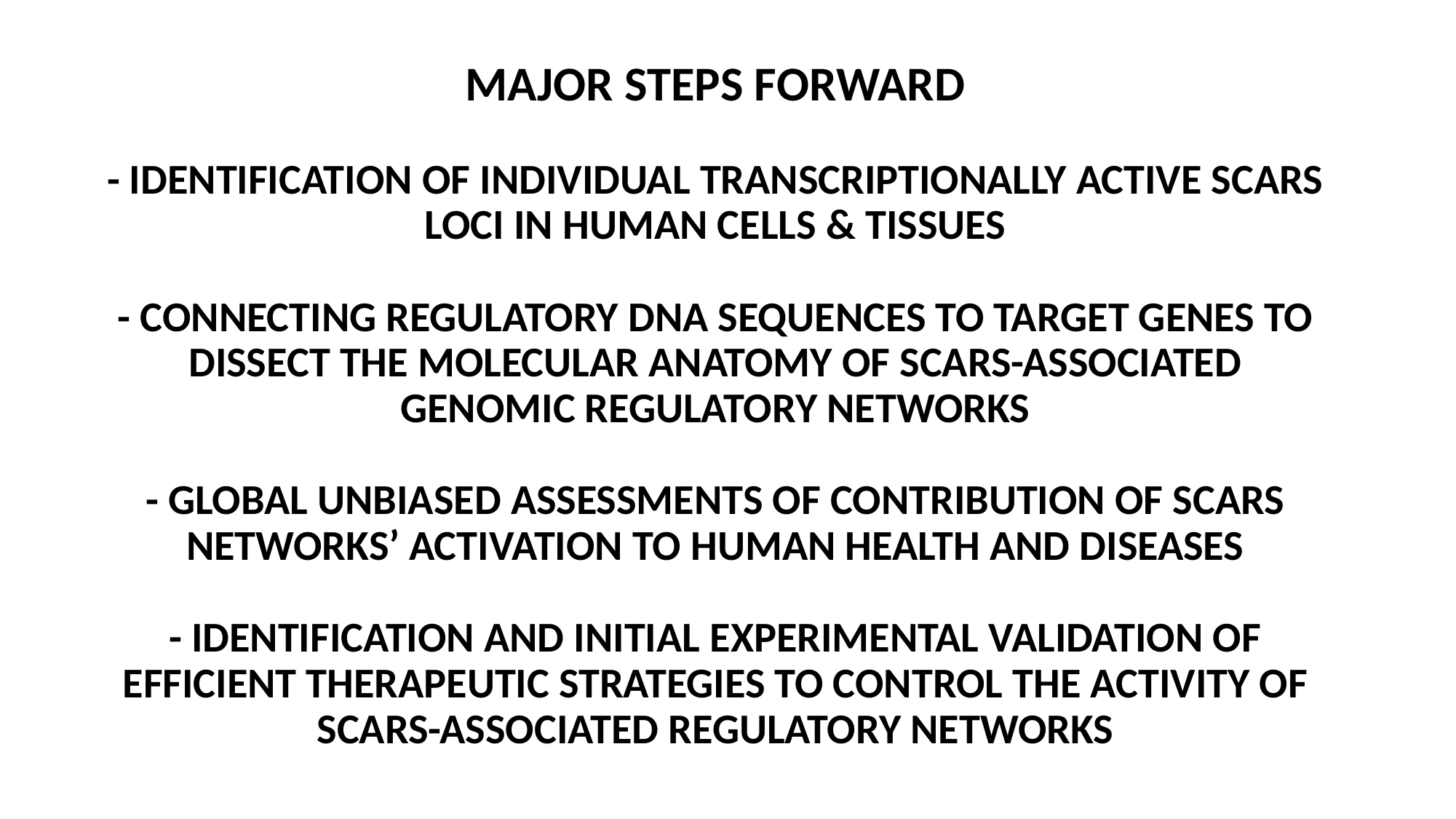

# MAJOR STEPS FORWARD - IDENTIFICATION OF INDIVIDUAL TRANSCRIPTIONALLY ACTIVE SCARS LOCI IN HUMAN CELLS & TISSUES - CONNECTING REGULATORY DNA SEQUENCES TO TARGET GENES TO DISSECT THE MOLECULAR ANATOMY OF SCARS-ASSOCIATED GENOMIC REGULATORY NETWORKS - GLOBAL UNBIASED ASSESSMENTS OF CONTRIBUTION OF SCARS NETWORKS’ ACTIVATION TO HUMAN HEALTH AND DISEASES - IDENTIFICATION AND INITIAL EXPERIMENTAL VALIDATION OF EFFICIENT THERAPEUTIC STRATEGIES TO CONTROL THE ACTIVITY OF SCARS-ASSOCIATED REGULATORY NETWORKS

## Slide 7
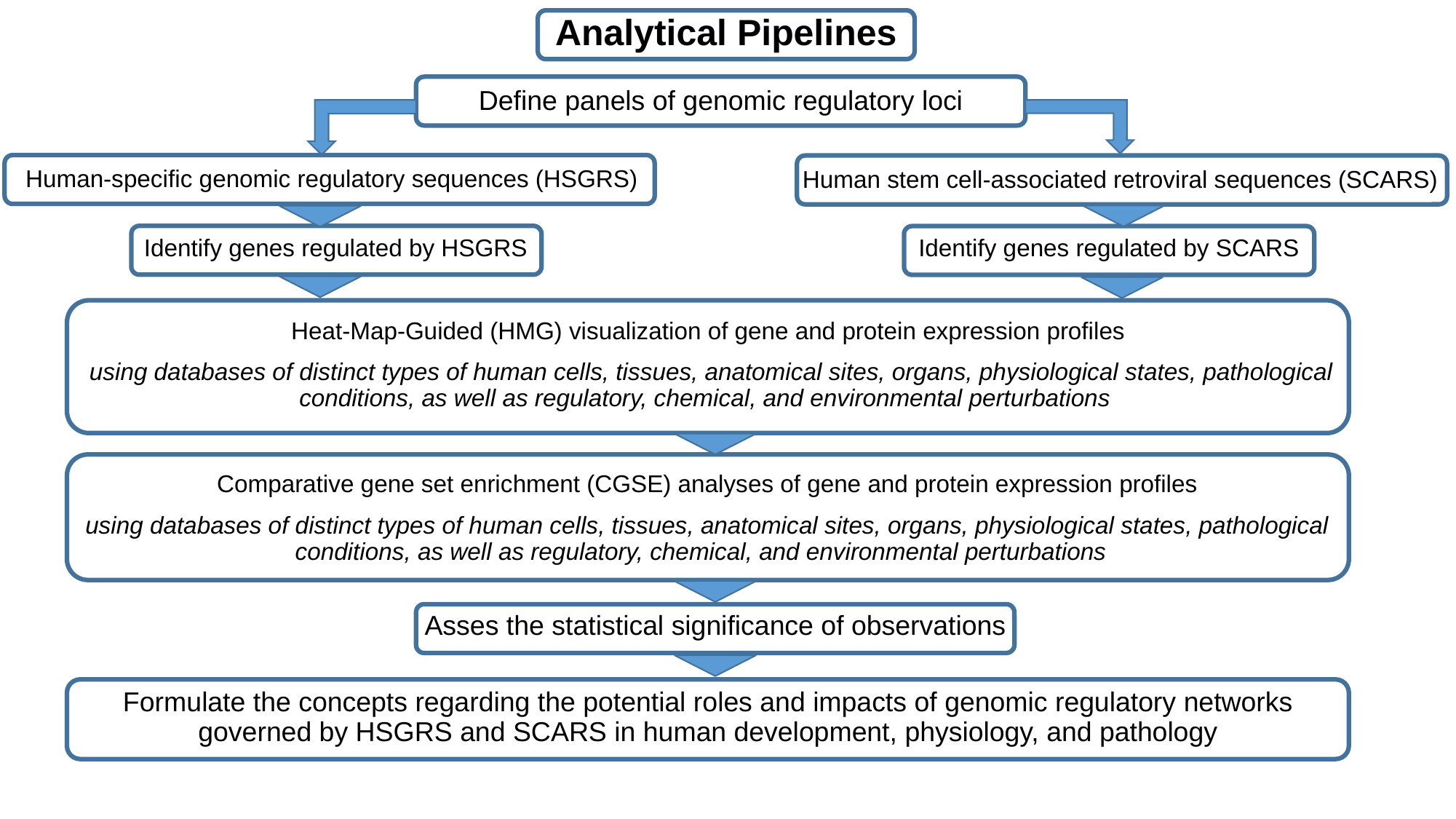

# Analytical Pipelines
Define panels of genomic regulatory loci
Human-specific genomic regulatory sequences (HSGRS)
Human stem cell-associated retroviral sequences (SCARS)
Identify genes regulated by HSGRS
Identify genes regulated by SCARS
Heat-Map-Guided (HMG) visualization of gene and protein expression profiles
using databases of distinct types of human cells, tissues, anatomical sites, organs, physiological states, pathological conditions, as well as regulatory, chemical, and environmental perturbations
Comparative gene set enrichment (CGSE) analyses of gene and protein expression profiles
using databases of distinct types of human cells, tissues, anatomical sites, organs, physiological states, pathological conditions, as well as regulatory, chemical, and environmental perturbations
Asses the statistical significance of observations
Formulate the concepts regarding the potential roles and impacts of genomic regulatory networks governed by HSGRS and SCARS in human development, physiology, and pathology

## Slide 8
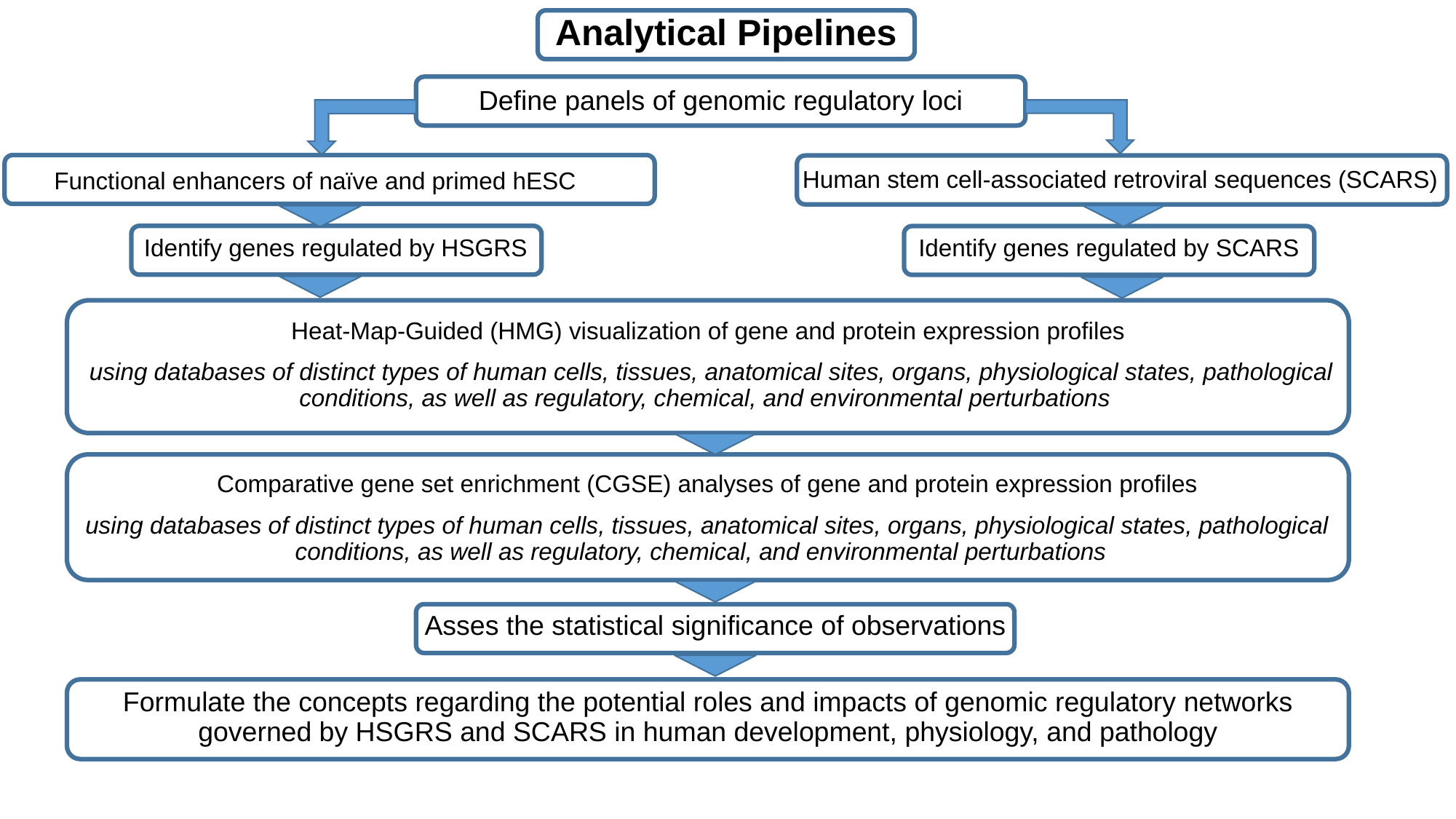

# Analytical Pipelines
Define panels of genomic regulatory loci
Human stem cell-associated retroviral sequences (SCARS)
Functional enhancers of naïve and primed hESC
Identify genes regulated by HSGRS
Identify genes regulated by SCARS
Heat-Map-Guided (HMG) visualization of gene and protein expression profiles
using databases of distinct types of human cells, tissues, anatomical sites, organs, physiological states, pathological conditions, as well as regulatory, chemical, and environmental perturbations
Comparative gene set enrichment (CGSE) analyses of gene and protein expression profiles
using databases of distinct types of human cells, tissues, anatomical sites, organs, physiological states, pathological conditions, as well as regulatory, chemical, and environmental perturbations
Asses the statistical significance of observations
Formulate the concepts regarding the potential roles and impacts of genomic regulatory networks governed by HSGRS and SCARS in human development, physiology, and pathology

## Slide 9
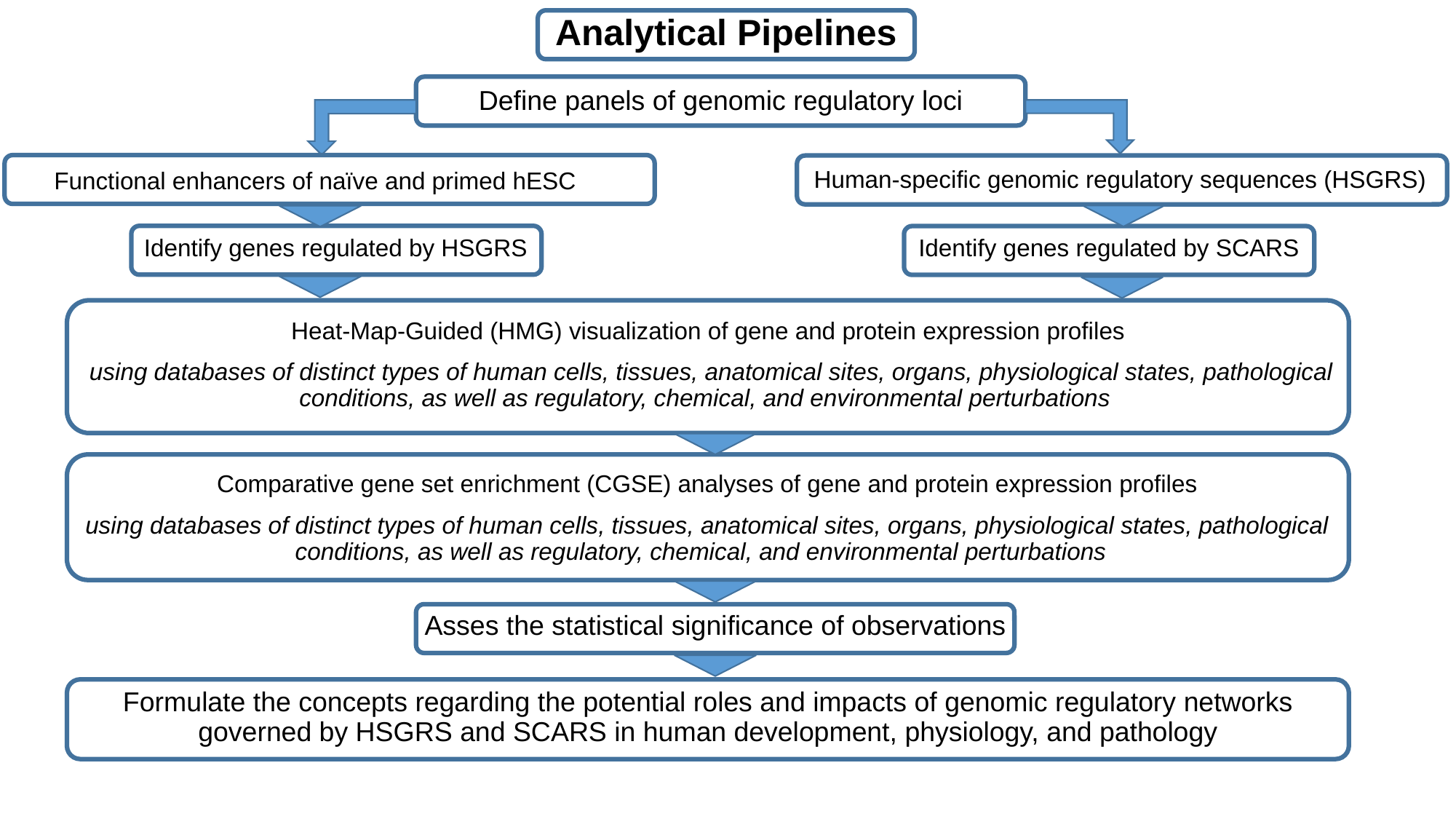

# Analytical Pipelines
Define panels of genomic regulatory loci
Human-specific genomic regulatory sequences (HSGRS)
Functional enhancers of naïve and primed hESC
Identify genes regulated by HSGRS
Identify genes regulated by SCARS
Heat-Map-Guided (HMG) visualization of gene and protein expression profiles
using databases of distinct types of human cells, tissues, anatomical sites, organs, physiological states, pathological conditions, as well as regulatory, chemical, and environmental perturbations
Comparative gene set enrichment (CGSE) analyses of gene and protein expression profiles
using databases of distinct types of human cells, tissues, anatomical sites, organs, physiological states, pathological conditions, as well as regulatory, chemical, and environmental perturbations
Asses the statistical significance of observations
Formulate the concepts regarding the potential roles and impacts of genomic regulatory networks governed by HSGRS and SCARS in human development, physiology, and pathology

## Slide 10
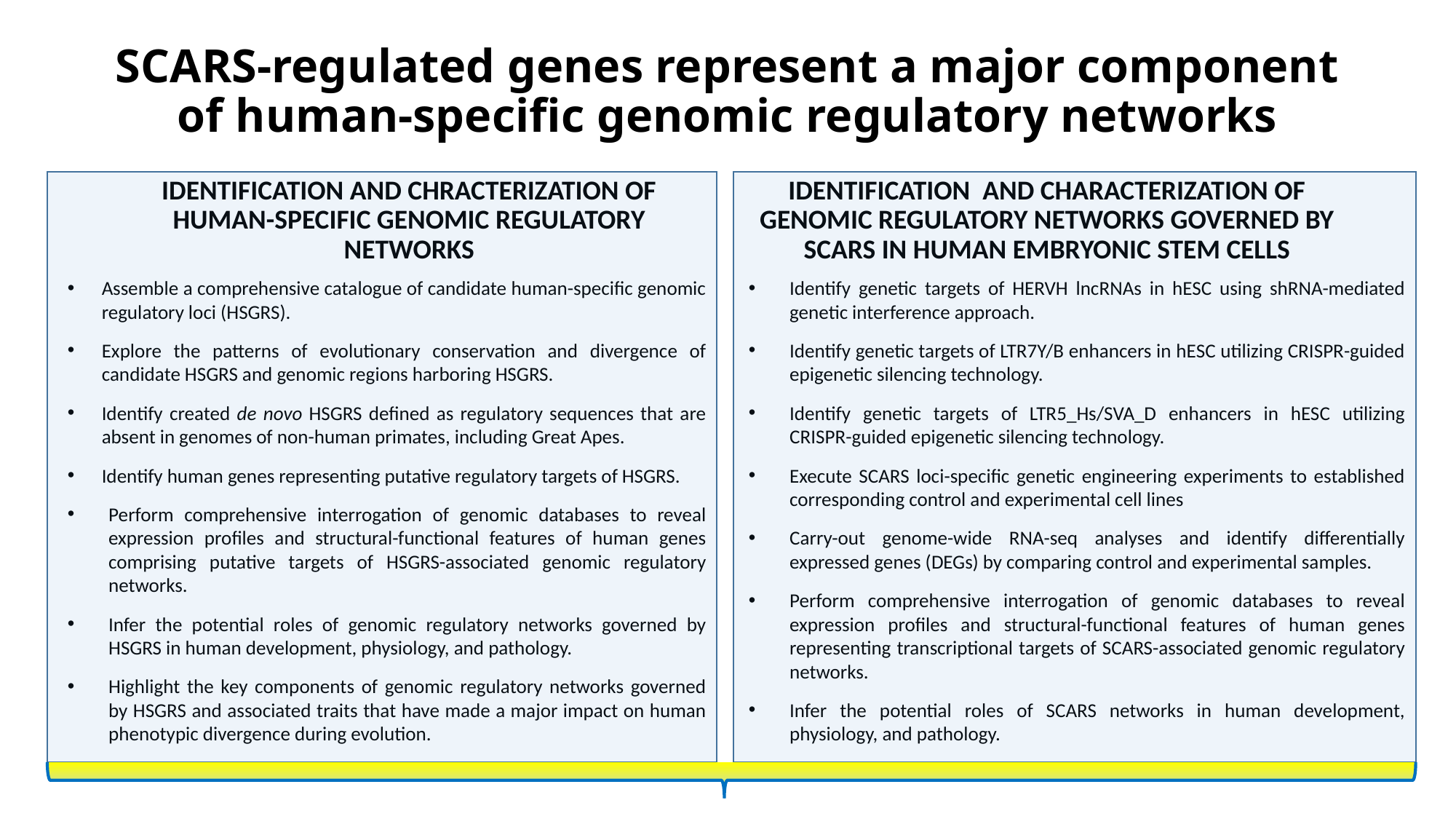

# SCARS-regulated genes represent a major component of human-specific genomic regulatory networks
IDENTIFICATION AND CHRACTERIZATION OF HUMAN-SPECIFIC GENOMIC REGULATORY NETWORKS
IDENTIFICATION AND CHARACTERIZATION OF GENOMIC REGULATORY NETWORKS GOVERNED BY SCARS IN HUMAN EMBRYONIC STEM CELLS
Assemble a comprehensive catalogue of candidate human-specific genomic regulatory loci (HSGRS).
Explore the patterns of evolutionary conservation and divergence of candidate HSGRS and genomic regions harboring HSGRS.
Identify created de novo HSGRS defined as regulatory sequences that are absent in genomes of non-human primates, including Great Apes.
Identify human genes representing putative regulatory targets of HSGRS.
Perform comprehensive interrogation of genomic databases to reveal expression profiles and structural-functional features of human genes comprising putative targets of HSGRS-associated genomic regulatory networks.
Infer the potential roles of genomic regulatory networks governed by HSGRS in human development, physiology, and pathology.
Highlight the key components of genomic regulatory networks governed by HSGRS and associated traits that have made a major impact on human phenotypic divergence during evolution.
Identify genetic targets of HERVH lncRNAs in hESC using shRNA-mediated genetic interference approach.
Identify genetic targets of LTR7Y/B enhancers in hESC utilizing CRISPR-guided epigenetic silencing technology.
Identify genetic targets of LTR5_Hs/SVA_D enhancers in hESC utilizing CRISPR-guided epigenetic silencing technology.
Execute SCARS loci-specific genetic engineering experiments to established corresponding control and experimental cell lines
Carry-out genome-wide RNA-seq analyses and identify differentially expressed genes (DEGs) by comparing control and experimental samples.
Perform comprehensive interrogation of genomic databases to reveal expression profiles and structural-functional features of human genes representing transcriptional targets of SCARS-associated genomic regulatory networks.
Infer the potential roles of SCARS networks in human development, physiology, and pathology.

## Slide 11
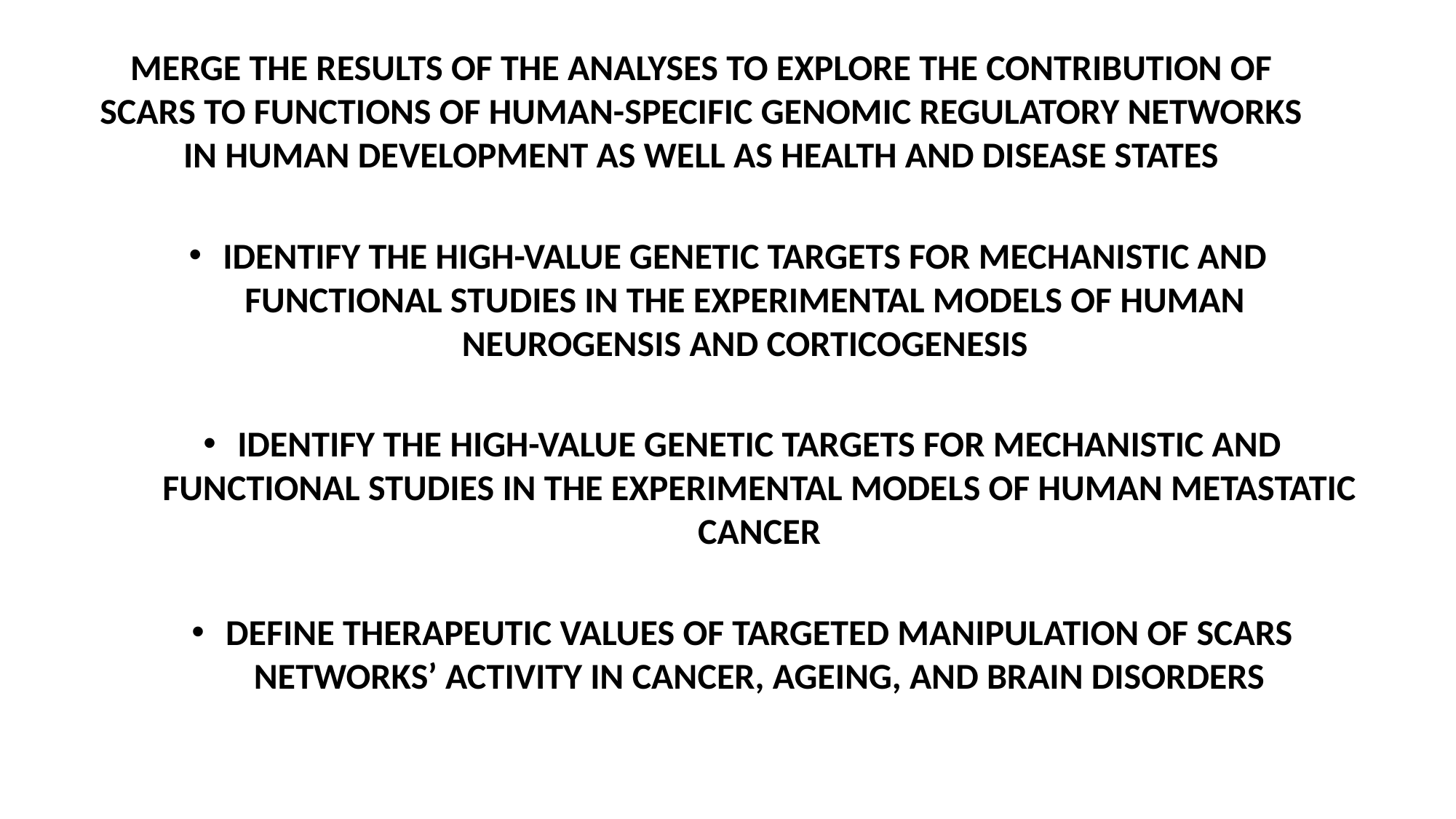

MERGE THE RESULTS OF THE ANALYSES TO EXPLORE THE CONTRIBUTION OF SCARS TO FUNCTIONS OF HUMAN-SPECIFIC GENOMIC REGULATORY NETWORKS IN HUMAN DEVELOPMENT AS WELL AS HEALTH AND DISEASE STATES
IDENTIFY THE HIGH-VALUE GENETIC TARGETS FOR MECHANISTIC AND FUNCTIONAL STUDIES IN THE EXPERIMENTAL MODELS OF HUMAN NEUROGENSIS AND CORTICOGENESIS
IDENTIFY THE HIGH-VALUE GENETIC TARGETS FOR MECHANISTIC AND FUNCTIONAL STUDIES IN THE EXPERIMENTAL MODELS OF HUMAN METASTATIC CANCER
DEFINE THERAPEUTIC VALUES OF TARGETED MANIPULATION OF SCARS NETWORKS’ ACTIVITY IN CANCER, AGEING, AND BRAIN DISORDERS

## Slide 12
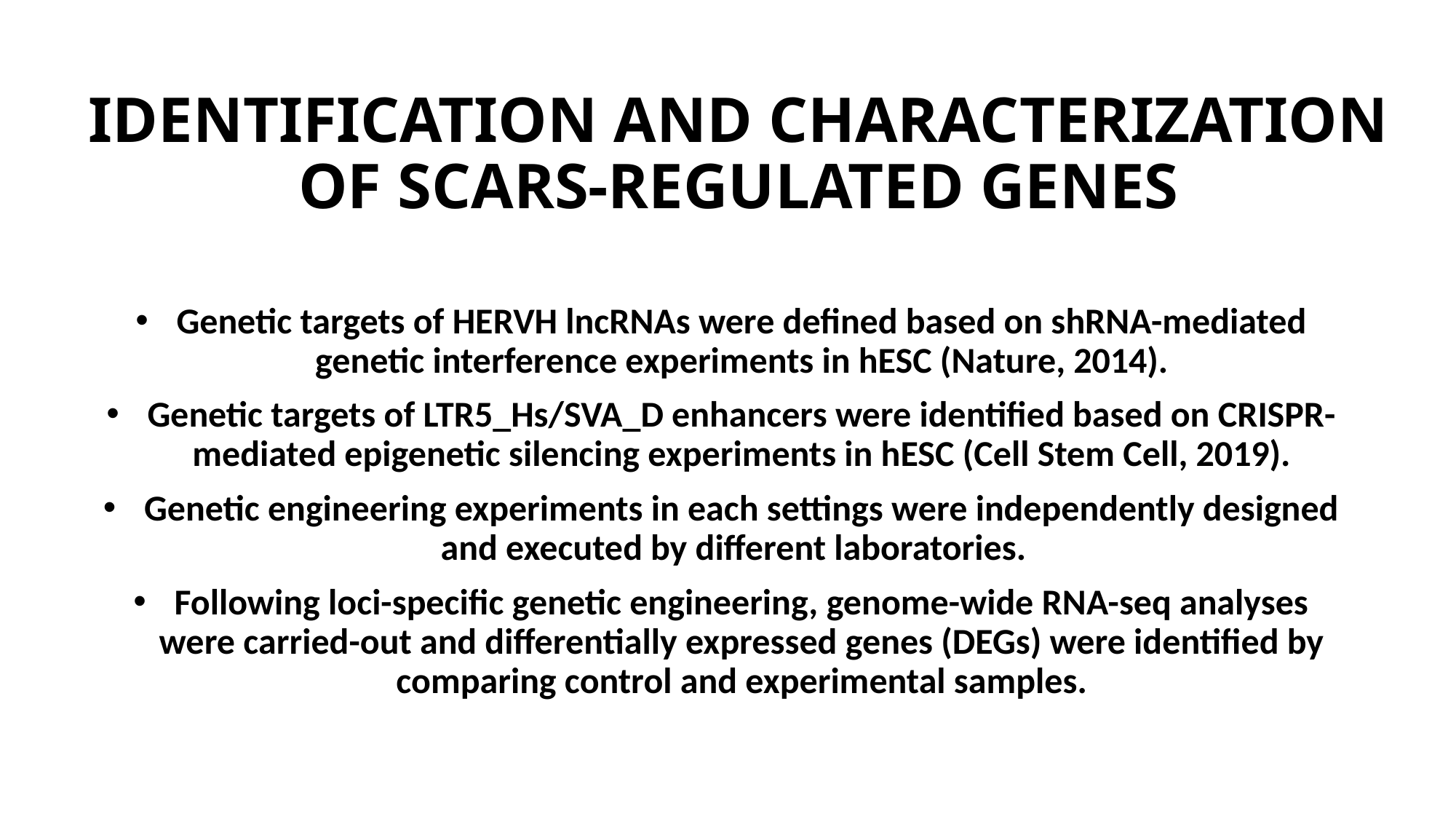

# IDENTIFICATION AND CHARACTERIZATION OF SCARS-REGULATED GENES
Genetic targets of HERVH lncRNAs were defined based on shRNA-mediated genetic interference experiments in hESC (Nature, 2014).
Genetic targets of LTR5_Hs/SVA_D enhancers were identified based on CRISPR-mediated epigenetic silencing experiments in hESC (Cell Stem Cell, 2019).
Genetic engineering experiments in each settings were independently designed and executed by different laboratories.
Following loci-specific genetic engineering, genome-wide RNA-seq analyses were carried-out and differentially expressed genes (DEGs) were identified by comparing control and experimental samples.

## Slide 13
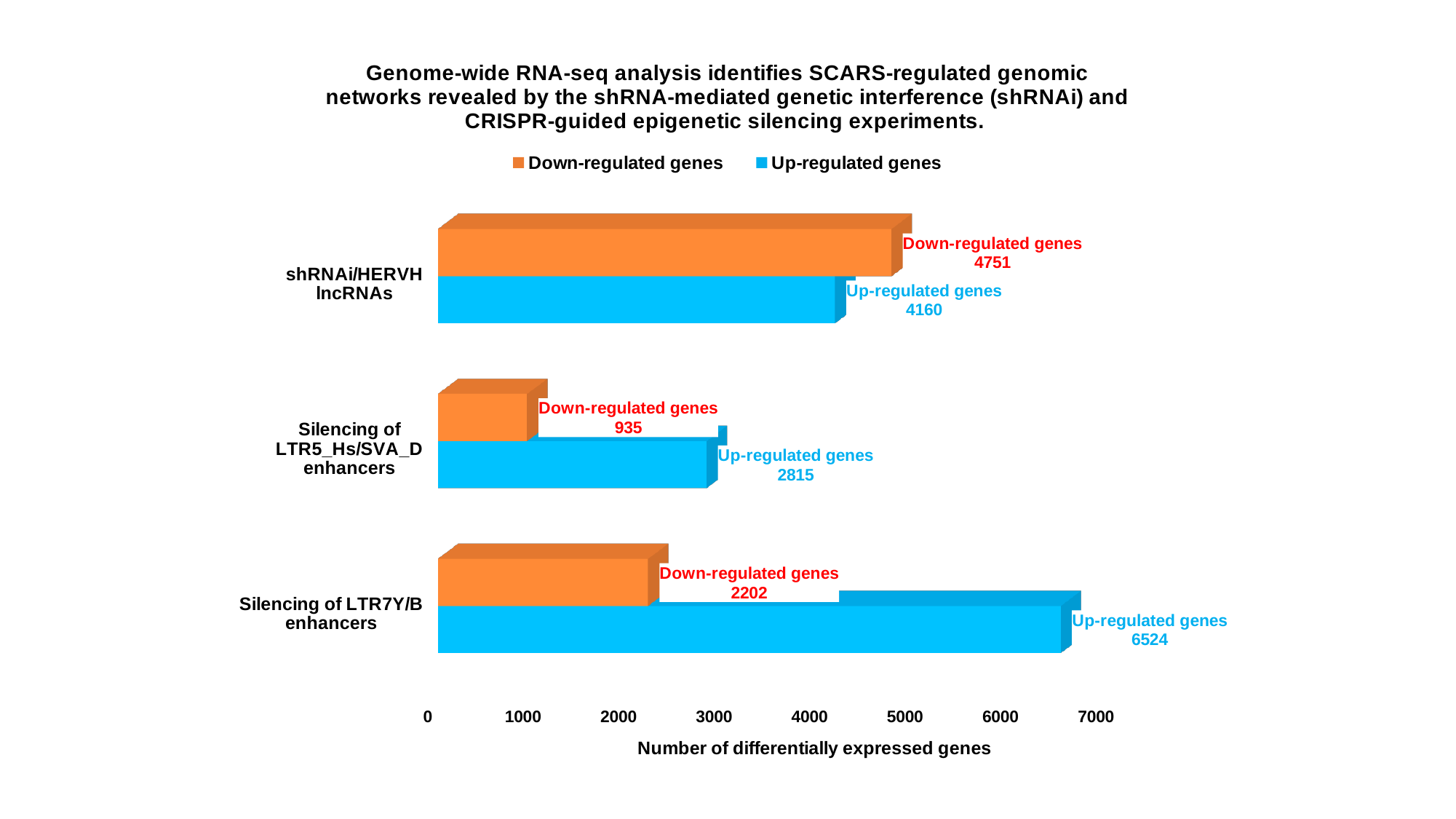

[unsupported chart]

## Slide 14
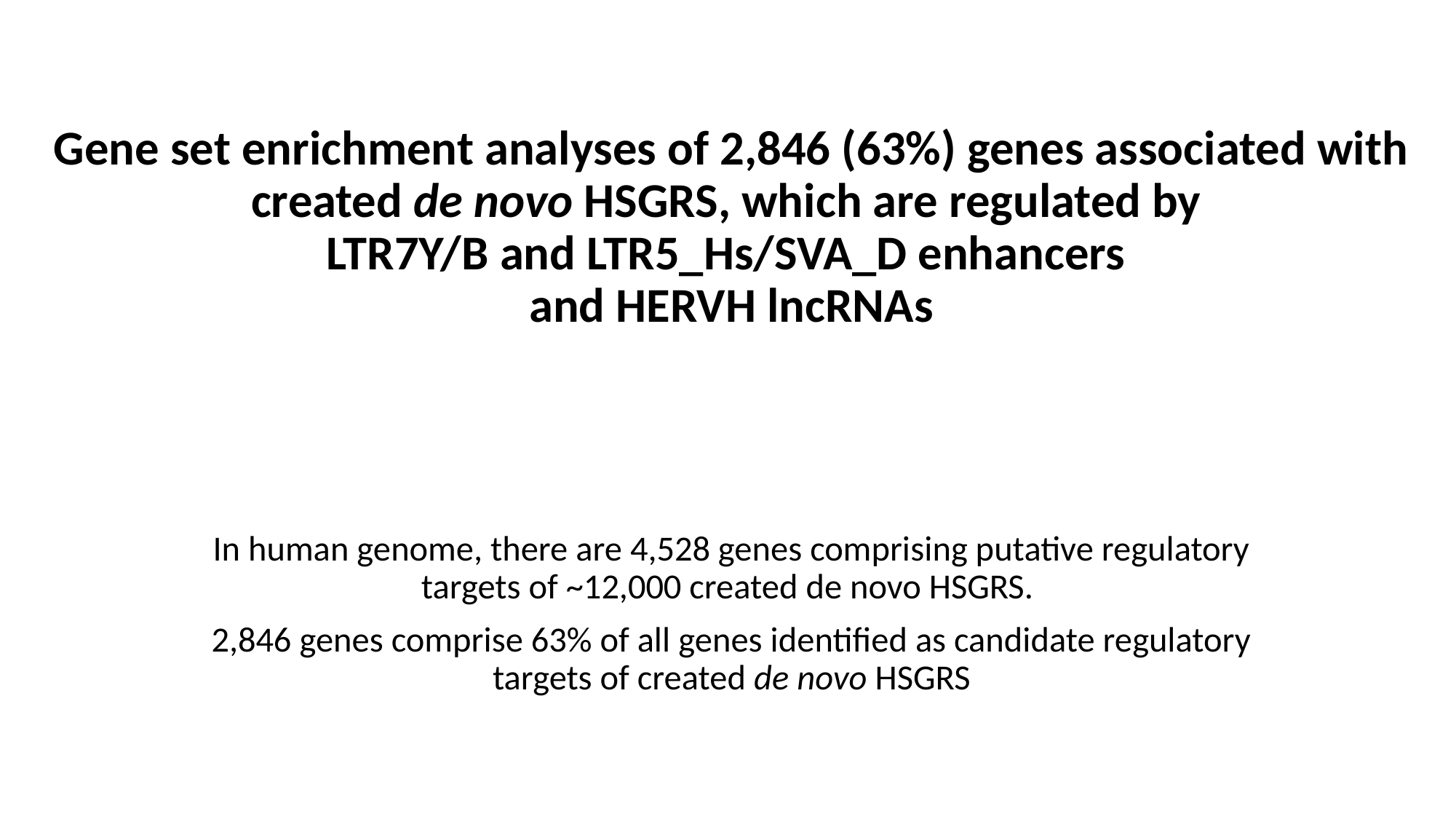

# Gene set enrichment analyses of 2,846 (63%) genes associated with created de novo HSGRS, which are regulated by LTR7Y/B and LTR5_Hs/SVA_D enhancers and HERVH lncRNAs
In human genome, there are 4,528 genes comprising putative regulatory targets of ~12,000 created de novo HSGRS.
2,846 genes comprise 63% of all genes identified as candidate regulatory targets of created de novo HSGRS

## Slide 15
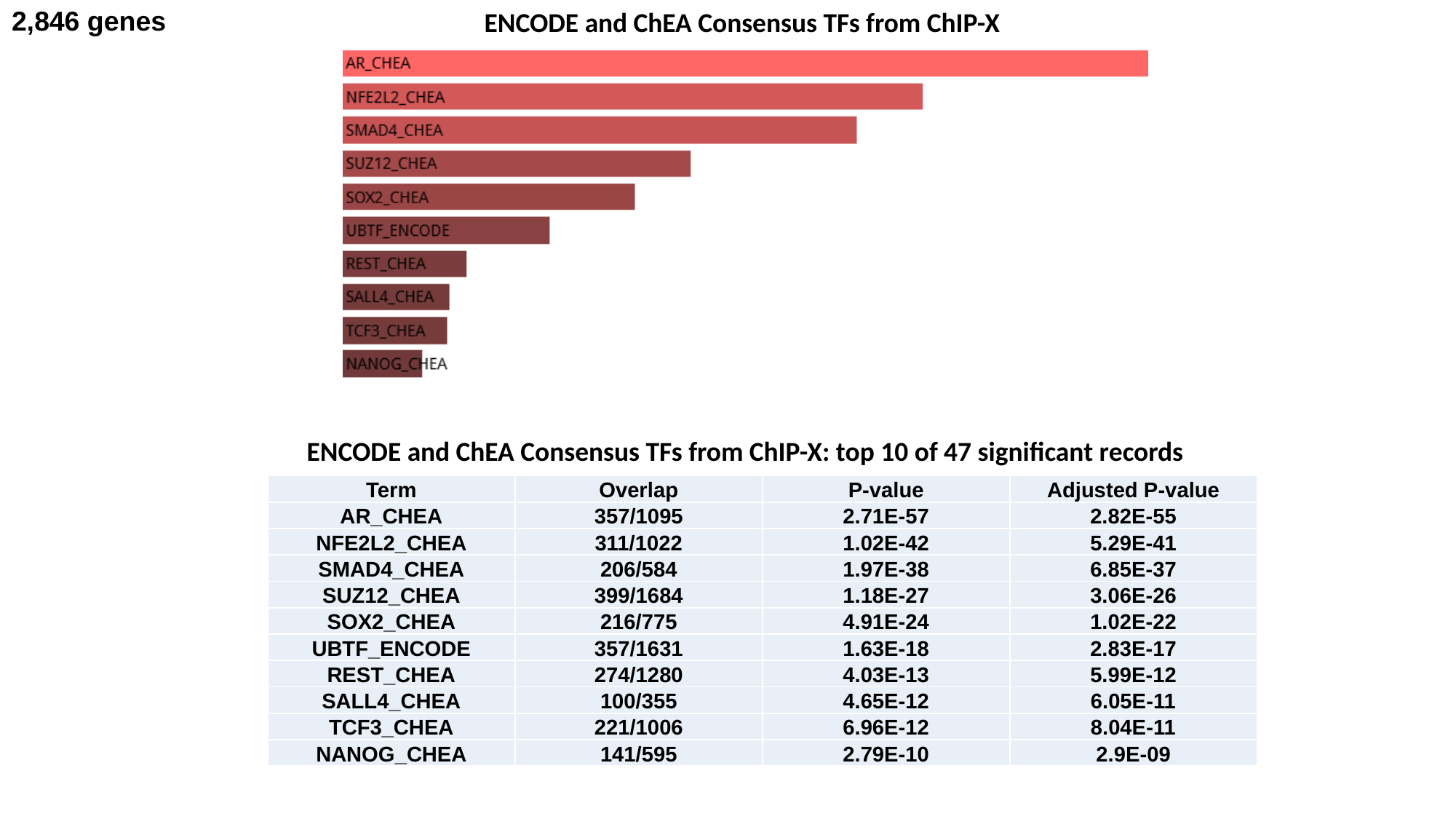

ENCODE and ChEA Consensus TFs from ChIP-X
2,846 genes
ENCODE and ChEA Consensus TFs from ChIP-X: top 10 of 47 significant records
| Term | Overlap | P-value | Adjusted P-value |
| --- | --- | --- | --- |
| AR\_CHEA | 357/1095 | 2.71E-57 | 2.82E-55 |
| NFE2L2\_CHEA | 311/1022 | 1.02E-42 | 5.29E-41 |
| SMAD4\_CHEA | 206/584 | 1.97E-38 | 6.85E-37 |
| SUZ12\_CHEA | 399/1684 | 1.18E-27 | 3.06E-26 |
| SOX2\_CHEA | 216/775 | 4.91E-24 | 1.02E-22 |
| UBTF\_ENCODE | 357/1631 | 1.63E-18 | 2.83E-17 |
| REST\_CHEA | 274/1280 | 4.03E-13 | 5.99E-12 |
| SALL4\_CHEA | 100/355 | 4.65E-12 | 6.05E-11 |
| TCF3\_CHEA | 221/1006 | 6.96E-12 | 8.04E-11 |
| NANOG\_CHEA | 141/595 | 2.79E-10 | 2.9E-09 |

## Slide 16
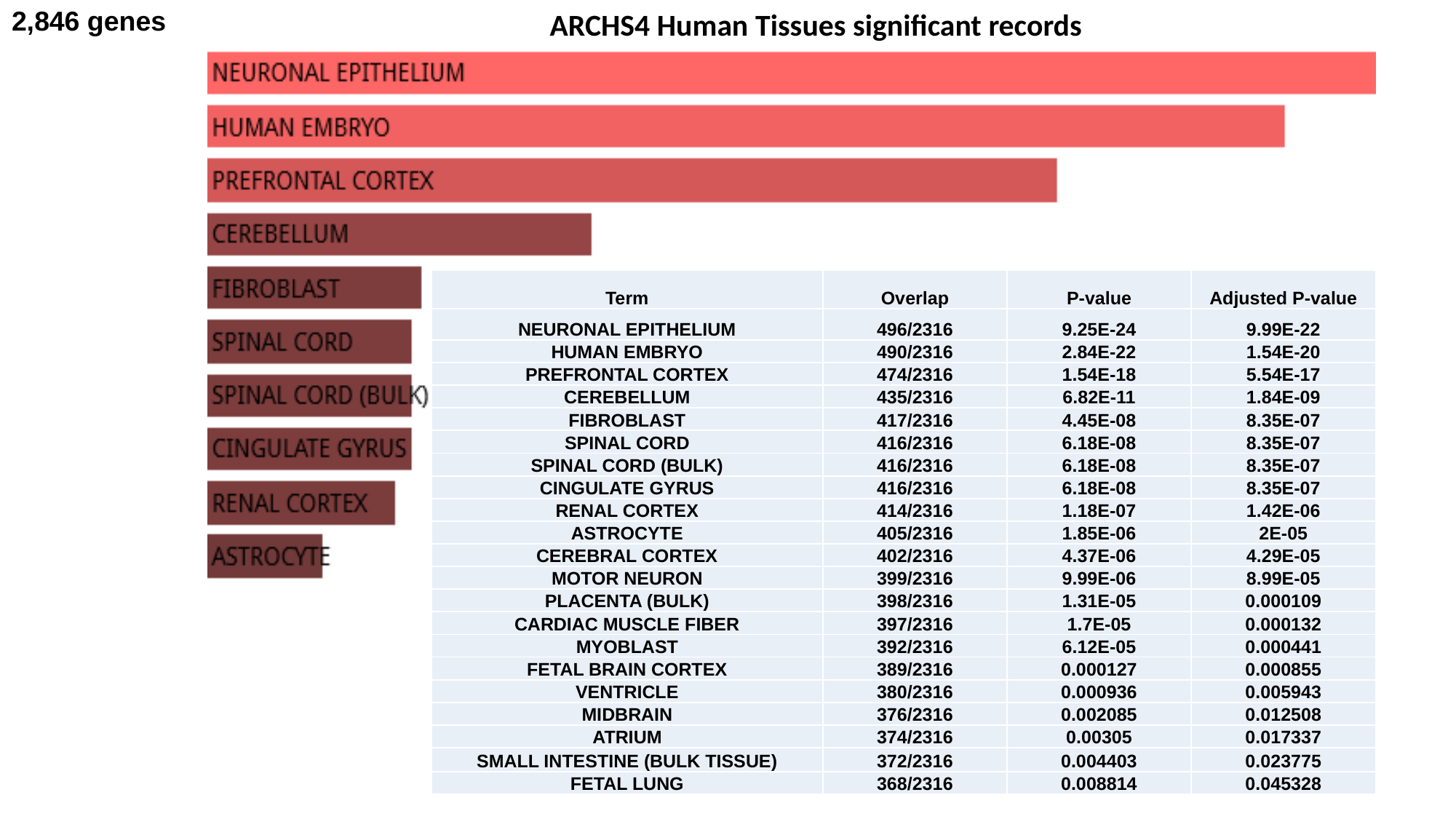

ARCHS4 Human Tissues significant records
2,846 genes
| Term | Overlap | P-value | Adjusted P-value |
| --- | --- | --- | --- |
| NEURONAL EPITHELIUM | 496/2316 | 9.25E-24 | 9.99E-22 |
| HUMAN EMBRYO | 490/2316 | 2.84E-22 | 1.54E-20 |
| PREFRONTAL CORTEX | 474/2316 | 1.54E-18 | 5.54E-17 |
| CEREBELLUM | 435/2316 | 6.82E-11 | 1.84E-09 |
| FIBROBLAST | 417/2316 | 4.45E-08 | 8.35E-07 |
| SPINAL CORD | 416/2316 | 6.18E-08 | 8.35E-07 |
| SPINAL CORD (BULK) | 416/2316 | 6.18E-08 | 8.35E-07 |
| CINGULATE GYRUS | 416/2316 | 6.18E-08 | 8.35E-07 |
| RENAL CORTEX | 414/2316 | 1.18E-07 | 1.42E-06 |
| ASTROCYTE | 405/2316 | 1.85E-06 | 2E-05 |
| CEREBRAL CORTEX | 402/2316 | 4.37E-06 | 4.29E-05 |
| MOTOR NEURON | 399/2316 | 9.99E-06 | 8.99E-05 |
| PLACENTA (BULK) | 398/2316 | 1.31E-05 | 0.000109 |
| CARDIAC MUSCLE FIBER | 397/2316 | 1.7E-05 | 0.000132 |
| MYOBLAST | 392/2316 | 6.12E-05 | 0.000441 |
| FETAL BRAIN CORTEX | 389/2316 | 0.000127 | 0.000855 |
| VENTRICLE | 380/2316 | 0.000936 | 0.005943 |
| MIDBRAIN | 376/2316 | 0.002085 | 0.012508 |
| ATRIUM | 374/2316 | 0.00305 | 0.017337 |
| SMALL INTESTINE (BULK TISSUE) | 372/2316 | 0.004403 | 0.023775 |
| FETAL LUNG | 368/2316 | 0.008814 | 0.045328 |

## Slide 17
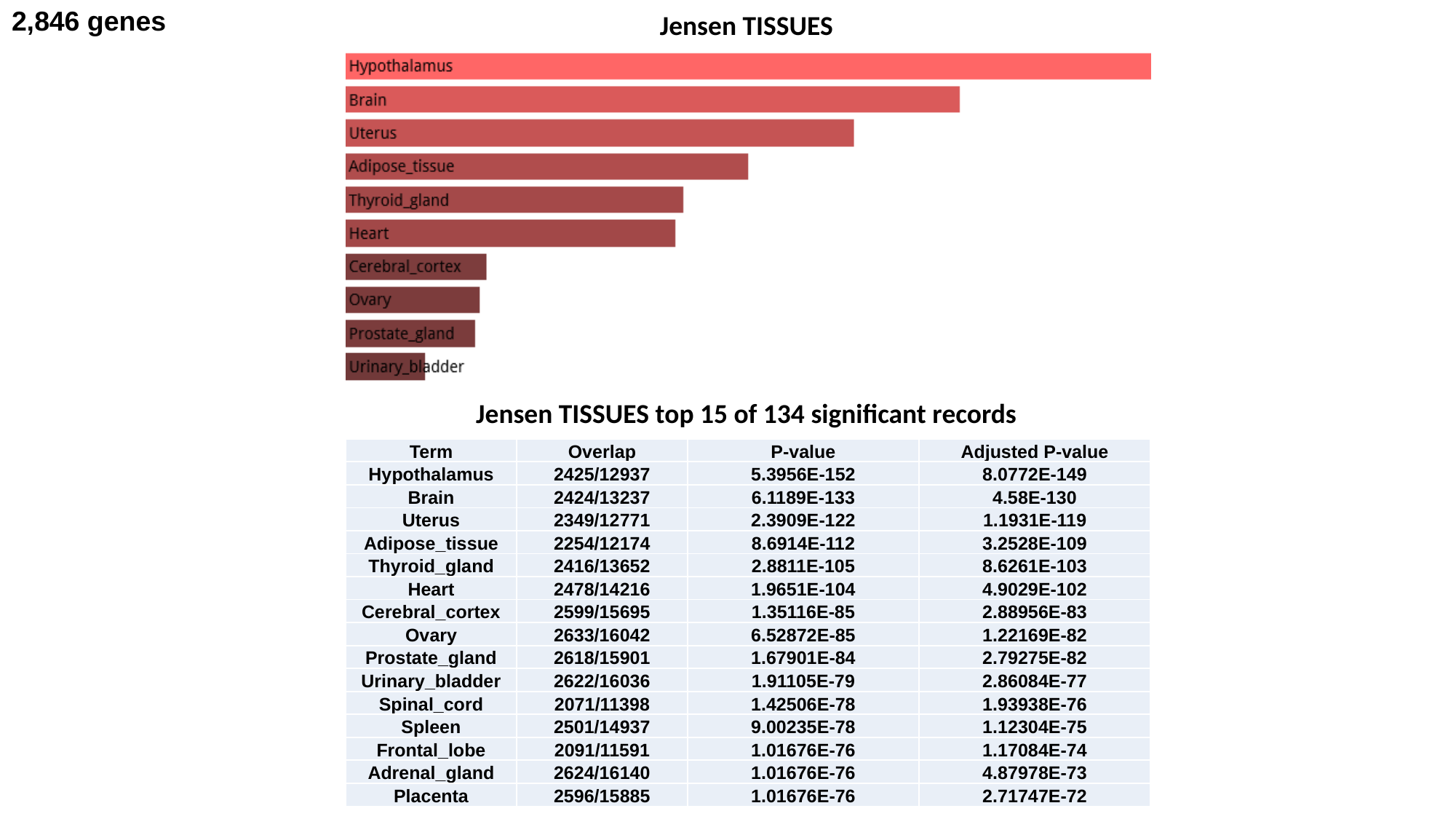

2,846 genes
Jensen TISSUES
Jensen TISSUES top 15 of 134 significant records
| Term | Overlap | P-value | Adjusted P-value |
| --- | --- | --- | --- |
| Hypothalamus | 2425/12937 | 5.3956E-152 | 8.0772E-149 |
| Brain | 2424/13237 | 6.1189E-133 | 4.58E-130 |
| Uterus | 2349/12771 | 2.3909E-122 | 1.1931E-119 |
| Adipose\_tissue | 2254/12174 | 8.6914E-112 | 3.2528E-109 |
| Thyroid\_gland | 2416/13652 | 2.8811E-105 | 8.6261E-103 |
| Heart | 2478/14216 | 1.9651E-104 | 4.9029E-102 |
| Cerebral\_cortex | 2599/15695 | 1.35116E-85 | 2.88956E-83 |
| Ovary | 2633/16042 | 6.52872E-85 | 1.22169E-82 |
| Prostate\_gland | 2618/15901 | 1.67901E-84 | 2.79275E-82 |
| Urinary\_bladder | 2622/16036 | 1.91105E-79 | 2.86084E-77 |
| Spinal\_cord | 2071/11398 | 1.42506E-78 | 1.93938E-76 |
| Spleen | 2501/14937 | 9.00235E-78 | 1.12304E-75 |
| Frontal\_lobe | 2091/11591 | 1.01676E-76 | 1.17084E-74 |
| Adrenal\_gland | 2624/16140 | 1.01676E-76 | 4.87978E-73 |
| Placenta | 2596/15885 | 1.01676E-76 | 2.71747E-72 |

## Slide 18
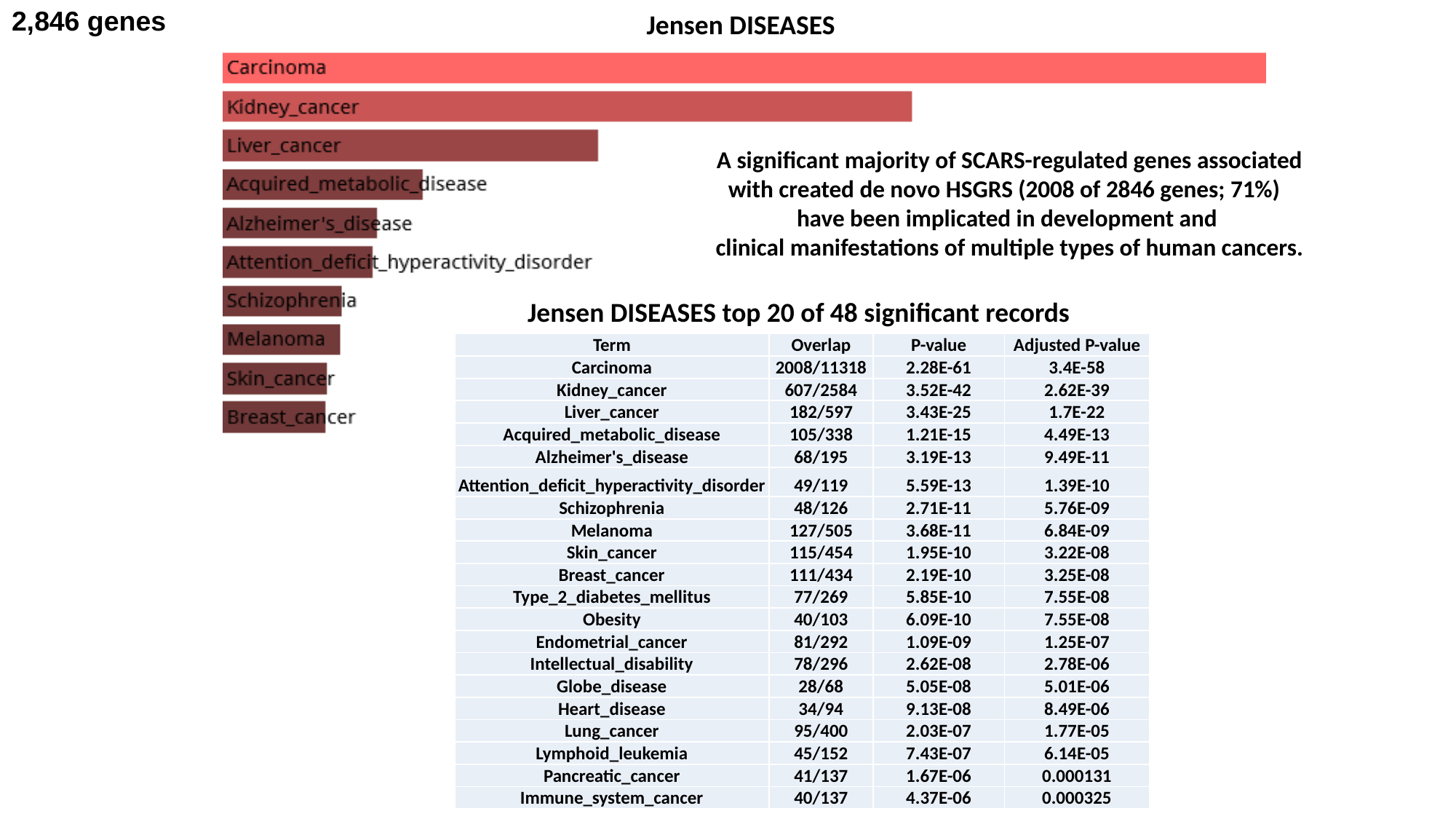

2,846 genes
Jensen DISEASES
A significant majority of SCARS-regulated genes associated with created de novo HSGRS (2008 of 2846 genes; 71%)
have been implicated in development and
clinical manifestations of multiple types of human cancers.
Jensen DISEASES top 20 of 48 significant records
| Term | Overlap | P-value | Adjusted P-value |
| --- | --- | --- | --- |
| Carcinoma | 2008/11318 | 2.28E-61 | 3.4E-58 |
| Kidney\_cancer | 607/2584 | 3.52E-42 | 2.62E-39 |
| Liver\_cancer | 182/597 | 3.43E-25 | 1.7E-22 |
| Acquired\_metabolic\_disease | 105/338 | 1.21E-15 | 4.49E-13 |
| Alzheimer's\_disease | 68/195 | 3.19E-13 | 9.49E-11 |
| Attention\_deficit\_hyperactivity\_disorder | 49/119 | 5.59E-13 | 1.39E-10 |
| Schizophrenia | 48/126 | 2.71E-11 | 5.76E-09 |
| Melanoma | 127/505 | 3.68E-11 | 6.84E-09 |
| Skin\_cancer | 115/454 | 1.95E-10 | 3.22E-08 |
| Breast\_cancer | 111/434 | 2.19E-10 | 3.25E-08 |
| Type\_2\_diabetes\_mellitus | 77/269 | 5.85E-10 | 7.55E-08 |
| Obesity | 40/103 | 6.09E-10 | 7.55E-08 |
| Endometrial\_cancer | 81/292 | 1.09E-09 | 1.25E-07 |
| Intellectual\_disability | 78/296 | 2.62E-08 | 2.78E-06 |
| Globe\_disease | 28/68 | 5.05E-08 | 5.01E-06 |
| Heart\_disease | 34/94 | 9.13E-08 | 8.49E-06 |
| Lung\_cancer | 95/400 | 2.03E-07 | 1.77E-05 |
| Lymphoid\_leukemia | 45/152 | 7.43E-07 | 6.14E-05 |
| Pancreatic\_cancer | 41/137 | 1.67E-06 | 0.000131 |
| Immune\_system\_cancer | 40/137 | 4.37E-06 | 0.000325 |

## Slide 19
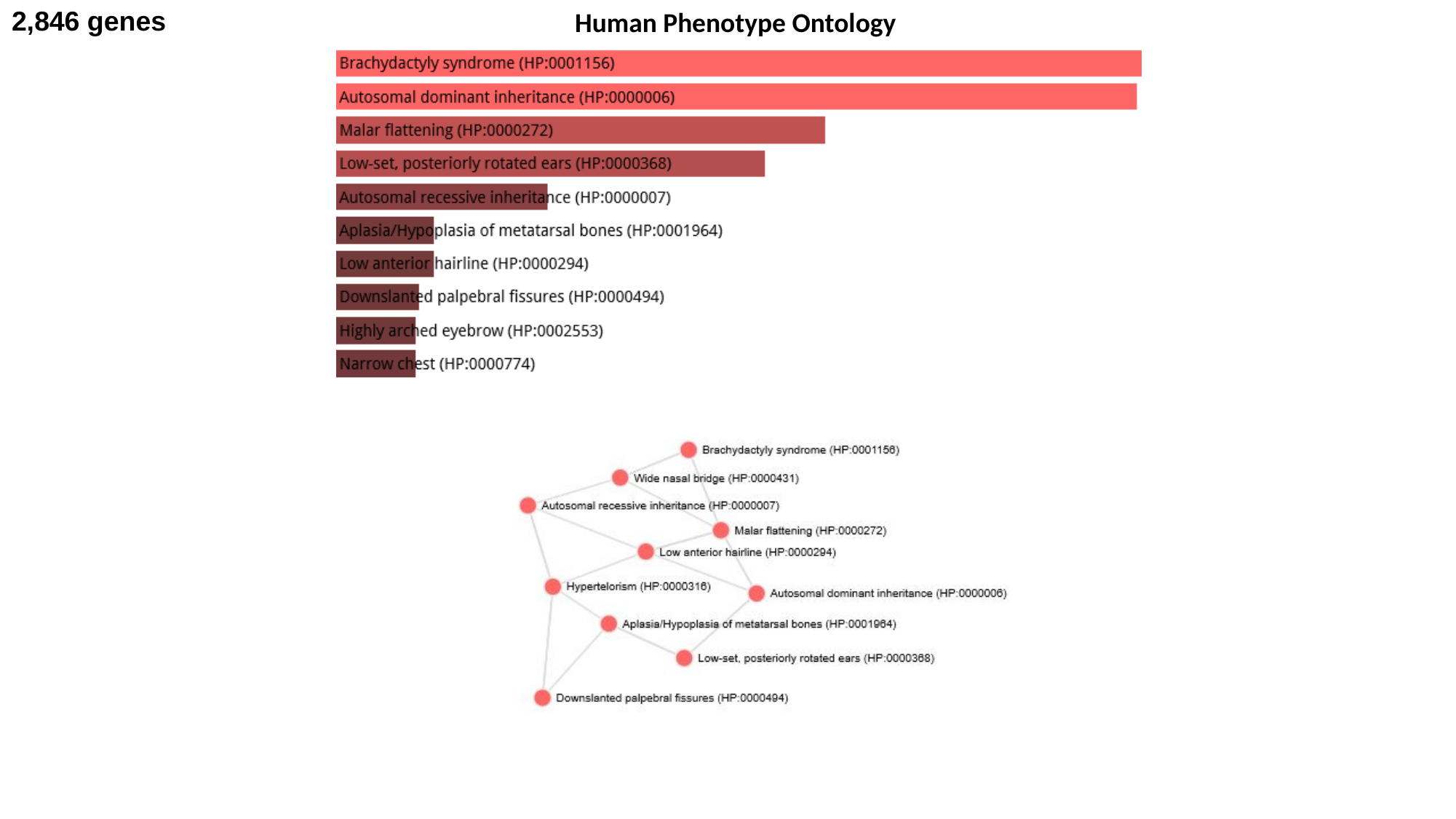

Human Phenotype Ontology
2,846 genes

## Slide 20
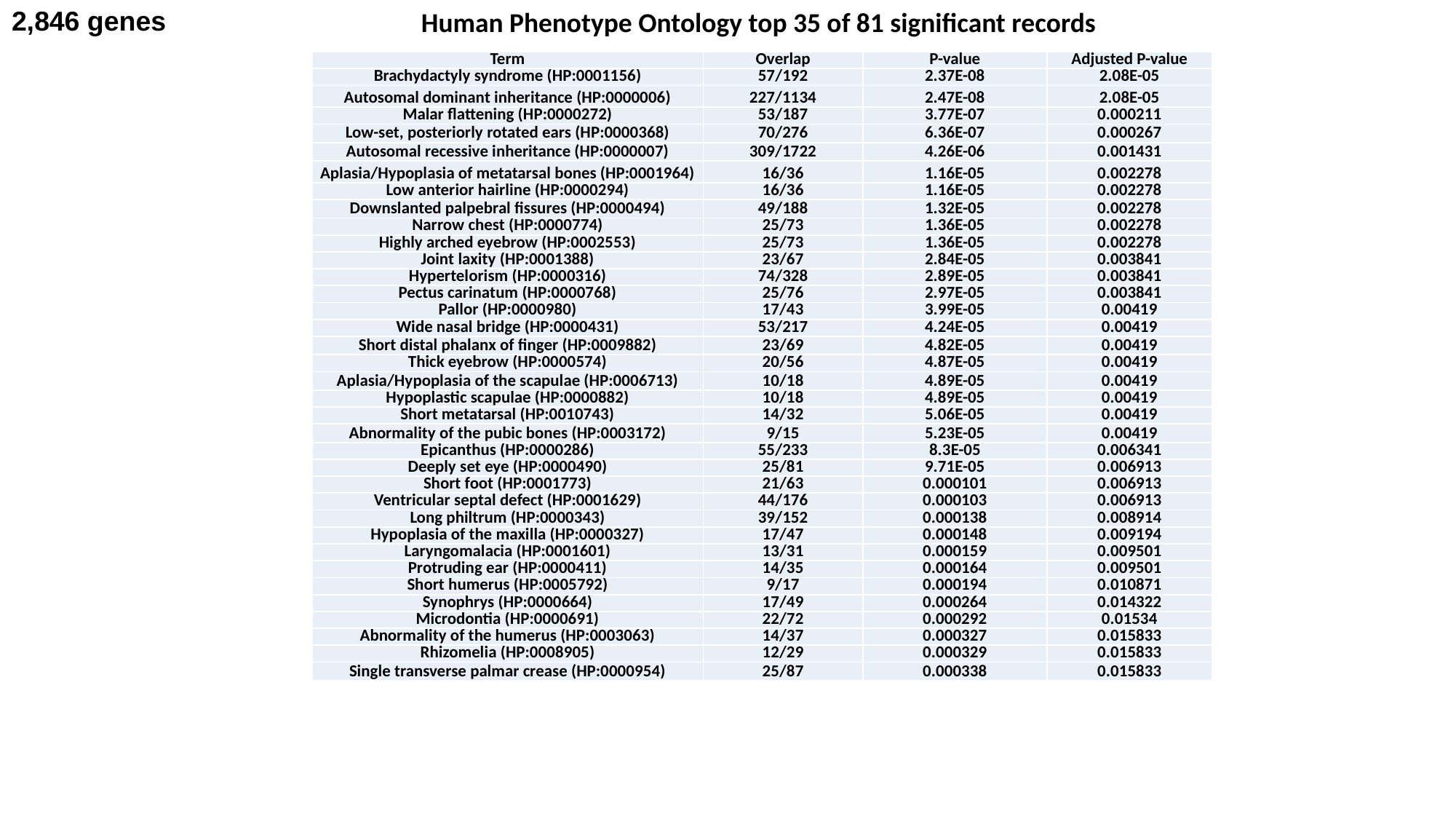

Human Phenotype Ontology top 35 of 81 significant records
2,846 genes
| Term | Overlap | P-value | Adjusted P-value |
| --- | --- | --- | --- |
| Brachydactyly syndrome (HP:0001156) | 57/192 | 2.37E-08 | 2.08E-05 |
| Autosomal dominant inheritance (HP:0000006) | 227/1134 | 2.47E-08 | 2.08E-05 |
| Malar flattening (HP:0000272) | 53/187 | 3.77E-07 | 0.000211 |
| Low-set, posteriorly rotated ears (HP:0000368) | 70/276 | 6.36E-07 | 0.000267 |
| Autosomal recessive inheritance (HP:0000007) | 309/1722 | 4.26E-06 | 0.001431 |
| Aplasia/Hypoplasia of metatarsal bones (HP:0001964) | 16/36 | 1.16E-05 | 0.002278 |
| Low anterior hairline (HP:0000294) | 16/36 | 1.16E-05 | 0.002278 |
| Downslanted palpebral fissures (HP:0000494) | 49/188 | 1.32E-05 | 0.002278 |
| Narrow chest (HP:0000774) | 25/73 | 1.36E-05 | 0.002278 |
| Highly arched eyebrow (HP:0002553) | 25/73 | 1.36E-05 | 0.002278 |
| Joint laxity (HP:0001388) | 23/67 | 2.84E-05 | 0.003841 |
| Hypertelorism (HP:0000316) | 74/328 | 2.89E-05 | 0.003841 |
| Pectus carinatum (HP:0000768) | 25/76 | 2.97E-05 | 0.003841 |
| Pallor (HP:0000980) | 17/43 | 3.99E-05 | 0.00419 |
| Wide nasal bridge (HP:0000431) | 53/217 | 4.24E-05 | 0.00419 |
| Short distal phalanx of finger (HP:0009882) | 23/69 | 4.82E-05 | 0.00419 |
| Thick eyebrow (HP:0000574) | 20/56 | 4.87E-05 | 0.00419 |
| Aplasia/Hypoplasia of the scapulae (HP:0006713) | 10/18 | 4.89E-05 | 0.00419 |
| Hypoplastic scapulae (HP:0000882) | 10/18 | 4.89E-05 | 0.00419 |
| Short metatarsal (HP:0010743) | 14/32 | 5.06E-05 | 0.00419 |
| Abnormality of the pubic bones (HP:0003172) | 9/15 | 5.23E-05 | 0.00419 |
| Epicanthus (HP:0000286) | 55/233 | 8.3E-05 | 0.006341 |
| Deeply set eye (HP:0000490) | 25/81 | 9.71E-05 | 0.006913 |
| Short foot (HP:0001773) | 21/63 | 0.000101 | 0.006913 |
| Ventricular septal defect (HP:0001629) | 44/176 | 0.000103 | 0.006913 |
| Long philtrum (HP:0000343) | 39/152 | 0.000138 | 0.008914 |
| Hypoplasia of the maxilla (HP:0000327) | 17/47 | 0.000148 | 0.009194 |
| Laryngomalacia (HP:0001601) | 13/31 | 0.000159 | 0.009501 |
| Protruding ear (HP:0000411) | 14/35 | 0.000164 | 0.009501 |
| Short humerus (HP:0005792) | 9/17 | 0.000194 | 0.010871 |
| Synophrys (HP:0000664) | 17/49 | 0.000264 | 0.014322 |
| Microdontia (HP:0000691) | 22/72 | 0.000292 | 0.01534 |
| Abnormality of the humerus (HP:0003063) | 14/37 | 0.000327 | 0.015833 |
| Rhizomelia (HP:0008905) | 12/29 | 0.000329 | 0.015833 |
| Single transverse palmar crease (HP:0000954) | 25/87 | 0.000338 | 0.015833 |

## Slide 21
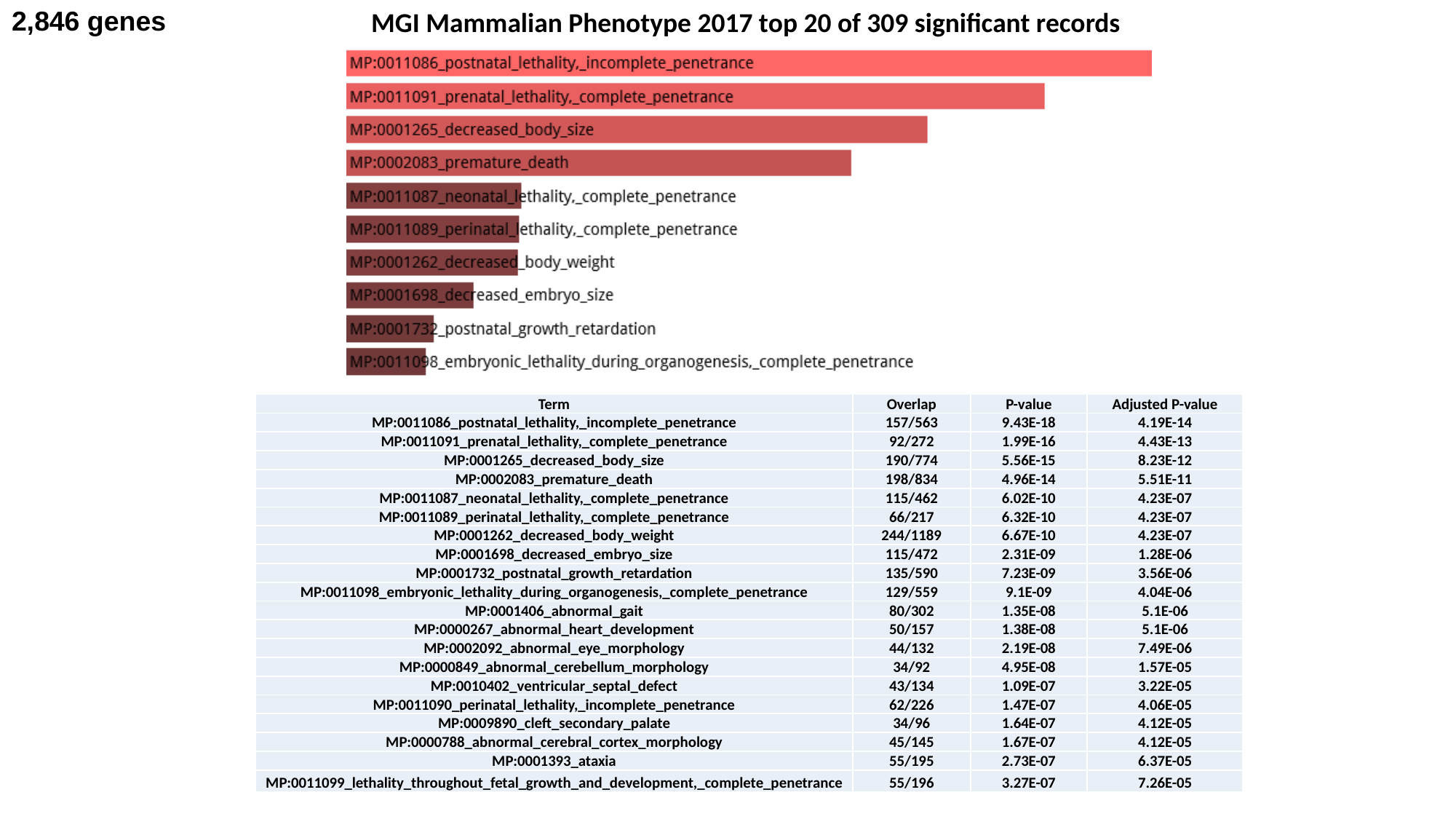

2,846 genes
MGI Mammalian Phenotype 2017 top 20 of 309 significant records
| Term | Overlap | P-value | Adjusted P-value |
| --- | --- | --- | --- |
| MP:0011086\_postnatal\_lethality,\_incomplete\_penetrance | 157/563 | 9.43E-18 | 4.19E-14 |
| MP:0011091\_prenatal\_lethality,\_complete\_penetrance | 92/272 | 1.99E-16 | 4.43E-13 |
| MP:0001265\_decreased\_body\_size | 190/774 | 5.56E-15 | 8.23E-12 |
| MP:0002083\_premature\_death | 198/834 | 4.96E-14 | 5.51E-11 |
| MP:0011087\_neonatal\_lethality,\_complete\_penetrance | 115/462 | 6.02E-10 | 4.23E-07 |
| MP:0011089\_perinatal\_lethality,\_complete\_penetrance | 66/217 | 6.32E-10 | 4.23E-07 |
| MP:0001262\_decreased\_body\_weight | 244/1189 | 6.67E-10 | 4.23E-07 |
| MP:0001698\_decreased\_embryo\_size | 115/472 | 2.31E-09 | 1.28E-06 |
| MP:0001732\_postnatal\_growth\_retardation | 135/590 | 7.23E-09 | 3.56E-06 |
| MP:0011098\_embryonic\_lethality\_during\_organogenesis,\_complete\_penetrance | 129/559 | 9.1E-09 | 4.04E-06 |
| MP:0001406\_abnormal\_gait | 80/302 | 1.35E-08 | 5.1E-06 |
| MP:0000267\_abnormal\_heart\_development | 50/157 | 1.38E-08 | 5.1E-06 |
| MP:0002092\_abnormal\_eye\_morphology | 44/132 | 2.19E-08 | 7.49E-06 |
| MP:0000849\_abnormal\_cerebellum\_morphology | 34/92 | 4.95E-08 | 1.57E-05 |
| MP:0010402\_ventricular\_septal\_defect | 43/134 | 1.09E-07 | 3.22E-05 |
| MP:0011090\_perinatal\_lethality,\_incomplete\_penetrance | 62/226 | 1.47E-07 | 4.06E-05 |
| MP:0009890\_cleft\_secondary\_palate | 34/96 | 1.64E-07 | 4.12E-05 |
| MP:0000788\_abnormal\_cerebral\_cortex\_morphology | 45/145 | 1.67E-07 | 4.12E-05 |
| MP:0001393\_ataxia | 55/195 | 2.73E-07 | 6.37E-05 |
| MP:0011099\_lethality\_throughout\_fetal\_growth\_and\_development,\_complete\_penetrance | 55/196 | 3.27E-07 | 7.26E-05 |

## Slide 22
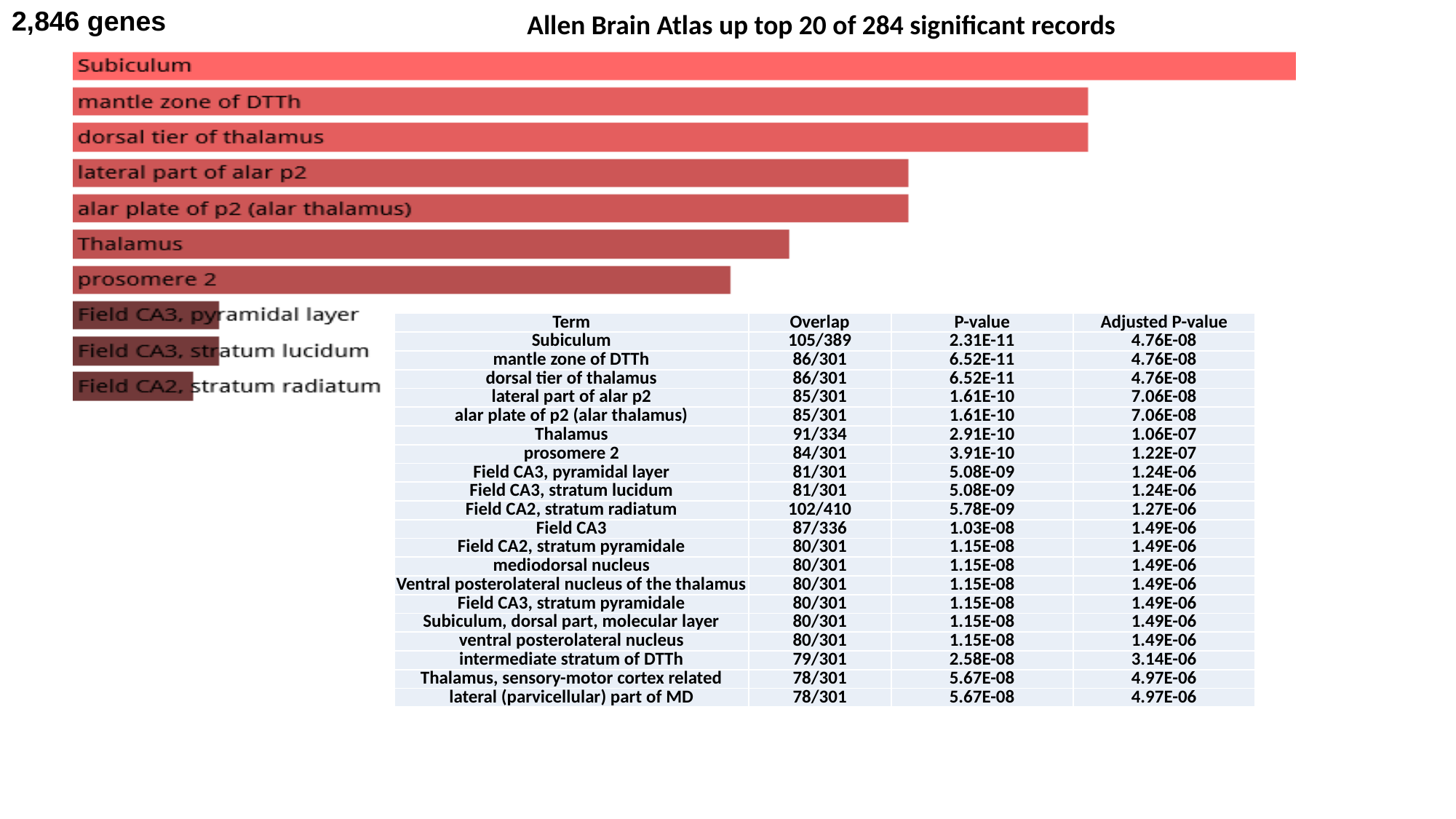

2,846 genes
Allen Brain Atlas up top 20 of 284 significant records
| Term | Overlap | P-value | Adjusted P-value |
| --- | --- | --- | --- |
| Subiculum | 105/389 | 2.31E-11 | 4.76E-08 |
| mantle zone of DTTh | 86/301 | 6.52E-11 | 4.76E-08 |
| dorsal tier of thalamus | 86/301 | 6.52E-11 | 4.76E-08 |
| lateral part of alar p2 | 85/301 | 1.61E-10 | 7.06E-08 |
| alar plate of p2 (alar thalamus) | 85/301 | 1.61E-10 | 7.06E-08 |
| Thalamus | 91/334 | 2.91E-10 | 1.06E-07 |
| prosomere 2 | 84/301 | 3.91E-10 | 1.22E-07 |
| Field CA3, pyramidal layer | 81/301 | 5.08E-09 | 1.24E-06 |
| Field CA3, stratum lucidum | 81/301 | 5.08E-09 | 1.24E-06 |
| Field CA2, stratum radiatum | 102/410 | 5.78E-09 | 1.27E-06 |
| Field CA3 | 87/336 | 1.03E-08 | 1.49E-06 |
| Field CA2, stratum pyramidale | 80/301 | 1.15E-08 | 1.49E-06 |
| mediodorsal nucleus | 80/301 | 1.15E-08 | 1.49E-06 |
| Ventral posterolateral nucleus of the thalamus | 80/301 | 1.15E-08 | 1.49E-06 |
| Field CA3, stratum pyramidale | 80/301 | 1.15E-08 | 1.49E-06 |
| Subiculum, dorsal part, molecular layer | 80/301 | 1.15E-08 | 1.49E-06 |
| ventral posterolateral nucleus | 80/301 | 1.15E-08 | 1.49E-06 |
| intermediate stratum of DTTh | 79/301 | 2.58E-08 | 3.14E-06 |
| Thalamus, sensory-motor cortex related | 78/301 | 5.67E-08 | 4.97E-06 |
| lateral (parvicellular) part of MD | 78/301 | 5.67E-08 | 4.97E-06 |

## Slide 23
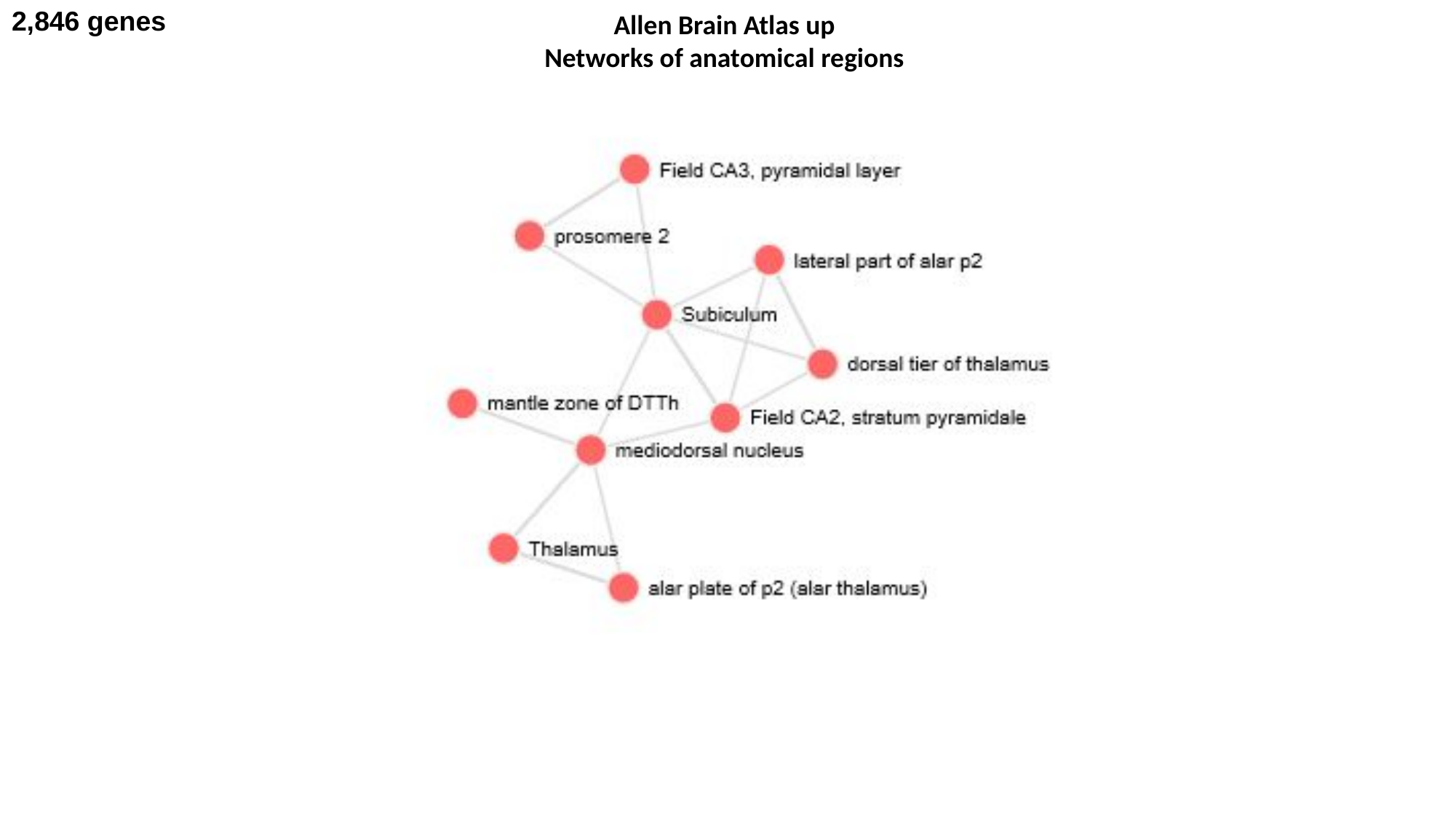

2,846 genes
Allen Brain Atlas up
Networks of anatomical regions

## Slide 24
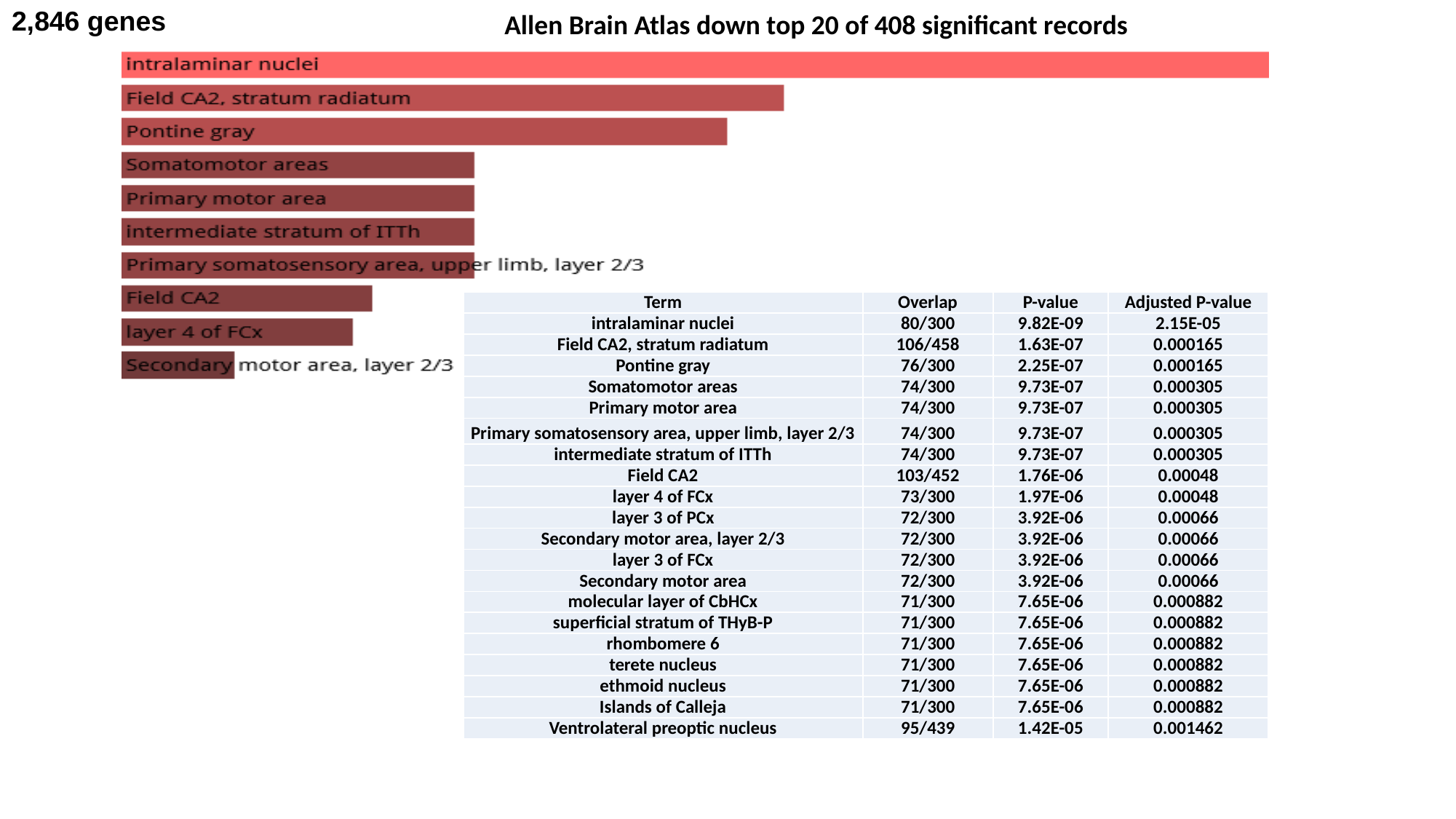

2,846 genes
Allen Brain Atlas down top 20 of 408 significant records
| Term | Overlap | P-value | Adjusted P-value |
| --- | --- | --- | --- |
| intralaminar nuclei | 80/300 | 9.82E-09 | 2.15E-05 |
| Field CA2, stratum radiatum | 106/458 | 1.63E-07 | 0.000165 |
| Pontine gray | 76/300 | 2.25E-07 | 0.000165 |
| Somatomotor areas | 74/300 | 9.73E-07 | 0.000305 |
| Primary motor area | 74/300 | 9.73E-07 | 0.000305 |
| Primary somatosensory area, upper limb, layer 2/3 | 74/300 | 9.73E-07 | 0.000305 |
| intermediate stratum of ITTh | 74/300 | 9.73E-07 | 0.000305 |
| Field CA2 | 103/452 | 1.76E-06 | 0.00048 |
| layer 4 of FCx | 73/300 | 1.97E-06 | 0.00048 |
| layer 3 of PCx | 72/300 | 3.92E-06 | 0.00066 |
| Secondary motor area, layer 2/3 | 72/300 | 3.92E-06 | 0.00066 |
| layer 3 of FCx | 72/300 | 3.92E-06 | 0.00066 |
| Secondary motor area | 72/300 | 3.92E-06 | 0.00066 |
| molecular layer of CbHCx | 71/300 | 7.65E-06 | 0.000882 |
| superficial stratum of THyB-P | 71/300 | 7.65E-06 | 0.000882 |
| rhombomere 6 | 71/300 | 7.65E-06 | 0.000882 |
| terete nucleus | 71/300 | 7.65E-06 | 0.000882 |
| ethmoid nucleus | 71/300 | 7.65E-06 | 0.000882 |
| Islands of Calleja | 71/300 | 7.65E-06 | 0.000882 |
| Ventrolateral preoptic nucleus | 95/439 | 1.42E-05 | 0.001462 |

## Slide 25
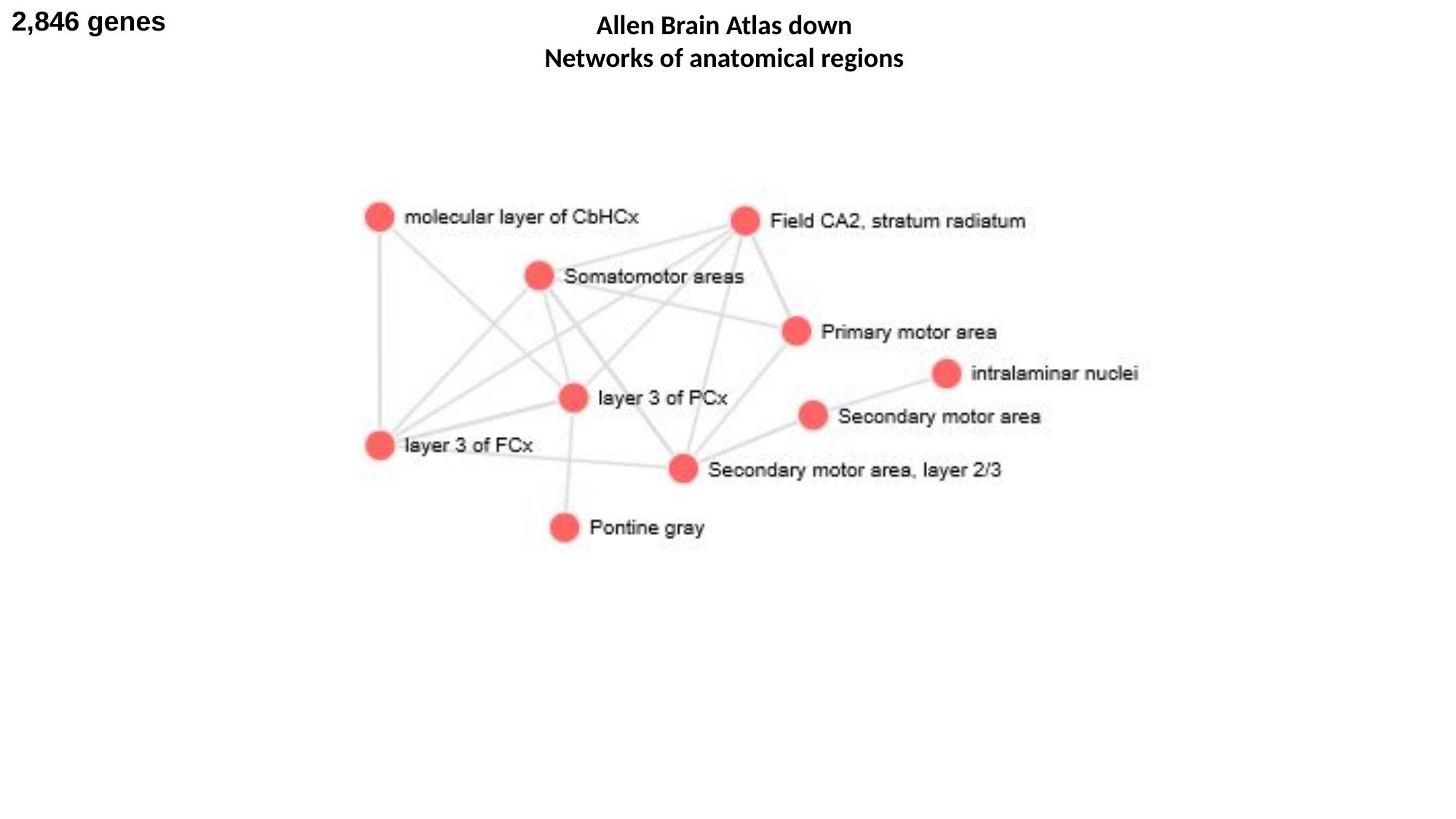

2,846 genes
Allen Brain Atlas down
Networks of anatomical regions

## Slide 26
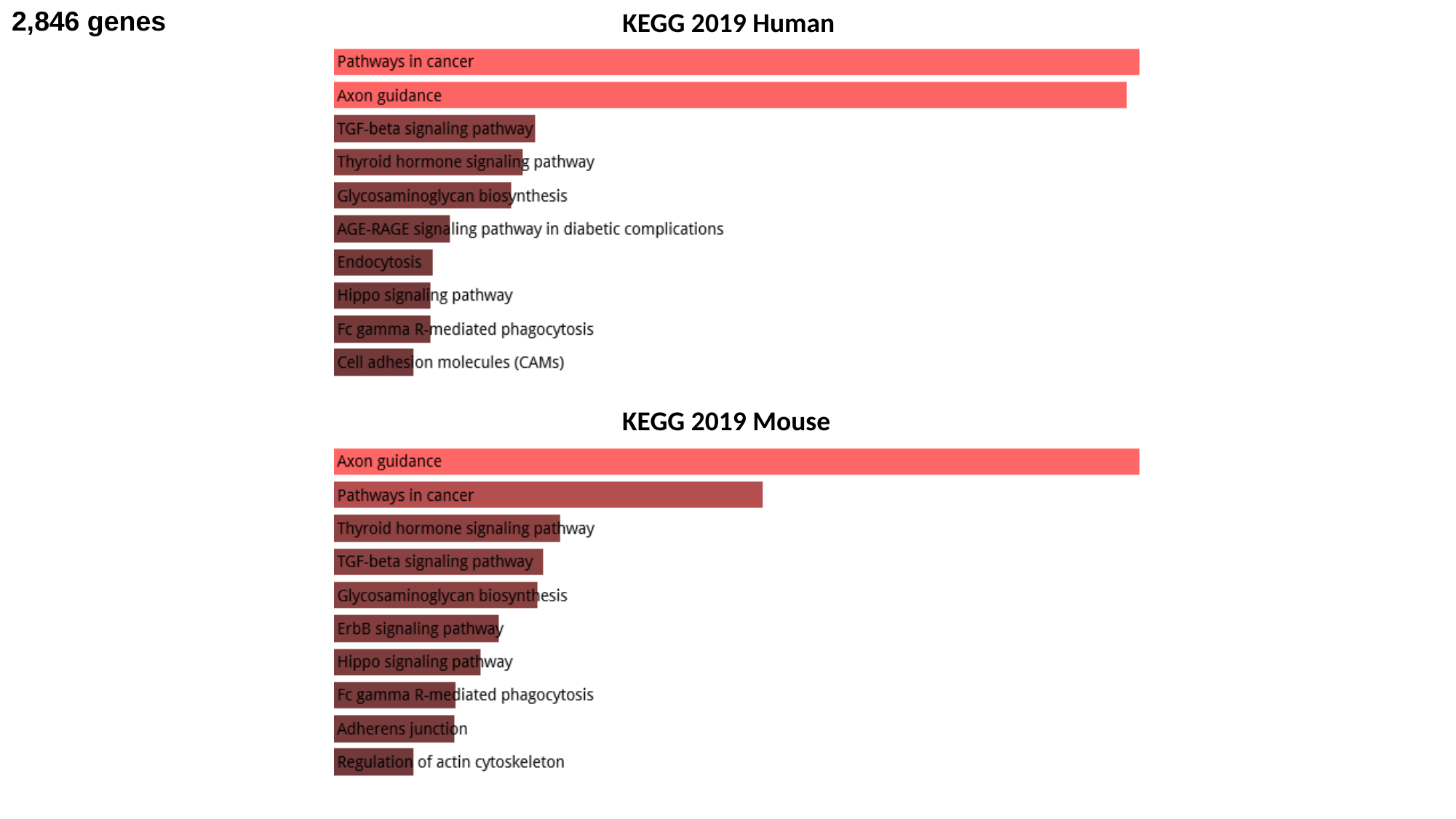

KEGG 2019 Human
2,846 genes
KEGG 2019 Mouse

## Slide 27
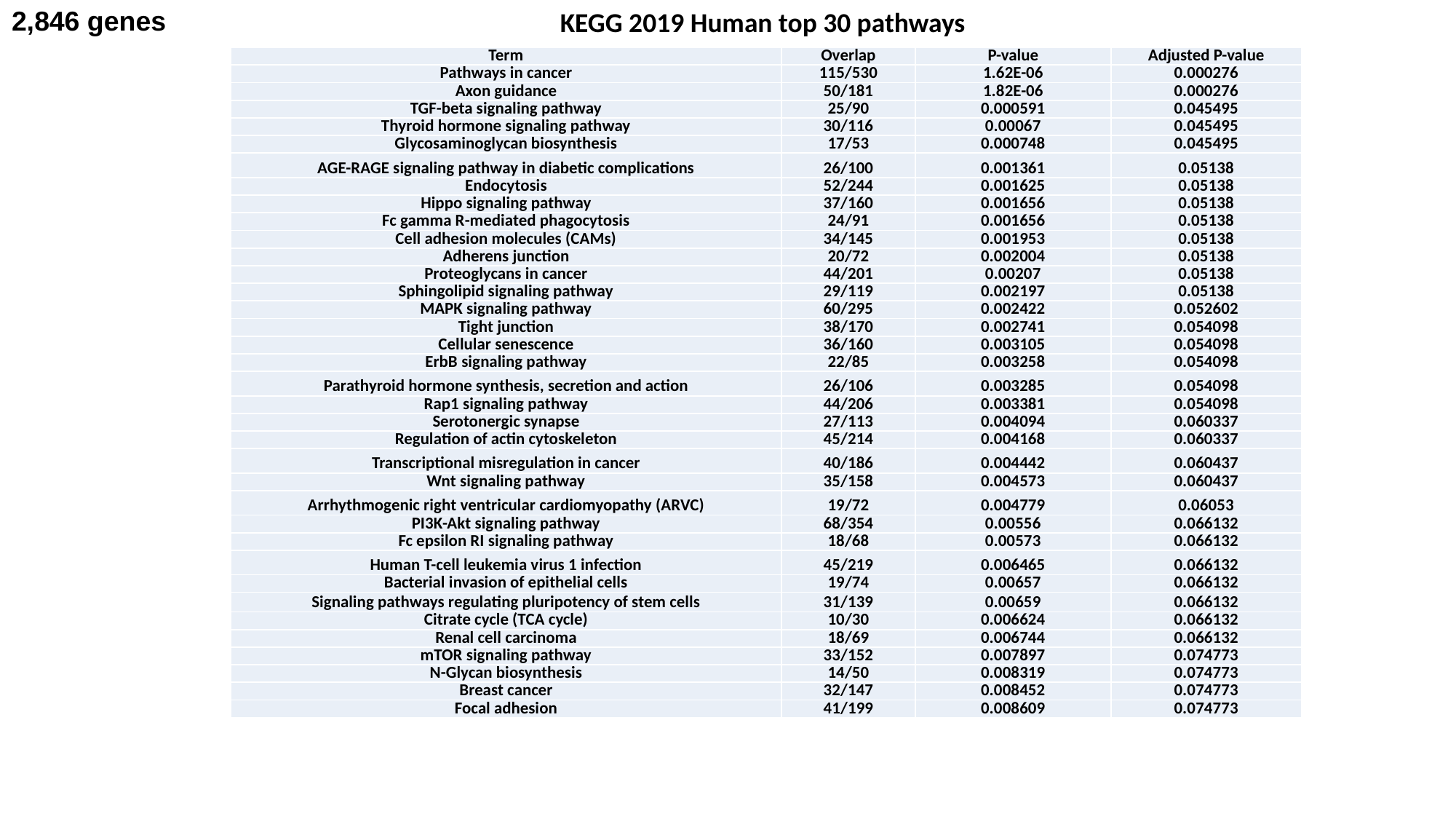

KEGG 2019 Human top 30 pathways
2,846 genes
| Term | Overlap | P-value | Adjusted P-value |
| --- | --- | --- | --- |
| Pathways in cancer | 115/530 | 1.62E-06 | 0.000276 |
| Axon guidance | 50/181 | 1.82E-06 | 0.000276 |
| TGF-beta signaling pathway | 25/90 | 0.000591 | 0.045495 |
| Thyroid hormone signaling pathway | 30/116 | 0.00067 | 0.045495 |
| Glycosaminoglycan biosynthesis | 17/53 | 0.000748 | 0.045495 |
| AGE-RAGE signaling pathway in diabetic complications | 26/100 | 0.001361 | 0.05138 |
| Endocytosis | 52/244 | 0.001625 | 0.05138 |
| Hippo signaling pathway | 37/160 | 0.001656 | 0.05138 |
| Fc gamma R-mediated phagocytosis | 24/91 | 0.001656 | 0.05138 |
| Cell adhesion molecules (CAMs) | 34/145 | 0.001953 | 0.05138 |
| Adherens junction | 20/72 | 0.002004 | 0.05138 |
| Proteoglycans in cancer | 44/201 | 0.00207 | 0.05138 |
| Sphingolipid signaling pathway | 29/119 | 0.002197 | 0.05138 |
| MAPK signaling pathway | 60/295 | 0.002422 | 0.052602 |
| Tight junction | 38/170 | 0.002741 | 0.054098 |
| Cellular senescence | 36/160 | 0.003105 | 0.054098 |
| ErbB signaling pathway | 22/85 | 0.003258 | 0.054098 |
| Parathyroid hormone synthesis, secretion and action | 26/106 | 0.003285 | 0.054098 |
| Rap1 signaling pathway | 44/206 | 0.003381 | 0.054098 |
| Serotonergic synapse | 27/113 | 0.004094 | 0.060337 |
| Regulation of actin cytoskeleton | 45/214 | 0.004168 | 0.060337 |
| Transcriptional misregulation in cancer | 40/186 | 0.004442 | 0.060437 |
| Wnt signaling pathway | 35/158 | 0.004573 | 0.060437 |
| Arrhythmogenic right ventricular cardiomyopathy (ARVC) | 19/72 | 0.004779 | 0.06053 |
| PI3K-Akt signaling pathway | 68/354 | 0.00556 | 0.066132 |
| Fc epsilon RI signaling pathway | 18/68 | 0.00573 | 0.066132 |
| Human T-cell leukemia virus 1 infection | 45/219 | 0.006465 | 0.066132 |
| Bacterial invasion of epithelial cells | 19/74 | 0.00657 | 0.066132 |
| Signaling pathways regulating pluripotency of stem cells | 31/139 | 0.00659 | 0.066132 |
| Citrate cycle (TCA cycle) | 10/30 | 0.006624 | 0.066132 |
| Renal cell carcinoma | 18/69 | 0.006744 | 0.066132 |
| mTOR signaling pathway | 33/152 | 0.007897 | 0.074773 |
| N-Glycan biosynthesis | 14/50 | 0.008319 | 0.074773 |
| Breast cancer | 32/147 | 0.008452 | 0.074773 |
| Focal adhesion | 41/199 | 0.008609 | 0.074773 |

## Slide 28
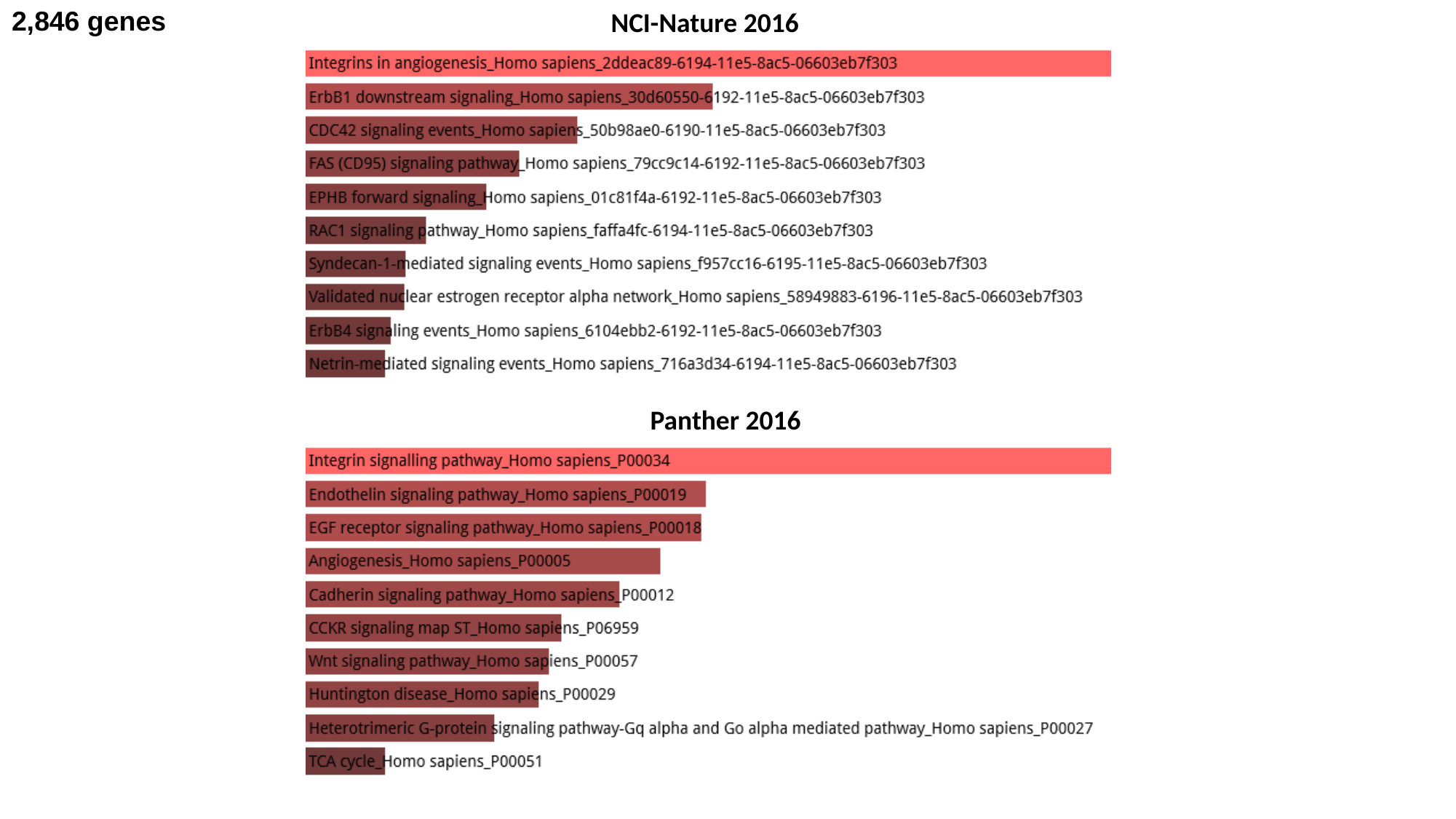

NCI-Nature 2016
2,846 genes
Panther 2016

## Slide 29
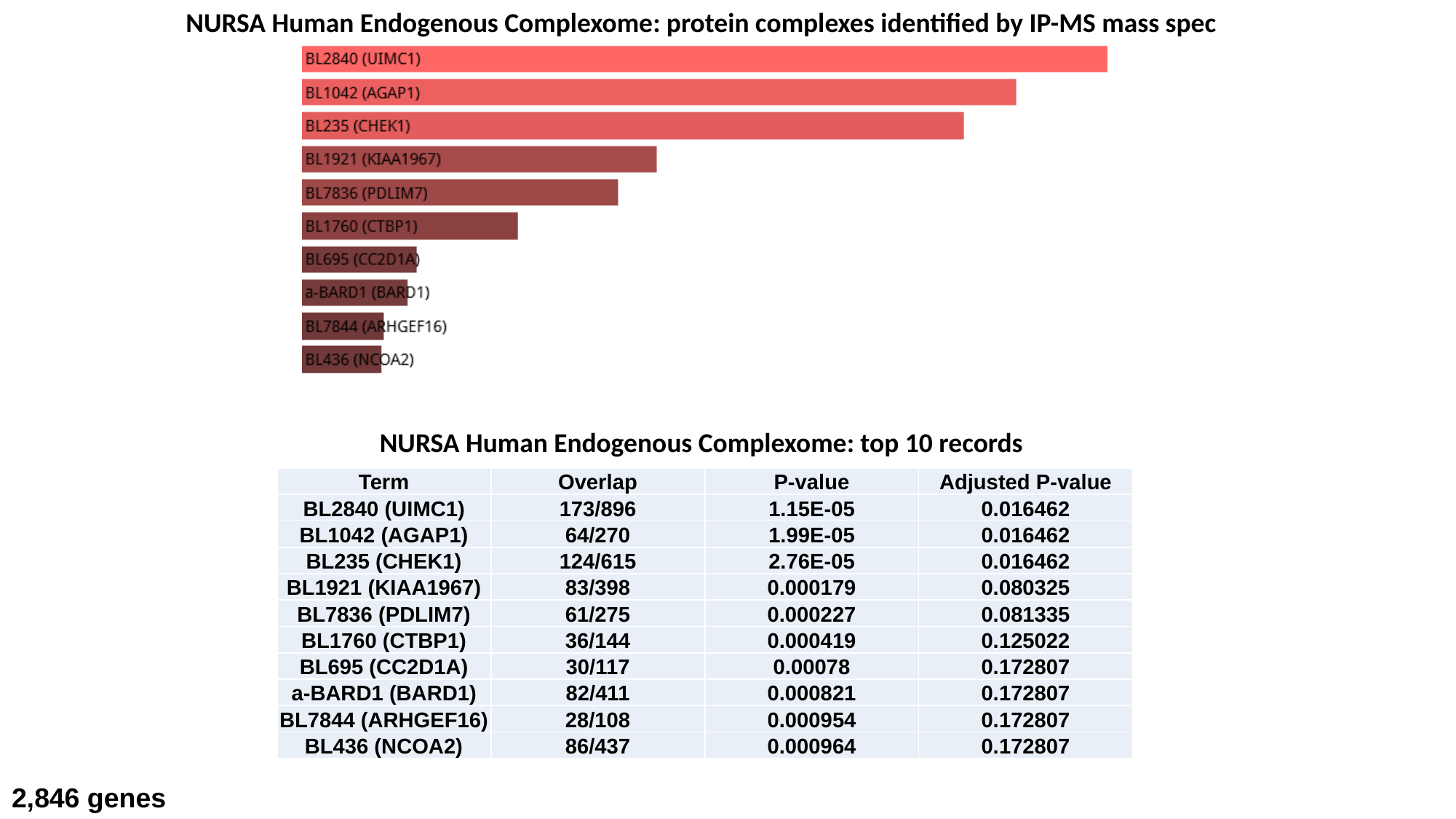

NURSA Human Endogenous Complexome: protein complexes identified by IP-MS mass spec
NURSA Human Endogenous Complexome: top 10 records
| Term | Overlap | P-value | Adjusted P-value |
| --- | --- | --- | --- |
| BL2840 (UIMC1) | 173/896 | 1.15E-05 | 0.016462 |
| BL1042 (AGAP1) | 64/270 | 1.99E-05 | 0.016462 |
| BL235 (CHEK1) | 124/615 | 2.76E-05 | 0.016462 |
| BL1921 (KIAA1967) | 83/398 | 0.000179 | 0.080325 |
| BL7836 (PDLIM7) | 61/275 | 0.000227 | 0.081335 |
| BL1760 (CTBP1) | 36/144 | 0.000419 | 0.125022 |
| BL695 (CC2D1A) | 30/117 | 0.00078 | 0.172807 |
| a-BARD1 (BARD1) | 82/411 | 0.000821 | 0.172807 |
| BL7844 (ARHGEF16) | 28/108 | 0.000954 | 0.172807 |
| BL436 (NCOA2) | 86/437 | 0.000964 | 0.172807 |
2,846 genes

## Slide 30
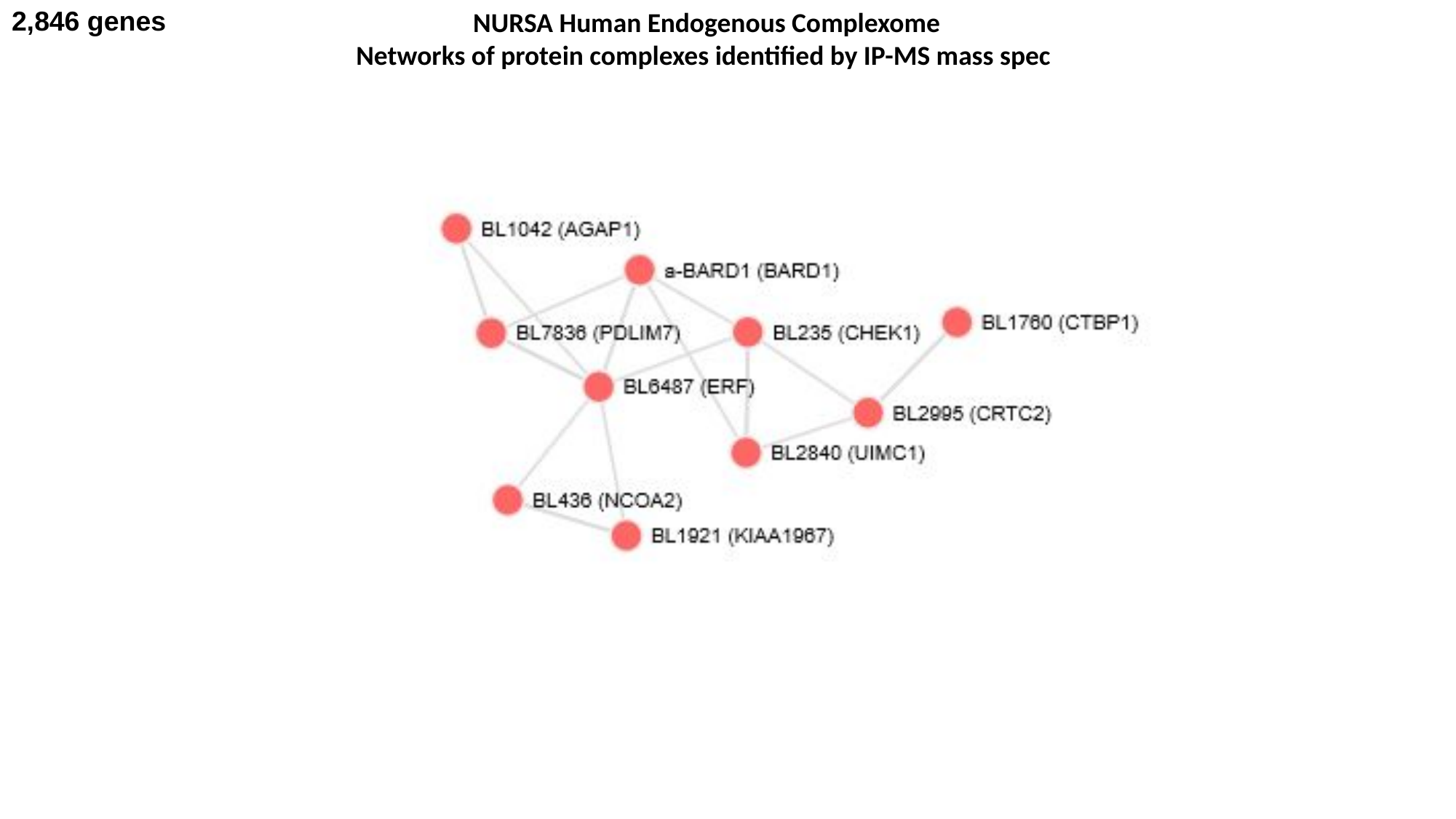

NURSA Human Endogenous Complexome
Networks of protein complexes identified by IP-MS mass spec
2,846 genes

## Slide 31
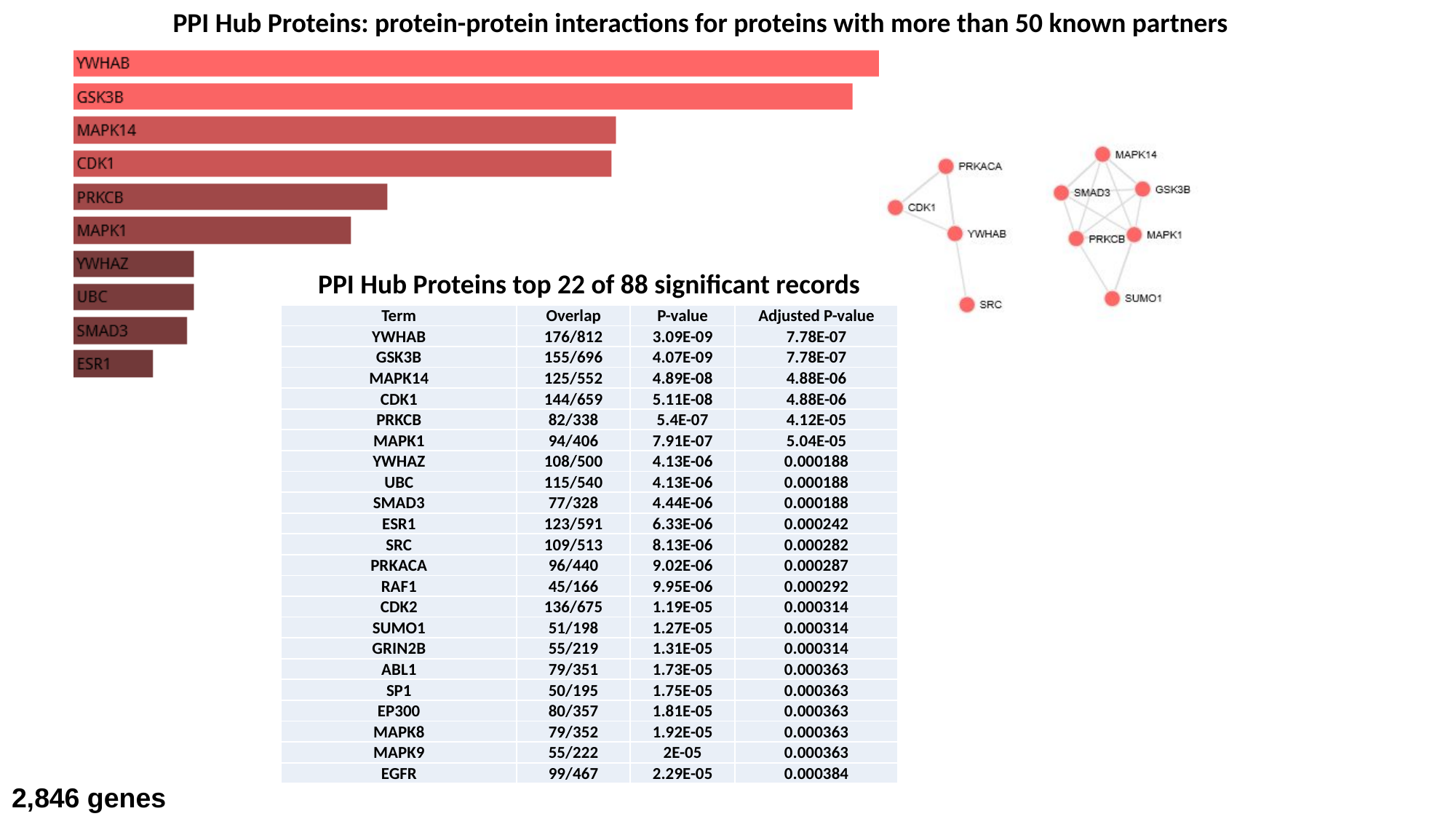

PPI Hub Proteins: protein-protein interactions for proteins with more than 50 known partners
PPI Hub Proteins top 22 of 88 significant records
| Term | Overlap | P-value | Adjusted P-value |
| --- | --- | --- | --- |
| YWHAB | 176/812 | 3.09E-09 | 7.78E-07 |
| GSK3B | 155/696 | 4.07E-09 | 7.78E-07 |
| MAPK14 | 125/552 | 4.89E-08 | 4.88E-06 |
| CDK1 | 144/659 | 5.11E-08 | 4.88E-06 |
| PRKCB | 82/338 | 5.4E-07 | 4.12E-05 |
| MAPK1 | 94/406 | 7.91E-07 | 5.04E-05 |
| YWHAZ | 108/500 | 4.13E-06 | 0.000188 |
| UBC | 115/540 | 4.13E-06 | 0.000188 |
| SMAD3 | 77/328 | 4.44E-06 | 0.000188 |
| ESR1 | 123/591 | 6.33E-06 | 0.000242 |
| SRC | 109/513 | 8.13E-06 | 0.000282 |
| PRKACA | 96/440 | 9.02E-06 | 0.000287 |
| RAF1 | 45/166 | 9.95E-06 | 0.000292 |
| CDK2 | 136/675 | 1.19E-05 | 0.000314 |
| SUMO1 | 51/198 | 1.27E-05 | 0.000314 |
| GRIN2B | 55/219 | 1.31E-05 | 0.000314 |
| ABL1 | 79/351 | 1.73E-05 | 0.000363 |
| SP1 | 50/195 | 1.75E-05 | 0.000363 |
| EP300 | 80/357 | 1.81E-05 | 0.000363 |
| MAPK8 | 79/352 | 1.92E-05 | 0.000363 |
| MAPK9 | 55/222 | 2E-05 | 0.000363 |
| EGFR | 99/467 | 2.29E-05 | 0.000384 |
2,846 genes

## Slide 32
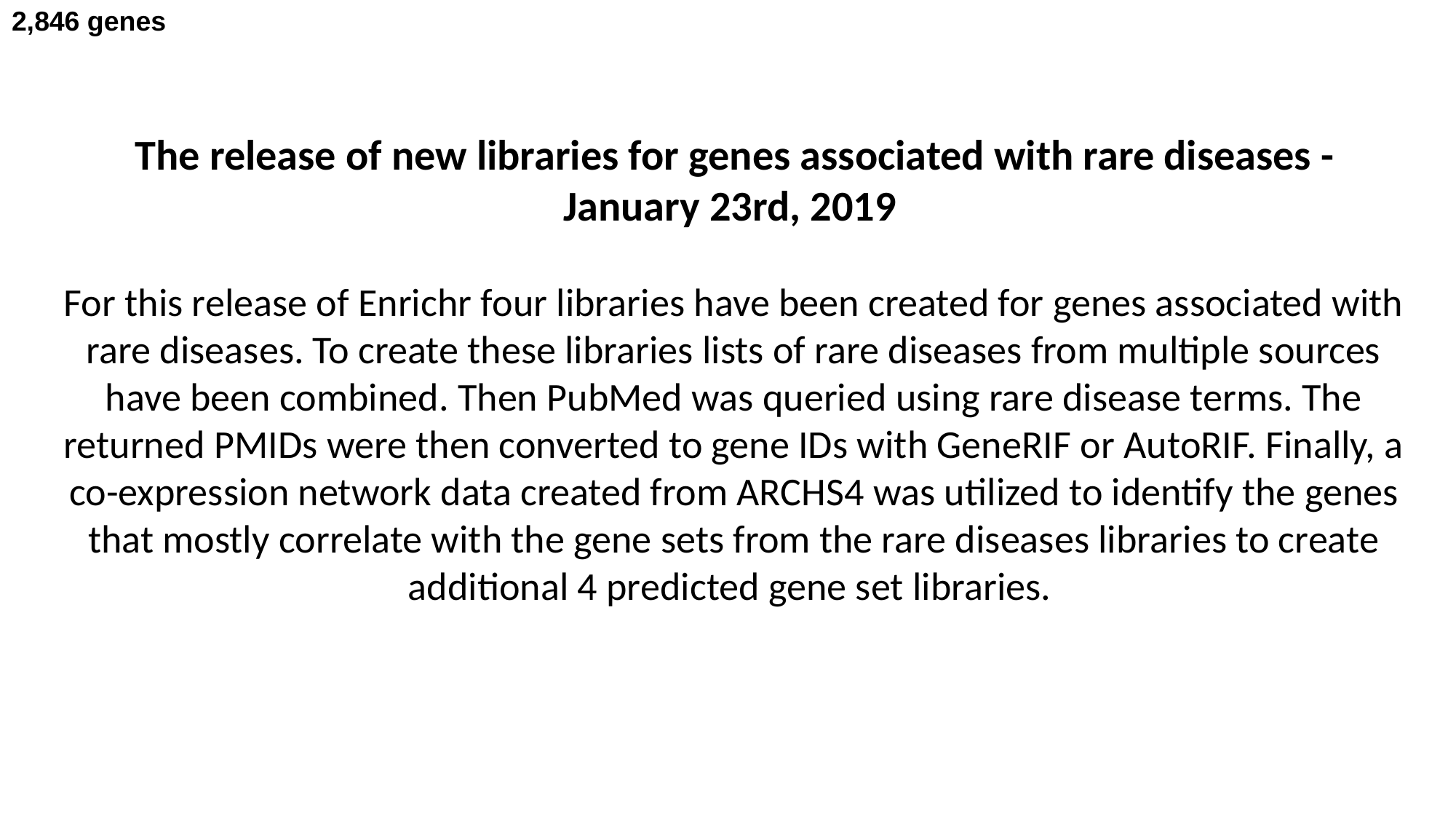

2,846 genes
The release of new libraries for genes associated with rare diseases - January 23rd, 2019
For this release of Enrichr four libraries have been created for genes associated with rare diseases. To create these libraries lists of rare diseases from multiple sources have been combined. Then PubMed was queried using rare disease terms. The returned PMIDs were then converted to gene IDs with GeneRIF or AutoRIF. Finally, a co-expression network data created from ARCHS4 was utilized to identify the genes that mostly correlate with the gene sets from the rare diseases libraries to create additional 4 predicted gene set libraries.

## Slide 33
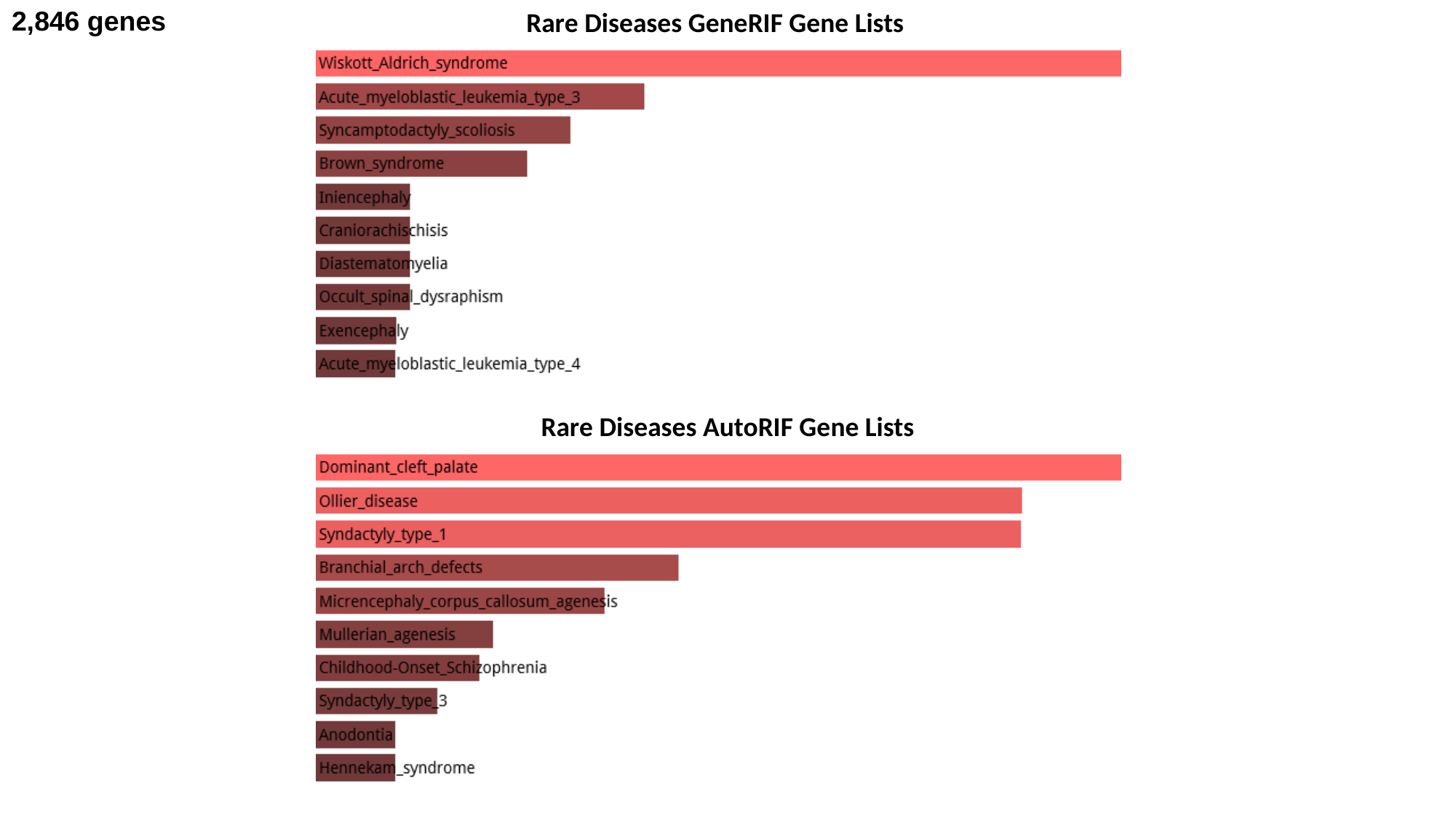

Rare Diseases GeneRIF Gene Lists
2,846 genes
Rare Diseases AutoRIF Gene Lists

## Slide 34
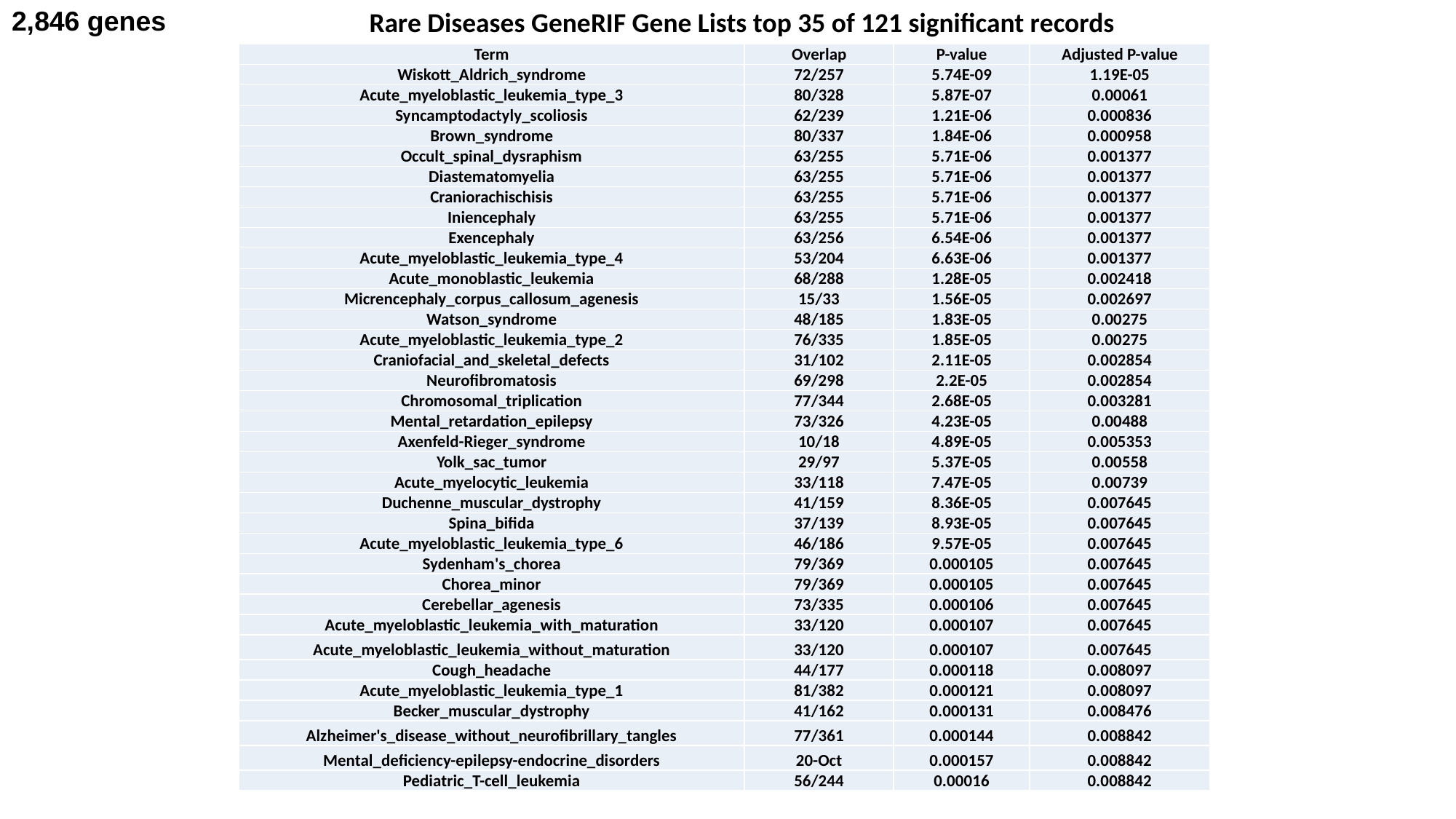

Rare Diseases GeneRIF Gene Lists top 35 of 121 significant records
2,846 genes
| Term | Overlap | P-value | Adjusted P-value |
| --- | --- | --- | --- |
| Wiskott\_Aldrich\_syndrome | 72/257 | 5.74E-09 | 1.19E-05 |
| Acute\_myeloblastic\_leukemia\_type\_3 | 80/328 | 5.87E-07 | 0.00061 |
| Syncamptodactyly\_scoliosis | 62/239 | 1.21E-06 | 0.000836 |
| Brown\_syndrome | 80/337 | 1.84E-06 | 0.000958 |
| Occult\_spinal\_dysraphism | 63/255 | 5.71E-06 | 0.001377 |
| Diastematomyelia | 63/255 | 5.71E-06 | 0.001377 |
| Craniorachischisis | 63/255 | 5.71E-06 | 0.001377 |
| Iniencephaly | 63/255 | 5.71E-06 | 0.001377 |
| Exencephaly | 63/256 | 6.54E-06 | 0.001377 |
| Acute\_myeloblastic\_leukemia\_type\_4 | 53/204 | 6.63E-06 | 0.001377 |
| Acute\_monoblastic\_leukemia | 68/288 | 1.28E-05 | 0.002418 |
| Micrencephaly\_corpus\_callosum\_agenesis | 15/33 | 1.56E-05 | 0.002697 |
| Watson\_syndrome | 48/185 | 1.83E-05 | 0.00275 |
| Acute\_myeloblastic\_leukemia\_type\_2 | 76/335 | 1.85E-05 | 0.00275 |
| Craniofacial\_and\_skeletal\_defects | 31/102 | 2.11E-05 | 0.002854 |
| Neurofibromatosis | 69/298 | 2.2E-05 | 0.002854 |
| Chromosomal\_triplication | 77/344 | 2.68E-05 | 0.003281 |
| Mental\_retardation\_epilepsy | 73/326 | 4.23E-05 | 0.00488 |
| Axenfeld-Rieger\_syndrome | 10/18 | 4.89E-05 | 0.005353 |
| Yolk\_sac\_tumor | 29/97 | 5.37E-05 | 0.00558 |
| Acute\_myelocytic\_leukemia | 33/118 | 7.47E-05 | 0.00739 |
| Duchenne\_muscular\_dystrophy | 41/159 | 8.36E-05 | 0.007645 |
| Spina\_bifida | 37/139 | 8.93E-05 | 0.007645 |
| Acute\_myeloblastic\_leukemia\_type\_6 | 46/186 | 9.57E-05 | 0.007645 |
| Sydenham's\_chorea | 79/369 | 0.000105 | 0.007645 |
| Chorea\_minor | 79/369 | 0.000105 | 0.007645 |
| Cerebellar\_agenesis | 73/335 | 0.000106 | 0.007645 |
| Acute\_myeloblastic\_leukemia\_with\_maturation | 33/120 | 0.000107 | 0.007645 |
| Acute\_myeloblastic\_leukemia\_without\_maturation | 33/120 | 0.000107 | 0.007645 |
| Cough\_headache | 44/177 | 0.000118 | 0.008097 |
| Acute\_myeloblastic\_leukemia\_type\_1 | 81/382 | 0.000121 | 0.008097 |
| Becker\_muscular\_dystrophy | 41/162 | 0.000131 | 0.008476 |
| Alzheimer's\_disease\_without\_neurofibrillary\_tangles | 77/361 | 0.000144 | 0.008842 |
| Mental\_deficiency-epilepsy-endocrine\_disorders | 20-Oct | 0.000157 | 0.008842 |
| Pediatric\_T-cell\_leukemia | 56/244 | 0.00016 | 0.008842 |

## Slide 35
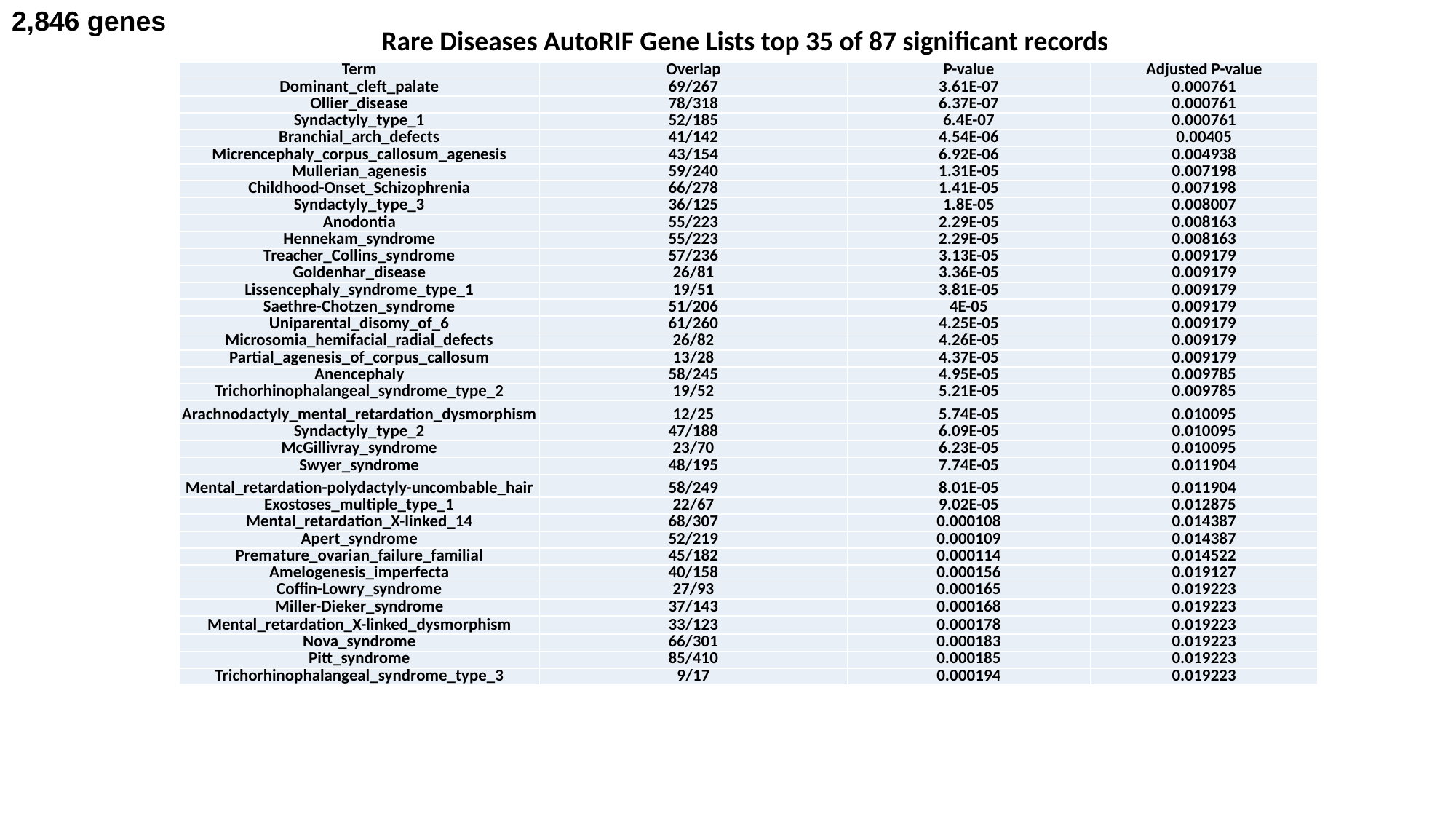

2,846 genes
Rare Diseases AutoRIF Gene Lists top 35 of 87 significant records
| Term | Overlap | P-value | Adjusted P-value |
| --- | --- | --- | --- |
| Dominant\_cleft\_palate | 69/267 | 3.61E-07 | 0.000761 |
| Ollier\_disease | 78/318 | 6.37E-07 | 0.000761 |
| Syndactyly\_type\_1 | 52/185 | 6.4E-07 | 0.000761 |
| Branchial\_arch\_defects | 41/142 | 4.54E-06 | 0.00405 |
| Micrencephaly\_corpus\_callosum\_agenesis | 43/154 | 6.92E-06 | 0.004938 |
| Mullerian\_agenesis | 59/240 | 1.31E-05 | 0.007198 |
| Childhood-Onset\_Schizophrenia | 66/278 | 1.41E-05 | 0.007198 |
| Syndactyly\_type\_3 | 36/125 | 1.8E-05 | 0.008007 |
| Anodontia | 55/223 | 2.29E-05 | 0.008163 |
| Hennekam\_syndrome | 55/223 | 2.29E-05 | 0.008163 |
| Treacher\_Collins\_syndrome | 57/236 | 3.13E-05 | 0.009179 |
| Goldenhar\_disease | 26/81 | 3.36E-05 | 0.009179 |
| Lissencephaly\_syndrome\_type\_1 | 19/51 | 3.81E-05 | 0.009179 |
| Saethre-Chotzen\_syndrome | 51/206 | 4E-05 | 0.009179 |
| Uniparental\_disomy\_of\_6 | 61/260 | 4.25E-05 | 0.009179 |
| Microsomia\_hemifacial\_radial\_defects | 26/82 | 4.26E-05 | 0.009179 |
| Partial\_agenesis\_of\_corpus\_callosum | 13/28 | 4.37E-05 | 0.009179 |
| Anencephaly | 58/245 | 4.95E-05 | 0.009785 |
| Trichorhinophalangeal\_syndrome\_type\_2 | 19/52 | 5.21E-05 | 0.009785 |
| Arachnodactyly\_mental\_retardation\_dysmorphism | 12/25 | 5.74E-05 | 0.010095 |
| Syndactyly\_type\_2 | 47/188 | 6.09E-05 | 0.010095 |
| McGillivray\_syndrome | 23/70 | 6.23E-05 | 0.010095 |
| Swyer\_syndrome | 48/195 | 7.74E-05 | 0.011904 |
| Mental\_retardation-polydactyly-uncombable\_hair | 58/249 | 8.01E-05 | 0.011904 |
| Exostoses\_multiple\_type\_1 | 22/67 | 9.02E-05 | 0.012875 |
| Mental\_retardation\_X-linked\_14 | 68/307 | 0.000108 | 0.014387 |
| Apert\_syndrome | 52/219 | 0.000109 | 0.014387 |
| Premature\_ovarian\_failure\_familial | 45/182 | 0.000114 | 0.014522 |
| Amelogenesis\_imperfecta | 40/158 | 0.000156 | 0.019127 |
| Coffin-Lowry\_syndrome | 27/93 | 0.000165 | 0.019223 |
| Miller-Dieker\_syndrome | 37/143 | 0.000168 | 0.019223 |
| Mental\_retardation\_X-linked\_dysmorphism | 33/123 | 0.000178 | 0.019223 |
| Nova\_syndrome | 66/301 | 0.000183 | 0.019223 |
| Pitt\_syndrome | 85/410 | 0.000185 | 0.019223 |
| Trichorhinophalangeal\_syndrome\_type\_3 | 9/17 | 0.000194 | 0.019223 |

## Slide 36
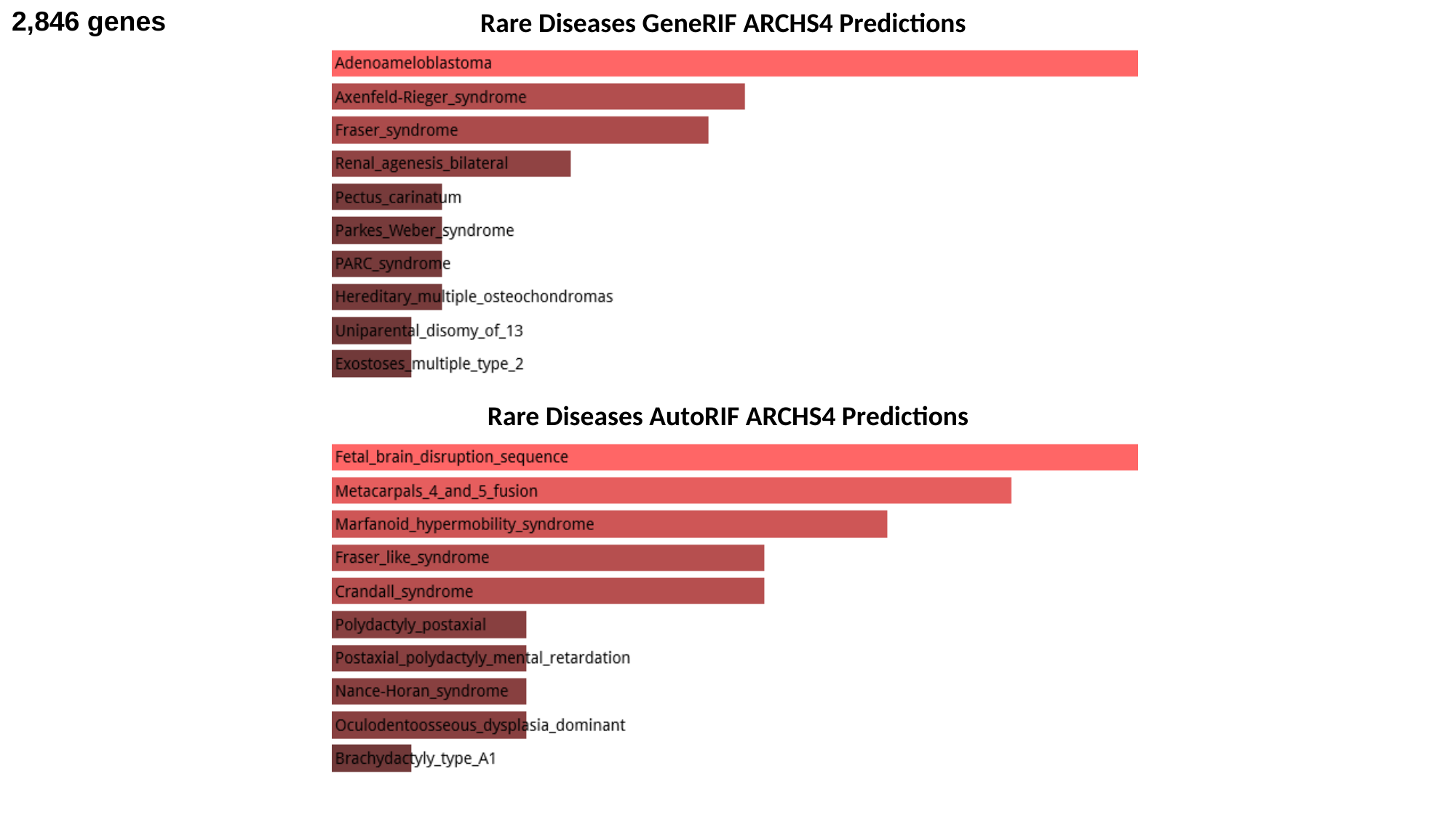

Rare Diseases GeneRIF ARCHS4 Predictions
2,846 genes
Rare Diseases AutoRIF ARCHS4 Predictions

## Slide 37
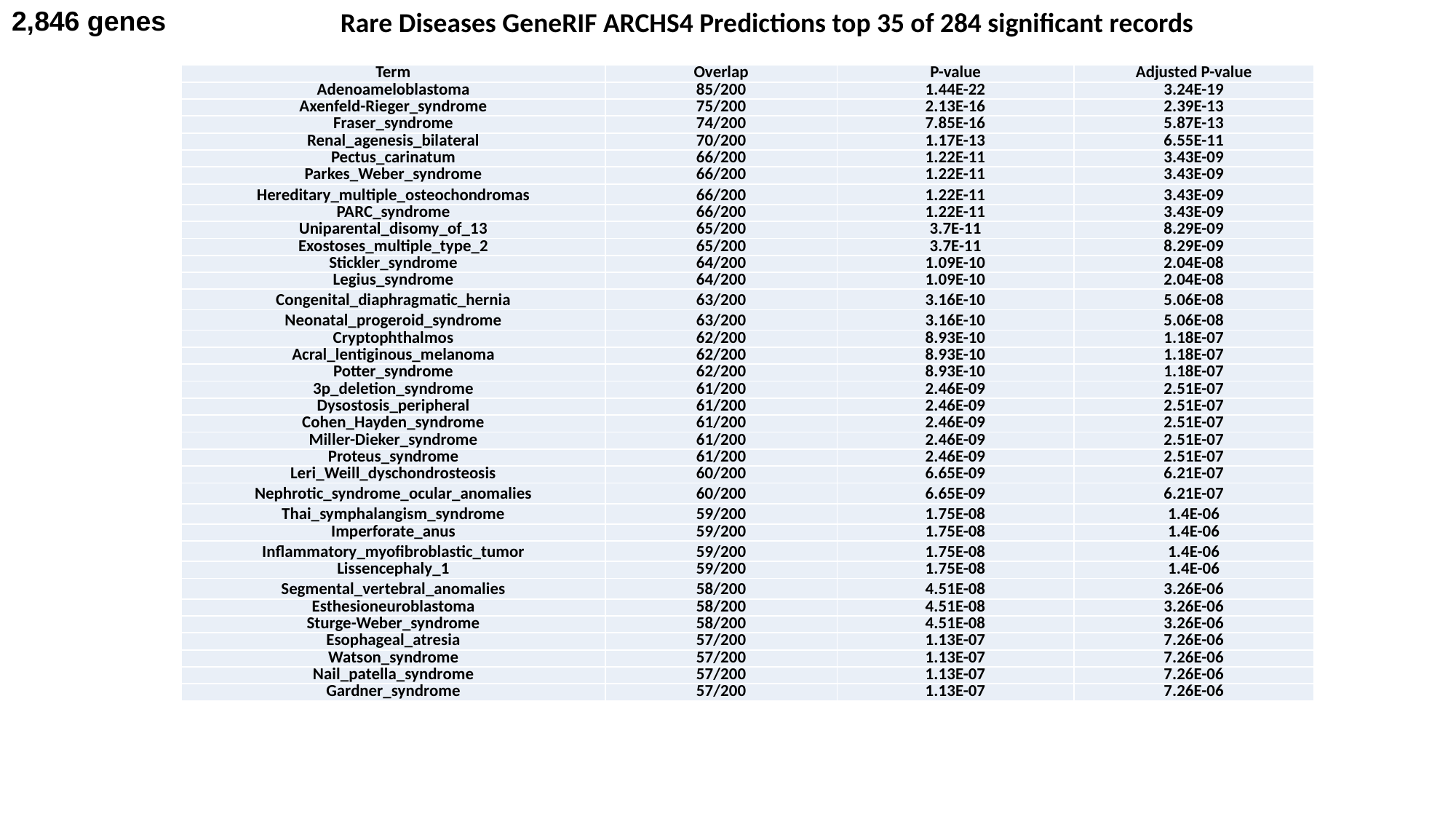

Rare Diseases GeneRIF ARCHS4 Predictions top 35 of 284 significant records
2,846 genes
| Term | Overlap | P-value | Adjusted P-value |
| --- | --- | --- | --- |
| Adenoameloblastoma | 85/200 | 1.44E-22 | 3.24E-19 |
| Axenfeld-Rieger\_syndrome | 75/200 | 2.13E-16 | 2.39E-13 |
| Fraser\_syndrome | 74/200 | 7.85E-16 | 5.87E-13 |
| Renal\_agenesis\_bilateral | 70/200 | 1.17E-13 | 6.55E-11 |
| Pectus\_carinatum | 66/200 | 1.22E-11 | 3.43E-09 |
| Parkes\_Weber\_syndrome | 66/200 | 1.22E-11 | 3.43E-09 |
| Hereditary\_multiple\_osteochondromas | 66/200 | 1.22E-11 | 3.43E-09 |
| PARC\_syndrome | 66/200 | 1.22E-11 | 3.43E-09 |
| Uniparental\_disomy\_of\_13 | 65/200 | 3.7E-11 | 8.29E-09 |
| Exostoses\_multiple\_type\_2 | 65/200 | 3.7E-11 | 8.29E-09 |
| Stickler\_syndrome | 64/200 | 1.09E-10 | 2.04E-08 |
| Legius\_syndrome | 64/200 | 1.09E-10 | 2.04E-08 |
| Congenital\_diaphragmatic\_hernia | 63/200 | 3.16E-10 | 5.06E-08 |
| Neonatal\_progeroid\_syndrome | 63/200 | 3.16E-10 | 5.06E-08 |
| Cryptophthalmos | 62/200 | 8.93E-10 | 1.18E-07 |
| Acral\_lentiginous\_melanoma | 62/200 | 8.93E-10 | 1.18E-07 |
| Potter\_syndrome | 62/200 | 8.93E-10 | 1.18E-07 |
| 3p\_deletion\_syndrome | 61/200 | 2.46E-09 | 2.51E-07 |
| Dysostosis\_peripheral | 61/200 | 2.46E-09 | 2.51E-07 |
| Cohen\_Hayden\_syndrome | 61/200 | 2.46E-09 | 2.51E-07 |
| Miller-Dieker\_syndrome | 61/200 | 2.46E-09 | 2.51E-07 |
| Proteus\_syndrome | 61/200 | 2.46E-09 | 2.51E-07 |
| Leri\_Weill\_dyschondrosteosis | 60/200 | 6.65E-09 | 6.21E-07 |
| Nephrotic\_syndrome\_ocular\_anomalies | 60/200 | 6.65E-09 | 6.21E-07 |
| Thai\_symphalangism\_syndrome | 59/200 | 1.75E-08 | 1.4E-06 |
| Imperforate\_anus | 59/200 | 1.75E-08 | 1.4E-06 |
| Inflammatory\_myofibroblastic\_tumor | 59/200 | 1.75E-08 | 1.4E-06 |
| Lissencephaly\_1 | 59/200 | 1.75E-08 | 1.4E-06 |
| Segmental\_vertebral\_anomalies | 58/200 | 4.51E-08 | 3.26E-06 |
| Esthesioneuroblastoma | 58/200 | 4.51E-08 | 3.26E-06 |
| Sturge-Weber\_syndrome | 58/200 | 4.51E-08 | 3.26E-06 |
| Esophageal\_atresia | 57/200 | 1.13E-07 | 7.26E-06 |
| Watson\_syndrome | 57/200 | 1.13E-07 | 7.26E-06 |
| Nail\_patella\_syndrome | 57/200 | 1.13E-07 | 7.26E-06 |
| Gardner\_syndrome | 57/200 | 1.13E-07 | 7.26E-06 |

## Slide 38
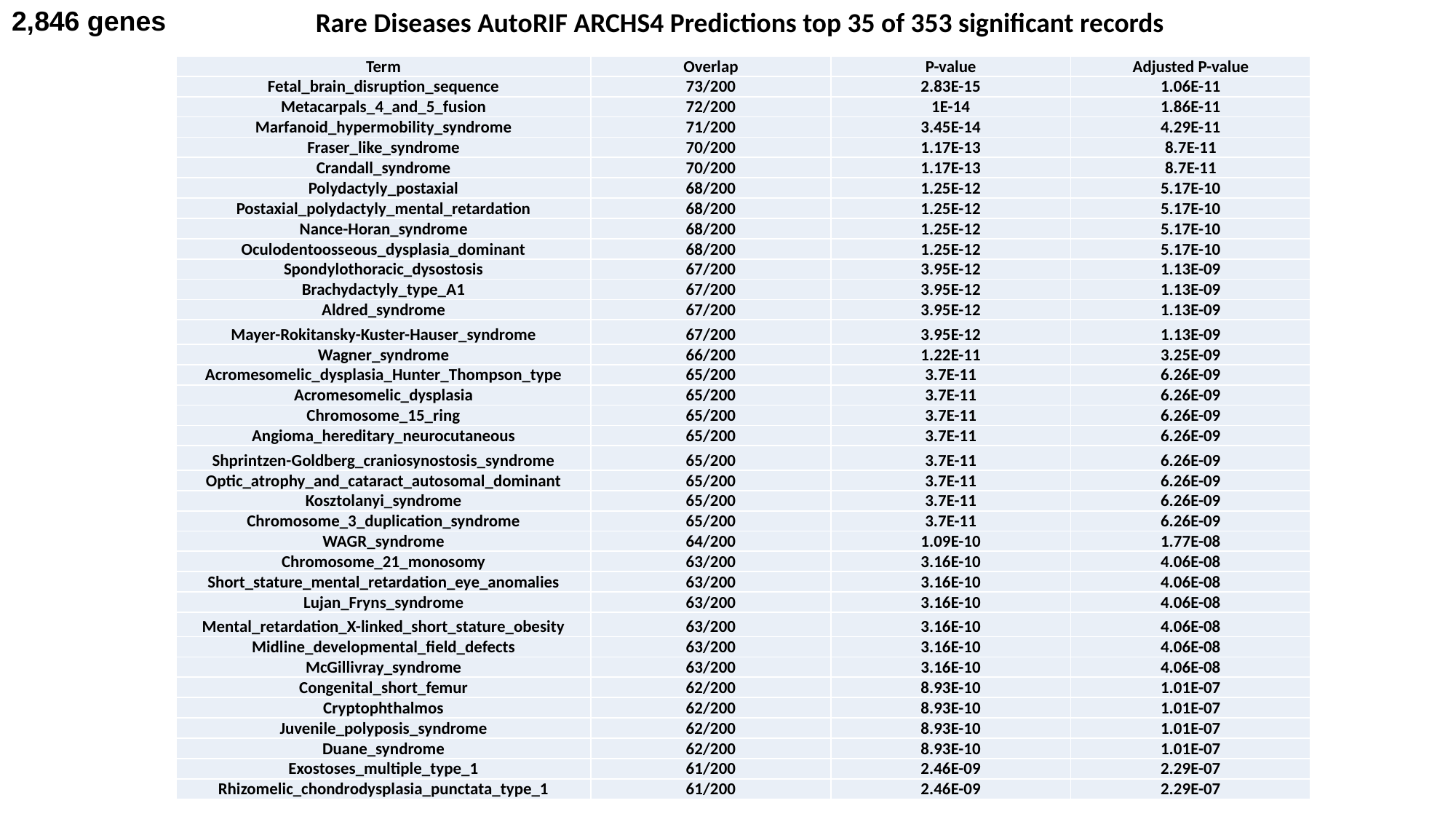

Rare Diseases AutoRIF ARCHS4 Predictions top 35 of 353 significant records
2,846 genes
| Term | Overlap | P-value | Adjusted P-value |
| --- | --- | --- | --- |
| Fetal\_brain\_disruption\_sequence | 73/200 | 2.83E-15 | 1.06E-11 |
| Metacarpals\_4\_and\_5\_fusion | 72/200 | 1E-14 | 1.86E-11 |
| Marfanoid\_hypermobility\_syndrome | 71/200 | 3.45E-14 | 4.29E-11 |
| Fraser\_like\_syndrome | 70/200 | 1.17E-13 | 8.7E-11 |
| Crandall\_syndrome | 70/200 | 1.17E-13 | 8.7E-11 |
| Polydactyly\_postaxial | 68/200 | 1.25E-12 | 5.17E-10 |
| Postaxial\_polydactyly\_mental\_retardation | 68/200 | 1.25E-12 | 5.17E-10 |
| Nance-Horan\_syndrome | 68/200 | 1.25E-12 | 5.17E-10 |
| Oculodentoosseous\_dysplasia\_dominant | 68/200 | 1.25E-12 | 5.17E-10 |
| Spondylothoracic\_dysostosis | 67/200 | 3.95E-12 | 1.13E-09 |
| Brachydactyly\_type\_A1 | 67/200 | 3.95E-12 | 1.13E-09 |
| Aldred\_syndrome | 67/200 | 3.95E-12 | 1.13E-09 |
| Mayer-Rokitansky-Kuster-Hauser\_syndrome | 67/200 | 3.95E-12 | 1.13E-09 |
| Wagner\_syndrome | 66/200 | 1.22E-11 | 3.25E-09 |
| Acromesomelic\_dysplasia\_Hunter\_Thompson\_type | 65/200 | 3.7E-11 | 6.26E-09 |
| Acromesomelic\_dysplasia | 65/200 | 3.7E-11 | 6.26E-09 |
| Chromosome\_15\_ring | 65/200 | 3.7E-11 | 6.26E-09 |
| Angioma\_hereditary\_neurocutaneous | 65/200 | 3.7E-11 | 6.26E-09 |
| Shprintzen-Goldberg\_craniosynostosis\_syndrome | 65/200 | 3.7E-11 | 6.26E-09 |
| Optic\_atrophy\_and\_cataract\_autosomal\_dominant | 65/200 | 3.7E-11 | 6.26E-09 |
| Kosztolanyi\_syndrome | 65/200 | 3.7E-11 | 6.26E-09 |
| Chromosome\_3\_duplication\_syndrome | 65/200 | 3.7E-11 | 6.26E-09 |
| WAGR\_syndrome | 64/200 | 1.09E-10 | 1.77E-08 |
| Chromosome\_21\_monosomy | 63/200 | 3.16E-10 | 4.06E-08 |
| Short\_stature\_mental\_retardation\_eye\_anomalies | 63/200 | 3.16E-10 | 4.06E-08 |
| Lujan\_Fryns\_syndrome | 63/200 | 3.16E-10 | 4.06E-08 |
| Mental\_retardation\_X-linked\_short\_stature\_obesity | 63/200 | 3.16E-10 | 4.06E-08 |
| Midline\_developmental\_field\_defects | 63/200 | 3.16E-10 | 4.06E-08 |
| McGillivray\_syndrome | 63/200 | 3.16E-10 | 4.06E-08 |
| Congenital\_short\_femur | 62/200 | 8.93E-10 | 1.01E-07 |
| Cryptophthalmos | 62/200 | 8.93E-10 | 1.01E-07 |
| Juvenile\_polyposis\_syndrome | 62/200 | 8.93E-10 | 1.01E-07 |
| Duane\_syndrome | 62/200 | 8.93E-10 | 1.01E-07 |
| Exostoses\_multiple\_type\_1 | 61/200 | 2.46E-09 | 2.29E-07 |
| Rhizomelic\_chondrodysplasia\_punctata\_type\_1 | 61/200 | 2.46E-09 | 2.29E-07 |

## Slide 39
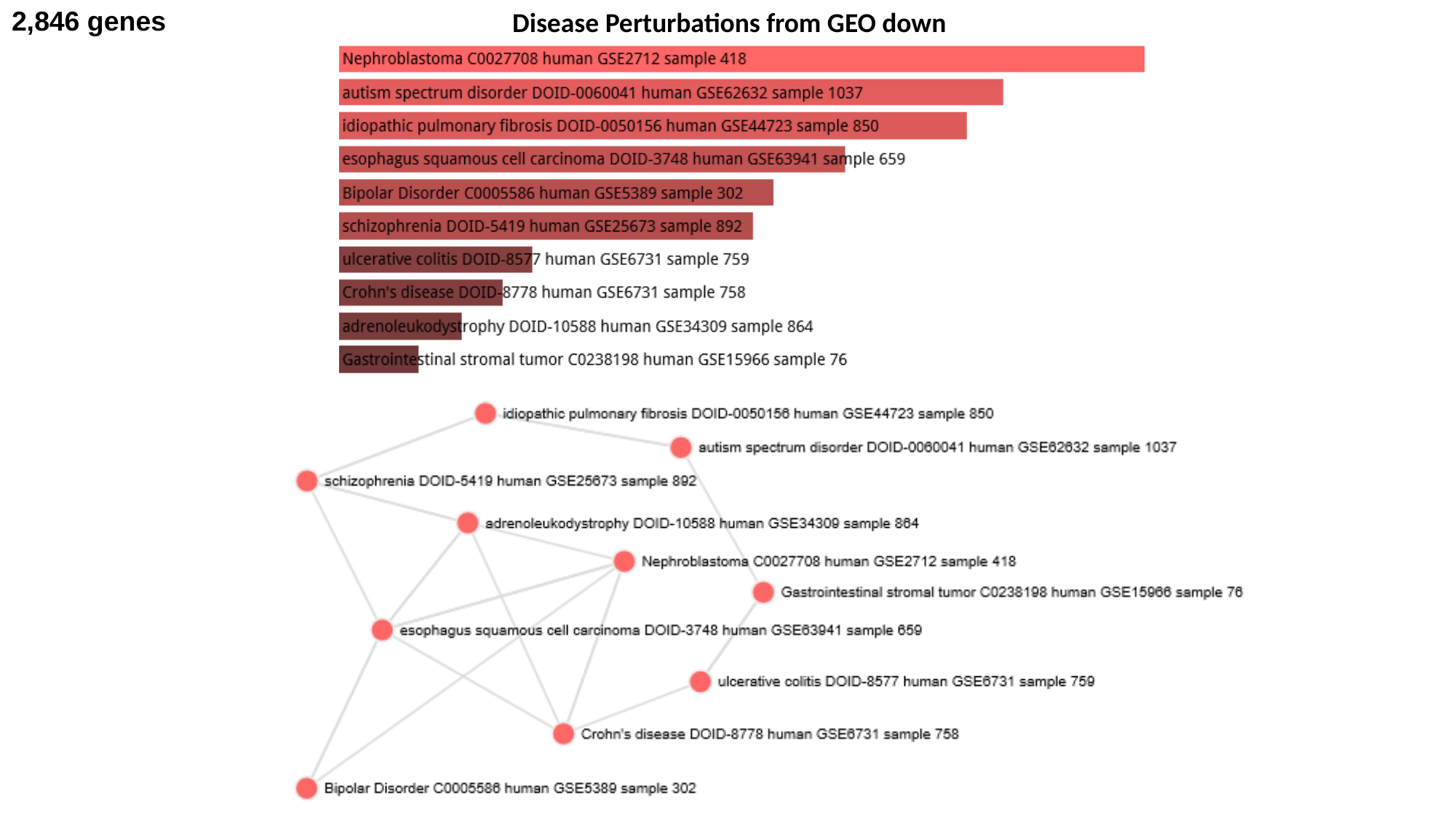

Disease Perturbations from GEO down
2,846 genes

## Slide 40
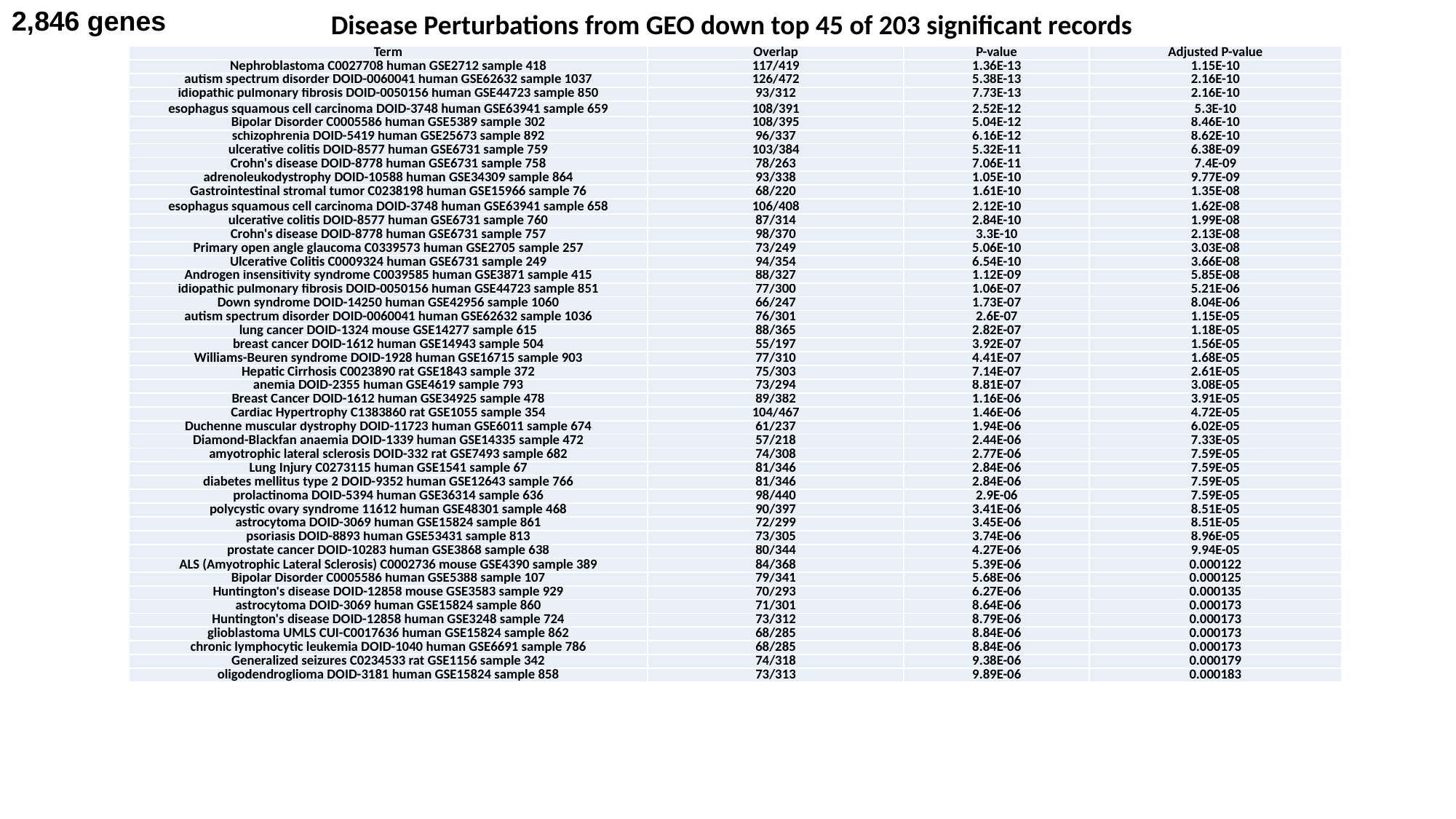

2,846 genes
Disease Perturbations from GEO down top 45 of 203 significant records
| Term | Overlap | P-value | Adjusted P-value |
| --- | --- | --- | --- |
| Nephroblastoma C0027708 human GSE2712 sample 418 | 117/419 | 1.36E-13 | 1.15E-10 |
| autism spectrum disorder DOID-0060041 human GSE62632 sample 1037 | 126/472 | 5.38E-13 | 2.16E-10 |
| idiopathic pulmonary fibrosis DOID-0050156 human GSE44723 sample 850 | 93/312 | 7.73E-13 | 2.16E-10 |
| esophagus squamous cell carcinoma DOID-3748 human GSE63941 sample 659 | 108/391 | 2.52E-12 | 5.3E-10 |
| Bipolar Disorder C0005586 human GSE5389 sample 302 | 108/395 | 5.04E-12 | 8.46E-10 |
| schizophrenia DOID-5419 human GSE25673 sample 892 | 96/337 | 6.16E-12 | 8.62E-10 |
| ulcerative colitis DOID-8577 human GSE6731 sample 759 | 103/384 | 5.32E-11 | 6.38E-09 |
| Crohn's disease DOID-8778 human GSE6731 sample 758 | 78/263 | 7.06E-11 | 7.4E-09 |
| adrenoleukodystrophy DOID-10588 human GSE34309 sample 864 | 93/338 | 1.05E-10 | 9.77E-09 |
| Gastrointestinal stromal tumor C0238198 human GSE15966 sample 76 | 68/220 | 1.61E-10 | 1.35E-08 |
| esophagus squamous cell carcinoma DOID-3748 human GSE63941 sample 658 | 106/408 | 2.12E-10 | 1.62E-08 |
| ulcerative colitis DOID-8577 human GSE6731 sample 760 | 87/314 | 2.84E-10 | 1.99E-08 |
| Crohn's disease DOID-8778 human GSE6731 sample 757 | 98/370 | 3.3E-10 | 2.13E-08 |
| Primary open angle glaucoma C0339573 human GSE2705 sample 257 | 73/249 | 5.06E-10 | 3.03E-08 |
| Ulcerative Colitis C0009324 human GSE6731 sample 249 | 94/354 | 6.54E-10 | 3.66E-08 |
| Androgen insensitivity syndrome C0039585 human GSE3871 sample 415 | 88/327 | 1.12E-09 | 5.85E-08 |
| idiopathic pulmonary fibrosis DOID-0050156 human GSE44723 sample 851 | 77/300 | 1.06E-07 | 5.21E-06 |
| Down syndrome DOID-14250 human GSE42956 sample 1060 | 66/247 | 1.73E-07 | 8.04E-06 |
| autism spectrum disorder DOID-0060041 human GSE62632 sample 1036 | 76/301 | 2.6E-07 | 1.15E-05 |
| lung cancer DOID-1324 mouse GSE14277 sample 615 | 88/365 | 2.82E-07 | 1.18E-05 |
| breast cancer DOID-1612 human GSE14943 sample 504 | 55/197 | 3.92E-07 | 1.56E-05 |
| Williams-Beuren syndrome DOID-1928 human GSE16715 sample 903 | 77/310 | 4.41E-07 | 1.68E-05 |
| Hepatic Cirrhosis C0023890 rat GSE1843 sample 372 | 75/303 | 7.14E-07 | 2.61E-05 |
| anemia DOID-2355 human GSE4619 sample 793 | 73/294 | 8.81E-07 | 3.08E-05 |
| Breast Cancer DOID-1612 human GSE34925 sample 478 | 89/382 | 1.16E-06 | 3.91E-05 |
| Cardiac Hypertrophy C1383860 rat GSE1055 sample 354 | 104/467 | 1.46E-06 | 4.72E-05 |
| Duchenne muscular dystrophy DOID-11723 human GSE6011 sample 674 | 61/237 | 1.94E-06 | 6.02E-05 |
| Diamond-Blackfan anaemia DOID-1339 human GSE14335 sample 472 | 57/218 | 2.44E-06 | 7.33E-05 |
| amyotrophic lateral sclerosis DOID-332 rat GSE7493 sample 682 | 74/308 | 2.77E-06 | 7.59E-05 |
| Lung Injury C0273115 human GSE1541 sample 67 | 81/346 | 2.84E-06 | 7.59E-05 |
| diabetes mellitus type 2 DOID-9352 human GSE12643 sample 766 | 81/346 | 2.84E-06 | 7.59E-05 |
| prolactinoma DOID-5394 human GSE36314 sample 636 | 98/440 | 2.9E-06 | 7.59E-05 |
| polycystic ovary syndrome 11612 human GSE48301 sample 468 | 90/397 | 3.41E-06 | 8.51E-05 |
| astrocytoma DOID-3069 human GSE15824 sample 861 | 72/299 | 3.45E-06 | 8.51E-05 |
| psoriasis DOID-8893 human GSE53431 sample 813 | 73/305 | 3.74E-06 | 8.96E-05 |
| prostate cancer DOID-10283 human GSE3868 sample 638 | 80/344 | 4.27E-06 | 9.94E-05 |
| ALS (Amyotrophic Lateral Sclerosis) C0002736 mouse GSE4390 sample 389 | 84/368 | 5.39E-06 | 0.000122 |
| Bipolar Disorder C0005586 human GSE5388 sample 107 | 79/341 | 5.68E-06 | 0.000125 |
| Huntington's disease DOID-12858 mouse GSE3583 sample 929 | 70/293 | 6.27E-06 | 0.000135 |
| astrocytoma DOID-3069 human GSE15824 sample 860 | 71/301 | 8.64E-06 | 0.000173 |
| Huntington's disease DOID-12858 human GSE3248 sample 724 | 73/312 | 8.79E-06 | 0.000173 |
| glioblastoma UMLS CUI-C0017636 human GSE15824 sample 862 | 68/285 | 8.84E-06 | 0.000173 |
| chronic lymphocytic leukemia DOID-1040 human GSE6691 sample 786 | 68/285 | 8.84E-06 | 0.000173 |
| Generalized seizures C0234533 rat GSE1156 sample 342 | 74/318 | 9.38E-06 | 0.000179 |
| oligodendroglioma DOID-3181 human GSE15824 sample 858 | 73/313 | 9.89E-06 | 0.000183 |

## Slide 41
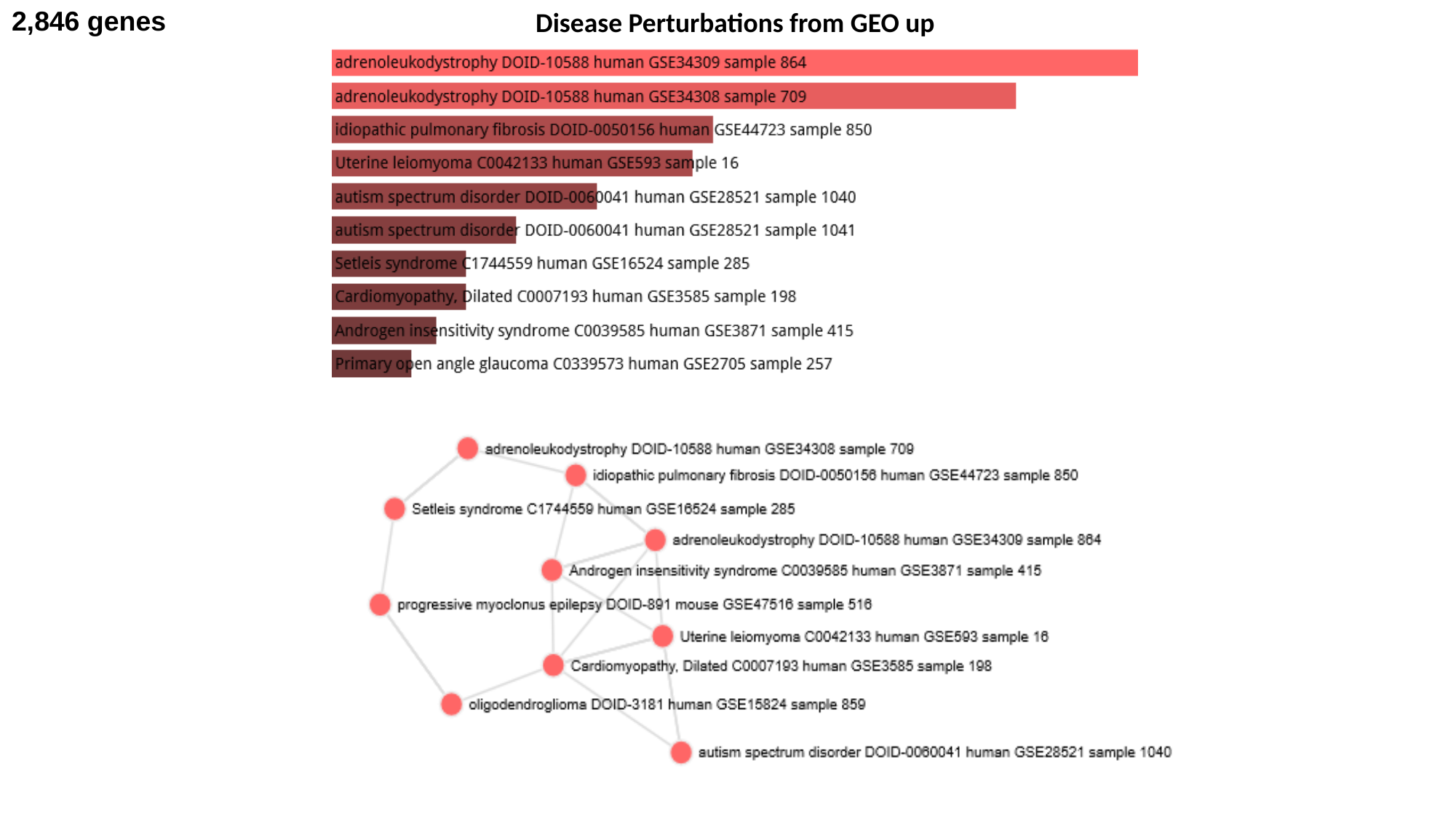

Disease Perturbations from GEO up
2,846 genes

## Slide 42
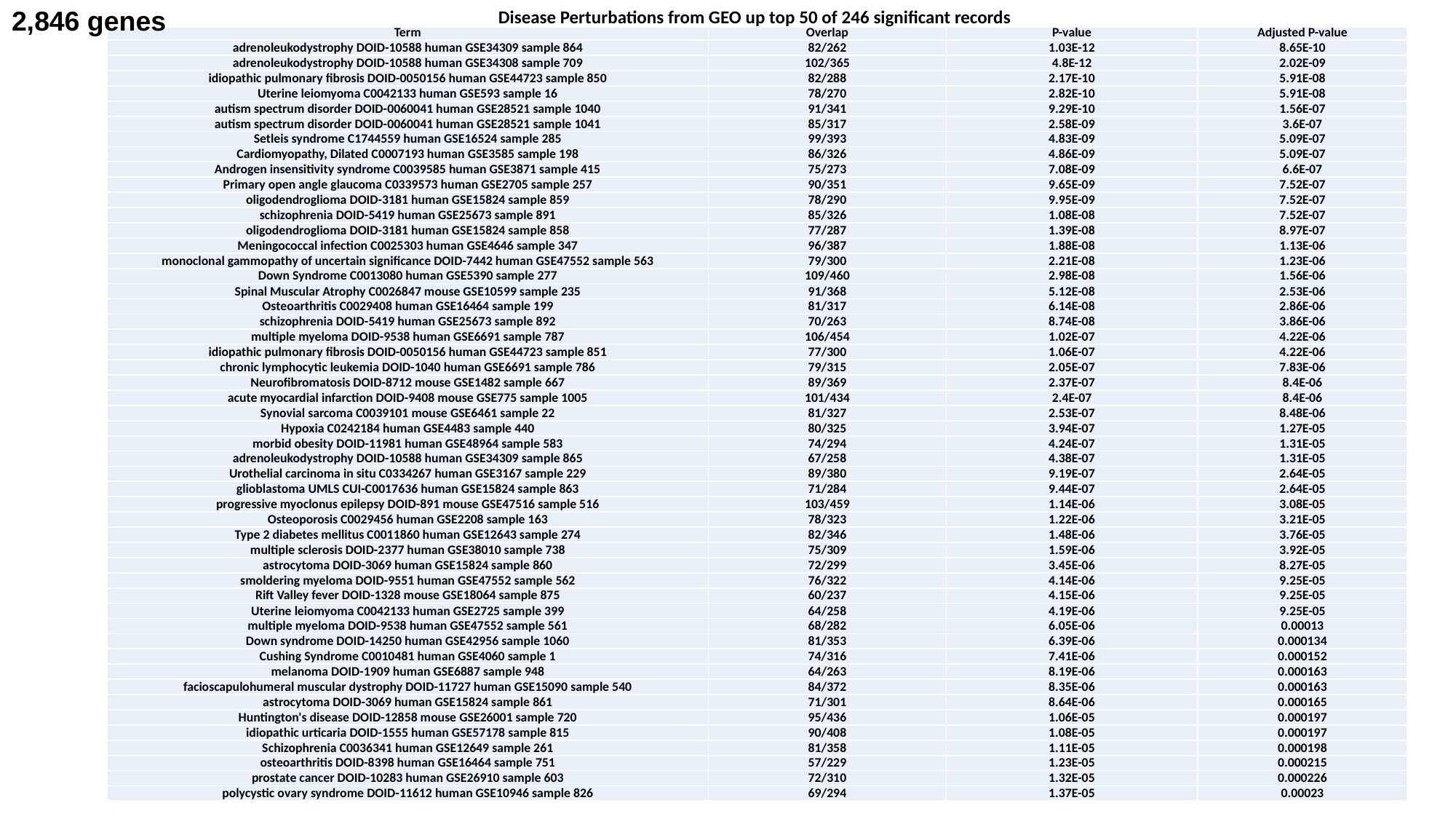

Disease Perturbations from GEO up top 50 of 246 significant records
2,846 genes
| Term | Overlap | P-value | Adjusted P-value |
| --- | --- | --- | --- |
| adrenoleukodystrophy DOID-10588 human GSE34309 sample 864 | 82/262 | 1.03E-12 | 8.65E-10 |
| adrenoleukodystrophy DOID-10588 human GSE34308 sample 709 | 102/365 | 4.8E-12 | 2.02E-09 |
| idiopathic pulmonary fibrosis DOID-0050156 human GSE44723 sample 850 | 82/288 | 2.17E-10 | 5.91E-08 |
| Uterine leiomyoma C0042133 human GSE593 sample 16 | 78/270 | 2.82E-10 | 5.91E-08 |
| autism spectrum disorder DOID-0060041 human GSE28521 sample 1040 | 91/341 | 9.29E-10 | 1.56E-07 |
| autism spectrum disorder DOID-0060041 human GSE28521 sample 1041 | 85/317 | 2.58E-09 | 3.6E-07 |
| Setleis syndrome C1744559 human GSE16524 sample 285 | 99/393 | 4.83E-09 | 5.09E-07 |
| Cardiomyopathy, Dilated C0007193 human GSE3585 sample 198 | 86/326 | 4.86E-09 | 5.09E-07 |
| Androgen insensitivity syndrome C0039585 human GSE3871 sample 415 | 75/273 | 7.08E-09 | 6.6E-07 |
| Primary open angle glaucoma C0339573 human GSE2705 sample 257 | 90/351 | 9.65E-09 | 7.52E-07 |
| oligodendroglioma DOID-3181 human GSE15824 sample 859 | 78/290 | 9.95E-09 | 7.52E-07 |
| schizophrenia DOID-5419 human GSE25673 sample 891 | 85/326 | 1.08E-08 | 7.52E-07 |
| oligodendroglioma DOID-3181 human GSE15824 sample 858 | 77/287 | 1.39E-08 | 8.97E-07 |
| Meningococcal infection C0025303 human GSE4646 sample 347 | 96/387 | 1.88E-08 | 1.13E-06 |
| monoclonal gammopathy of uncertain significance DOID-7442 human GSE47552 sample 563 | 79/300 | 2.21E-08 | 1.23E-06 |
| Down Syndrome C0013080 human GSE5390 sample 277 | 109/460 | 2.98E-08 | 1.56E-06 |
| Spinal Muscular Atrophy C0026847 mouse GSE10599 sample 235 | 91/368 | 5.12E-08 | 2.53E-06 |
| Osteoarthritis C0029408 human GSE16464 sample 199 | 81/317 | 6.14E-08 | 2.86E-06 |
| schizophrenia DOID-5419 human GSE25673 sample 892 | 70/263 | 8.74E-08 | 3.86E-06 |
| multiple myeloma DOID-9538 human GSE6691 sample 787 | 106/454 | 1.02E-07 | 4.22E-06 |
| idiopathic pulmonary fibrosis DOID-0050156 human GSE44723 sample 851 | 77/300 | 1.06E-07 | 4.22E-06 |
| chronic lymphocytic leukemia DOID-1040 human GSE6691 sample 786 | 79/315 | 2.05E-07 | 7.83E-06 |
| Neurofibromatosis DOID-8712 mouse GSE1482 sample 667 | 89/369 | 2.37E-07 | 8.4E-06 |
| acute myocardial infarction DOID-9408 mouse GSE775 sample 1005 | 101/434 | 2.4E-07 | 8.4E-06 |
| Synovial sarcoma C0039101 mouse GSE6461 sample 22 | 81/327 | 2.53E-07 | 8.48E-06 |
| Hypoxia C0242184 human GSE4483 sample 440 | 80/325 | 3.94E-07 | 1.27E-05 |
| morbid obesity DOID-11981 human GSE48964 sample 583 | 74/294 | 4.24E-07 | 1.31E-05 |
| adrenoleukodystrophy DOID-10588 human GSE34309 sample 865 | 67/258 | 4.38E-07 | 1.31E-05 |
| Urothelial carcinoma in situ C0334267 human GSE3167 sample 229 | 89/380 | 9.19E-07 | 2.64E-05 |
| glioblastoma UMLS CUI-C0017636 human GSE15824 sample 863 | 71/284 | 9.44E-07 | 2.64E-05 |
| progressive myoclonus epilepsy DOID-891 mouse GSE47516 sample 516 | 103/459 | 1.14E-06 | 3.08E-05 |
| Osteoporosis C0029456 human GSE2208 sample 163 | 78/323 | 1.22E-06 | 3.21E-05 |
| Type 2 diabetes mellitus C0011860 human GSE12643 sample 274 | 82/346 | 1.48E-06 | 3.76E-05 |
| multiple sclerosis DOID-2377 human GSE38010 sample 738 | 75/309 | 1.59E-06 | 3.92E-05 |
| astrocytoma DOID-3069 human GSE15824 sample 860 | 72/299 | 3.45E-06 | 8.27E-05 |
| smoldering myeloma DOID-9551 human GSE47552 sample 562 | 76/322 | 4.14E-06 | 9.25E-05 |
| Rift Valley fever DOID-1328 mouse GSE18064 sample 875 | 60/237 | 4.15E-06 | 9.25E-05 |
| Uterine leiomyoma C0042133 human GSE2725 sample 399 | 64/258 | 4.19E-06 | 9.25E-05 |
| multiple myeloma DOID-9538 human GSE47552 sample 561 | 68/282 | 6.05E-06 | 0.00013 |
| Down syndrome DOID-14250 human GSE42956 sample 1060 | 81/353 | 6.39E-06 | 0.000134 |
| Cushing Syndrome C0010481 human GSE4060 sample 1 | 74/316 | 7.41E-06 | 0.000152 |
| melanoma DOID-1909 human GSE6887 sample 948 | 64/263 | 8.19E-06 | 0.000163 |
| facioscapulohumeral muscular dystrophy DOID-11727 human GSE15090 sample 540 | 84/372 | 8.35E-06 | 0.000163 |
| astrocytoma DOID-3069 human GSE15824 sample 861 | 71/301 | 8.64E-06 | 0.000165 |
| Huntington's disease DOID-12858 mouse GSE26001 sample 720 | 95/436 | 1.06E-05 | 0.000197 |
| idiopathic urticaria DOID-1555 human GSE57178 sample 815 | 90/408 | 1.08E-05 | 0.000197 |
| Schizophrenia C0036341 human GSE12649 sample 261 | 81/358 | 1.11E-05 | 0.000198 |
| osteoarthritis DOID-8398 human GSE16464 sample 751 | 57/229 | 1.23E-05 | 0.000215 |
| prostate cancer DOID-10283 human GSE26910 sample 603 | 72/310 | 1.32E-05 | 0.000226 |
| polycystic ovary syndrome DOID-11612 human GSE10946 sample 826 | 69/294 | 1.37E-05 | 0.00023 |

## Slide 43
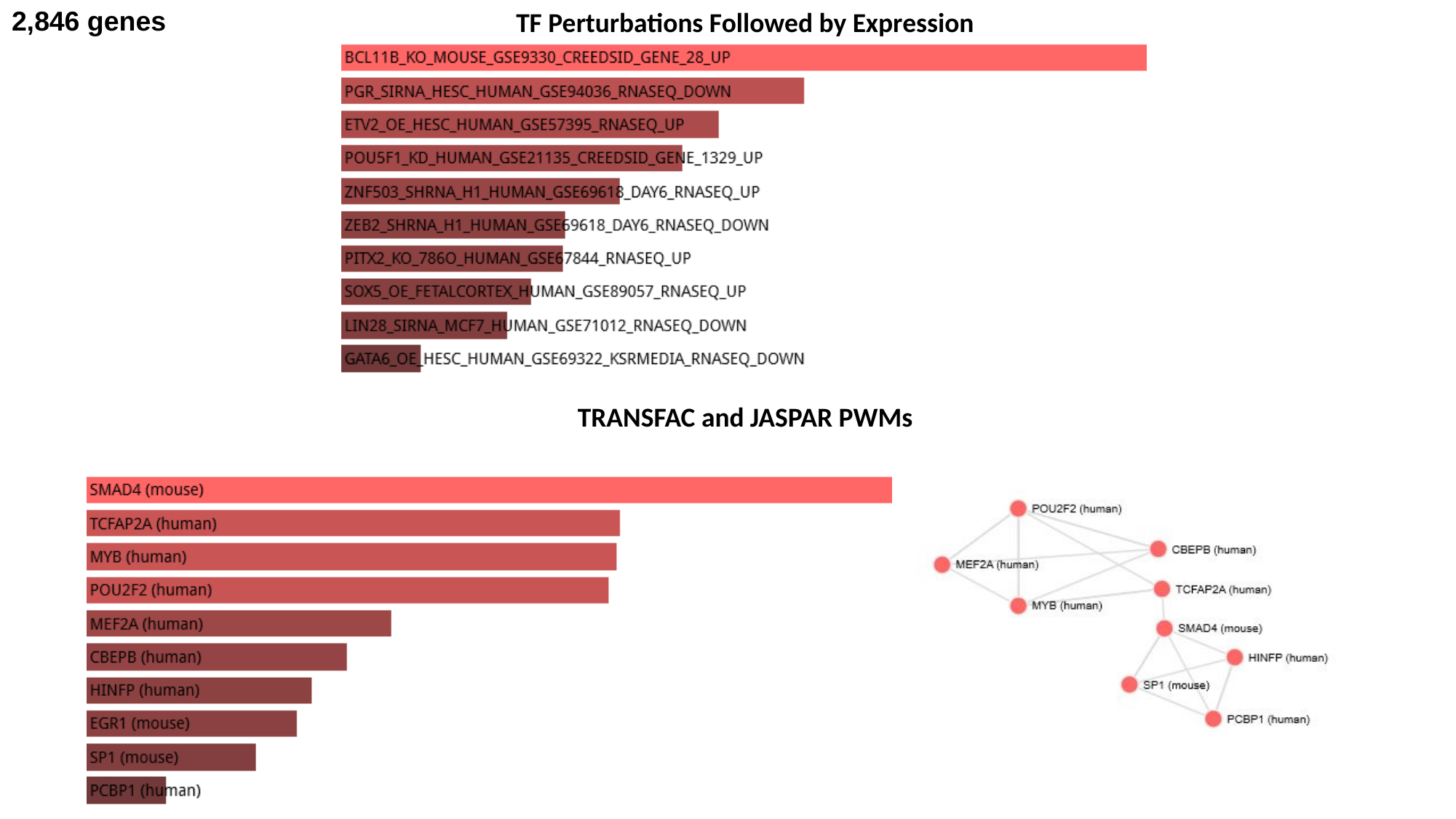

TF Perturbations Followed by Expression
2,846 genes
TRANSFAC and JASPAR PWMs

## Slide 44
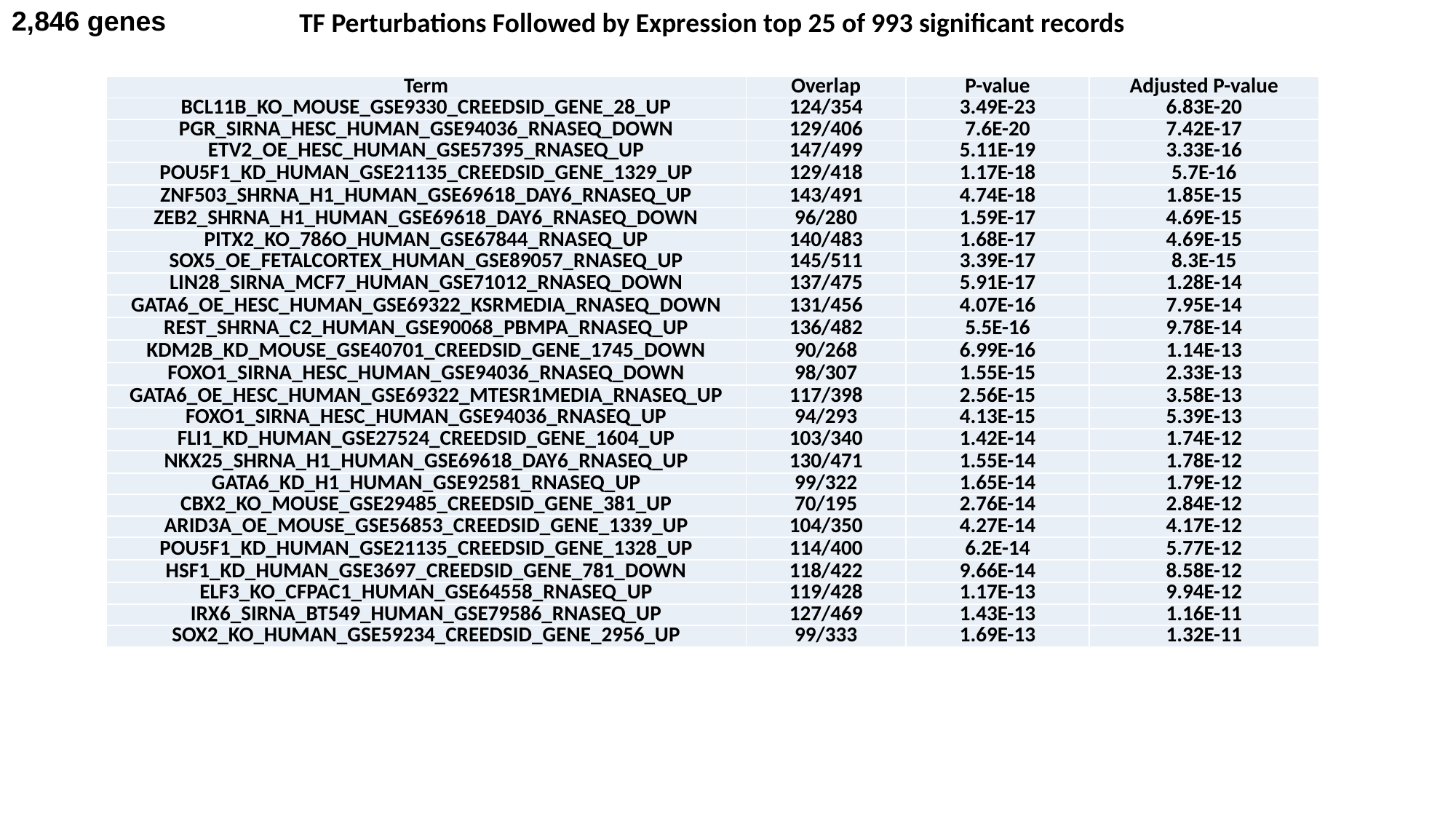

TF Perturbations Followed by Expression top 25 of 993 significant records
2,846 genes
| Term | Overlap | P-value | Adjusted P-value |
| --- | --- | --- | --- |
| BCL11B\_KO\_MOUSE\_GSE9330\_CREEDSID\_GENE\_28\_UP | 124/354 | 3.49E-23 | 6.83E-20 |
| PGR\_SIRNA\_HESC\_HUMAN\_GSE94036\_RNASEQ\_DOWN | 129/406 | 7.6E-20 | 7.42E-17 |
| ETV2\_OE\_HESC\_HUMAN\_GSE57395\_RNASEQ\_UP | 147/499 | 5.11E-19 | 3.33E-16 |
| POU5F1\_KD\_HUMAN\_GSE21135\_CREEDSID\_GENE\_1329\_UP | 129/418 | 1.17E-18 | 5.7E-16 |
| ZNF503\_SHRNA\_H1\_HUMAN\_GSE69618\_DAY6\_RNASEQ\_UP | 143/491 | 4.74E-18 | 1.85E-15 |
| ZEB2\_SHRNA\_H1\_HUMAN\_GSE69618\_DAY6\_RNASEQ\_DOWN | 96/280 | 1.59E-17 | 4.69E-15 |
| PITX2\_KO\_786O\_HUMAN\_GSE67844\_RNASEQ\_UP | 140/483 | 1.68E-17 | 4.69E-15 |
| SOX5\_OE\_FETALCORTEX\_HUMAN\_GSE89057\_RNASEQ\_UP | 145/511 | 3.39E-17 | 8.3E-15 |
| LIN28\_SIRNA\_MCF7\_HUMAN\_GSE71012\_RNASEQ\_DOWN | 137/475 | 5.91E-17 | 1.28E-14 |
| GATA6\_OE\_HESC\_HUMAN\_GSE69322\_KSRMEDIA\_RNASEQ\_DOWN | 131/456 | 4.07E-16 | 7.95E-14 |
| REST\_SHRNA\_C2\_HUMAN\_GSE90068\_PBMPA\_RNASEQ\_UP | 136/482 | 5.5E-16 | 9.78E-14 |
| KDM2B\_KD\_MOUSE\_GSE40701\_CREEDSID\_GENE\_1745\_DOWN | 90/268 | 6.99E-16 | 1.14E-13 |
| FOXO1\_SIRNA\_HESC\_HUMAN\_GSE94036\_RNASEQ\_DOWN | 98/307 | 1.55E-15 | 2.33E-13 |
| GATA6\_OE\_HESC\_HUMAN\_GSE69322\_MTESR1MEDIA\_RNASEQ\_UP | 117/398 | 2.56E-15 | 3.58E-13 |
| FOXO1\_SIRNA\_HESC\_HUMAN\_GSE94036\_RNASEQ\_UP | 94/293 | 4.13E-15 | 5.39E-13 |
| FLI1\_KD\_HUMAN\_GSE27524\_CREEDSID\_GENE\_1604\_UP | 103/340 | 1.42E-14 | 1.74E-12 |
| NKX25\_SHRNA\_H1\_HUMAN\_GSE69618\_DAY6\_RNASEQ\_UP | 130/471 | 1.55E-14 | 1.78E-12 |
| GATA6\_KD\_H1\_HUMAN\_GSE92581\_RNASEQ\_UP | 99/322 | 1.65E-14 | 1.79E-12 |
| CBX2\_KO\_MOUSE\_GSE29485\_CREEDSID\_GENE\_381\_UP | 70/195 | 2.76E-14 | 2.84E-12 |
| ARID3A\_OE\_MOUSE\_GSE56853\_CREEDSID\_GENE\_1339\_UP | 104/350 | 4.27E-14 | 4.17E-12 |
| POU5F1\_KD\_HUMAN\_GSE21135\_CREEDSID\_GENE\_1328\_UP | 114/400 | 6.2E-14 | 5.77E-12 |
| HSF1\_KD\_HUMAN\_GSE3697\_CREEDSID\_GENE\_781\_DOWN | 118/422 | 9.66E-14 | 8.58E-12 |
| ELF3\_KO\_CFPAC1\_HUMAN\_GSE64558\_RNASEQ\_UP | 119/428 | 1.17E-13 | 9.94E-12 |
| IRX6\_SIRNA\_BT549\_HUMAN\_GSE79586\_RNASEQ\_UP | 127/469 | 1.43E-13 | 1.16E-11 |
| SOX2\_KO\_HUMAN\_GSE59234\_CREEDSID\_GENE\_2956\_UP | 99/333 | 1.69E-13 | 1.32E-11 |

## Slide 45
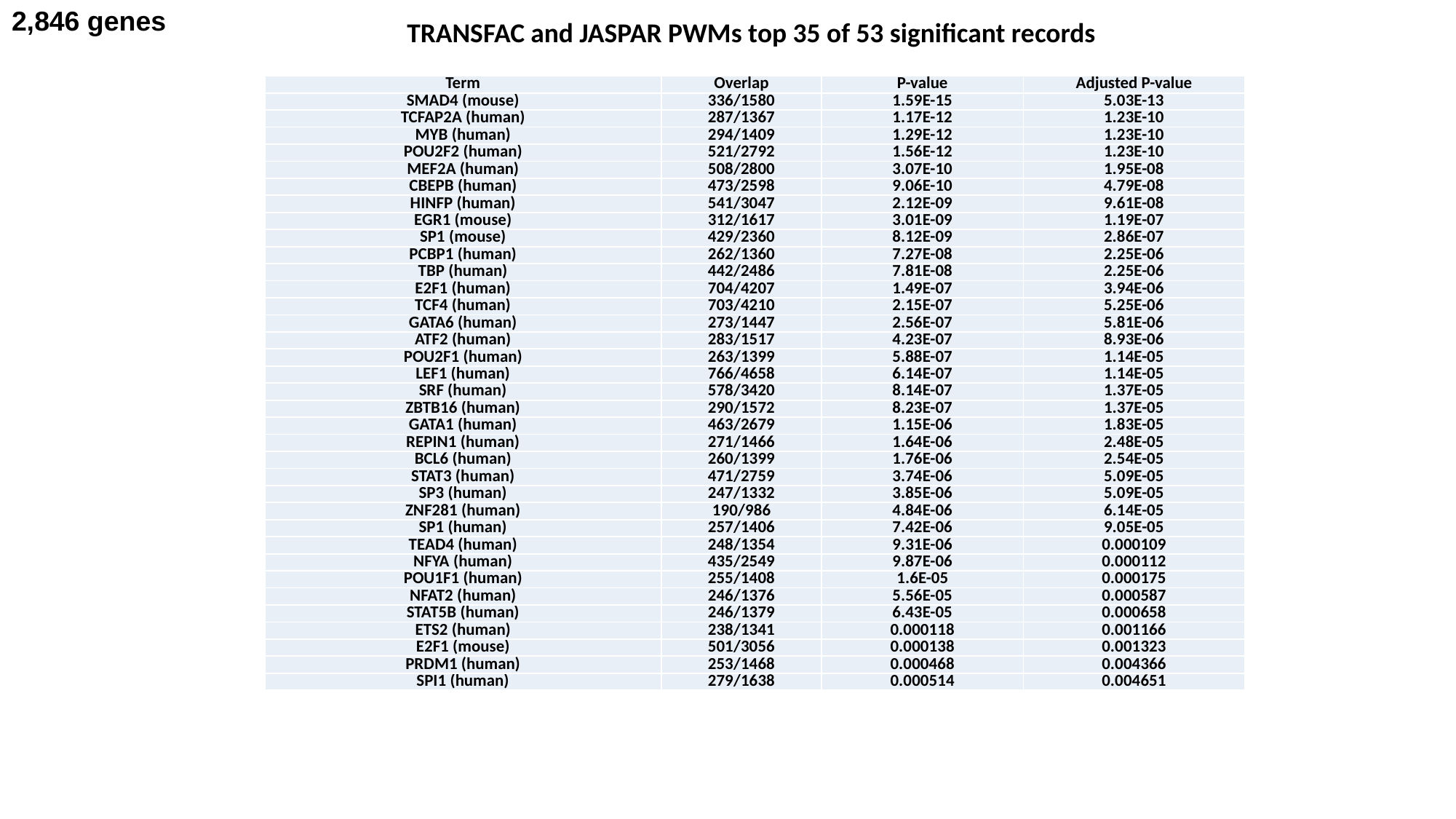

2,846 genes
TRANSFAC and JASPAR PWMs top 35 of 53 significant records
| Term | Overlap | P-value | Adjusted P-value |
| --- | --- | --- | --- |
| SMAD4 (mouse) | 336/1580 | 1.59E-15 | 5.03E-13 |
| TCFAP2A (human) | 287/1367 | 1.17E-12 | 1.23E-10 |
| MYB (human) | 294/1409 | 1.29E-12 | 1.23E-10 |
| POU2F2 (human) | 521/2792 | 1.56E-12 | 1.23E-10 |
| MEF2A (human) | 508/2800 | 3.07E-10 | 1.95E-08 |
| CBEPB (human) | 473/2598 | 9.06E-10 | 4.79E-08 |
| HINFP (human) | 541/3047 | 2.12E-09 | 9.61E-08 |
| EGR1 (mouse) | 312/1617 | 3.01E-09 | 1.19E-07 |
| SP1 (mouse) | 429/2360 | 8.12E-09 | 2.86E-07 |
| PCBP1 (human) | 262/1360 | 7.27E-08 | 2.25E-06 |
| TBP (human) | 442/2486 | 7.81E-08 | 2.25E-06 |
| E2F1 (human) | 704/4207 | 1.49E-07 | 3.94E-06 |
| TCF4 (human) | 703/4210 | 2.15E-07 | 5.25E-06 |
| GATA6 (human) | 273/1447 | 2.56E-07 | 5.81E-06 |
| ATF2 (human) | 283/1517 | 4.23E-07 | 8.93E-06 |
| POU2F1 (human) | 263/1399 | 5.88E-07 | 1.14E-05 |
| LEF1 (human) | 766/4658 | 6.14E-07 | 1.14E-05 |
| SRF (human) | 578/3420 | 8.14E-07 | 1.37E-05 |
| ZBTB16 (human) | 290/1572 | 8.23E-07 | 1.37E-05 |
| GATA1 (human) | 463/2679 | 1.15E-06 | 1.83E-05 |
| REPIN1 (human) | 271/1466 | 1.64E-06 | 2.48E-05 |
| BCL6 (human) | 260/1399 | 1.76E-06 | 2.54E-05 |
| STAT3 (human) | 471/2759 | 3.74E-06 | 5.09E-05 |
| SP3 (human) | 247/1332 | 3.85E-06 | 5.09E-05 |
| ZNF281 (human) | 190/986 | 4.84E-06 | 6.14E-05 |
| SP1 (human) | 257/1406 | 7.42E-06 | 9.05E-05 |
| TEAD4 (human) | 248/1354 | 9.31E-06 | 0.000109 |
| NFYA (human) | 435/2549 | 9.87E-06 | 0.000112 |
| POU1F1 (human) | 255/1408 | 1.6E-05 | 0.000175 |
| NFAT2 (human) | 246/1376 | 5.56E-05 | 0.000587 |
| STAT5B (human) | 246/1379 | 6.43E-05 | 0.000658 |
| ETS2 (human) | 238/1341 | 0.000118 | 0.001166 |
| E2F1 (mouse) | 501/3056 | 0.000138 | 0.001323 |
| PRDM1 (human) | 253/1468 | 0.000468 | 0.004366 |
| SPI1 (human) | 279/1638 | 0.000514 | 0.004651 |

## Slide 46
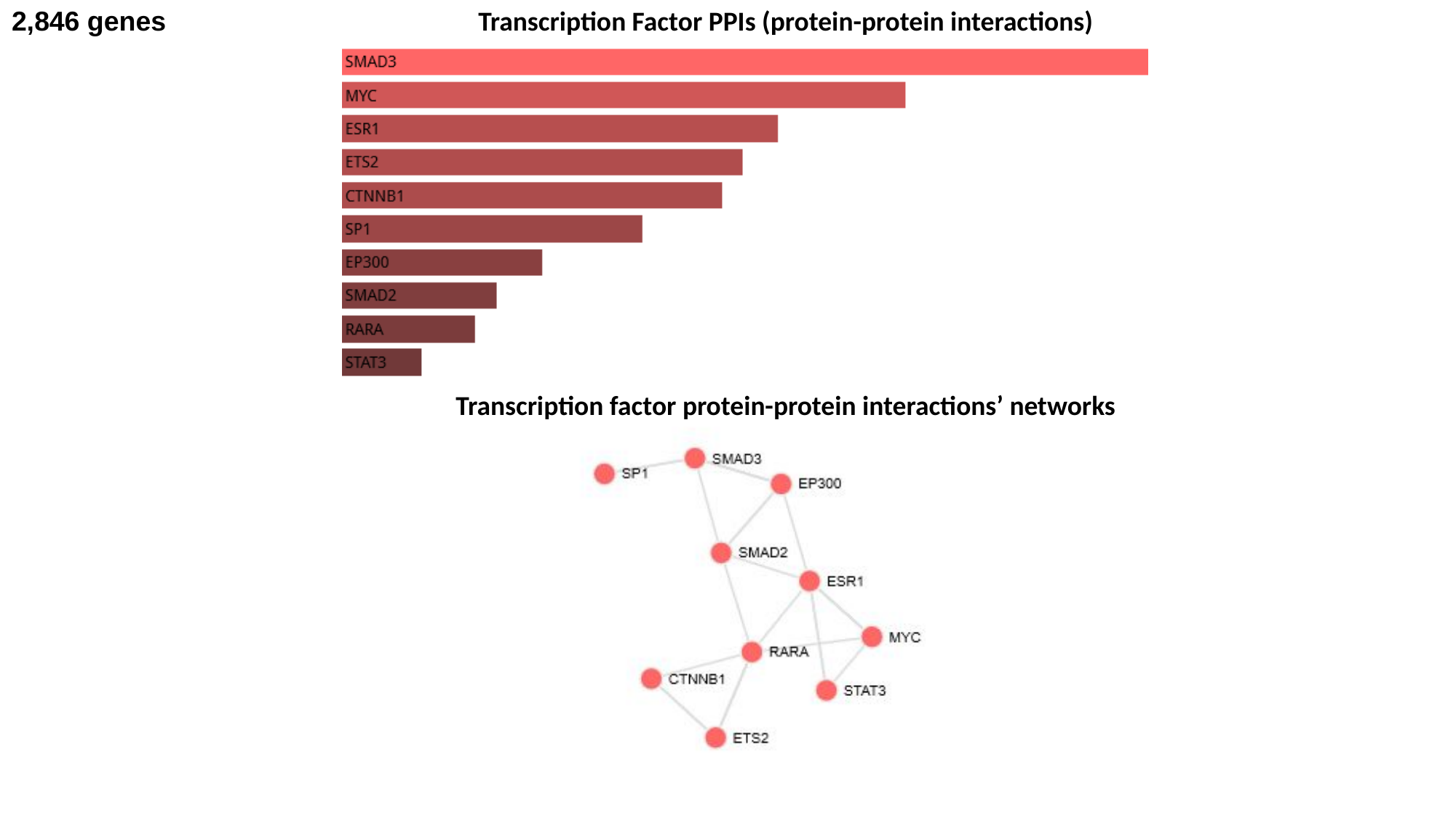

2,846 genes
Transcription Factor PPIs (protein-protein interactions)
Transcription factor protein-protein interactions’ networks

## Slide 47
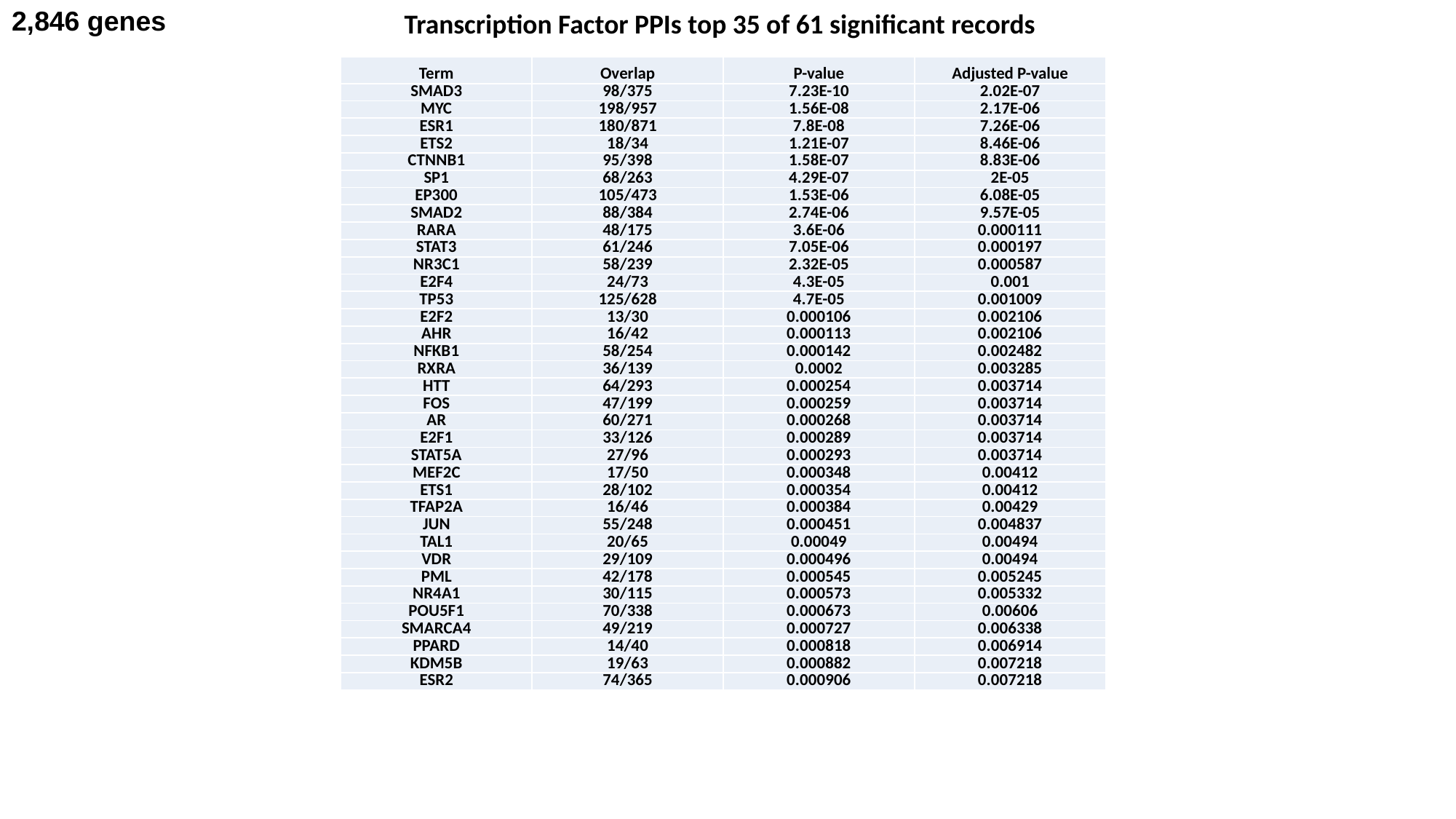

2,846 genes
Transcription Factor PPIs top 35 of 61 significant records
| Term | Overlap | P-value | Adjusted P-value |
| --- | --- | --- | --- |
| SMAD3 | 98/375 | 7.23E-10 | 2.02E-07 |
| MYC | 198/957 | 1.56E-08 | 2.17E-06 |
| ESR1 | 180/871 | 7.8E-08 | 7.26E-06 |
| ETS2 | 18/34 | 1.21E-07 | 8.46E-06 |
| CTNNB1 | 95/398 | 1.58E-07 | 8.83E-06 |
| SP1 | 68/263 | 4.29E-07 | 2E-05 |
| EP300 | 105/473 | 1.53E-06 | 6.08E-05 |
| SMAD2 | 88/384 | 2.74E-06 | 9.57E-05 |
| RARA | 48/175 | 3.6E-06 | 0.000111 |
| STAT3 | 61/246 | 7.05E-06 | 0.000197 |
| NR3C1 | 58/239 | 2.32E-05 | 0.000587 |
| E2F4 | 24/73 | 4.3E-05 | 0.001 |
| TP53 | 125/628 | 4.7E-05 | 0.001009 |
| E2F2 | 13/30 | 0.000106 | 0.002106 |
| AHR | 16/42 | 0.000113 | 0.002106 |
| NFKB1 | 58/254 | 0.000142 | 0.002482 |
| RXRA | 36/139 | 0.0002 | 0.003285 |
| HTT | 64/293 | 0.000254 | 0.003714 |
| FOS | 47/199 | 0.000259 | 0.003714 |
| AR | 60/271 | 0.000268 | 0.003714 |
| E2F1 | 33/126 | 0.000289 | 0.003714 |
| STAT5A | 27/96 | 0.000293 | 0.003714 |
| MEF2C | 17/50 | 0.000348 | 0.00412 |
| ETS1 | 28/102 | 0.000354 | 0.00412 |
| TFAP2A | 16/46 | 0.000384 | 0.00429 |
| JUN | 55/248 | 0.000451 | 0.004837 |
| TAL1 | 20/65 | 0.00049 | 0.00494 |
| VDR | 29/109 | 0.000496 | 0.00494 |
| PML | 42/178 | 0.000545 | 0.005245 |
| NR4A1 | 30/115 | 0.000573 | 0.005332 |
| POU5F1 | 70/338 | 0.000673 | 0.00606 |
| SMARCA4 | 49/219 | 0.000727 | 0.006338 |
| PPARD | 14/40 | 0.000818 | 0.006914 |
| KDM5B | 19/63 | 0.000882 | 0.007218 |
| ESR2 | 74/365 | 0.000906 | 0.007218 |
