# Supplementary material for: Genomics-Guided Drawing of Molecular and Pathophysiological Components of Malignant Regulatory Signatures Reveals a Pivotal Role in Human Diseases of Stem Cell-Associated Retroviral Sequences and Functionally-Active hESC Enhancers
Source: Front Oncol. 2021 Mar 31;11:638363. doi: 10.3389/fonc.2021.638363 (PMC8044830; doi:10.3389/fonc.2021.638363)
Supplement: Supplementary file 1 [file Presentation_1.zip › Supplemental Note S4. HSRS SCARS CA drivers and survival genes.pptx]

## Slide 1
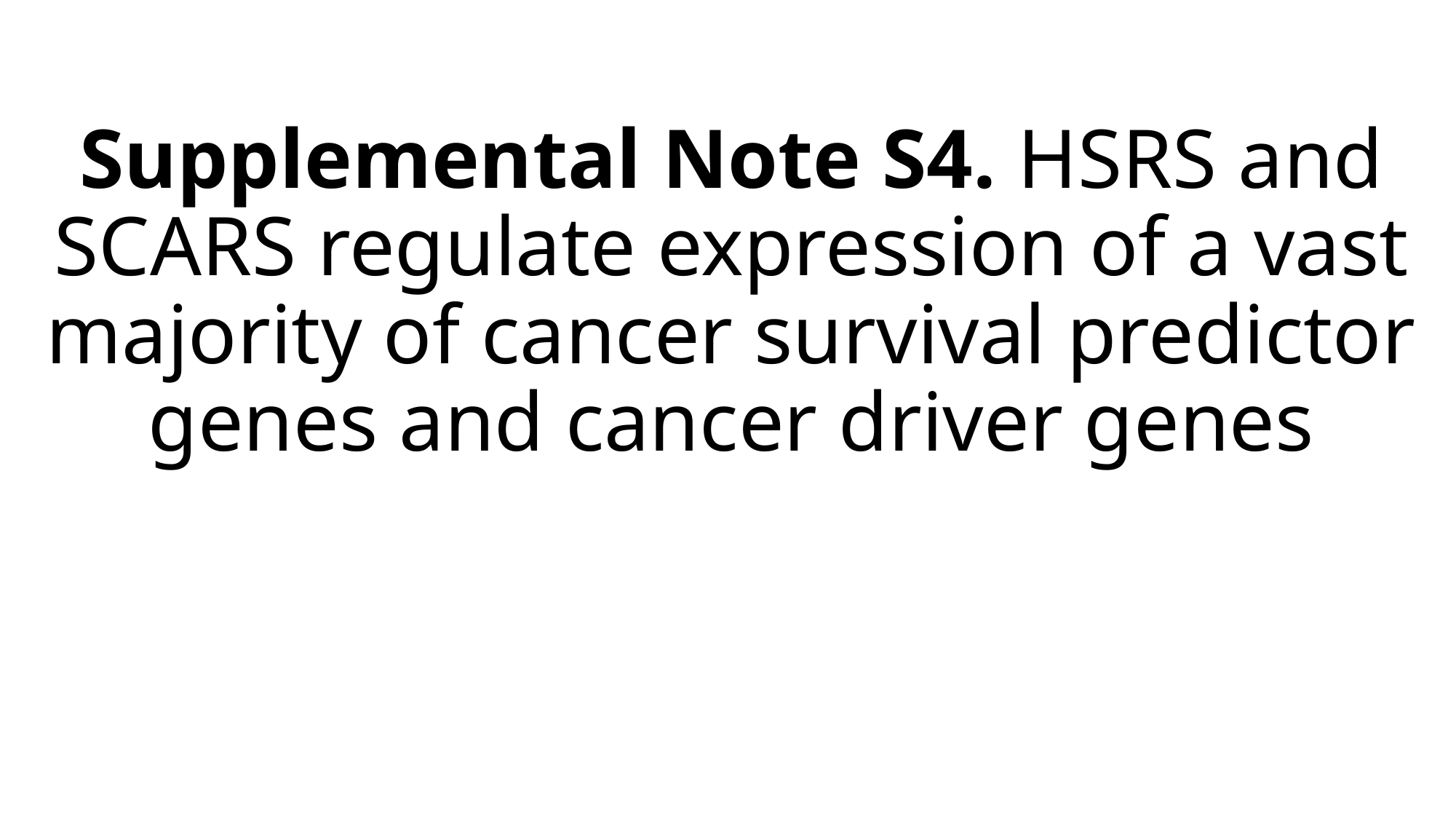

# Supplemental Note S4. HSRS and SCARS regulate expression of a vast majority of cancer survival predictor genes and cancer driver genes

## Slide 2
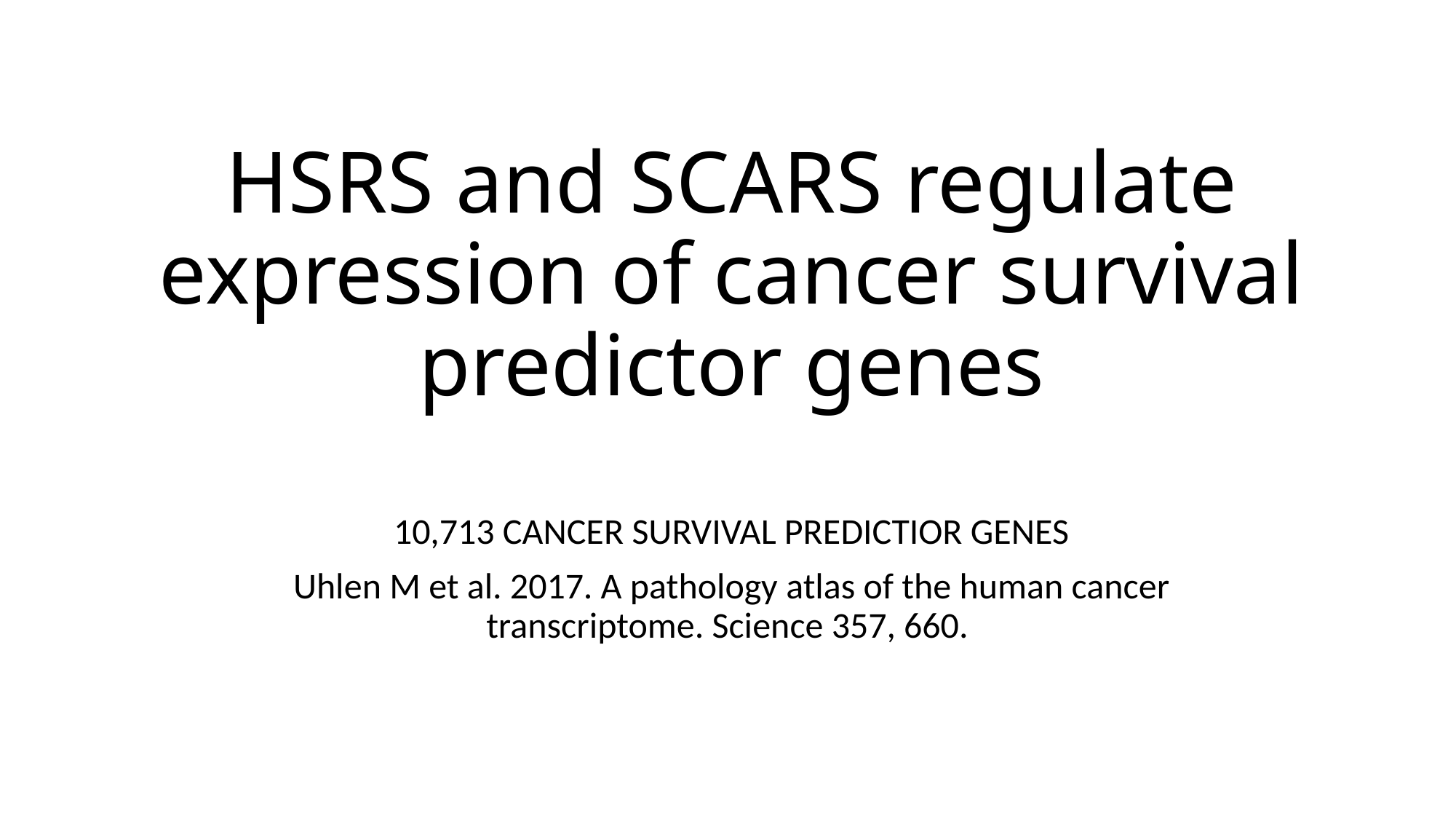

# HSRS and SCARS regulate expression of cancer survival predictor genes
10,713 CANCER SURVIVAL PREDICTIOR GENES
Uhlen M et al. 2017. A pathology atlas of the human cancer transcriptome. Science 357, 660.

## Slide 3
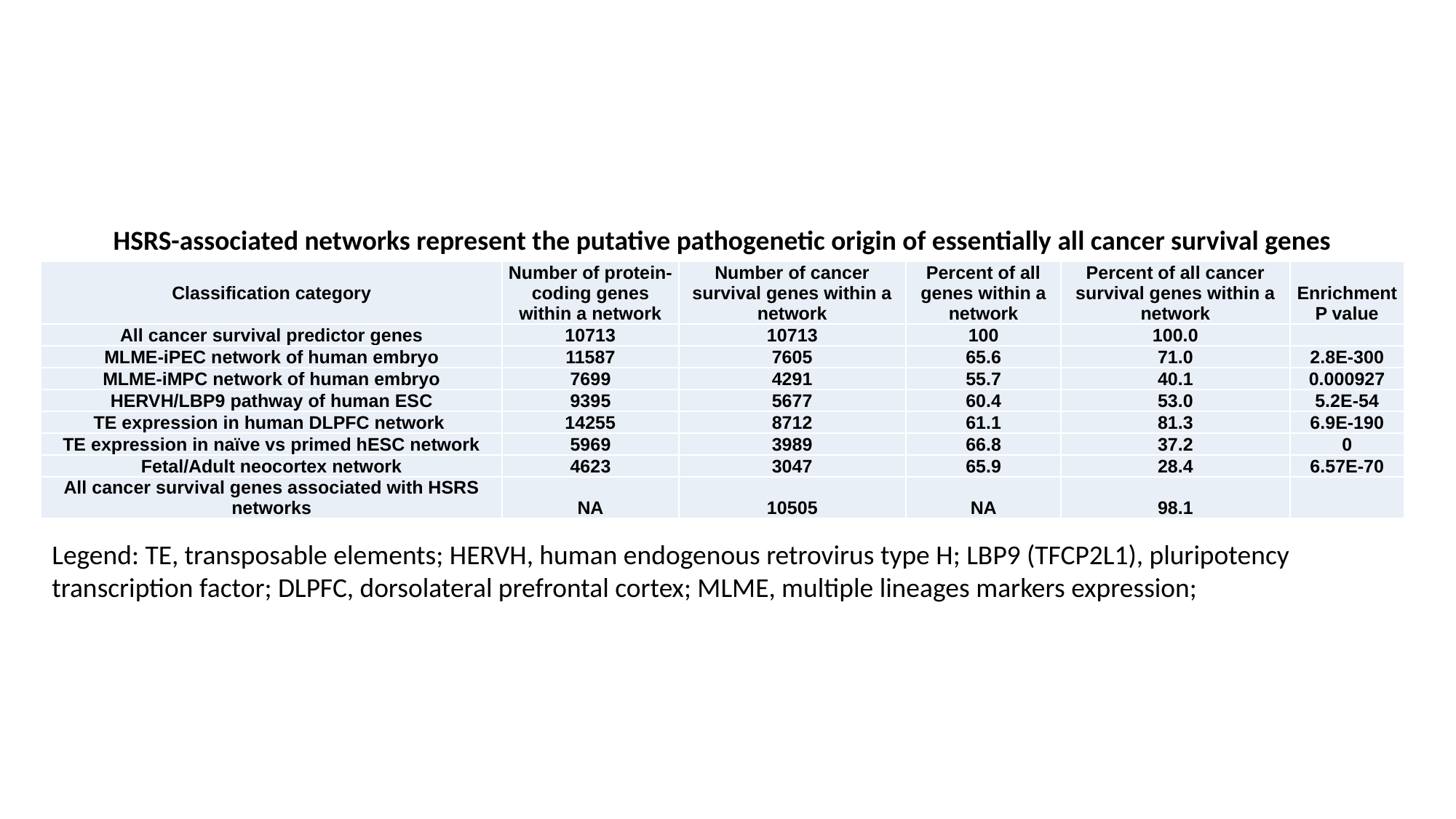

HSRS-associated networks represent the putative pathogenetic origin of essentially all cancer survival genes
| Classification category | Number of protein-coding genes within a network | Number of cancer survival genes within a network | Percent of all genes within a network | Percent of all cancer survival genes within a network | Enrichment P value |
| --- | --- | --- | --- | --- | --- |
| All cancer survival predictor genes | 10713 | 10713 | 100 | 100.0 | |
| MLME-iPEC network of human embryo | 11587 | 7605 | 65.6 | 71.0 | 2.8E-300 |
| MLME-iMPC network of human embryo | 7699 | 4291 | 55.7 | 40.1 | 0.000927 |
| HERVH/LBP9 pathway of human ESC | 9395 | 5677 | 60.4 | 53.0 | 5.2E-54 |
| TE expression in human DLPFC network | 14255 | 8712 | 61.1 | 81.3 | 6.9E-190 |
| TE expression in naïve vs primed hESC network | 5969 | 3989 | 66.8 | 37.2 | 0 |
| Fetal/Adult neocortex network | 4623 | 3047 | 65.9 | 28.4 | 6.57E-70 |
| All cancer survival genes associated with HSRS networks | NA | 10505 | NA | 98.1 | |
Legend: TE, transposable elements; HERVH, human endogenous retrovirus type H; LBP9 (TFCP2L1), pluripotency transcription factor; DLPFC, dorsolateral prefrontal cortex; MLME, multiple lineages markers expression;

## Slide 4
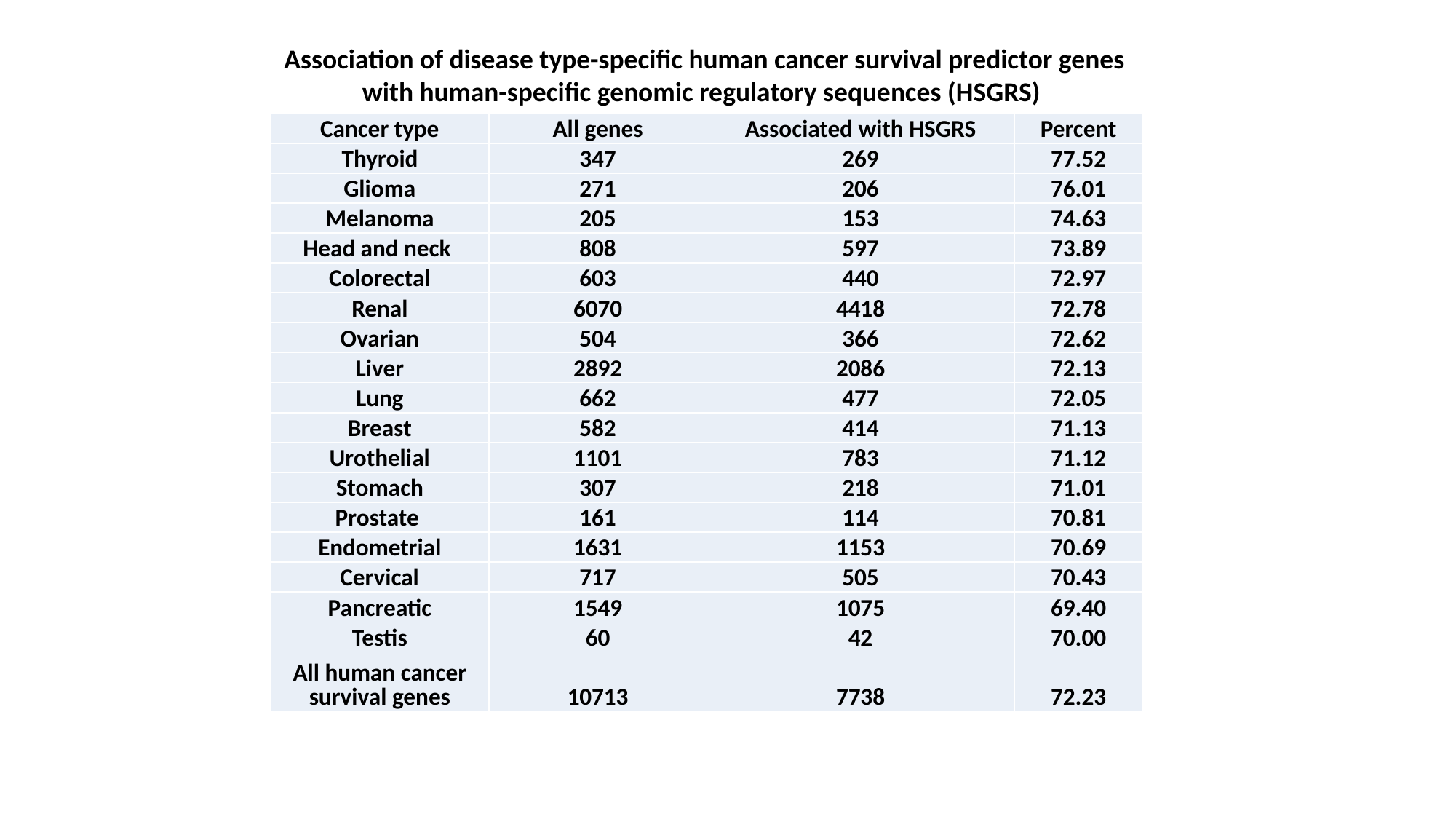

Association of disease type-specific human cancer survival predictor genes with human-specific genomic regulatory sequences (HSGRS)
| Cancer type | All genes | Associated with HSGRS | Percent |
| --- | --- | --- | --- |
| Thyroid | 347 | 269 | 77.52 |
| Glioma | 271 | 206 | 76.01 |
| Melanoma | 205 | 153 | 74.63 |
| Head and neck | 808 | 597 | 73.89 |
| Colorectal | 603 | 440 | 72.97 |
| Renal | 6070 | 4418 | 72.78 |
| Ovarian | 504 | 366 | 72.62 |
| Liver | 2892 | 2086 | 72.13 |
| Lung | 662 | 477 | 72.05 |
| Breast | 582 | 414 | 71.13 |
| Urothelial | 1101 | 783 | 71.12 |
| Stomach | 307 | 218 | 71.01 |
| Prostate | 161 | 114 | 70.81 |
| Endometrial | 1631 | 1153 | 70.69 |
| Cervical | 717 | 505 | 70.43 |
| Pancreatic | 1549 | 1075 | 69.40 |
| Testis | 60 | 42 | 70.00 |
| All human cancer survival genes | 10713 | 7738 | 72.23 |

## Slide 5
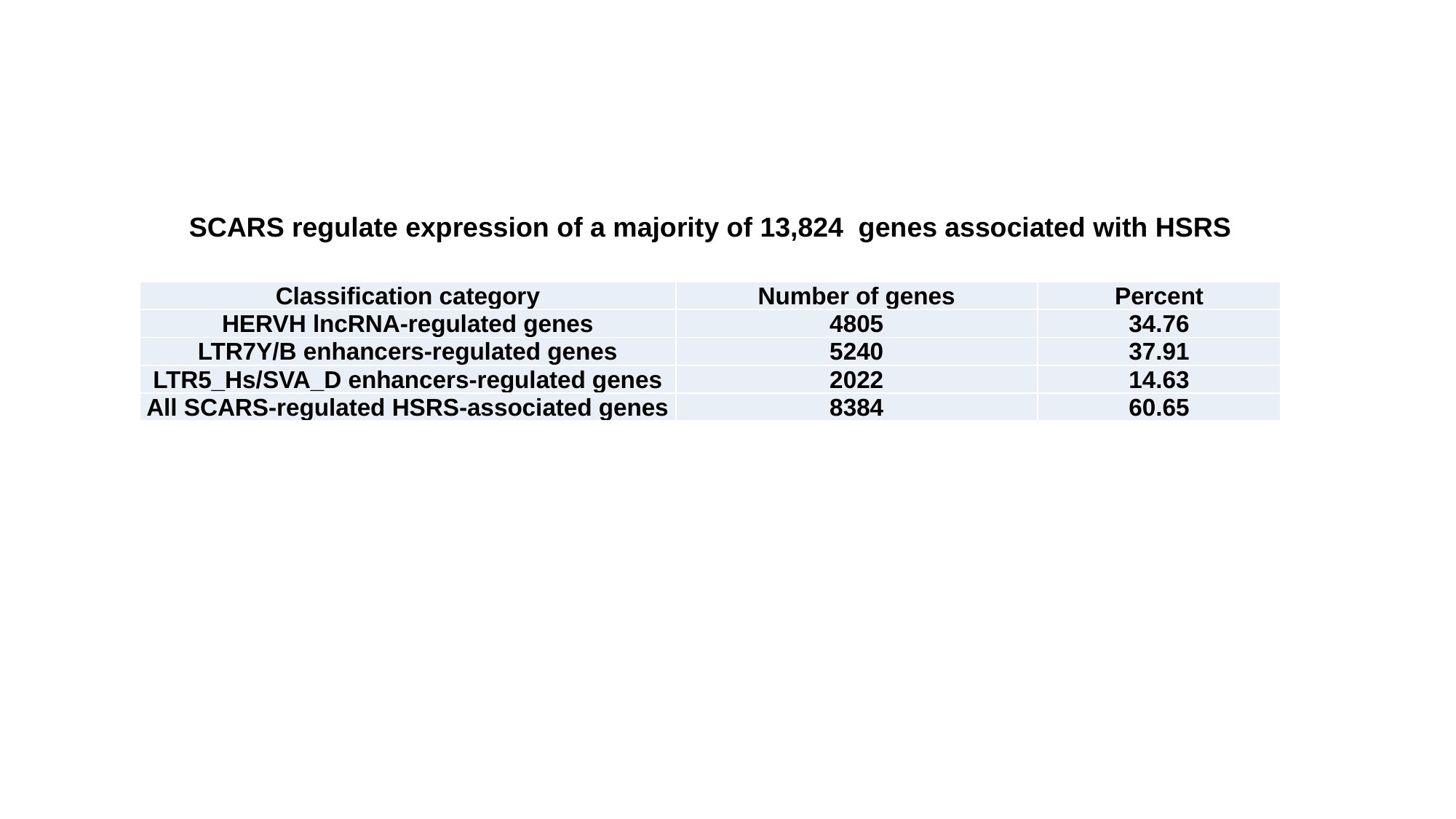

SCARS regulate expression of a majority of 13,824 genes associated with HSRS
| Classification category | Number of genes | Percent |
| --- | --- | --- |
| HERVH lncRNA-regulated genes | 4805 | 34.76 |
| LTR7Y/B enhancers-regulated genes | 5240 | 37.91 |
| LTR5\_Hs/SVA\_D enhancers-regulated genes | 2022 | 14.63 |
| All SCARS-regulated HSRS-associated genes | 8384 | 60.65 |

## Slide 6
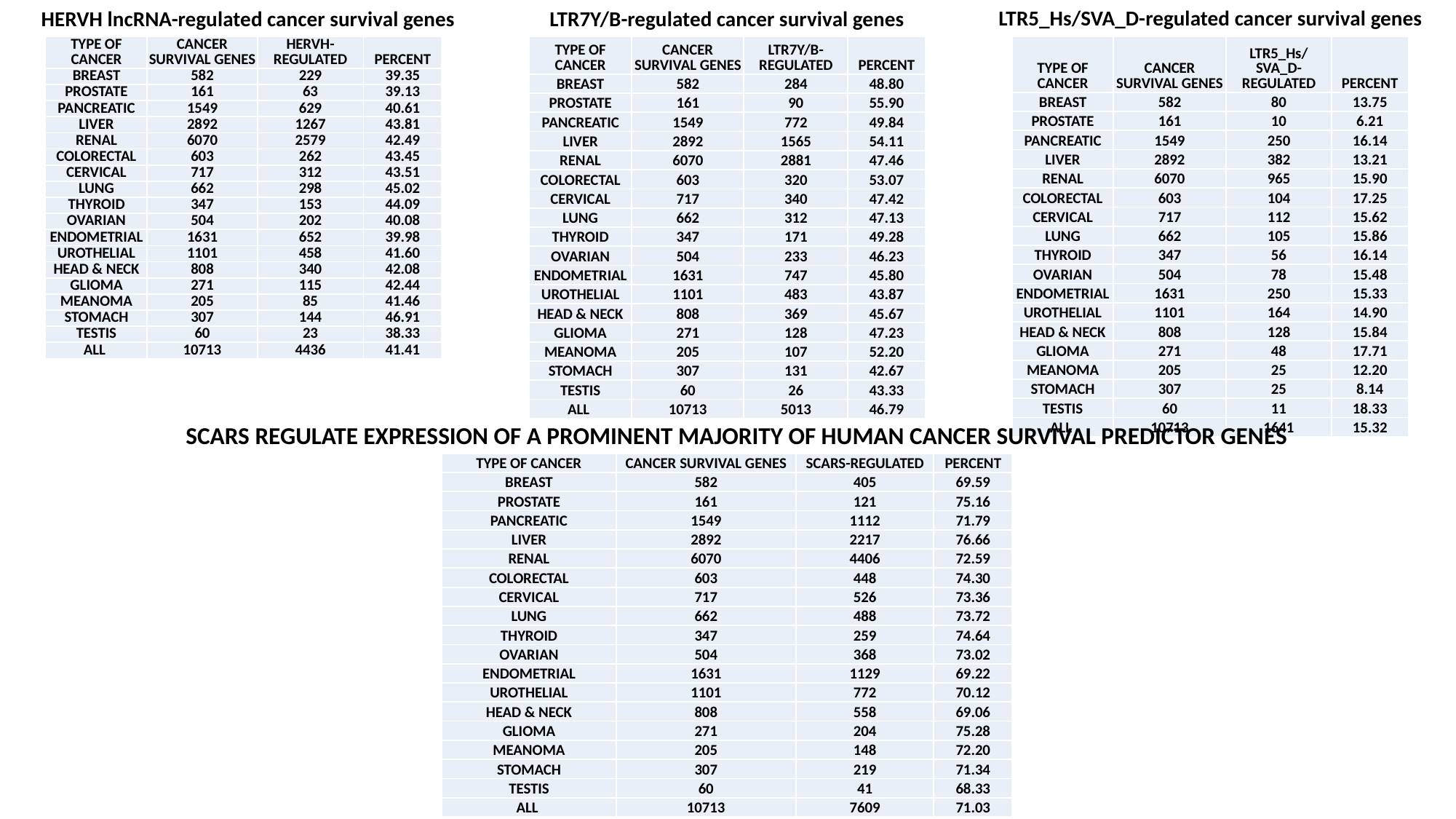

LTR5_Hs/SVA_D-regulated cancer survival genes
HERVH lncRNA-regulated cancer survival genes
LTR7Y/B-regulated cancer survival genes
| TYPE OF CANCER | CANCER SURVIVAL GENES | LTR7Y/B-REGULATED | PERCENT |
| --- | --- | --- | --- |
| BREAST | 582 | 284 | 48.80 |
| PROSTATE | 161 | 90 | 55.90 |
| PANCREATIC | 1549 | 772 | 49.84 |
| LIVER | 2892 | 1565 | 54.11 |
| RENAL | 6070 | 2881 | 47.46 |
| COLORECTAL | 603 | 320 | 53.07 |
| CERVICAL | 717 | 340 | 47.42 |
| LUNG | 662 | 312 | 47.13 |
| THYROID | 347 | 171 | 49.28 |
| OVARIAN | 504 | 233 | 46.23 |
| ENDOMETRIAL | 1631 | 747 | 45.80 |
| UROTHELIAL | 1101 | 483 | 43.87 |
| HEAD & NECK | 808 | 369 | 45.67 |
| GLIOMA | 271 | 128 | 47.23 |
| MEANOMA | 205 | 107 | 52.20 |
| STOMACH | 307 | 131 | 42.67 |
| TESTIS | 60 | 26 | 43.33 |
| ALL | 10713 | 5013 | 46.79 |
| TYPE OF CANCER | CANCER SURVIVAL GENES | LTR5\_Hs/SVA\_D-REGULATED | PERCENT |
| --- | --- | --- | --- |
| BREAST | 582 | 80 | 13.75 |
| PROSTATE | 161 | 10 | 6.21 |
| PANCREATIC | 1549 | 250 | 16.14 |
| LIVER | 2892 | 382 | 13.21 |
| RENAL | 6070 | 965 | 15.90 |
| COLORECTAL | 603 | 104 | 17.25 |
| CERVICAL | 717 | 112 | 15.62 |
| LUNG | 662 | 105 | 15.86 |
| THYROID | 347 | 56 | 16.14 |
| OVARIAN | 504 | 78 | 15.48 |
| ENDOMETRIAL | 1631 | 250 | 15.33 |
| UROTHELIAL | 1101 | 164 | 14.90 |
| HEAD & NECK | 808 | 128 | 15.84 |
| GLIOMA | 271 | 48 | 17.71 |
| MEANOMA | 205 | 25 | 12.20 |
| STOMACH | 307 | 25 | 8.14 |
| TESTIS | 60 | 11 | 18.33 |
| ALL | 10713 | 1641 | 15.32 |
| TYPE OF CANCER | CANCER SURVIVAL GENES | HERVH-REGULATED | PERCENT |
| --- | --- | --- | --- |
| BREAST | 582 | 229 | 39.35 |
| PROSTATE | 161 | 63 | 39.13 |
| PANCREATIC | 1549 | 629 | 40.61 |
| LIVER | 2892 | 1267 | 43.81 |
| RENAL | 6070 | 2579 | 42.49 |
| COLORECTAL | 603 | 262 | 43.45 |
| CERVICAL | 717 | 312 | 43.51 |
| LUNG | 662 | 298 | 45.02 |
| THYROID | 347 | 153 | 44.09 |
| OVARIAN | 504 | 202 | 40.08 |
| ENDOMETRIAL | 1631 | 652 | 39.98 |
| UROTHELIAL | 1101 | 458 | 41.60 |
| HEAD & NECK | 808 | 340 | 42.08 |
| GLIOMA | 271 | 115 | 42.44 |
| MEANOMA | 205 | 85 | 41.46 |
| STOMACH | 307 | 144 | 46.91 |
| TESTIS | 60 | 23 | 38.33 |
| ALL | 10713 | 4436 | 41.41 |
SCARS REGULATE EXPRESSION OF A PROMINENT MAJORITY OF HUMAN CANCER SURVIVAL PREDICTOR GENES
| TYPE OF CANCER | CANCER SURVIVAL GENES | SCARS-REGULATED | PERCENT |
| --- | --- | --- | --- |
| BREAST | 582 | 405 | 69.59 |
| PROSTATE | 161 | 121 | 75.16 |
| PANCREATIC | 1549 | 1112 | 71.79 |
| LIVER | 2892 | 2217 | 76.66 |
| RENAL | 6070 | 4406 | 72.59 |
| COLORECTAL | 603 | 448 | 74.30 |
| CERVICAL | 717 | 526 | 73.36 |
| LUNG | 662 | 488 | 73.72 |
| THYROID | 347 | 259 | 74.64 |
| OVARIAN | 504 | 368 | 73.02 |
| ENDOMETRIAL | 1631 | 1129 | 69.22 |
| UROTHELIAL | 1101 | 772 | 70.12 |
| HEAD & NECK | 808 | 558 | 69.06 |
| GLIOMA | 271 | 204 | 75.28 |
| MEANOMA | 205 | 148 | 72.20 |
| STOMACH | 307 | 219 | 71.34 |
| TESTIS | 60 | 41 | 68.33 |
| ALL | 10713 | 7609 | 71.03 |

## Slide 7
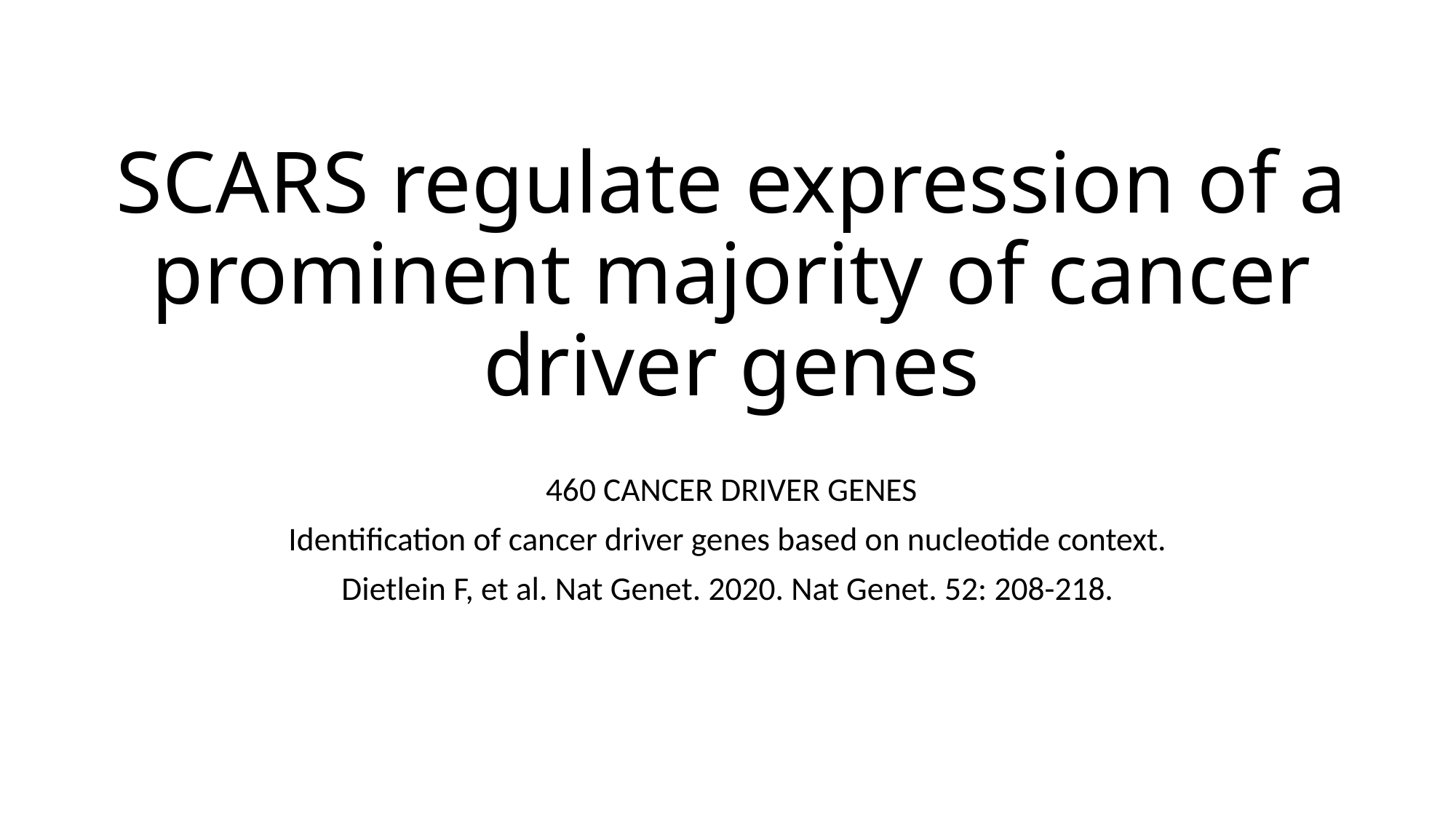

# SCARS regulate expression of a prominent majority of cancer driver genes
460 CANCER DRIVER GENES
Identification of cancer driver genes based on nucleotide context.
Dietlein F, et al. Nat Genet. 2020. Nat Genet. 52: 208-218.

## Slide 8
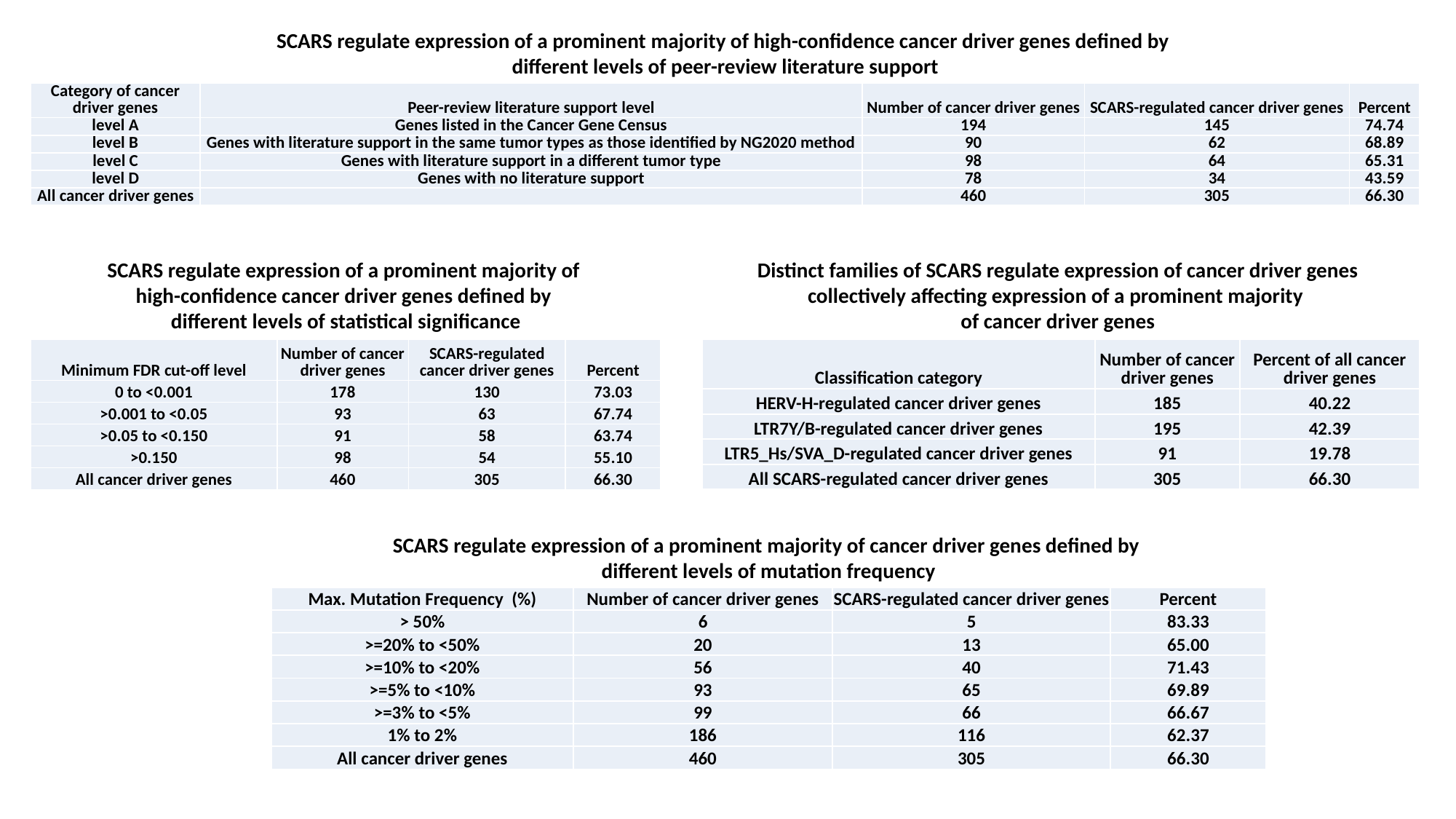

SCARS regulate expression of a prominent majority of high-confidence cancer driver genes defined by
different levels of peer-review literature support
| Category of cancer driver genes | Peer-review literature support level | Number of cancer driver genes | SCARS-regulated cancer driver genes | Percent |
| --- | --- | --- | --- | --- |
| level A | Genes listed in the Cancer Gene Census | 194 | 145 | 74.74 |
| level B | Genes with literature support in the same tumor types as those identified by NG2020 method | 90 | 62 | 68.89 |
| level C | Genes with literature support in a different tumor type | 98 | 64 | 65.31 |
| level D | Genes with no literature support | 78 | 34 | 43.59 |
| All cancer driver genes | | 460 | 305 | 66.30 |
SCARS regulate expression of a prominent majority of
high-confidence cancer driver genes defined by
different levels of statistical significance
Distinct families of SCARS regulate expression of cancer driver genes collectively affecting expression of a prominent majority
of cancer driver genes
| Minimum FDR cut-off level | Number of cancer driver genes | SCARS-regulated cancer driver genes | Percent |
| --- | --- | --- | --- |
| 0 to <0.001 | 178 | 130 | 73.03 |
| >0.001 to <0.05 | 93 | 63 | 67.74 |
| >0.05 to <0.150 | 91 | 58 | 63.74 |
| >0.150 | 98 | 54 | 55.10 |
| All cancer driver genes | 460 | 305 | 66.30 |
| Classification category | Number of cancer driver genes | Percent of all cancer driver genes |
| --- | --- | --- |
| HERV-H-regulated cancer driver genes | 185 | 40.22 |
| LTR7Y/B-regulated cancer driver genes | 195 | 42.39 |
| LTR5\_Hs/SVA\_D-regulated cancer driver genes | 91 | 19.78 |
| All SCARS-regulated cancer driver genes | 305 | 66.30 |
SCARS regulate expression of a prominent majority of cancer driver genes defined by
different levels of mutation frequency
| Max. Mutation Frequency (%) | Number of cancer driver genes | SCARS-regulated cancer driver genes | Percent |
| --- | --- | --- | --- |
| > 50% | 6 | 5 | 83.33 |
| >=20% to <50% | 20 | 13 | 65.00 |
| >=10% to <20% | 56 | 40 | 71.43 |
| >=5% to <10% | 93 | 65 | 69.89 |
| >=3% to <5% | 99 | 66 | 66.67 |
| 1% to 2% | 186 | 116 | 62.37 |
| All cancer driver genes | 460 | 305 | 66.30 |

## Slide 9
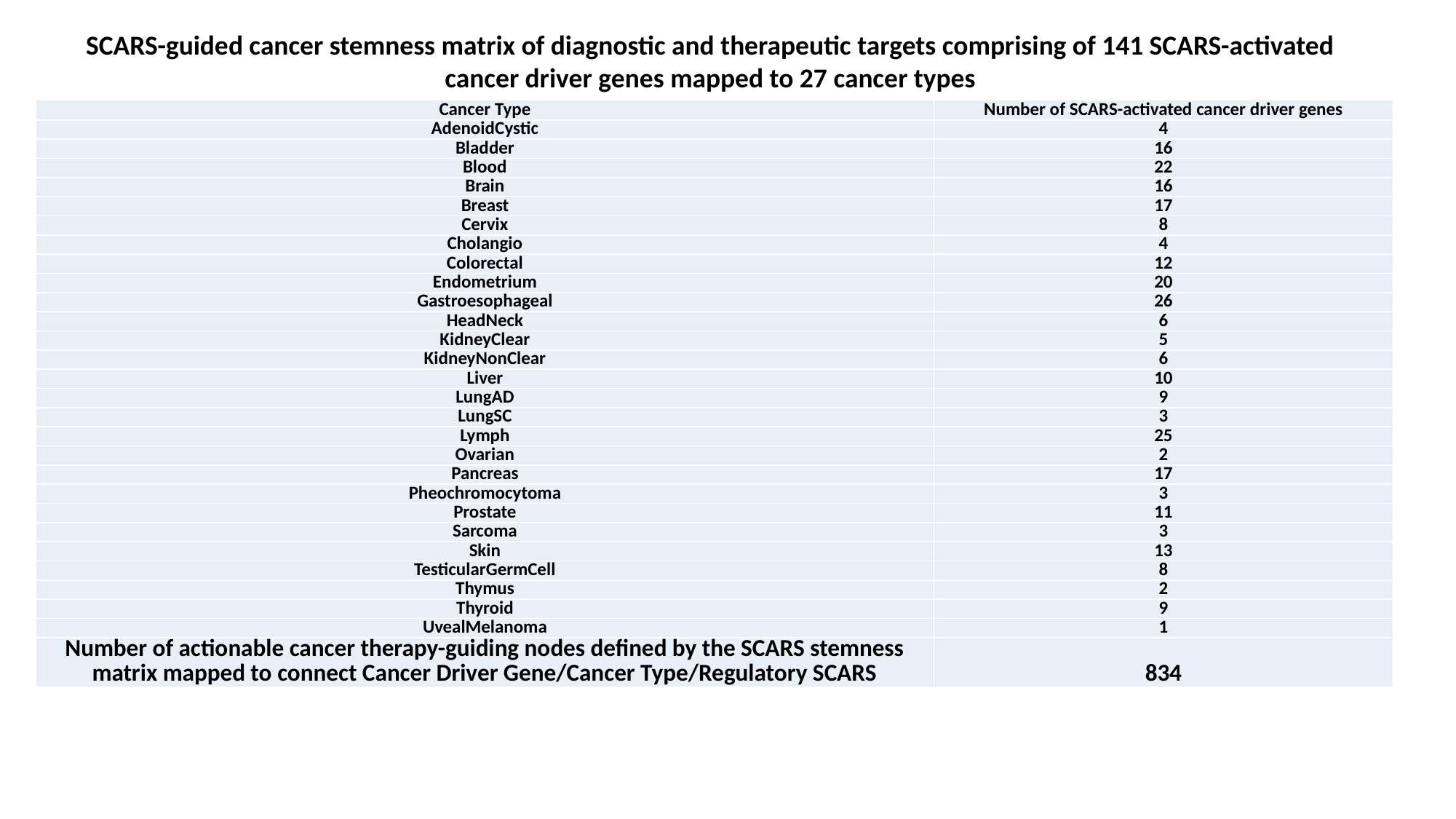

SCARS-guided cancer stemness matrix of diagnostic and therapeutic targets comprising of 141 SCARS-activated cancer driver genes mapped to 27 cancer types
| Cancer Type | Number of SCARS-activated cancer driver genes |
| --- | --- |
| AdenoidCystic | 4 |
| Bladder | 16 |
| Blood | 22 |
| Brain | 16 |
| Breast | 17 |
| Cervix | 8 |
| Cholangio | 4 |
| Colorectal | 12 |
| Endometrium | 20 |
| Gastroesophageal | 26 |
| HeadNeck | 6 |
| KidneyClear | 5 |
| KidneyNonClear | 6 |
| Liver | 10 |
| LungAD | 9 |
| LungSC | 3 |
| Lymph | 25 |
| Ovarian | 2 |
| Pancreas | 17 |
| Pheochromocytoma | 3 |
| Prostate | 11 |
| Sarcoma | 3 |
| Skin | 13 |
| TesticularGermCell | 8 |
| Thymus | 2 |
| Thyroid | 9 |
| UvealMelanoma | 1 |
| Number of actionable cancer therapy-guiding nodes defined by the SCARS stemness matrix mapped to connect Cancer Driver Gene/Cancer Type/Regulatory SCARS | 834 |

## Slide 10
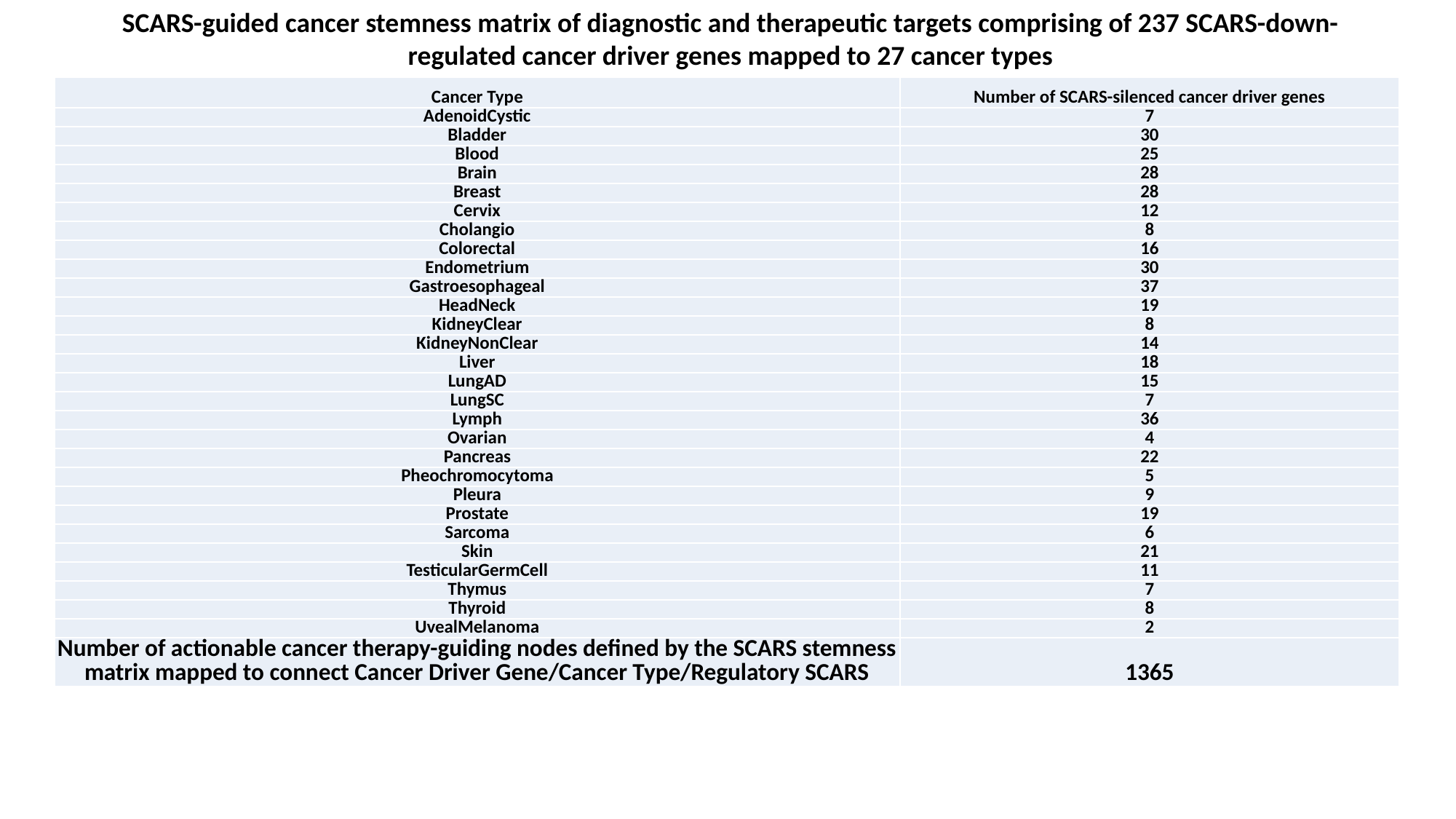

SCARS-guided cancer stemness matrix of diagnostic and therapeutic targets comprising of 237 SCARS-down-regulated cancer driver genes mapped to 27 cancer types
| Cancer Type | Number of SCARS-silenced cancer driver genes |
| --- | --- |
| AdenoidCystic | 7 |
| Bladder | 30 |
| Blood | 25 |
| Brain | 28 |
| Breast | 28 |
| Cervix | 12 |
| Cholangio | 8 |
| Colorectal | 16 |
| Endometrium | 30 |
| Gastroesophageal | 37 |
| HeadNeck | 19 |
| KidneyClear | 8 |
| KidneyNonClear | 14 |
| Liver | 18 |
| LungAD | 15 |
| LungSC | 7 |
| Lymph | 36 |
| Ovarian | 4 |
| Pancreas | 22 |
| Pheochromocytoma | 5 |
| Pleura | 9 |
| Prostate | 19 |
| Sarcoma | 6 |
| Skin | 21 |
| TesticularGermCell | 11 |
| Thymus | 7 |
| Thyroid | 8 |
| UvealMelanoma | 2 |
| Number of actionable cancer therapy-guiding nodes defined by the SCARS stemness matrix mapped to connect Cancer Driver Gene/Cancer Type/Regulatory SCARS | 1365 |

## Slide 11
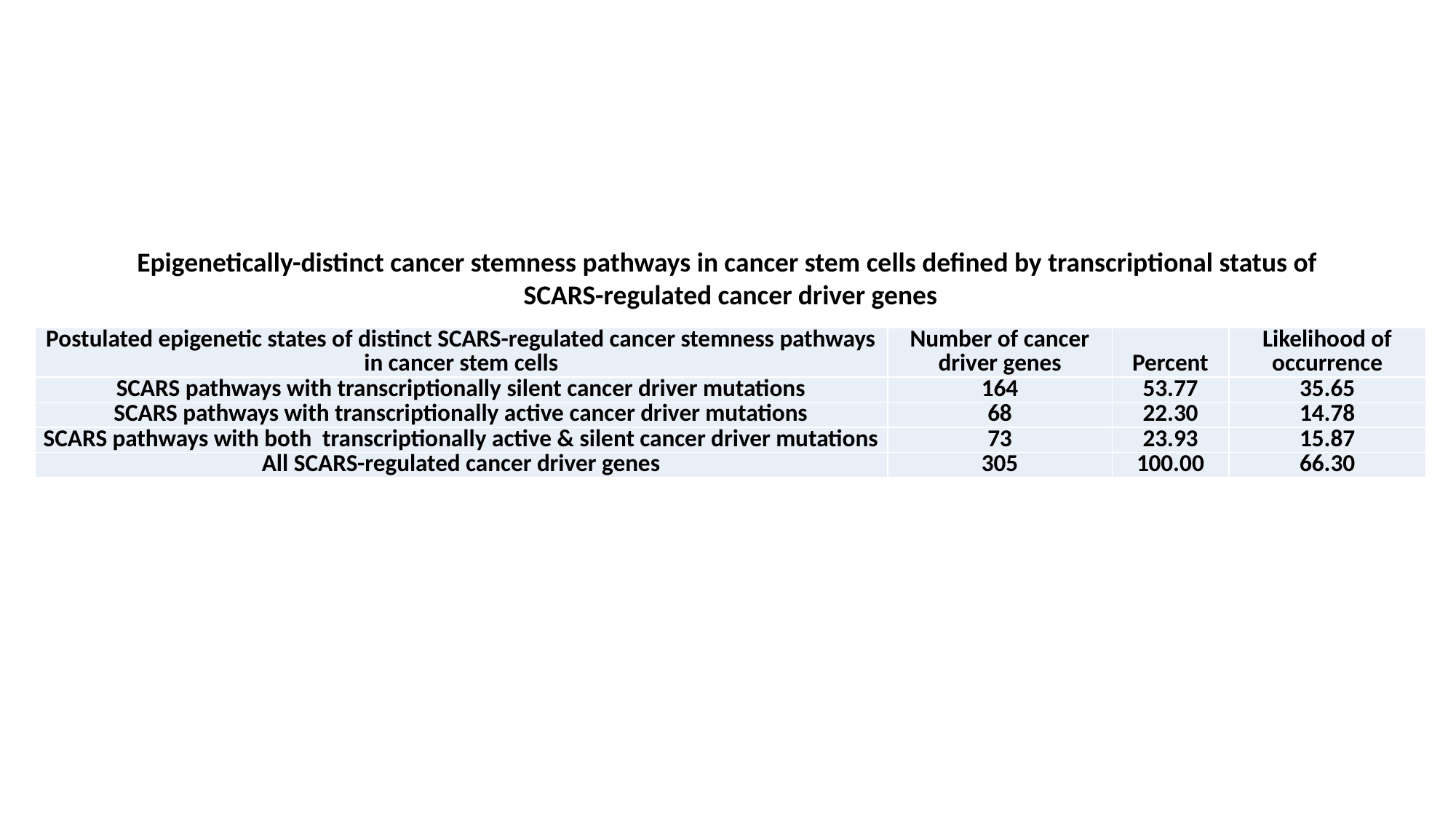

Epigenetically-distinct cancer stemness pathways in cancer stem cells defined by transcriptional status of
SCARS-regulated cancer driver genes
| Postulated epigenetic states of distinct SCARS-regulated cancer stemness pathways in cancer stem cells | Number of cancer driver genes | Percent | Likelihood of occurrence |
| --- | --- | --- | --- |
| SCARS pathways with transcriptionally silent cancer driver mutations | 164 | 53.77 | 35.65 |
| SCARS pathways with transcriptionally active cancer driver mutations | 68 | 22.30 | 14.78 |
| SCARS pathways with both transcriptionally active & silent cancer driver mutations | 73 | 23.93 | 15.87 |
| All SCARS-regulated cancer driver genes | 305 | 100.00 | 66.30 |

## Slide 12
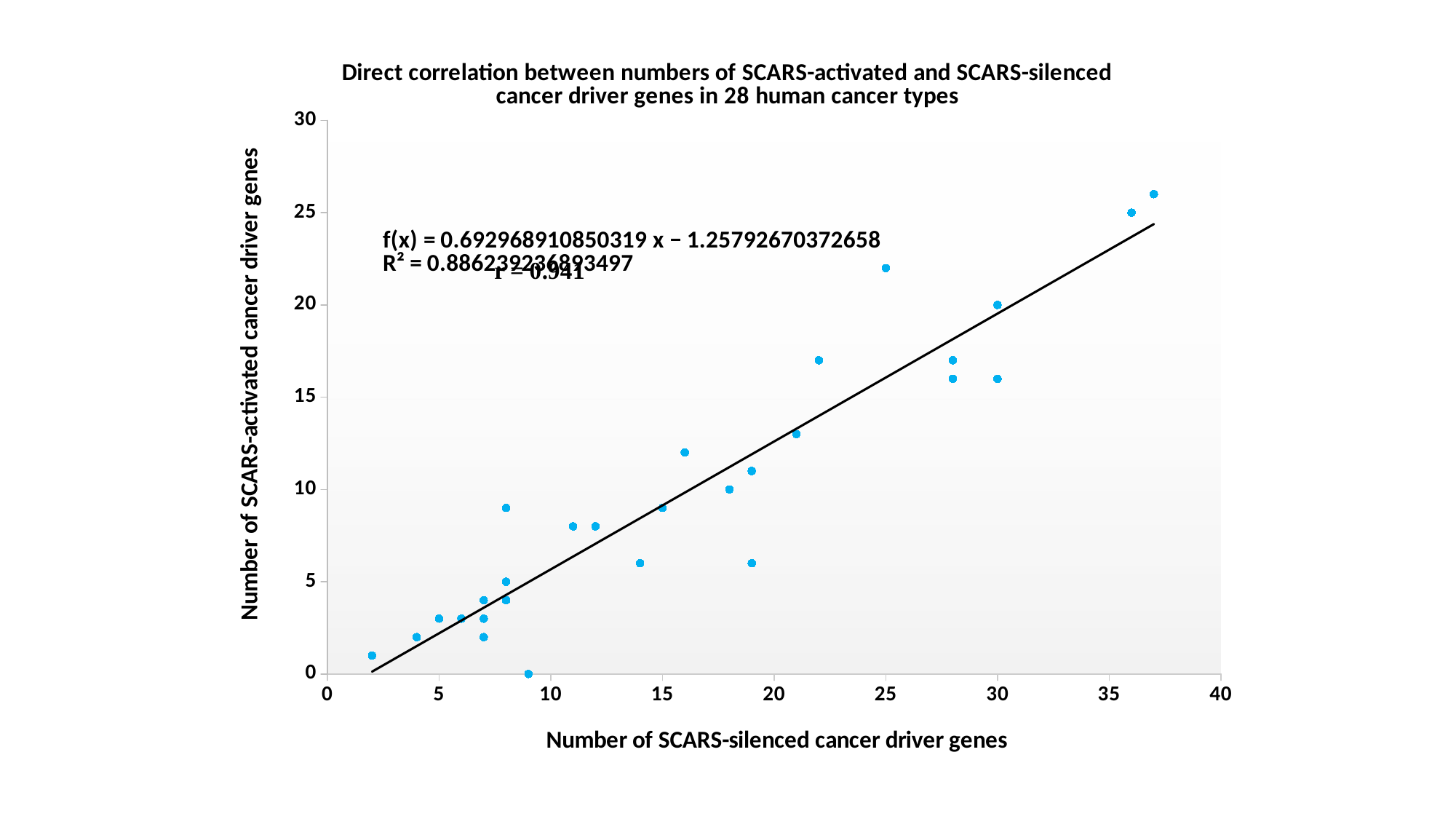

### Chart: Direct correlation between numbers of SCARS-activated and SCARS-silenced cancer driver genes in 28 human cancer types
| Category | Number of SCARS-activated cancer driver genes |
|---|---|
