# Supplementary material for: Genomics-Guided Drawing of Molecular and Pathophysiological Components of Malignant Regulatory Signatures Reveals a Pivotal Role in Human Diseases of Stem Cell-Associated Retroviral Sequences and Functionally-Active hESC Enhancers
Source: Front Oncol. 2021 Mar 31;11:638363. doi: 10.3389/fonc.2021.638363 (PMC8044830; doi:10.3389/fonc.2021.638363)
Supplement: Supplementary file 1 [file Presentation_1.zip › Supplemental Note S4A. SCARS Networks CaMarkers Genes.pptx]

## Slide 1
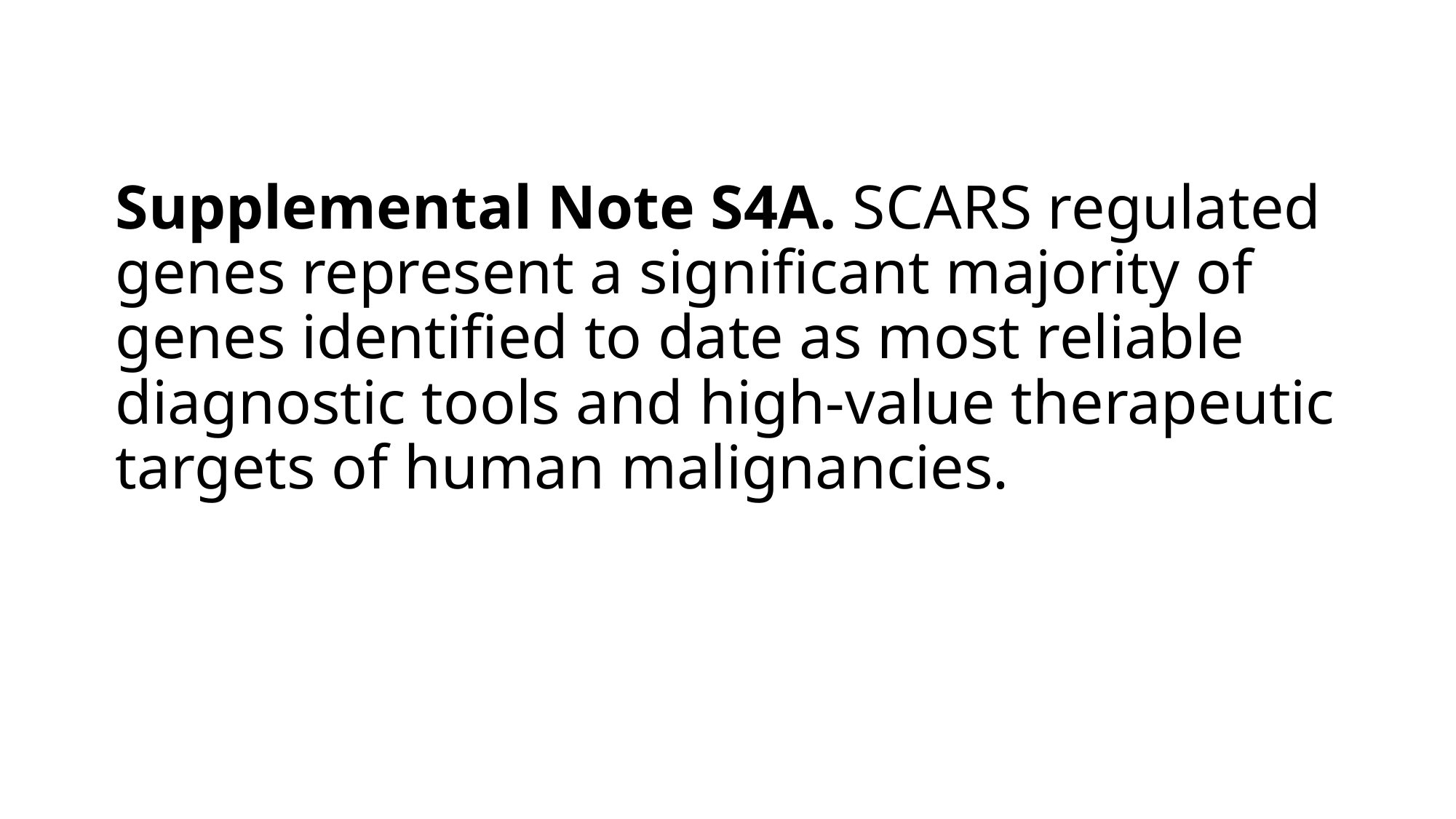

# Supplemental Note S4A. SCARS regulated genes represent a significant majority of genes identified to date as most reliable diagnostic tools and high-value therapeutic targets of human malignancies.

## Slide 2
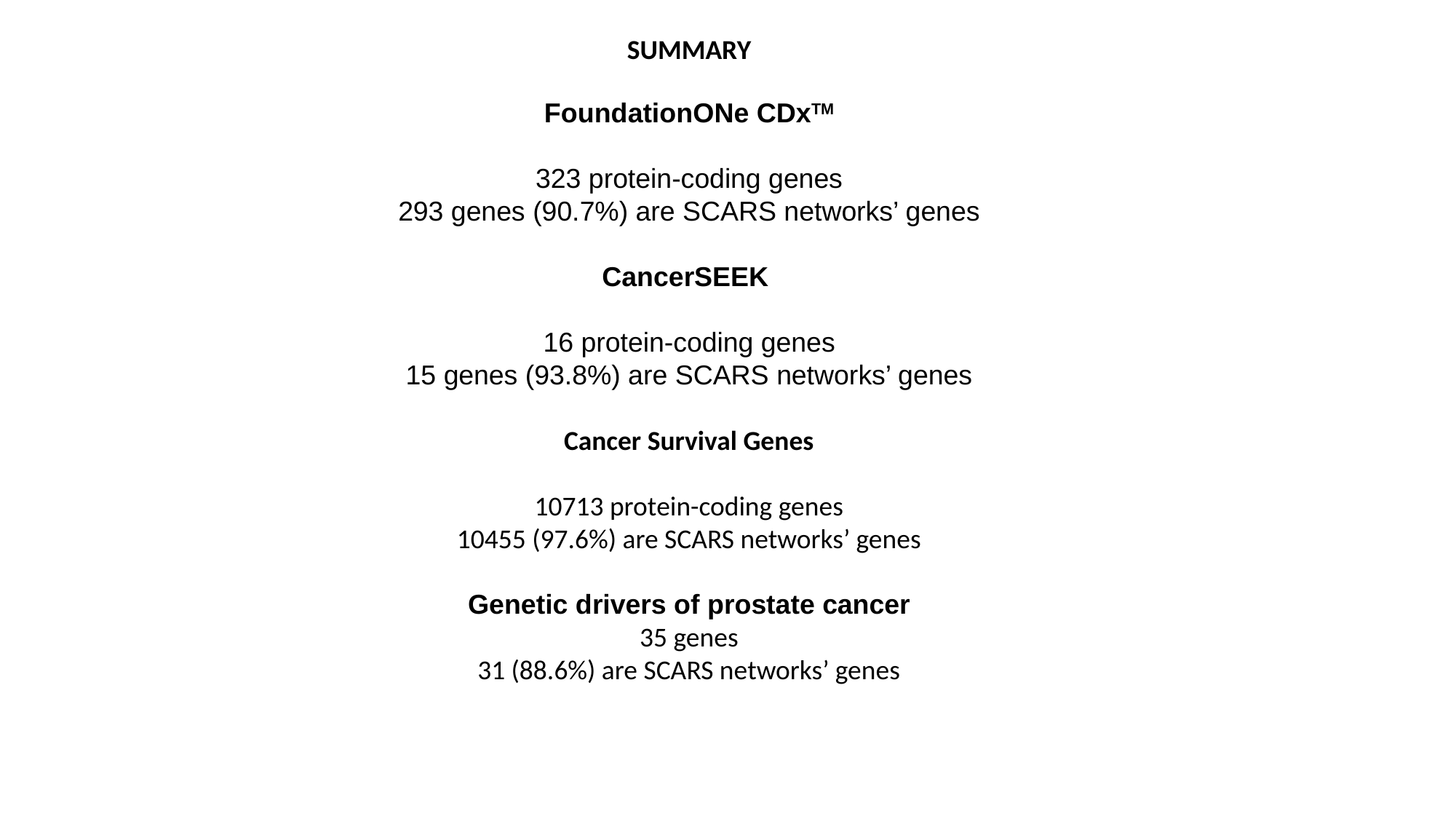

SUMMARY
FoundationONe CDxTM
323 protein-coding genes
293 genes (90.7%) are SCARS networks’ genes
CancerSEEK
16 protein-coding genes
15 genes (93.8%) are SCARS networks’ genes
Cancer Survival Genes
10713 protein-coding genes
10455 (97.6%) are SCARS networks’ genes
Genetic drivers of prostate cancer
35 genes
31 (88.6%) are SCARS networks’ genes

## Slide 3
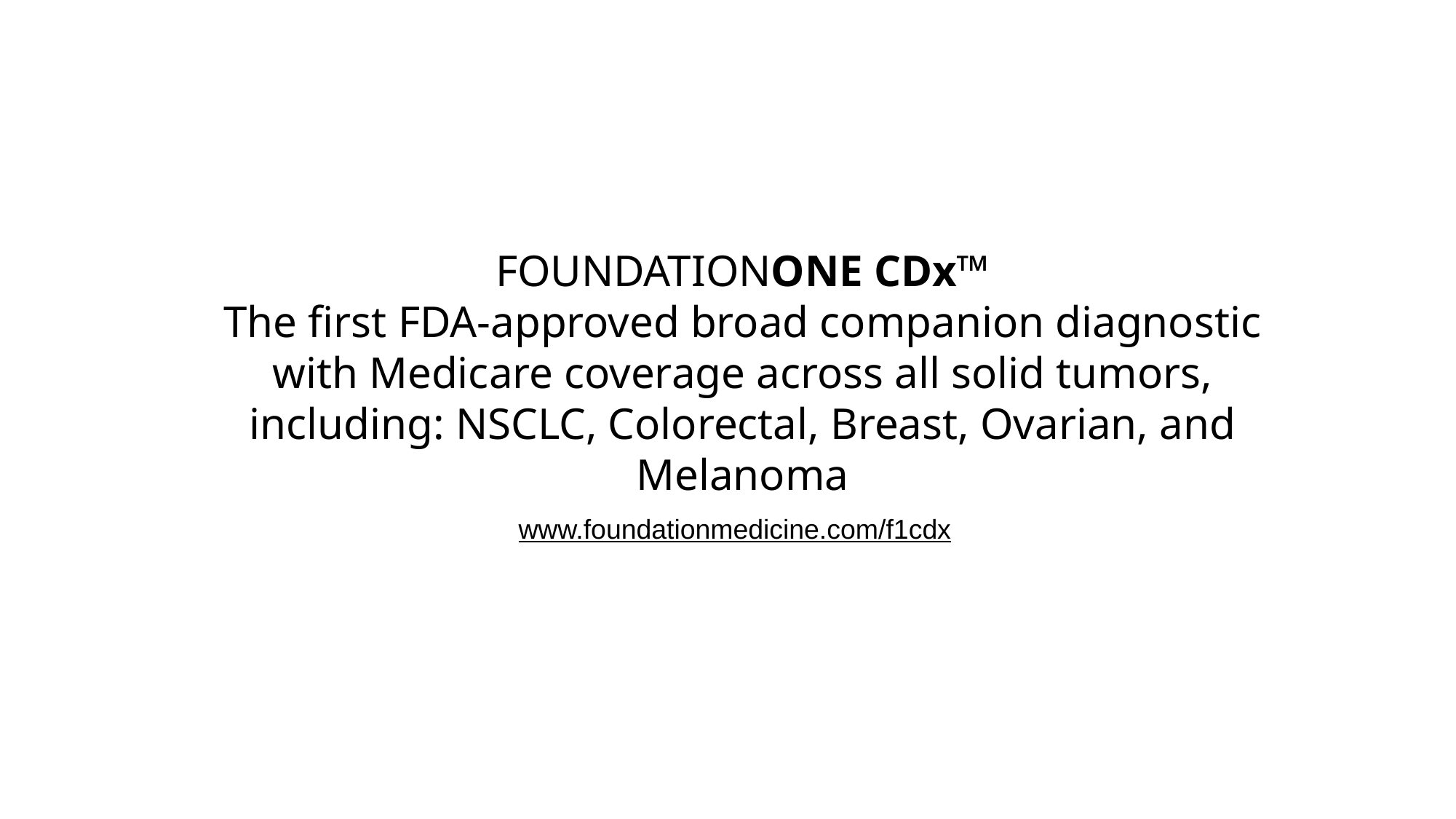

FOUNDATIONONE CDx™
The first FDA-approved broad companion diagnostic with Medicare coverage across all solid tumors, including: NSCLC, Colorectal, Breast, Ovarian, and Melanoma
www.foundationmedicine.com/f1cdx

## Slide 4
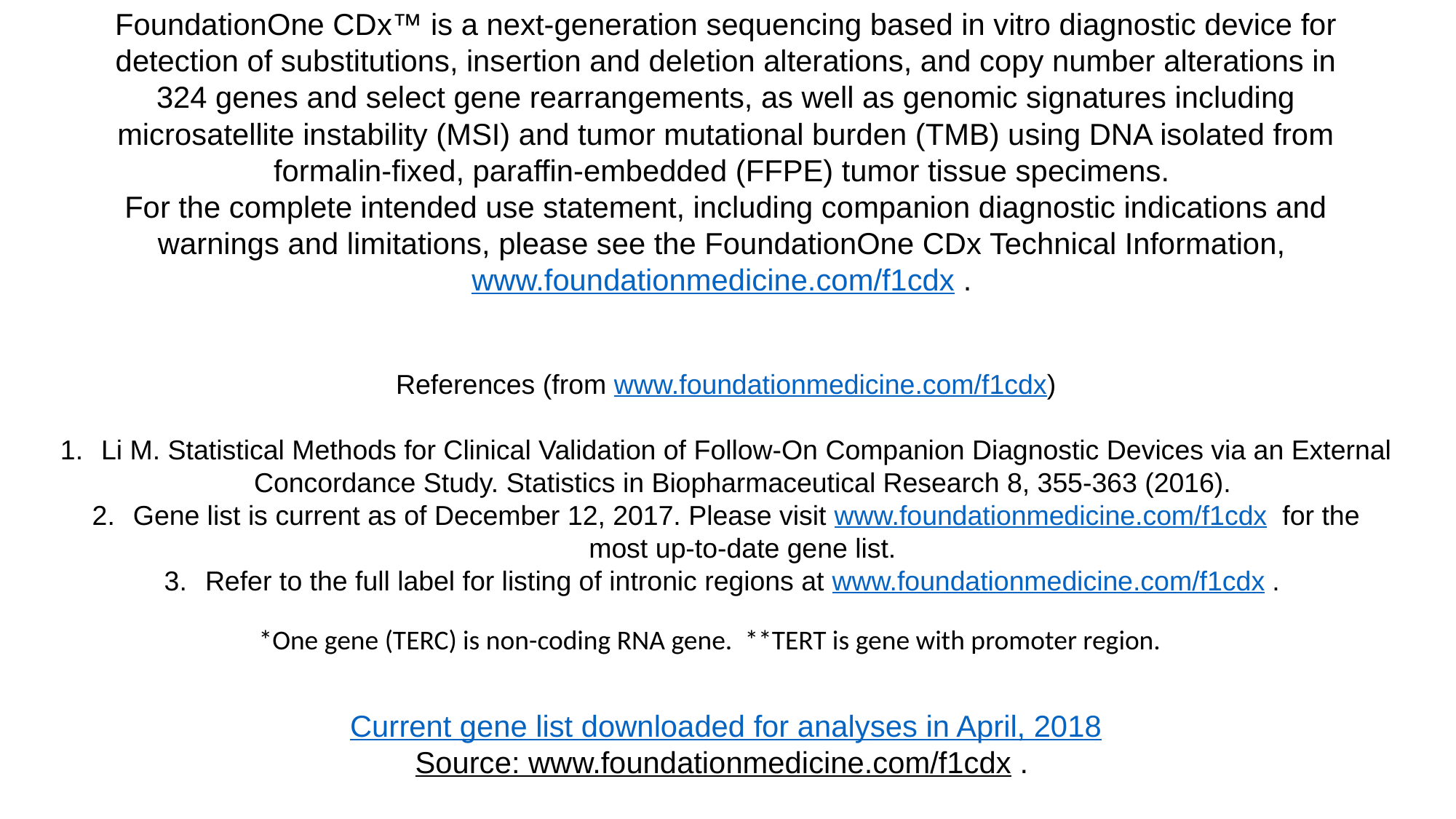

FoundationOne CDx™ is a next-generation sequencing based in vitro diagnostic device for detection of substitutions, insertion and deletion alterations, and copy number alterations in 324 genes and select gene rearrangements, as well as genomic signatures including microsatellite instability (MSI) and tumor mutational burden (TMB) using DNA isolated from formalin-fixed, paraffin-embedded (FFPE) tumor tissue specimens.
For the complete intended use statement, including companion diagnostic indications and warnings and limitations, please see the FoundationOne CDx Technical Information, www.foundationmedicine.com/f1cdx .
References (from www.foundationmedicine.com/f1cdx)
Li M. Statistical Methods for Clinical Validation of Follow-On Companion Diagnostic Devices via an External Concordance Study. Statistics in Biopharmaceutical Research 8, 355-363 (2016).
Gene list is current as of December 12, 2017. Please visit www.foundationmedicine.com/f1cdx for the most up-to-date gene list.
Refer to the full label for listing of intronic regions at www.foundationmedicine.com/f1cdx .
*One gene (TERC) is non-coding RNA gene. **TERT is gene with promoter region.
Current gene list downloaded for analyses in April, 2018
Source: www.foundationmedicine.com/f1cdx .

## Slide 5
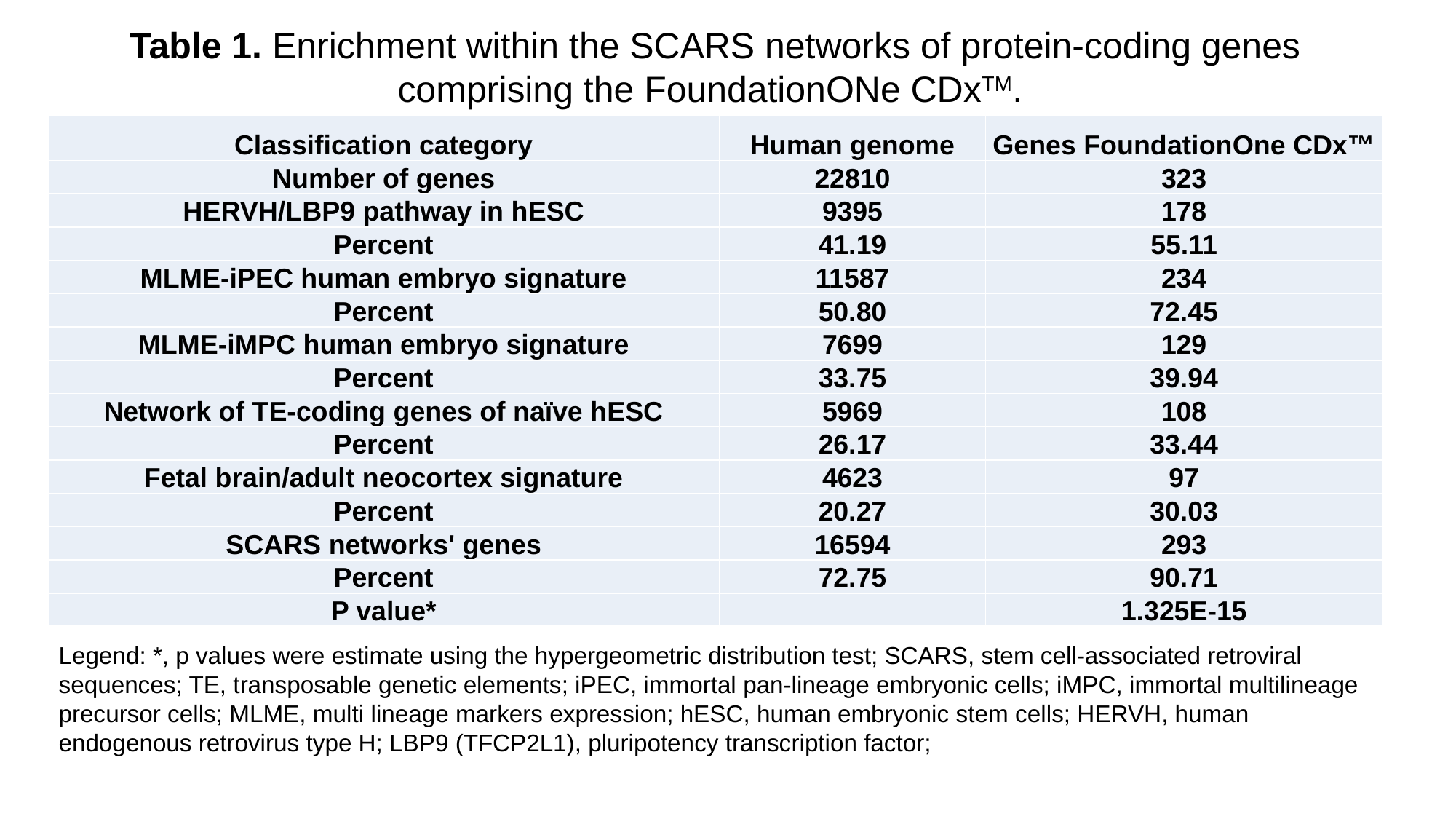

Table 1. Enrichment within the SCARS networks of protein-coding genes comprising the FoundationONe CDxTM.
| Classification category | Human genome | Genes FoundationOne CDx™ |
| --- | --- | --- |
| Number of genes | 22810 | 323 |
| HERVH/LBP9 pathway in hESC | 9395 | 178 |
| Percent | 41.19 | 55.11 |
| MLME-iPEC human embryo signature | 11587 | 234 |
| Percent | 50.80 | 72.45 |
| MLME-iMPC human embryo signature | 7699 | 129 |
| Percent | 33.75 | 39.94 |
| Network of TE-coding genes of naïve hESC | 5969 | 108 |
| Percent | 26.17 | 33.44 |
| Fetal brain/adult neocortex signature | 4623 | 97 |
| Percent | 20.27 | 30.03 |
| SCARS networks' genes | 16594 | 293 |
| Percent | 72.75 | 90.71 |
| P value\* | | 1.325E-15 |
Legend: *, p values were estimate using the hypergeometric distribution test; SCARS, stem cell-associated retroviral sequences; TE, transposable genetic elements; iPEC, immortal pan-lineage embryonic cells; iMPC, immortal multilineage precursor cells; MLME, multi lineage markers expression; hESC, human embryonic stem cells; HERVH, human endogenous retrovirus type H; LBP9 (TFCP2L1), pluripotency transcription factor;

## Slide 6
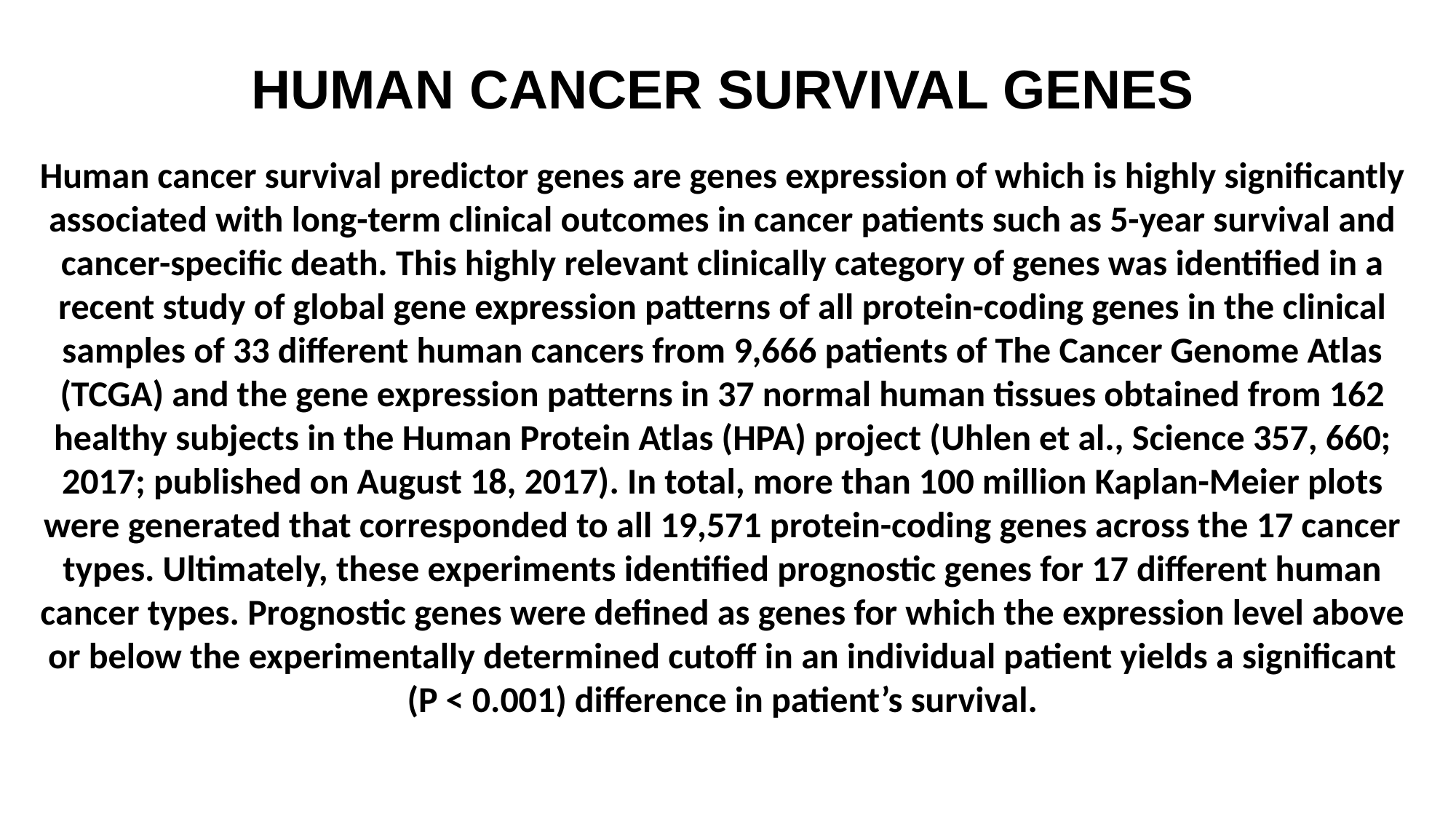

# HUMAN CANCER SURVIVAL GENES
Human cancer survival predictor genes are genes expression of which is highly significantly associated with long-term clinical outcomes in cancer patients such as 5-year survival and cancer-specific death. This highly relevant clinically category of genes was identified in a recent study of global gene expression patterns of all protein-coding genes in the clinical samples of 33 different human cancers from 9,666 patients of The Cancer Genome Atlas (TCGA) and the gene expression patterns in 37 normal human tissues obtained from 162 healthy subjects in the Human Protein Atlas (HPA) project (Uhlen et al., Science 357, 660; 2017; published on August 18, 2017). In total, more than 100 million Kaplan-Meier plots were generated that corresponded to all 19,571 protein-coding genes across the 17 cancer types. Ultimately, these experiments identified prognostic genes for 17 different human cancer types. Prognostic genes were defined as genes for which the expression level above or below the experimentally determined cutoff in an individual patient yields a significant (P < 0.001) difference in patient’s survival.

## Slide 7
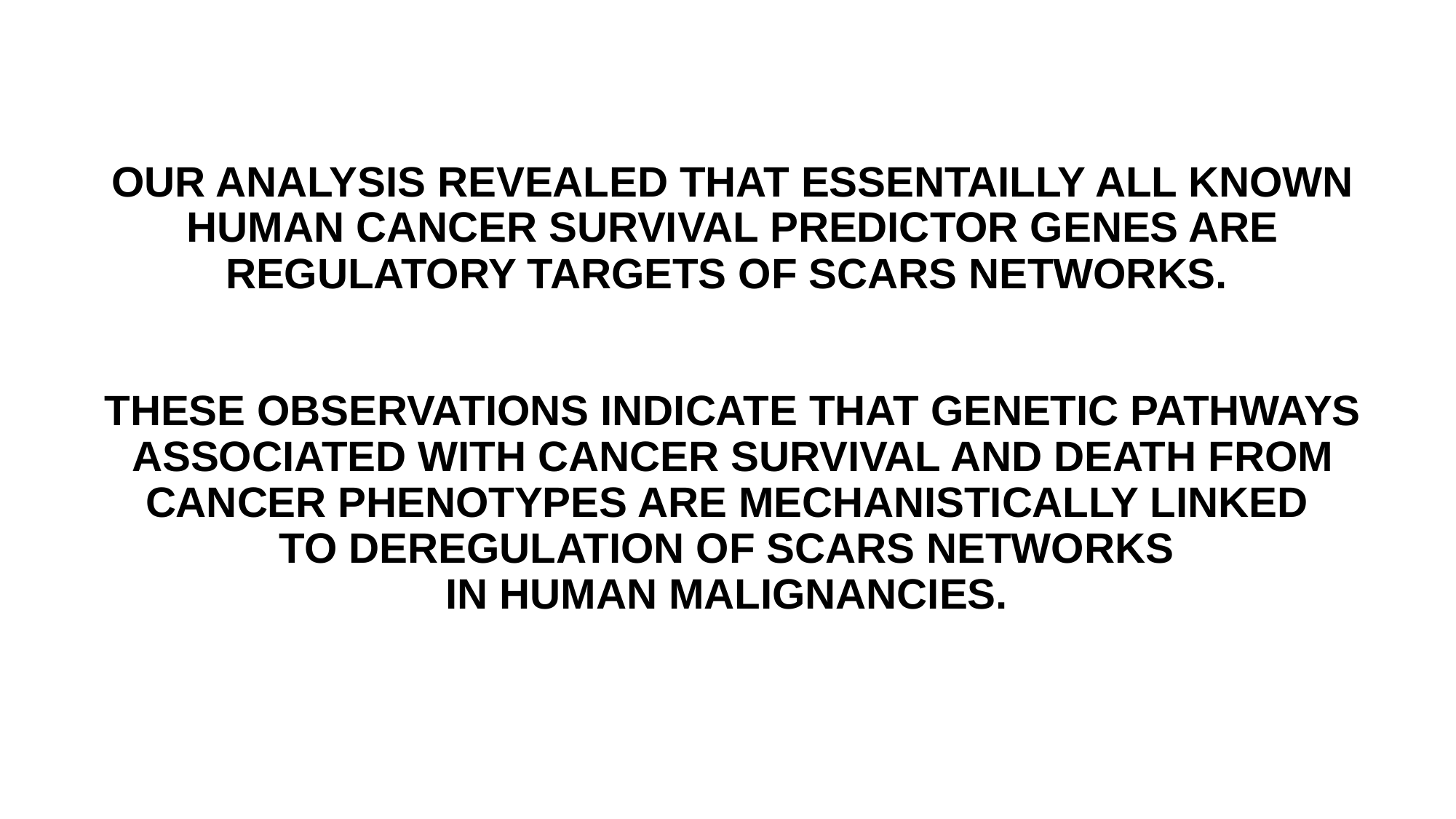

# OUR ANALYSIS REVEALED THAT ESSENTAILLY ALL KNOWN HUMAN CANCER SURVIVAL PREDICTOR GENES ARE REGULATORY TARGETS OF SCARS NETWORKS. THESE OBSERVATIONS INDICATE THAT GENETIC PATHWAYS ASSOCIATED WITH CANCER SURVIVAL AND DEATH FROM CANCER PHENOTYPES ARE MECHANISTICALLY LINKED TO DEREGULATION OF SCARS NETWORKS IN HUMAN MALIGNANCIES.

## Slide 8
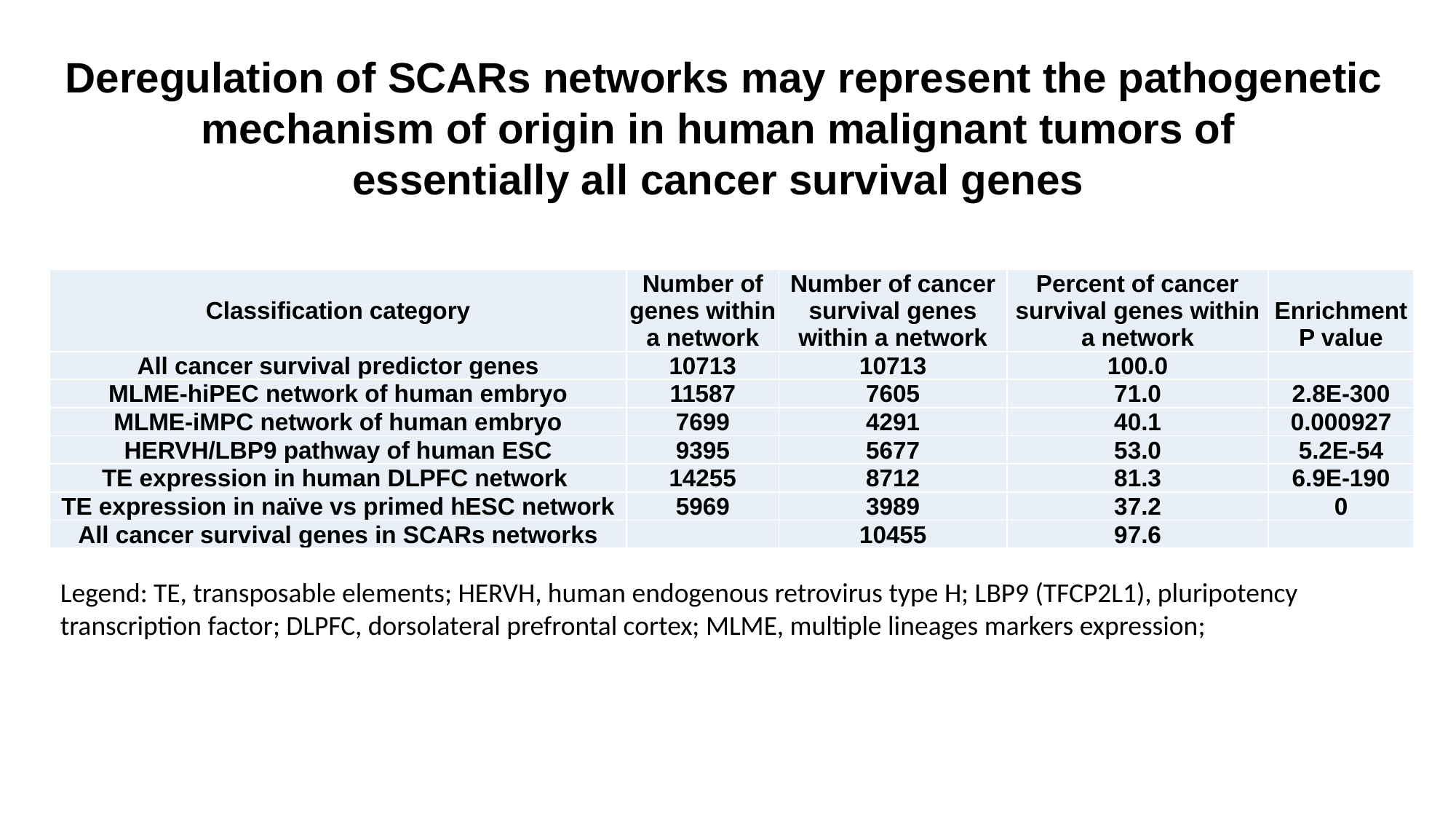

Deregulation of SCARs networks may represent the pathogenetic mechanism of origin in human malignant tumors of
essentially all cancer survival genes
| Classification category | Number of genes within a network | Number of cancer survival genes within a network | Percent of cancer survival genes within a network | Enrichment P value |
| --- | --- | --- | --- | --- |
| All cancer survival predictor genes | 10713 | 10713 | 100.0 | |
| MLME-hiPEC network of human embryo | 11587 | 7605 | 71.0 | 2.8E-300 |
| MLME-iMPC network of human embryo | 7699 | 4291 | 40.1 | 0.000927 |
| HERVH/LBP9 pathway of human ESC | 9395 | 5677 | 53.0 | 5.2E-54 |
| TE expression in human DLPFC network | 14255 | 8712 | 81.3 | 6.9E-190 |
| TE expression in naïve vs primed hESC network | 5969 | 3989 | 37.2 | 0 |
| All cancer survival genes in SCARs networks | | 10455 | 97.6 | |
Legend: TE, transposable elements; HERVH, human endogenous retrovirus type H; LBP9 (TFCP2L1), pluripotency transcription factor; DLPFC, dorsolateral prefrontal cortex; MLME, multiple lineages markers expression;

## Slide 9
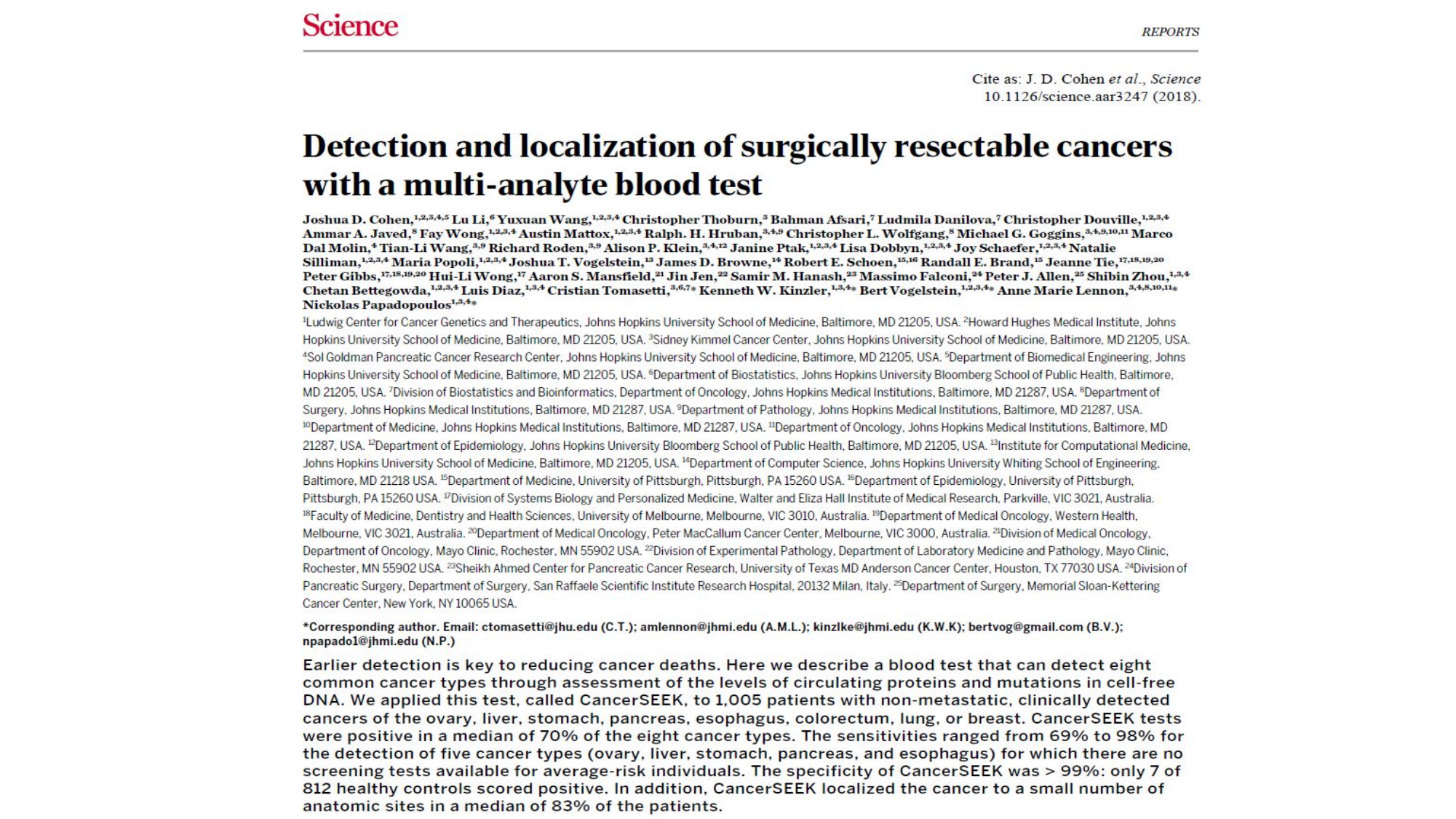

## Slide 10
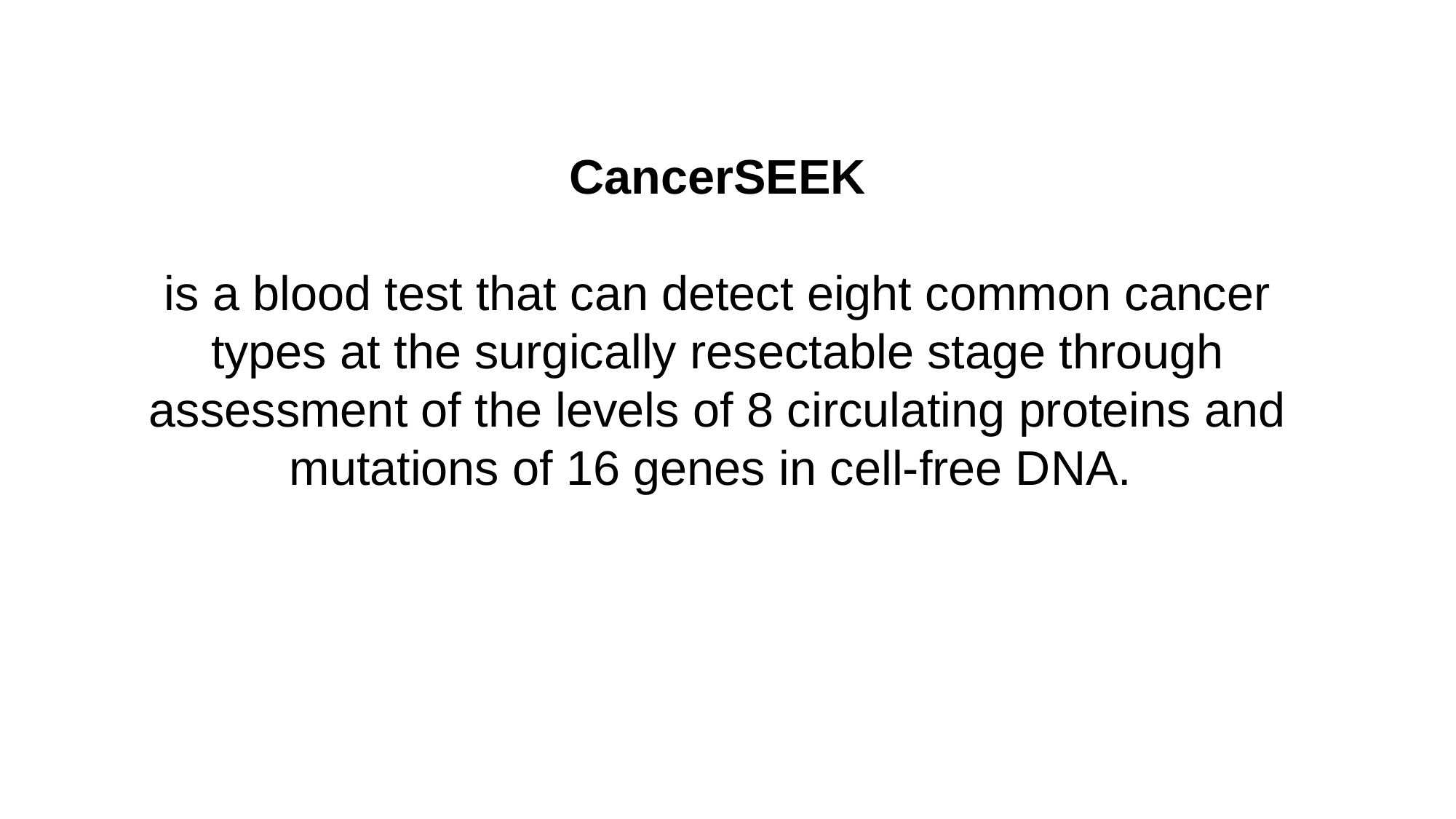

CancerSEEK
is a blood test that can detect eight common cancer types at the surgically resectable stage through assessment of the levels of 8 circulating proteins and mutations of 16 genes in cell-free DNA.

## Slide 11
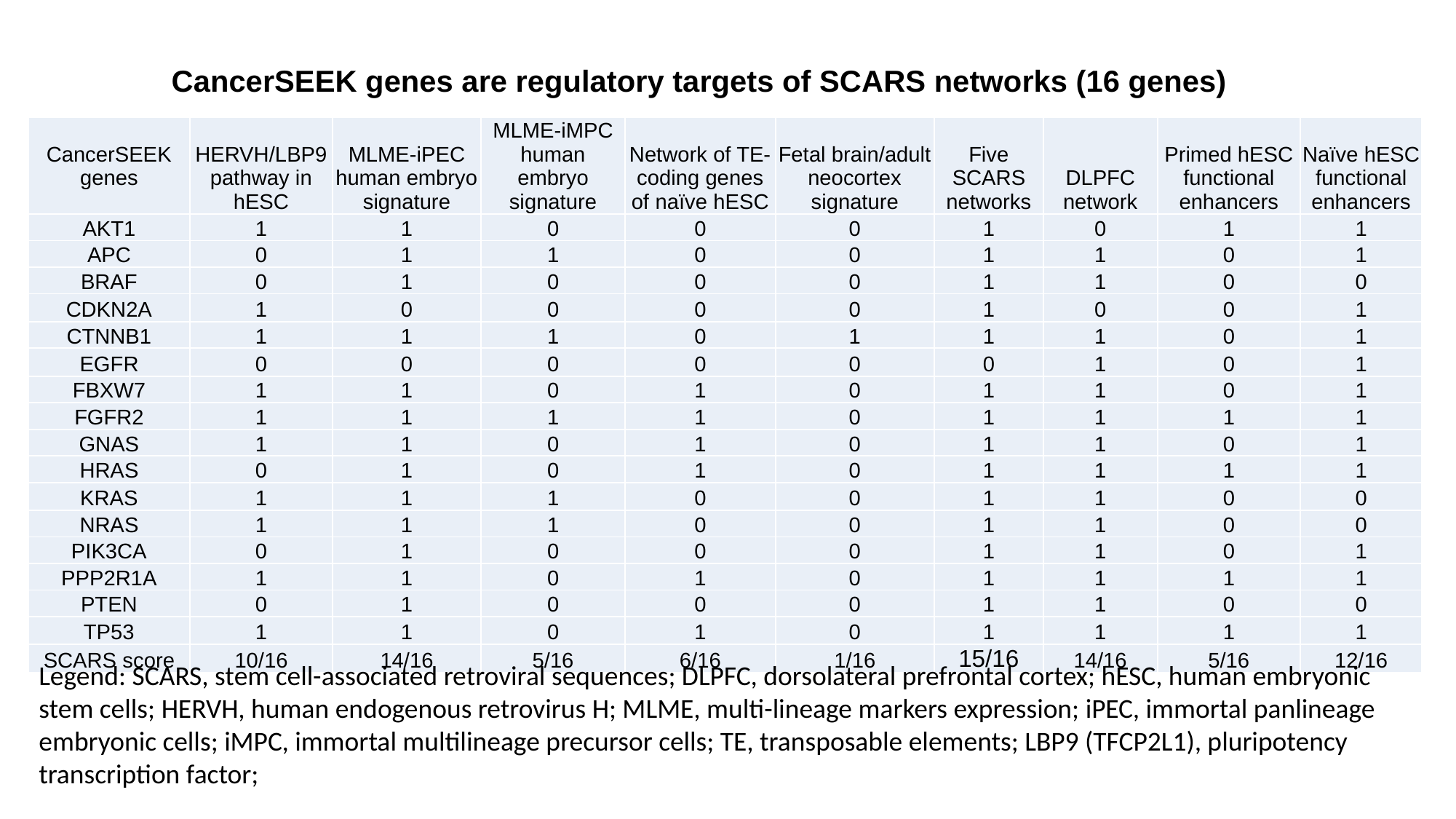

CancerSEEK genes are regulatory targets of SCARS networks (16 genes)
| CancerSEEK genes | HERVH/LBP9 pathway in hESC | MLME-iPEC human embryo signature | MLME-iMPC human embryo signature | Network of TE-coding genes of naïve hESC | Fetal brain/adult neocortex signature | Five SCARS networks | DLPFC network | Primed hESC functional enhancers | Naïve hESC functional enhancers |
| --- | --- | --- | --- | --- | --- | --- | --- | --- | --- |
| AKT1 | 1 | 1 | 0 | 0 | 0 | 1 | 0 | 1 | 1 |
| APC | 0 | 1 | 1 | 0 | 0 | 1 | 1 | 0 | 1 |
| BRAF | 0 | 1 | 0 | 0 | 0 | 1 | 1 | 0 | 0 |
| CDKN2A | 1 | 0 | 0 | 0 | 0 | 1 | 0 | 0 | 1 |
| CTNNB1 | 1 | 1 | 1 | 0 | 1 | 1 | 1 | 0 | 1 |
| EGFR | 0 | 0 | 0 | 0 | 0 | 0 | 1 | 0 | 1 |
| FBXW7 | 1 | 1 | 0 | 1 | 0 | 1 | 1 | 0 | 1 |
| FGFR2 | 1 | 1 | 1 | 1 | 0 | 1 | 1 | 1 | 1 |
| GNAS | 1 | 1 | 0 | 1 | 0 | 1 | 1 | 0 | 1 |
| HRAS | 0 | 1 | 0 | 1 | 0 | 1 | 1 | 1 | 1 |
| KRAS | 1 | 1 | 1 | 0 | 0 | 1 | 1 | 0 | 0 |
| NRAS | 1 | 1 | 1 | 0 | 0 | 1 | 1 | 0 | 0 |
| PIK3CA | 0 | 1 | 0 | 0 | 0 | 1 | 1 | 0 | 1 |
| PPP2R1A | 1 | 1 | 0 | 1 | 0 | 1 | 1 | 1 | 1 |
| PTEN | 0 | 1 | 0 | 0 | 0 | 1 | 1 | 0 | 0 |
| TP53 | 1 | 1 | 0 | 1 | 0 | 1 | 1 | 1 | 1 |
| SCARS score | 10/16 | 14/16 | 5/16 | 6/16 | 1/16 | 15/16 | 14/16 | 5/16 | 12/16 |
Legend: SCARS, stem cell-associated retroviral sequences; DLPFC, dorsolateral prefrontal cortex; hESC, human embryonic stem cells; HERVH, human endogenous retrovirus H; MLME, multi-lineage markers expression; iPEC, immortal panlineage embryonic cells; iMPC, immortal multilineage precursor cells; TE, transposable elements; LBP9 (TFCP2L1), pluripotency transcription factor;

## Slide 12
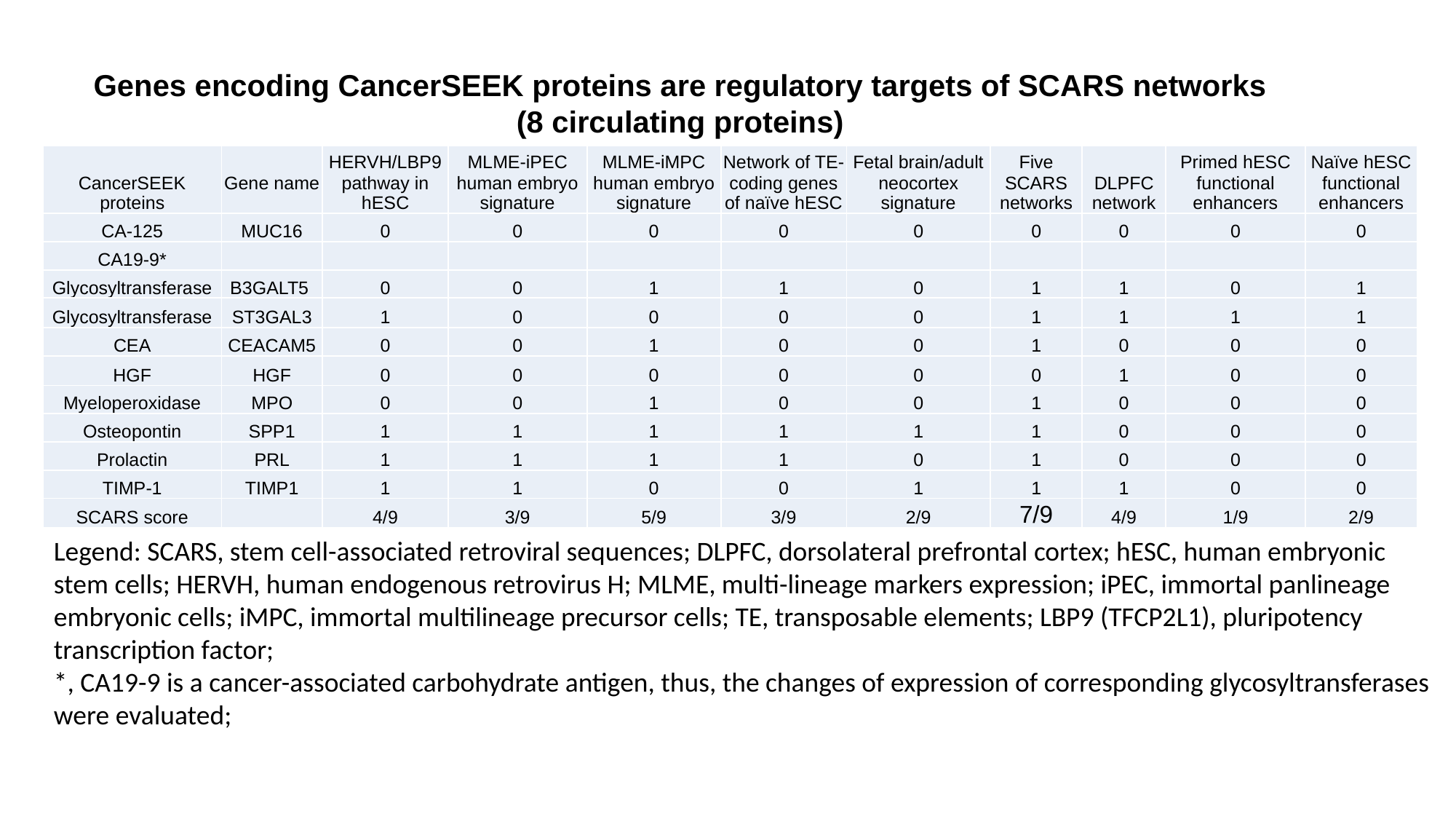

Genes encoding CancerSEEK proteins are regulatory targets of SCARS networks
(8 circulating proteins)
| CancerSEEK proteins | Gene name | HERVH/LBP9 pathway in hESC | MLME-iPEC human embryo signature | MLME-iMPC human embryo signature | Network of TE-coding genes of naïve hESC | Fetal brain/adult neocortex signature | Five SCARS networks | DLPFC network | Primed hESC functional enhancers | Naïve hESC functional enhancers |
| --- | --- | --- | --- | --- | --- | --- | --- | --- | --- | --- |
| CA-125 | MUC16 | 0 | 0 | 0 | 0 | 0 | 0 | 0 | 0 | 0 |
| CA19-9\* | | | | | | | | | | |
| Glycosyltransferase | B3GALT5 | 0 | 0 | 1 | 1 | 0 | 1 | 1 | 0 | 1 |
| Glycosyltransferase | ST3GAL3 | 1 | 0 | 0 | 0 | 0 | 1 | 1 | 1 | 1 |
| CEA | CEACAM5 | 0 | 0 | 1 | 0 | 0 | 1 | 0 | 0 | 0 |
| HGF | HGF | 0 | 0 | 0 | 0 | 0 | 0 | 1 | 0 | 0 |
| Myeloperoxidase | MPO | 0 | 0 | 1 | 0 | 0 | 1 | 0 | 0 | 0 |
| Osteopontin | SPP1 | 1 | 1 | 1 | 1 | 1 | 1 | 0 | 0 | 0 |
| Prolactin | PRL | 1 | 1 | 1 | 1 | 0 | 1 | 0 | 0 | 0 |
| TIMP-1 | TIMP1 | 1 | 1 | 0 | 0 | 1 | 1 | 1 | 0 | 0 |
| SCARS score | | 4/9 | 3/9 | 5/9 | 3/9 | 2/9 | 7/9 | 4/9 | 1/9 | 2/9 |
Legend: SCARS, stem cell-associated retroviral sequences; DLPFC, dorsolateral prefrontal cortex; hESC, human embryonic stem cells; HERVH, human endogenous retrovirus H; MLME, multi-lineage markers expression; iPEC, immortal panlineage embryonic cells; iMPC, immortal multilineage precursor cells; TE, transposable elements; LBP9 (TFCP2L1), pluripotency transcription factor;
*, CA19-9 is a cancer-associated carbohydrate antigen, thus, the changes of expression of corresponding glycosyltransferases were evaluated;

## Slide 13
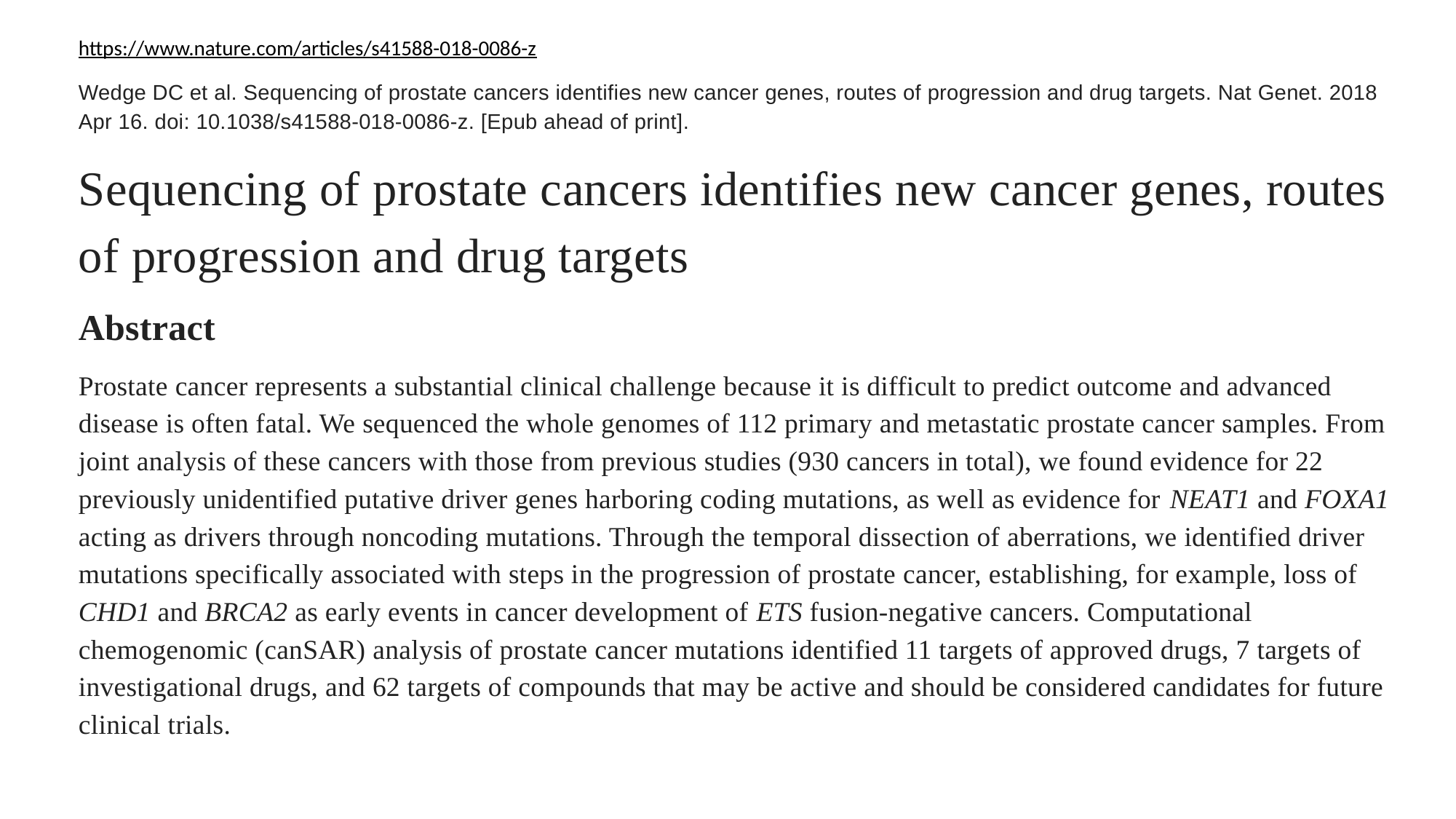

https://www.nature.com/articles/s41588-018-0086-z
Wedge DC et al. Sequencing of prostate cancers identifies new cancer genes, routes of progression and drug targets. Nat Genet. 2018 Apr 16. doi: 10.1038/s41588-018-0086-z. [Epub ahead of print].
Sequencing of prostate cancers identifies new cancer genes, routes of progression and drug targets
Abstract
Prostate cancer represents a substantial clinical challenge because it is difficult to predict outcome and advanced disease is often fatal. We sequenced the whole genomes of 112 primary and metastatic prostate cancer samples. From joint analysis of these cancers with those from previous studies (930 cancers in total), we found evidence for 22 previously unidentified putative driver genes harboring coding mutations, as well as evidence for NEAT1 and FOXA1 acting as drivers through noncoding mutations. Through the temporal dissection of aberrations, we identified driver mutations specifically associated with steps in the progression of prostate cancer, establishing, for example, loss of CHD1 and BRCA2 as early events in cancer development of ETS fusion-negative cancers. Computational chemogenomic (canSAR) analysis of prostate cancer mutations identified 11 targets of approved drugs, 7 targets of investigational drugs, and 62 targets of compounds that may be active and should be considered candidates for future clinical trials.

## Slide 14
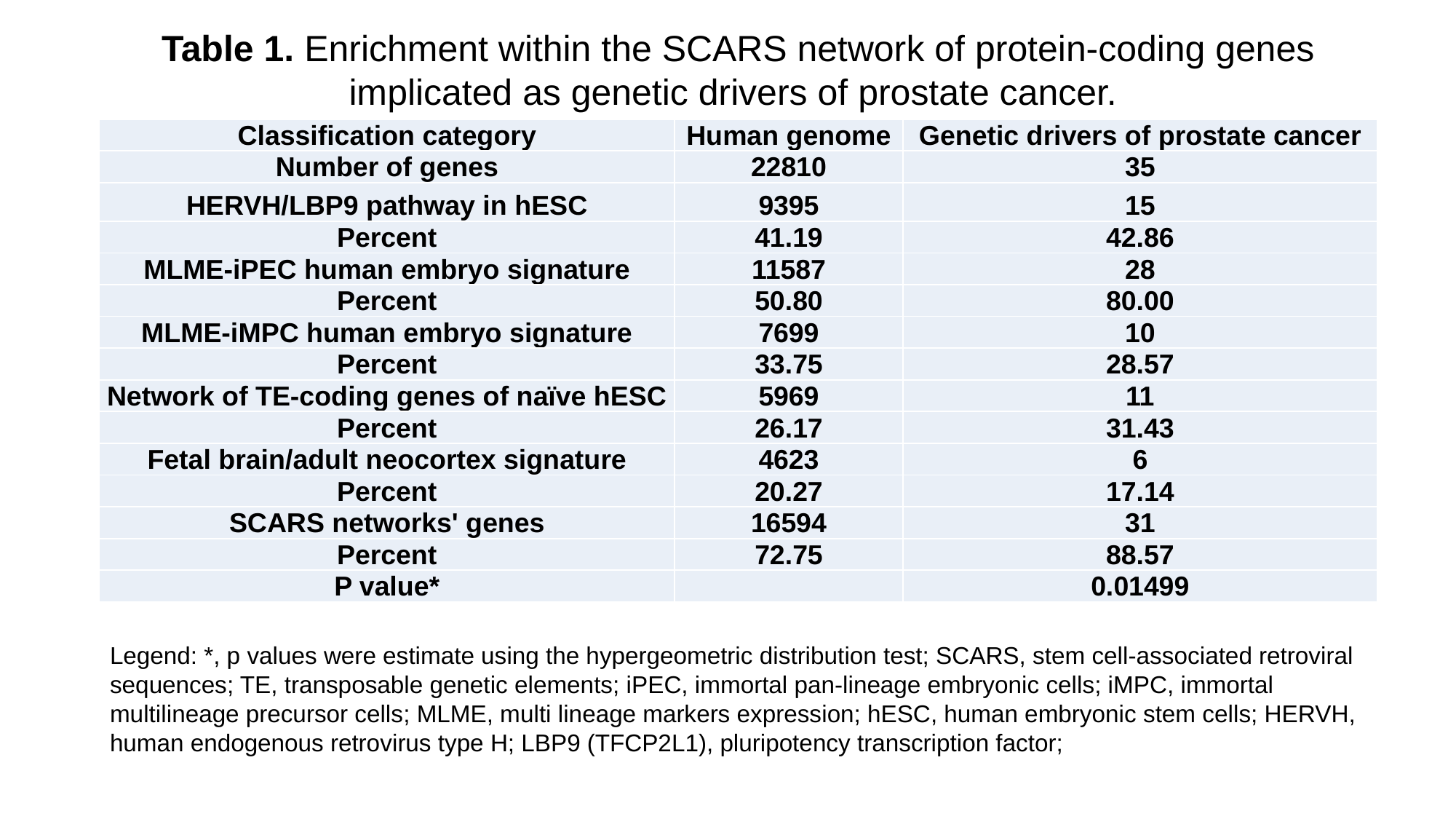

Table 1. Enrichment within the SCARS network of protein-coding genes implicated as genetic drivers of prostate cancer.
| Classification category | Human genome | Genetic drivers of prostate cancer |
| --- | --- | --- |
| Number of genes | 22810 | 35 |
| HERVH/LBP9 pathway in hESC | 9395 | 15 |
| Percent | 41.19 | 42.86 |
| MLME-iPEC human embryo signature | 11587 | 28 |
| Percent | 50.80 | 80.00 |
| MLME-iMPC human embryo signature | 7699 | 10 |
| Percent | 33.75 | 28.57 |
| Network of TE-coding genes of naïve hESC | 5969 | 11 |
| Percent | 26.17 | 31.43 |
| Fetal brain/adult neocortex signature | 4623 | 6 |
| Percent | 20.27 | 17.14 |
| SCARS networks' genes | 16594 | 31 |
| Percent | 72.75 | 88.57 |
| P value\* | | 0.01499 |
Legend: *, p values were estimate using the hypergeometric distribution test; SCARS, stem cell-associated retroviral sequences; TE, transposable genetic elements; iPEC, immortal pan-lineage embryonic cells; iMPC, immortal multilineage precursor cells; MLME, multi lineage markers expression; hESC, human embryonic stem cells; HERVH, human endogenous retrovirus type H; LBP9 (TFCP2L1), pluripotency transcription factor;

## Slide 15
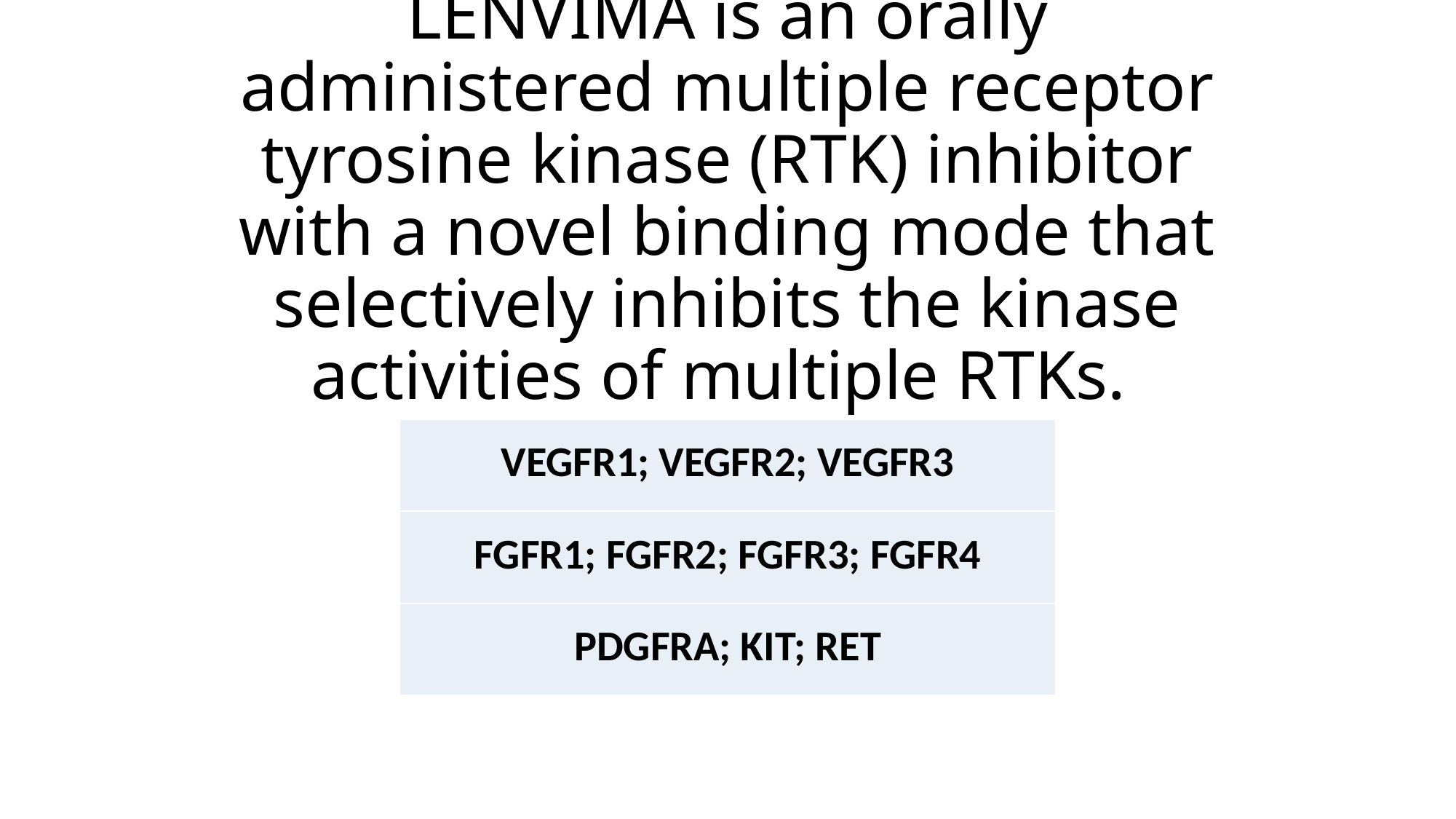

# LENVIMA is an orally administered multiple receptor tyrosine kinase (RTK) inhibitor with a novel binding mode that selectively inhibits the kinase activities of multiple RTKs.
| VEGFR1; VEGFR2; VEGFR3 |
| --- |
| FGFR1; FGFR2; FGFR3; FGFR4 |
| PDGFRA; KIT; RET |

## Slide 16
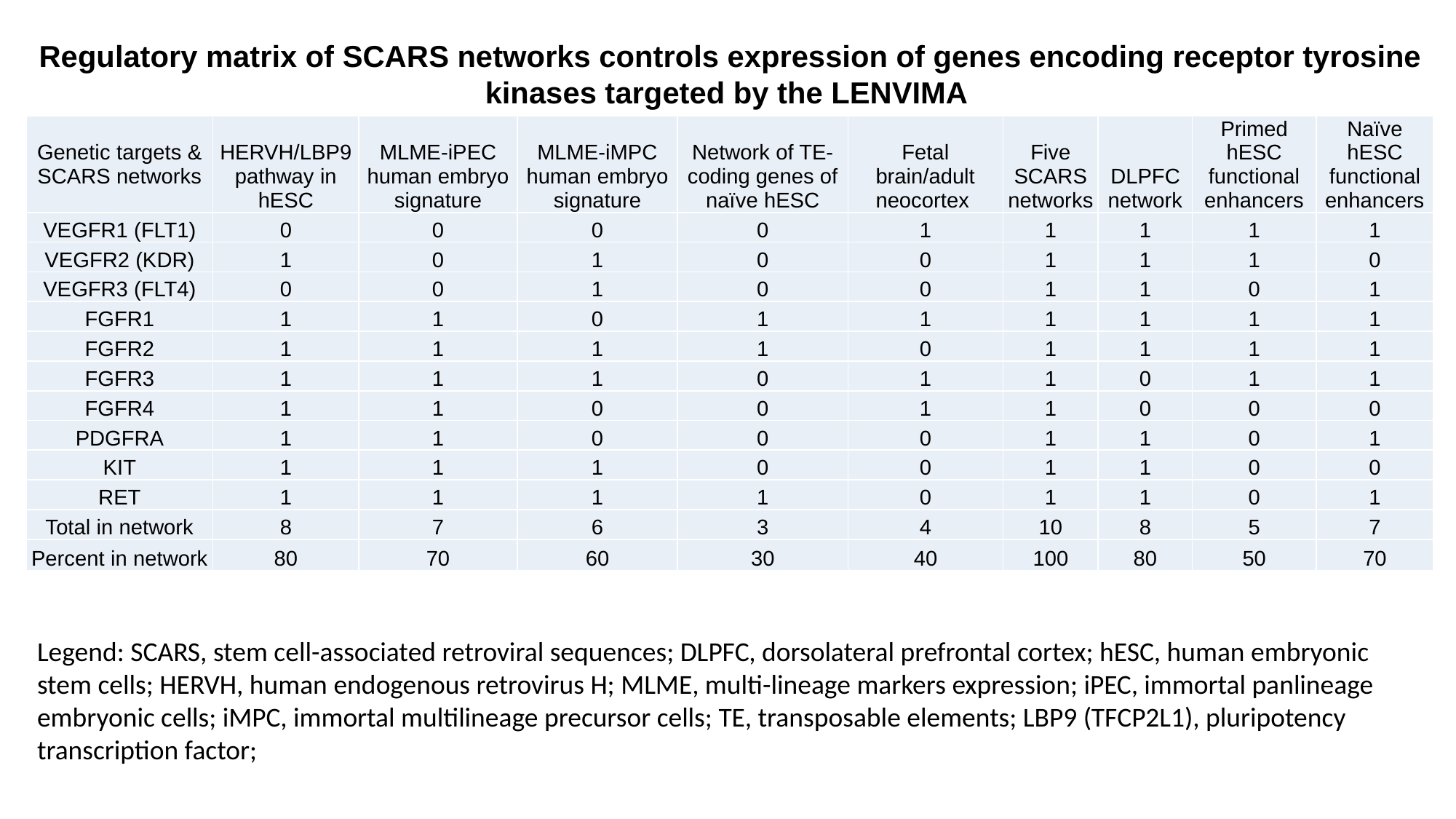

Regulatory matrix of SCARS networks controls expression of genes encoding receptor tyrosine kinases targeted by the LENVIMA
| Genetic targets & SCARS networks | HERVH/LBP9 pathway in hESC | MLME-iPEC human embryo signature | MLME-iMPC human embryo signature | Network of TE-coding genes of naïve hESC | Fetal brain/adult neocortex | Five SCARS networks | DLPFC network | Primed hESC functional enhancers | Naïve hESC functional enhancers |
| --- | --- | --- | --- | --- | --- | --- | --- | --- | --- |
| VEGFR1 (FLT1) | 0 | 0 | 0 | 0 | 1 | 1 | 1 | 1 | 1 |
| VEGFR2 (KDR) | 1 | 0 | 1 | 0 | 0 | 1 | 1 | 1 | 0 |
| VEGFR3 (FLT4) | 0 | 0 | 1 | 0 | 0 | 1 | 1 | 0 | 1 |
| FGFR1 | 1 | 1 | 0 | 1 | 1 | 1 | 1 | 1 | 1 |
| FGFR2 | 1 | 1 | 1 | 1 | 0 | 1 | 1 | 1 | 1 |
| FGFR3 | 1 | 1 | 1 | 0 | 1 | 1 | 0 | 1 | 1 |
| FGFR4 | 1 | 1 | 0 | 0 | 1 | 1 | 0 | 0 | 0 |
| PDGFRA | 1 | 1 | 0 | 0 | 0 | 1 | 1 | 0 | 1 |
| KIT | 1 | 1 | 1 | 0 | 0 | 1 | 1 | 0 | 0 |
| RET | 1 | 1 | 1 | 1 | 0 | 1 | 1 | 0 | 1 |
| Total in network | 8 | 7 | 6 | 3 | 4 | 10 | 8 | 5 | 7 |
| Percent in network | 80 | 70 | 60 | 30 | 40 | 100 | 80 | 50 | 70 |
Legend: SCARS, stem cell-associated retroviral sequences; DLPFC, dorsolateral prefrontal cortex; hESC, human embryonic stem cells; HERVH, human endogenous retrovirus H; MLME, multi-lineage markers expression; iPEC, immortal panlineage embryonic cells; iMPC, immortal multilineage precursor cells; TE, transposable elements; LBP9 (TFCP2L1), pluripotency transcription factor;
