# Supplementary material for: Genomics-Guided Drawing of Molecular and Pathophysiological Components of Malignant Regulatory Signatures Reveals a Pivotal Role in Human Diseases of Stem Cell-Associated Retroviral Sequences and Functionally-Active hESC Enhancers
Source: Front Oncol. 2021 Mar 31;11:638363. doi: 10.3389/fonc.2021.638363 (PMC8044830; doi:10.3389/fonc.2021.638363)
Supplement: Supplementary file 1 [file Presentation_1.zip › Supplemental Table S10..docx]

**Supplemental Table S10.** Enrichment within regulatory networks of Primed hESC functional enhancers (FE) of gene expression signatures (GES) defining human embryonic, neurodevelopmental, and cancer survival predictors' transcriptional networks.

| Classification category | Number of genes | Associated with FE | Percent | P value* | Observed/expected *** |
| --- | --- | --- | --- | --- | --- |
| Human genome | 63677 | 17131 | 26.9 |  |  |
| Fetal brain/adult neocortex signature | 4764 | 2347 | 49.3 | 1.49E-257 | 1.83 |
| TE/coding genes network of human DLPFC | 22863 | 8939 | 39.1 | 0 | 1.45 |
| MLME-iPEC human embryo signature | 12735 | 6140 | 48.2 | 0 | 1.79 |
| MLME-iMPC human embryo signature | 9251 | 3838 | 41.5 | 2.8878E-239 | 1.54 |
| Network of TE/coding genes of naïve hESC | 6265 | 3415 | 54.5 | 0 | 2.03 |
| HERVH/LBP9 pathway in hESC | 11507 | 4522 | 39.3 | 2.4064E-227 | 1.46 |
| Network of cancer survival predictor genes** | 10713 | 5622 | 52.5 | 4.2988E-270 | 1.95 |
| Human neuronal & non-neuronal brain cells signatures | 2072 | 1031 | 49.8 | 2.3862E-112 | 1.85 |
| Human neurons' sub-types & diversity signatures | 830 | 381 | 45.9 | 1.98913E-32 | 1.71 |

Legend: *, p values were estimate using the hypergeometric distribution test; **, only protein-coding genes were scored; ***, expected values were estimated based on the number of genes in the human genome (63,677) and the number of genes associated with functional enhancers of the primed hESC (17,131); FE, functional enhancers; TE, transposable genetic elements; DLPFC, dorsolateral prefrontal cortex; MLME-iPEC, multi-lineage markers expressing immortal pan-lineage embryonic cells; MLME-iMPC, multi-lineage markers expressing immortal multi-lineage precursor cells; MLME, multi-lineage markers expression; hESC, human embryonic stem cells; HERVH, human endogenous retrovirus type H; LBP9 (TFCP2L1), pluripotency transcription factor;
