# Supplementary material for: Genomics-Guided Drawing of Molecular and Pathophysiological Components of Malignant Regulatory Signatures Reveals a Pivotal Role in Human Diseases of Stem Cell-Associated Retroviral Sequences and Functionally-Active hESC Enhancers
Source: Front Oncol. 2021 Mar 31;11:638363. doi: 10.3389/fonc.2021.638363 (PMC8044830; doi:10.3389/fonc.2021.638363)
Supplement: Supplementary file 1 [file Presentation_1.zip › Supplemental Table S11. Neocortex HSGRNs.docx]

**Supplemental Table S11.** Enrichment within human-specific regulatory networks of genes comprising fetal brain/adult neocortex signature.

**Networks of genes associated with expression of transposable elements (TE) in human dorsolateral prefrontal cortex**

| Classification category | Number of genes | Human fetal brain/adult neocortex genes |
| --- | --- | --- |
| Human genome | 63677 | 4764 |
| Networks of genes associated with human DLPFC-expressed TE | 22863 | 4010 |
| Percent | 35.90 | 84.17 |
| Enrichment** | 1.00 | 2.34 |
| P value* |  | 0 |

| Classification category | Number of genes | Human fetal brain/adult neocortex genes |
| --- | --- | --- |
| Human genome | 63677 | 4764 |
| GES of the MLME cells of human preimplantation embryo | 12735 | 3060 |
| Percent | 20.00 | 64.23 |
| Enrichment** | 1.00 | 3.21 |
| P value* |  | 0 |

**GES of the Multi-lineage Markers Expressing (MLME) cells of human preimplantation embryo**

**Regulatory networks of genes associated with human-specific structural variants*****

| Classification category | Number of genes | Human fetal brain/adult neocortex genes |
| --- | --- | --- |
| Human genome | 63677 | 4764 |
| Genes associated with human-specific deletions and insertions | 10992 | 1495 |
| Percent | 17.26 | 31.38 |
| Enrichment** | 1.00 | 1.82 |
| P value* |  | 6.5262E-137 |

**Gene expression signature of the HERVH/LBP9 network in hESC**

| Classification category | Number of genes | Human fetal brain/adult neocortex genes |
| --- | --- | --- |
| Human genome | 63677 | 4764 |
| Genes associated with the HERVH/LBP9 pathway in hESC | 11507 | 2346 |
| Percent | 18.07 | 49.24 |
| Enrichment** | 1.00 | 2.73 |
| P value* |  | 0 |

**Network of genes associated with regulatory TE in naïve & primed hESC**

| Classification category | Number of genes | Human fetal brain/adult neocortex genes |
| --- | --- | --- |
| Human genome | 63677 | 4764 |
| Genes associated with regulatory TE in naïve & primed hESC | 6148 | 1658 |
| Percent | 9.65 | 34.80 |
| Enrichment** | 1.00 | 3.61 |
| P value* |  | 0 |

**Network of genes associated with naïve & primed hESC functional enhancers**

| Classification category | Number of genes | Human fetal brain/adult neocortex genes |
| --- | --- | --- |
| Human genome | 63677 | 4764 |
| Genes associated with naïve & primed hESC functional enhancers | 25241 | 3244 |
| Percent | 39.64 | 68.09 |
| Enrichment** | 1.00 | 1.72 |
| P value* |  | 0 |

Legend: *, p values were estimate using the hypergeometric distribution test; **, expected values were estimated based on the number of genes in the human genome (63,677) and the number of genes in the corresponding category of human-specific regulatory networks; ***, this category of genes was reported in Kronenberg et al. (2018); TE, transposable genetic elements; hESC, human embryonic stem cells; DLPFC, dorsolateral prefrontal cortex; MLME, multi lineage markers expression; Human fetal brain/adult neocortex GES has been reported in [35].
