# Supplementary material for: Genomics-Guided Drawing of Molecular and Pathophysiological Components of Malignant Regulatory Signatures Reveals a Pivotal Role in Human Diseases of Stem Cell-Associated Retroviral Sequences and Functionally-Active hESC Enhancers
Source: Front Oncol. 2021 Mar 31;11:638363. doi: 10.3389/fonc.2021.638363 (PMC8044830; doi:10.3389/fonc.2021.638363)
Supplement: Supplementary file 1 [file Presentation_1.zip › Supplemental Table S12. Neocortex FE hESC.docx]

**Supplemental Table S12.** Enrichment within regulatory networks of Naïve and Primed hESC functional enhancers of human fetal brain/adult neocortex signature genes.

| Naïve hESC functional enhancers |  |  |  |  |  |
| --- | --- | --- | --- | --- | --- |
| Classification category | **Number of genes** | **Number of genes associated with functional enhancers** | **Percent** | **P value*** | **Observed/ expected ratio**** |
| Human genome | 63677 | 18766 | 29.5 |  |  |
| Fetal brain/adult neocortex signature | 4764 | 2513 | 52.7 | 1.7E-268 | 1.79 |
| Primed hESC functional enhancers |  |  |  |  |  |
| Classification category | **Number of genes** | **Number of genes associated with functional enhancers** | **Percent** | **P value*** | **Observed/ expected ratio**** |
| Human genome | 63677 | 17131 | 26.9 |  |  |
| Fetal brain/adult neocortex signature | 4764 | 2347 | 49.3 | 1.5E-257 | 1.83 |
| Naïve & Primed hESC functional enhancers |  |  |  |  |  |
| Classification category | **Number of genes** | **Number of genes associated with functional enhancers** | **Percent** | **P value*** | **Observed/ expected ratio**** |
| Human genome | 63677 | 25241 | 39.6 |  |  |
| Fetal brain/adult neocortex signature | 4764 | 3244 | 68.1 | 0 | 1.72 |

Legend: *, p values were estimate using the hypergeometric distribution test; **, expected values were estimated based on the number of genes in the human genome (63,677); number of genes associated with functional enhancers of the Naïve hESC (18,766); number of genes associated with functional enhancers of the Primed hESC (17,131); number of genes associated with functional enhancers of both Naive and Primed hESC (25,421); and number of genes comprising human fetal brain/adult neocortex signature (4,764); Human fetal brain/adult neocortex gene expression signature was reported in [35].
