# Supplementary material for: Genomics-Guided Drawing of Molecular and Pathophysiological Components of Malignant Regulatory Signatures Reveals a Pivotal Role in Human Diseases of Stem Cell-Associated Retroviral Sequences and Functionally-Active hESC Enhancers
Source: Front Oncol. 2021 Mar 31;11:638363. doi: 10.3389/fonc.2021.638363 (PMC8044830; doi:10.3389/fonc.2021.638363)
Supplement: Supplementary file 1 [file Presentation_1.zip › Supplemental Table S9..docx]

**Supplemental Table S9.** Enrichment within regulatory networks of Naïve hESC functional enhancers of gene expression signatures (GES) defining human embryonic, neurodevelopmental, and cancer survival predictors' transcriptional networks.

| Classification category | Number of genes | Associated with FE | Percent | P value* | Observed/expected *** |
| --- | --- | --- | --- | --- | --- |
| Human genome | 63677 | 18766 | 29.5 |  |  |
| Fetal brain/adult neocortex signature | 4764 | 2513 | 52.7 | 1.6953E-268 | 1.79 |
| TE/coding genes network of human DLPFC | 22863 | 9634 | 42.1 | 0 | 1.43 |
| MLME-iPEC human embryo signature | 12735 | 6443 | 50.6 | 0 | 1.72 |
| MLME-iMPC human embryo signature | 9251 | 4149 | 44.8 | 5.7515E-254 | 1.52 |
| Network of TE/coding genes of naïve hESC | 6265 | 3609 | 57.6 | 0 | 1.95 |
| HERVH/LBP9 network in hESC | 11507 | 4809 | 41.8 | 7.8989E-215 | 1.42 |
| Network of cancer survival predictor genes** | 10713 | 5972 | 55.7 | 2.8484E-259 | 1.89 |
| Human neuronal & non-neuronal brain cells signatures | 2072 | 1082 | 52.2 | 1.2015E-107 | 1.77 |
| Human neurons' sub-types & diversity signatures | 830 | 410 | 49.4 | 3.84147E-34 | 1.67 |

Legend: *, p values were estimate using the hypergeometric distribution test; **, only protein-coding genes were scored; ***, expected values were estimated based on the number of genes in the human genome (63,677) and the number of genes associated with functional enhancers of the Naive hESC (18,766); FE, functional enhancers; TE, transposable genetic elements; DLPFC, dorsolateral prefrontal cortex; MLME-iPEC, multi-lineage markers expressing immortal pan-lineage embryonic cells; MLME-iMPC, multi-lineage markers expressing immortal multi-lineage precursor cells; MLME, multi-lineage markers expression; hESC, human embryonic stem cells; HERVH, human endogenous retrovirus type H; LBP9 (TFCP2L1), pluripotency transcription factor;
